# Diastereodivergent asymmetric Michael-alkylation reactions using chiral *N*,*N*′-dioxide/metal complexes†
†Electronic supplementary information (ESI) available. CCDC 1411858, 1412691 and 1545249. For ESI and crystallographic data in CIF or other electronic format see DOI: 10.1039/c7sc02757e


**DOI:** 10.1039/c7sc02757e

**Published:** 2017-11-08

**Authors:** Yulong Kuang, Bin Shen, Li Dai, Qian Yao, Xiaohua Liu, Lili Lin, Xiaoming Feng

**Affiliations:** a Key Laboratory of Green Chemistry & Technology , Ministry of Education , College of Chemistry , Sichuan University , Chengdu 610064 , China . Email: liuxh@scu.edu.cn ; Email: xmfeng@scu.edu.cn ; Fax: +86 28 85418249 ; Tel: +86 28 85418249; b Collaborative Innovation Center of Chemical Science and Engineering (Tianjin) , P. R. China

## Abstract

A diastereodivergent asymmetric Michael-alkylation reaction between 3-chloro-oxindoles and β,γ-unsaturated-α-ketoesters has been achieved using **L-RaPr_2_**/Sc(OTf)_3_ and **L-PrPr_2_**/Mg(OTf)_2_ metal complexes as catalysts. Both *rel*-(1*R*,2*S*,3*R*) and *rel*-(1*S*,2*S*,3*R*) chiral spiro cyclopropane oxindoles were constructed in good yields, diastereoselectivities and ee values. The diastereodivergent control may originate from different alkylation pathways after the Michael addition, with either intramolecular trapping of the aza-*ortho*-xylylene intermediate or direct S_N_2 substitution.

## Abstract 2

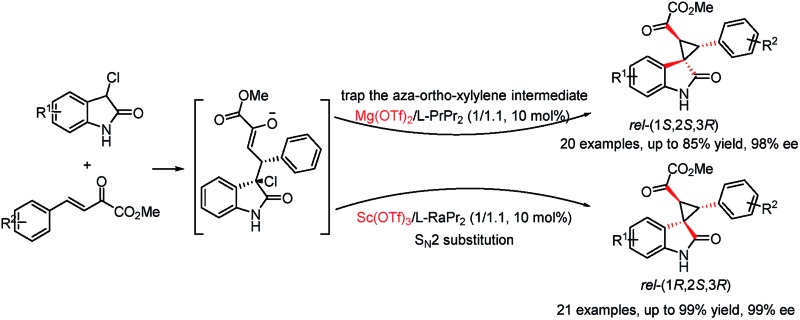
A diastereodivergent asymmetric cascade Michael-alkylation by switching the alkylation step of intramolecular trapping of the aza-*ortho*-xylylene intermediate or direct S_N_2 substitution has been developed.

## 

Tuning diastereoselectivity in catalytic asymmetric synthesis is challenging due to the inherent preference for forming one type of diastereomer in most reactions.^1^ However, relative configurations are as important as absolute configurations in pharmacology and drug discovery because both can influence the physiological activity of a molecule.^2^ In general, diastereodivergence^3^ can be realized by changing the catalyst,^4^ additive and solvent,^5^ substrate,^6^ and other methods. Nevertheless, diastereodivergent synthesis is still in its infancy. It’s desirable to develop new strategies and discover more diastereodivergent reactions.

Oxindoles with a unique spirocyclopropane moiety exhibit diverse biological activities, such as non-nucleoside reverse transcriptase inhibitor and antitumor activity.^7^ Among the synthetic methodologies to prepare these molecules,^8^ the cascade Michael-alkylation reactions^9^ of 3-chlorooxindole with α,β-unsaturated olefins provide an operationally simple, stepwise pathway for diastereodivergence. Currently, only organic proline-based silyl ethers,^10^ cinchona alkaloid-derived thioureas^11^ and squaramide^12^ catalysts have been developed. These reactions proceed *via* the intramolecular trapping of the chiral aza-*ortho*-xylylene intermediate, **A**, after the Michael addition to afford the thermodynamically favored *rel*-(1*S*,2*S*,3*R*)^13^ products (Scheme 1, path a). On the other hand, if the Michael addition products follow the direct S_N_2 substitution pathway (intermediate **B**), the *rel*-(1*R*,2*S*,3*R*) products will be formed (Scheme 1, path b). To the best of our knowledge, there is no precedent for synthesizing the *rel*-(1*R*,2*S*,3*R*) products as the major diastereomer, much less synthesizing both *rel*-(1*S*,2*S*,3*R*) and *rel*-(1*R*,2*S*,3*R*) products in high efficiency without drastically changing the reaction conditions. Herein, we reported a diastereodivergent asymmetric Michael-alkylation reaction between 3-chloro-oxindoles and β,γ-unsaturated-α-ketoesters using chiral *N*,*N*′-dioxide/metal complexes,^14^ synthesizing *rel*-(1*S*,2*S*,3*R*) and *rel*-(1*R*,2*S*,3*R*) spirocyclopropane oxindoles in high yields, diastereoselectivities and enantioselectivities.

**Scheme 1:**
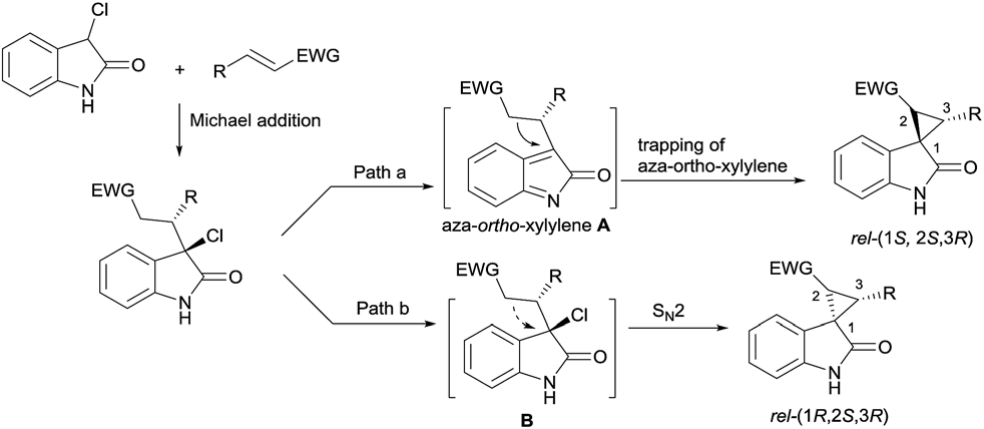
Proposed processes for the diastereodivergent Michael-alkylation.

Initially, the cascade reaction of 3-Cl oxindole, **1a**, with the β,γ-unsaturated-α-ketoester **2a** was chosen as the model reaction to optimize the reaction conditions. First, a series of metal salts were investigated by complexing with chiral *N*,*N*′-dioxide **L-PrPr_2_** in the presence of Na_2_CO_3_ as the base at 30 °C. It was found that both Sc(OTf)_3_ and Mg(OTf)_2_ complexes could catalyze the reaction with a preference for forming a different diastereomer. The enantioselectivity was moderate for each major diastereomer, which could be isolated by column chromatography. Next, other conditions were screened.^15^ The **L-RaPr_2_**/Sc(OTf)_3_ complex elevated the isolated yield of the product *rel*-(1*R*,2*S*,3*R*)-**3aa** to 92% with 78% ee (entry 3). After lowering the reaction temperature to 0 °C, increasing the stoichiometry of the base to 1.3 equivalents, and prolonging the reaction time to 72 h, the *rel*-(1*R*,2*S*,3*R*)-**3aa** could be obtained in 97% yield and 92% ee (entry 4, Table 1).

**Table 1:** Optimization of the reaction conditions^*a*^

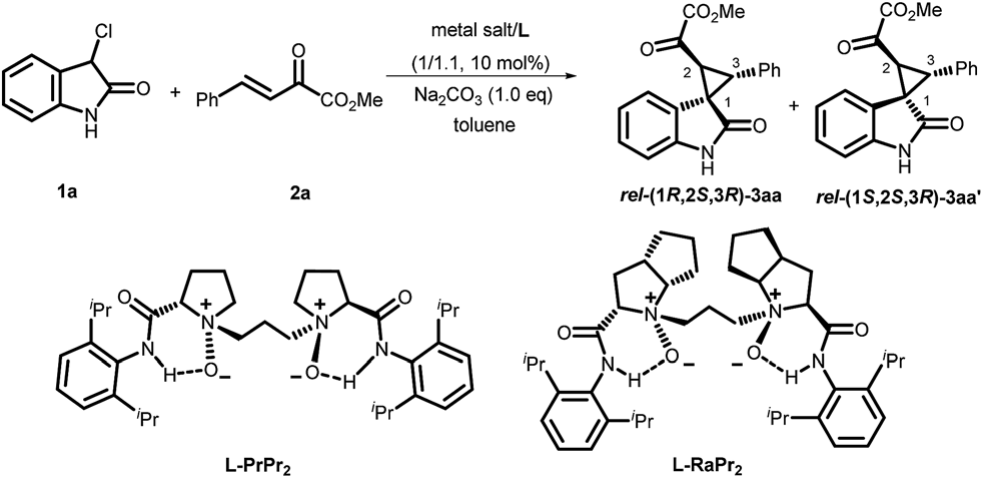				
Entry	Metal salt	L*	Yield^*b*^ (**3aa**/**3aa′**) (%)	ee^*c*^ (**3aa**/**3aa′**)
1	Sc(OTf)_3_	**L-PrPr**_2_	92/7	76/0
2	Mg(OTf)_2_	**L-PrPr**_2_	14/28	0/88
3	Sc(OTf)_3_	**L-RaPr**_2_	92/—	78/—
4^*d*^	Sc(OTf)_3_	**L-RaPr**_2_	97/—	92/—
5^*e*^	Mg(OTf)_2_	**L-PrPr**_2_	—/77	—/95

^*a*^Unless otherwise noted, the reaction proceeded with **1a** (0.1 mmol), **2a** (0.1 mmol), metal salt/ligand (1 : 1.1, 10 mol%), and Na_2_CO_3_ (1.0 equiv.) in toluene (1.0 mL) at 30 °C for 24 h.

^*b*^Isolated yield.

^*c*^Determined by chiral HPLC on a chiral stationary phase (Chiralcel IA and IE).

^*d*^The reaction proceeded at 0 °C for 72 h and with 1.3 eq. of Na_2_CO_3_.

^*e*^At 40 °C for 72 h.

On the other hand, using chiral **L-PrPr_2_**/Mg(OTf)_2_ as the catalyst and increasing the reaction temperature to 40 °C, the corresponding *rel*-(1*S*,2*S*,3*R*)-**3aa′** could be afforded in 77% yield and 95% ee after 72 hours (entry 5). It is worth mentioning that the metal cations dictate the diastereoselectivity and variation of the chiral ligand structure and the reaction temperature did not change the major diastereoisomer once Sc(OTf)_3_ or Mg(OTf)_2_ were identified as suitable catalysts. The absolute configurations of the major enantiomers were determined separately by X-ray crystallographic analysis of the corresponding *N*-Boc protected derivatives **5** 16 (Fig. 1). Through this method, *rel*-(1*S*,2*S*,3*R*)-**5aa′** obtained from the **L-PrPr_2_**/Mg(OTf)_2_ catalytic system was determined to be (1*S*, 2*S*, 3*R*), and *rel*-(1*R*,2*S*,3*R*)-**5aa** generated from **L-RaPr_2_**/Sc(OTf)_3_ was found to be (1*R*, 2*S*, 3*R*).

**Fig. 1:**
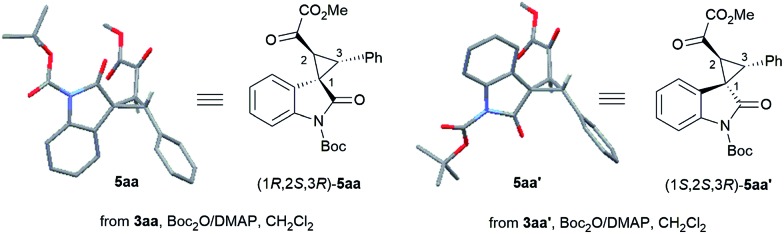
A wire-stick representation of the *N*-Boc derivatives **5aa^(^′^)^** from the products **3aa^(^′^)^**.

Next, the enantioselective and diastereodivergent synthesis of a series of spirocyclopropane oxindoles was carried out using these two chiral catalyst systems (Table 2). Under the optimized conditions, all of the substrates gave one major diastereomer (higher than 94:6 diastereoselectivity) with moderate to excellent yields and enantioselectivities. It is worth noting that most of the *rel*-(1*R*,2*S*,3*R*) products **3** were unstable under the HPLC analysis conditions. Fortunately, the enantiomeric excess could be determined after conversion of *rel*-(1*R*,2*S*,3*R*)-**3** into the corresponding derivatives *rel*-(1*R*,2*S*,3*R*)-**4** (Scheme 2). Generally, the *rel*-(1*S*,2*S*,3*R*)-diastereoisomers prepared from the **L-PrPr_2_**/Mg(OTf)_2_ catalyst were delivered in higher enantioselectivities than the *rel*-(1*R*,2*S*,3*R*) isomers from the **L-RaPr_2_**/Sc(OTf)_3_ catalyst. For the synthesis of *rel*-(1*S*,2*S*,3*R*) cyclopropanes, 3-chlorooxindoles **1** with halo-substituents at the C4 and C5-positions gave higher enantioselectivities than C6-substituted ones (entries 3–8). Electron-donating or -withdrawing substituents on the aromatic β,γ-unsaturated-α-ketoester **2** had a slight influence on the enantioselectivity (entries 10–22). 2-Naphthyl- and 2-thiophenyl substituted β,γ-unsaturated-α-ketoesters were also tolerated well (entries 21 and 22). Moreover, when the aliphatic substrates **2t** and **2u** were subjected to the reaction conditions, the corresponding *rel*-(1*R*,2*S*,3*R*)-products could be obtained in excellent yields with excellent enantioselectivities (up to 99% yield and 99% ee; entries 23 and 24).

**Table 2:** Substrate scope of the diastereodivergent Michael-alkylation reaction^*a*^

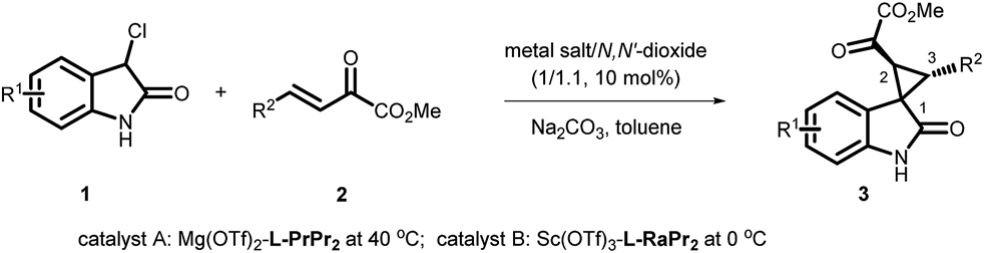					
Entry	**3**: *R*^1^; *R*^2^	*rel*-(1*S*,2*S*,3*R*)-**3′** (Cat A)		*rel*-(1*R*,2*S*,3*R*)-3 (Cat B)	
		Yield^*b*^ (%)	ee^*c*^ (%)	Yield^*b*^ (%)	ee^*d*^ (%)
1	**3aa^(^′^)^**: H; C_6_H_5_	77	95	97	92
2	**3ba^(^′^)^**: 4-Me; C_6_H_5_	—	—	67	86
3	**3ca^(^′^)^**: 4-F; C_6_H_5_	84	96	76	86
4	**3da^(^′^)^**: 5-Me; C_6_H_5_	72	96	96	89
5	**3ea^(^′^)^**: 5-F; C_6_H_5_	67	92	96	81
6	**3fa^(^′^)^**: 5-Cl; C_6_H_5_	61	88	—	—
7	**3ga^(^′^)^**: 6-F; C_6_H_5_	70	89	91	90
8	**3ha^(^′^)^**: 6-Cl; C_6_H_5_	63	84	98	88
9	**3ia^(^′^)^**: 6-Br; C_6_H_5_	—	—	83	84
10	**3ah^(^′^)^**: H; 2-MeOC_6_H_4_	69^*e*^	95^*e*^	71	91
11	**3ai^(^′^)^**: H; 3-MeOC_6_H_4_	70	96	98	95
12	**3aj^(^′^)^**: H; 4-MeOC_6_H_4_	59	94	96	72
13	**3ak^(^′^)^**: H; 2-MeC_6_H_4_	50	91	76	96
14	**3al^(^′^)^**: H; 4-MeC_6_H_4_	77	93	93	87
15	**3av^(^′^)^**: H; 4-FC_6_H_4_	—	—	96	90
16	**3am^(^′^)^**: H; 3-ClC_6_H_4_	77	92	76	87
17	**3an^(^′^)^**: H; 4-ClC_6_H_4_	63	91	79	93
18	**3ao^(^′^)^**: H; 2-BrC_6_H_4_	62	94	—	—
19	**3ap^(^′^)^**: H; 4-BrC_6_H_4_	81	94	83	94
20	**3aq^(^′^)^**: H; 4-PhC_6_H_4_	74	98	96	92
21	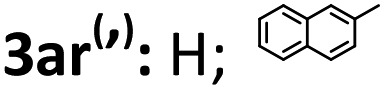	71	93	91	93
22	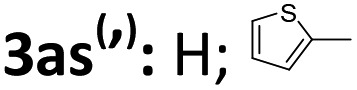	52	95	—	—
23	**3at^(^′^)^**: H; ^*n*^Bu	71	93	95	99
24	**3au^(^′^)^**: H; cyclohexyl	—	—	99	99

^*a*^Unless otherwise noted, reactions were performed with **1** (0.1 mmol), **2** (0.1 mmol), chiral catalyst (10 mol%) and Na_2_CO_3_ in toluene (1.0 mL) for 72 h. For *rel*-(1*S*,2*S*,3*R*)-**3′**, **L-PrPr_2_**/Mg(OTf)_2_ (1.1/1) and 1.0 eq. of Na_2_CO_3_ were used at 40 °C. For *rel*-(1*R*,2*S*,3*R*)-**3**, **L-RaPr_2_**/Sc(OTf)_3_ (1.1/1, 10 mol%) and 1.3 eq. of Na_2_CO_3_ were used at 0 °C.

^*b*^Isolated yield.

^*c*^Determined by HPLC on a chiral stationary phase.

^*d*^Determined by HPLC on a chiral stationary phase after transformation into **4**.

^*e*^The diastereoselectivity of **3ah** was determined to be 94:6 from HPLC.

**Scheme 2:**
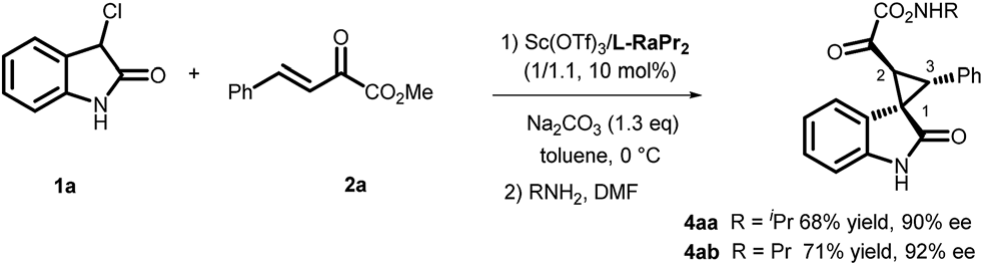
The stabilization of spiro-cyclopropane oxindole by amination.

Preliminary mechanistic studies were conducted to confirm our proposed diastereodivergent control mode. The relationship between the ee values of the ligand and product showed a linear correlation.^15^ Additionally, X-ray crystallographic analysis of the catalysts also showed a 1 : 1 ratio of ligand to metal.^17^ Both of these findings imply that the monomeric catalysts might be the main catalytically active species. What’s more, crystal structures of both catalysts displayed similar geometries and showed no significant differences in the accessibility of the substrate’s coordination site. To check whether **L-PrPr_2_**/Mg(OTf)_2_ and **L-RaPr_2_**/Sc(OTf)_3_ coordinated with different substrates, *in situ* HRMS analysis was performed.^15^ Based on the HRMS spectra, both of them coordinate with the same substrate, 3-Cl oxindole, to initiate the reaction. All of these experiments excluded the possibility that the observed diastereodivergence resulted from different coordinative styles of the two catalysts.

The intermediates of the Michael addition products were then synthesized and subjected to the optimized reaction conditions. The relative configuration of **6aa** was also confirmed by X-ray crystallographic analysis.^18^ As summarized in Table 3, the two diastereomers of **6** only transformed to the same **3aa′** with 76% yield and 31% yield, respectively, under the **L-PrPr_2_**/Mg(OTf)_2_ system (Table 3, entries 2 and 4), which may be due to the chiral match or mismatch effect. On the contrary, **3aa** or **3aa′** could be afforded from either **6aa** or **6aa′** in the **L-RaPr_2_**/Sc(OTf)_3_ system with 18% yield and 74% yield, respectively (Table 3, entries 1 and 3). By comparing the different results between the **L-RaPr_2_**/Sc(OTf)_3_ and **L-PrPr_2_**/Mg(OTf)_2_ catalyzed reactions, **L-PrPr_2_**/Mg(OTf)_2_ should promote this reaction with aza-*ortho*-xylylene intermediates, as has been reported previously, which induced thermodynamically favored *rel*-(1*S*,2*S*,3*R*) **3aa′** from different diastereomers of **6**. However, in the **L-RaPr_2_**/Sc(OTf)_3_ catalytic system, the chirality inversion at the quaternary carbon from **6aa** to *rel*-(1*R*,2*S*,3*R*) **3aa** and from **6aa′** to *rel*-(1*S*,2*S*,3*R*) **3aa′** illustrated that alkylation proceeded through a direct S_N_2 substitution pathway. Moreover, chiral **6aa** (–73% ee) could produce another enantiomer of **3aa′** in 75% yield and 76% ee under the **L-PrPr_2_**/Mg(OTf)_2_ conditions (Table 3, entry 5). Undoubtedly, the same enantioselectivity revealed that the first Michael addition step should be the chirality-determining step. For the result of entry 1, the product **3aa** was formed with 85% ee and the starting material was recovered in 80% yield with 84:16 diastereoselectivity (**6aa**:**6aa′**) from racemic **6aa** under the **L-RaPr_2_**/Sc(OTf)_3_ catalytic system (Table 3, entry 1). We propose that this result came from the chemical equilibrium between the retro-Michael reaction and Michael addition, which could also account for the lack of formation of *rel*-(1*S*,2*S*,3*R*) **3aa′**. The low yield also implied that the rate of S_N_2 substitution from the enolate ion is high and Na_2_CO_3_ is not a strong enough base to deprotonate the Michael intermediate. To evaluate the role of the N–H group, *N*-methylated 3-Cl oxindole was also subjected into the reaction, which showed a poor result.^15^

**Table 3:** The comparative experiments for diastereodivergent control

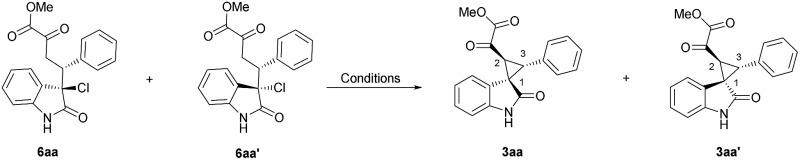				
Entry	**6**	Condition^*a*^	**3aa** ^*b*^ [yield, ee]	**3aa′** ^*b*^ [yield, ee]
1^*c*^	(±) **6aa**	**L-RaPr_2_**/Sc(OTf)_3_	18%, 85%	—
2	(±) **6aa**	**L-PrPr_2_**/Mg(OTf)_2_	—	76%, –7%
3	(±) **6aa′**	**L-RaPr_2_**/Sc(OTf)_3_	—	74%, 3%
4	(±) **6aa′**	**L-PrPr_2_**/Mg(OTf)_2_	—	31%, race
5^*d*^	(+) **6aa**	**L-PrPr_2_**/Mg(OTf)_2_	—	75%, –76%

^*a*^Unless otherwise noted, reactions were performed with corresponding **6** and catalyst (M/L = 1/1.1 10 mol%) and Na_2_CO_3_ (1.0 eq.) in toluene (1.0 mL) at 0 °C for 72 h.

^*b*^Isolated yield. ee was determined by HPLC on a chiral stationary phase.

^*c*^The recovered **6aa** had a yield of 80%, 84:16 dr, and –17% ee/19% ee, and the diastereoselectivity of corresponding **3aa** was 84:16.

^*d*^The ee of **6aa** was –73% ee.

## Conclusions

A diastereodivergent asymmetric Michael-alkylation reaction between 3-Cl oxindoles and β,γ-unsaturated-α-ketoesters was accomplished by tuning metal catalysts and adjusting the ligands and temperature. Under the optimized conditions, both *rel*-(1*R*,2*S*,3*R*) and *rel*-(1*S*,2*S*,3*R*) spiro cyclopropane oxindoles were synthesized with high yields, diastereoselectivities and enantioselectivities. Mechanistic studies also revealed that the diastereodivergent control should come from either trapping the aza-*ortho*-xylylene intermediates or direct S_N_2 substitution in the alkylation step, which may be caused by the different characteristics of the metal catalysts. Developing other diastereodivergent asymmetric methodologies with this strategy is ongoing.

## Conflicts of interest

There are no conflicts to declare.

## Supplementary Material

Supplementary information

Crystal structure data
