# Supplementary material for: Diastereodivergent asymmetric Michael-alkylation reactions using chiral N,N′-dioxide/metal complexes
Source: Chem Sci. 2017 Nov 8;9(3):688–92. doi: 10.1039/c7sc02757e (PMC5869575; doi:10.1039/c7sc02757e)
Supplement: Supplementary file 1 [file SC-009-C7SC02757E-s001.pdf]

Electronic Supplementary Material (ESI) for Chemical Science.
This journal is © The Royal Society of Chemistry 2017

Supporting Information

Diastereodivergent Asymmetric Michael-Alkylation Reaction by Chiral *N,N'*-Dioxide/Metal Complexes

Yulong Kuang,[b] Bin Shen,[b] Li Dai, Qian Yao, Xiaohua Liu, Lili Lin, Xiaoming Feng*[a]

Key Laboratory of Green Chemistry & Technology, Ministry of Education, College of Chemistry, Sichuan University, Chengdu 610064, People's Republic of China

Contents

1. GENERAL REMAKES............ 1

2 SELECTED RESULTS OF OPTIMIZATION OF THE REACTION CONDITIONS............ 1

1) The summarization of the reaction condition............ 1

2) Selected examples of the optimization of the ligands............ 2

3) Selected examples of optimization of the temperature............ 3

3 GENERAL PROCEDURE............ 4

4 DETERMINATION OF ABSOLUTE CONFIGURATION OF 3AA............ 5

5 STUDIES OF THE MECHANISM............ 6

5.1 Nonlinear effect............ 6

5.2 The ORTEP diagramme of catalyst species............ 7

5.3 HRMS analysis for the coordinative type............ 7

5.4 Comparative experiments............ 8

6 CHARACTERIZATION OF THE PRODUCTS............ 9

7 REFERENCE............ 44

8 COPIES OF THE CD SPECTRA............ 44

9 COPIES OF THE NMR SPECTRA............ 51

1. General Remakes

Reactions were carried out with commercial available reagent in dried apparatus. The THF and toluene were pretreated under the potassium hydroxide, and then distilled from the sodium benzophenone under nitrogen atmosphere before use. The yield was the purified state by flash chromatography in silica gel. Enantiomeric excesses were determined by HPLC analysis using the corresponding commercial chiral column (chiralcel ID, IA, IE column) as stated in the experimental procedures at 23 °C with the UV detector at 254 nm. Optical rotation were reported as follows: $[\alpha]^{T}_{D}$ (c g/100 mL, in solvent). The melting points of sprio-cyclo-propane-oxindoles were determined by melting instrument. ^{1}H NMR spectra were recorded on commercial instruments (400 MHz). Chemical shifts were reported in ppm from tetramethylsilane with the solvent resonance as the internal standard ($CDCl_3$, δ = 7.26). Spectra were reported as follows: chemical shift (δ ppm), multiplicity (s = singlet, d = doublet, t = triplet, q = quartet, hept = heptet, m = multiplet), coupling constants (Hz), integration and assignment. ^{13}C NMR spectra were recorded on commercial instruments (100 MHz). Chemical shift were reported in ppm from tetramethylsilane with the solvent resonance as the internal standard ($CDCl_3$, δ = 77). HRMS was recorded on a commercial apparatus (ESI Source).

3-Chloride substituted oxindoles **1**[1] and the substituted β,γ-unsaturated-α-ketonesters **2**[2] were prepared from the procedure. TLC was performed on glass-backed silica plates. Chiral *N,N'*-dioxide ligands were prepared according to the methods mentioned in catalyst preparation section[3].

2 Selected Results of Optimization

1) The summarization of the reaction condition

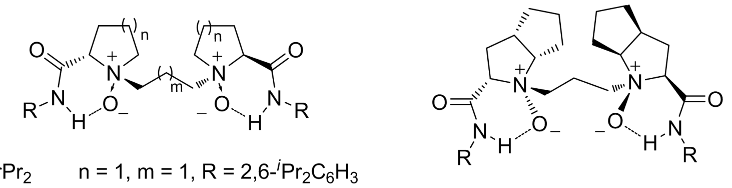

L-PrPr$_2$ n = 1, m = 1, R = 2,6-^{i}Pr$_2$C$_6$H$_3$
L-PrEt$_2$*p*Me n = 1, m = 1, R = 2,6-^{i}Pr$_2$-4-Me-C$_6$H$_2$
L-PrPr$_3$ n = 1, m = 1, R = 2,4,6-^{i}Pr$_3$C$_6$H$_2$
L$_2$-PrPr$_2$ n = 1, m = 0, R = 2,6-^{i}Pr$_2$C$_6$H$_3$
L-PrAd n = 1, m = 1, R = 1-Admantyl
L-PiPr$_2$ n = 2, m = 1, R = 2,6-^{i}Pr$_2$C$_6$H$_3$

L-RaPr$_2$ R = 2,6-^{i}Pr$_2$C$_6$H$_3$
L-RaPr$_3$ R = 2,4,6-^{i}Pr$_3$C$_6$H$_2$
L-RaEt$_2$ R = 2,6-Et$_2$C$_6$H$_3$

Scheme 2.1 The Screened Chiral *N,N'*-dioxide Ligands.

Table 2.1 Exploring the reaction conditions

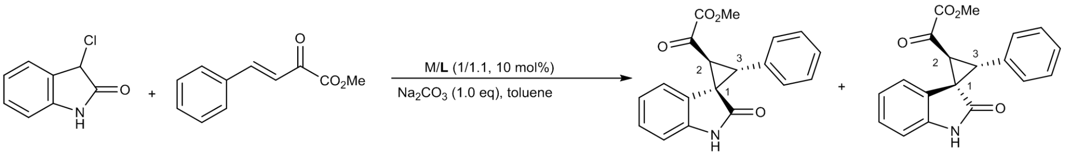

1a 2a *rel*-(1*R*,2*S*,3*R*)-3aa *rel*-(1*S*,2*S*,3*R*)-3aa'

Entry	Metal salt	**L**	Yield (**3aa/3aa'**) (%)^{b}	Ee (**3aa/3aa'**) (%)^{c}

1	Sc(OTf)3	L-PrPr2	92/7	76/0
2	Mg(OTf)2	L-PrPr2	14/28	0/88
3	Y(OTf)3	L-PrPr2	47/37	-28/68
4	Sc(NTf)3	L-PrPr2	59/16	50/-5
5	Ca(OTf)2	L-PrPr2	38/12	-12/0
6	Mg(NTf)2	L-PrPr2	60/32	17/86
7	Sc(OTf)3	L-RaPr2	92/--	78/--
8 [d]	Sc(OTf)3	L-RaPr2	73/--	91/--
9 [d,e]	Sc(OTf)3	L-RaPr2	99/--	92/--
10 [f]	Mg(OTf)2	L-PrPr2	--/77	--/95

[a] Unless otherwise noted, the reaction was proceeded with **1a** (0.1 mmol), **2a** (0.1 mmol), metal salt (10 mol%), **L** (11 mol%), and Na_2CO_3 (1.0 eq) in toluene (1.0 mL) at 30 ºC for 24 h. [b] Isolated yield. [c] Determined by chiral HPLC on a chiral stationary phase (Chiralcel IA and IE). [d] At 0 ºC. [e] The reaction proceeded for 72 h. [f] At 40 ºC for 72 h.

2) Selected examples of the optimization of the ligands

Table 2.2 Exploring the Efficiency of the ligand with Sc(OTf)3.

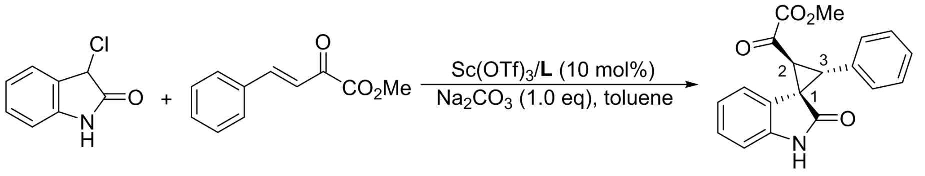

1a **2a** ***rel*-(1*R*,2*S*,3*R*)-3aa**

Entry	**L**	Yield of **3aa** (%)[b]	Ee of **3aa** (%)[c]
1	L-PrPr2	92	76
2	L-PrAd	52	42
3	L-PiPr2	94	70
4	L-RaPr2	92	78
5	L-RaPr3	99	68
6	L-RaEt2	89	77

[a] The reaction proceeded with **1a** (0.1 mmol), **2a** (0.1 mmol), prepared **L**/Sc(OTf)3 (1.1/1, 10 mol%) in THF, and Na_2CO_3 (1.0 eq) in toluene (1.0 mL) at 30 ºC for 24 h. [b] Isolated yield of *rel*-(1*R*,2*S*,3*R*)-**3aa**. [c] Determined by HPLC on a chiral stationary phase (chiralcel IE).

Table 2.3 Exploring the Efficiency of the ligand with Mg(OTf)2.

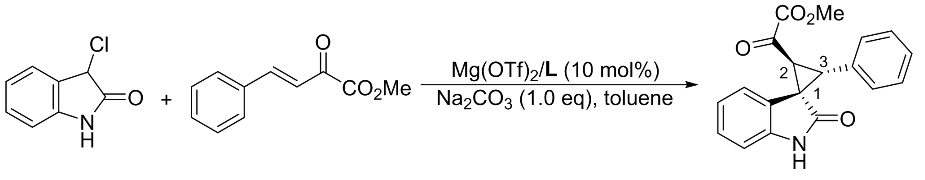

1a **2a** ***rel*-(1*S*,2*S*,3*R*)-3aa'**

Entry	L	Yield of **3aa’** (%)[b]	Ee of **3aa’** (%)[c]
1	L-PrPr2	28	88
2	L-PrPr3	67	70
3	L-PiPr2	29	15
4	L-RaPr2	21	81
5	L-PrEt2*p*Me	13	8

[a] The reaction proceeded with **1a** (0.1 mmol), **2a** (0.1 mmol), prepared **L**/Mg(OTf)2 (1.1/1, 10 mol%) in THF and Na_2CO_3 (1.0 eq) in toluene (1.0 mL) at 30 ºC for 24 h. [b] Isolated yield of *rel*-(1*S*,2*S*,3*R*)-**3aa’**. [c] Determined by HPLC on a chiral stationary phase (chiralcel IA).

3) Selected examples of optimization of the temperature

Table 2.4 The effect of temperature in **L-RaPr2**/Sc(OTf)3 complex catalyzed reaction.

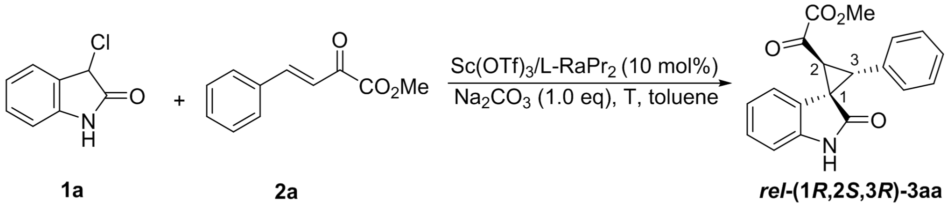

Entry	T (ºC)	Yield of **3aa** (%)[b]	ee of **3aa** (%)[c]
1	30	92	78
2	0	73	91
3	-10	59	45

[a] The reaction was proceeded with **1a** (0.1 mmol), **2a** (0.1 mmol), prepared **L-RaPr2**/Sc(OTf)3 (1.1/1, 10 mol%) in THF and Na_2CO_3 (1.0 eq) in toluene (1.0 mL) at the indicated temperature for 24 h. [b] Isolated yield of *rel*-(1*R*,2*S*,3*R*)-**3aa**. [c] Determined by HPLC on a chiral stationary phase (chiralcel IE).

Table 2.5 The effect of temperature in **L-PrPr2**/Mg(OTf)2 complex catalyzed reaction.

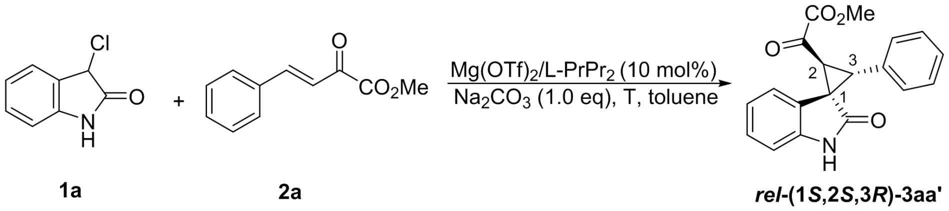

entry	T (ºC)	yield of **3aa**’ (%)[b]	ee of **3aa’** (%)[c]
1	30	65	88
2	40	77	95
3	50	67	89

[a] The reaction was proceeded with **1a** (0.1 mmol), **2a** (0.1 mmol), prepared **L-PrPr2**/Mg(OTf)2 (1.1/1, 10 mol%) in THF and Na_2CO_3 (1.0 eq) in toluene (1.0 mL) at the indicated temperature for 72 h. [b] Isolated yield of *rel*-(1*S*,2*S*,3*R*)-**3aa’**. [c] Determined by HPLC on a chiral stationary phase

(chiralcel IA).

3 General procedure

1）Synthesis of *rel*-(1*R*,2*S*,3*R*)-**3** and **4**:

THF was added to a $Sc(OTf)_3$ (10 mol%) and **L-RaPr$_2$** (11 mol%) charged dried reaction tube under nitrogen atmosphere. After stirring at 30 ºC for 1 h, the solvent was removed under vacuum. Subsequently, 3-Cl oxindole (0.1 mmol), β,γ-unsaturated-α-ketonester (0.1 mmol), and Na_2CO_3 (0.13 mmol) were added. Toluene (1.0 mL) was lastly added to the nitrogen charged reaction tube at 0 °C and kept stirring for 72 h. The mixture was directly purified by the flash column chromatography and used for the next step if necessary.

^{i}PrNH$_2$/DMF amidation: To the *rel*-(1*R*,2*S*,3*R*)-product **3** was added ^{i}PrNH$_2$ (0.10 mL) and DMF (0.30 mL) at room temperature. After 12 hours, the mixture was purified by the flash column chromatography (PE: EA = 1:1).

Table 3.1 The Substrates Scope under **L-RaPr$_2$**/$Sc(OTf)_3$ condition.

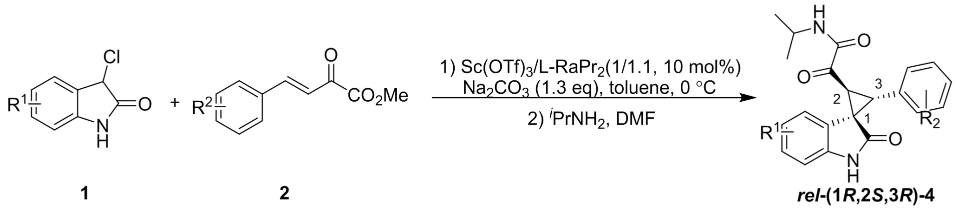

Entry	R[1]	R[2]	4	Yield(%)[b]	d.r.[c]	Ee[c]
1	H	H	**4aa**	68 (97)	> 99:1	91
2	4-Me	H	**4ba**	43 (67)	> 99:1	86
3	4-F	H	**4ca**	49 (76)	> 99:1	86
4	5-Me	H	**4da**	64 (96)	> 99:1	89
5	5-F	H	**4ea**	58 (96)	> 99:1	81
6	6-F	H	**4ga**	58 (91)	> 99:1	90
7	6-Cl	H	**4ha**	67 (98)	98:2	88
8	6-Br	H	**4ia**	66 (83)	> 99:1	84
9	H	2-MeO	**4ah**	59 (71)	94:6	91
10	H	3-MeO	**4ai**	62 (98)	> 99:1	95
11	H	4-MeO	**4aj**	63 (96)	> 99:1	72
12	H	2-Me	**4ak**	47 (76)	> 99:1	96
13	H	4-Me	**4al**	60 (93)	> 99:1	87
14	H	4-F	**4av**	46 (96)	> 99:1	90
15	H	3-Cl	**4am**	50 (76)	> 99:1	85
16	H	4-Cl	**4an**	44 (79)	> 99:1	93
17	H	4-Br	**4ap**	42 (83)	> 99:1	94
18	H	4-Ph	**4aq**	51 (96)	> 99:1	92
19	H	2-Nap	**4ar**	56 (91)	> 99:1	93

20	H	^{n}Bu	**4at**	68 (95)	76:24	99
21	H	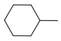	**4au**	60 (99)	72:28	99

[a] Unless otherwise noted, the reaction was performed with **1** (0.1 mmol), **2** (0.1 mmol), **L-RaPr$_2$**/Sc(OTf)$_3$/ (1.1/1, 10 mol%), and Na_2CO_3 (1.3 eq) in toluene (1.0 mL) at 0 °C for 72 h; then the reaction was isolated by the flash chromatography column and was added with ^{i}PrNH$_2$ (0.1 mL) in DMF (0.30 mL) for 12 h. [b] Isolated yield, The data in parentheses was the isolated yield after 1). [c] Determined by HPLC on a chiral stationary phase.

2）Synthesis of *rel*-(1*S*,2*S*,3*R*)-**3'**:

Mg(OTf)$_2$ (10 mol%), **L-PrPr$_2$** (11 mol%) were added to an over-dried reaction tube under nitrogen atmosphere. After THF (0.2 mL) was added, the mixture started stirring at 30 ºC for 1 h. Then the solvent was totally removed under vacuum. 3-Cl oxindole (0.1 mmol), β,γ-unsaturated-α-ketonester (0.1 mmol) and Na_2CO_3 (0.1 mmol) were added to the tube. Subsequently, toluene (1.0 mL) was added to the nitrogen charged reaction tube and stirred at 40 ºC for 72 h. This mixture was directly isolated under the flash chromatography (PE:Et_2O = 1:2).

4 Determination of absolute configuration of 3aa(')

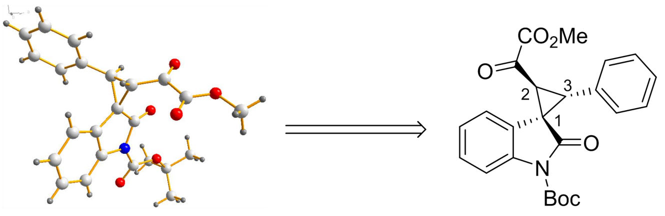

(1*R*,2*S*,3*R*)-**5aa** from **3aa**

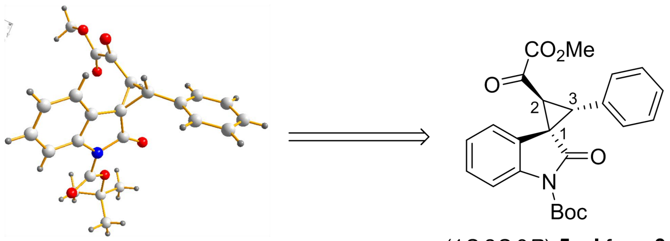

(1*S*,2*S*,3*R*)-**5aa'** from **3aa'**

3aa and **3aa'** were synthesized from **L-RaPr$_2$**/Sc(OTf)$_3$ and **L-PrPr$_2$**/Mg(OTf)$_2$ respectively according to the general procedure. And then they were derived by using Boc$_2$O/DMAP to give the corresponding **5aa** and **5aa'**.

Finally, they were recrystallized from CH_2Cl_2 and petrol ether. CCDC 1411858 (*rel*-(1*R*,2*S*,3*R*)-**5aa)** and CCDC 1412691 (*rel*-(1*S*,2*S*,3*R*)-**5aa')** were assigned to be (1*R*,2*S*,3*R*) and (1*S*,2*S*,3*R*) respectively. These data can be obtained free from the Cambridge Crystallographic Data Centre via www.ccdc.cam.ac.uk/data_requst/cif.

5 Studies of the mechanism

5.1 Nonlinear effect

a. **L-RaPr$_2$**/Sc(OTf)$_3$ condition

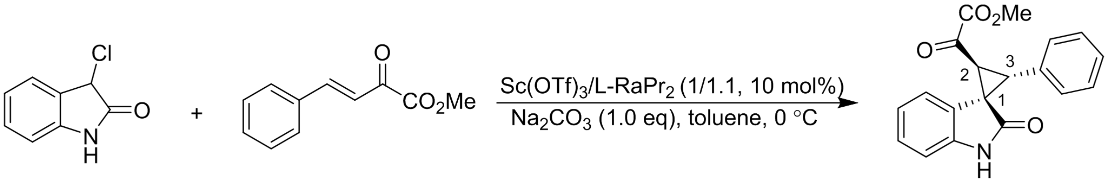

1a **2a** ***rel*-(1*R*,2*S*,3*R*)-3aa**

Entry	Ee of L-RaPr$_2$ (%)	Yield of **3aa** (%)	Ee of **3aa** (%)
1	0	60	3
2	20	68	27
3	40	74	48
4	60	71	64
5	80	78	80
6	100	81	90

[a] The reaction was performed with **1a** (0.1 mmol), **2a** (0.1 mmol), Sc(OTf)$_3$ (10 mol%), corresponding **L-RaPr$_2$** (11 mol%) and Na$_2$CO$_3$ (1.0 eq) in toluene (1.0 mL) at 0 ℃ for 48 h.

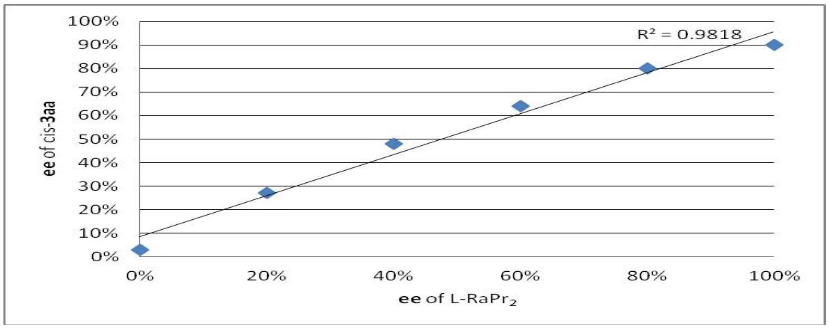

The nonlinear effect suggested that monomeric catalyst might be the main catalytical species.

b. **L-PrPr$_2$**/Mg(OTf)$_2$ condition

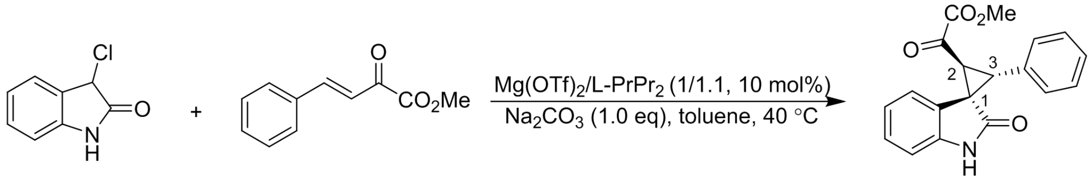

1a **2a** ***rel*-(1*S*,2*S*,3*R*)-3aa'**

Entry	Ee of L-PrPr$_2$ (%)	Yield of **3aa'** (%)	Ee of **3aa'** (%)
1	0	48	3
2	20	56	20
3	40	59	40
4	60	47	60
5	80	63	76
6	100	60	92

[a] The reaction performed with **1a** (0.1 mmol), **2a** (0.1 mmol), Mg(OTf)$_2$ (10 mol%), corresponding **L-PrPr$_2$** (11 mol%), and Na$_2$CO$_3$ (1.0 eq) at 40 ºC in toluene (1.0 mL) for 48 h.

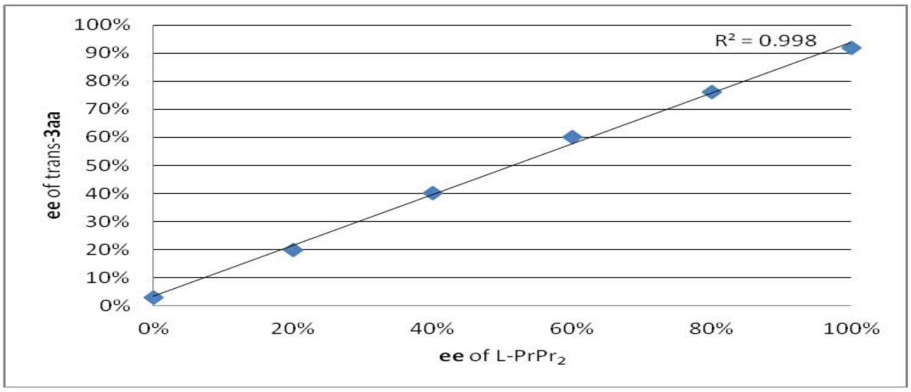

The nonlinear effect suggested that monomeric catalyst might be the main catalytical species.

5.2 The ORTEP diagramme of catalyst species

The crystalagraphic structure of CCDC 804337 [**L-RaPr$_2$**/Mg(OTf)$_2$], and CCDC 70400 [**L-PrPr$_2$**/Sc(OTf)$_3$], CCDC 882608 [**L-RaPr$_2$**/ Sc(OTf)$_3$/] have been confirmed in literature.[4] The coordination of *N,N′*-dioxides with either Sc(III) or Mg(II) is the same.

5.3 HRMS analysis of coordinative type

To check the coordinative type between substrates and Metal complexes, the complexes were stirred with the different substrate respectively. However, none of the possible coordinative fragment was found between β,γ-unsaturated-α-ketonester and metal complexes. On the contrary, the coordinative fragments from 3-Cl oxindole were detected.

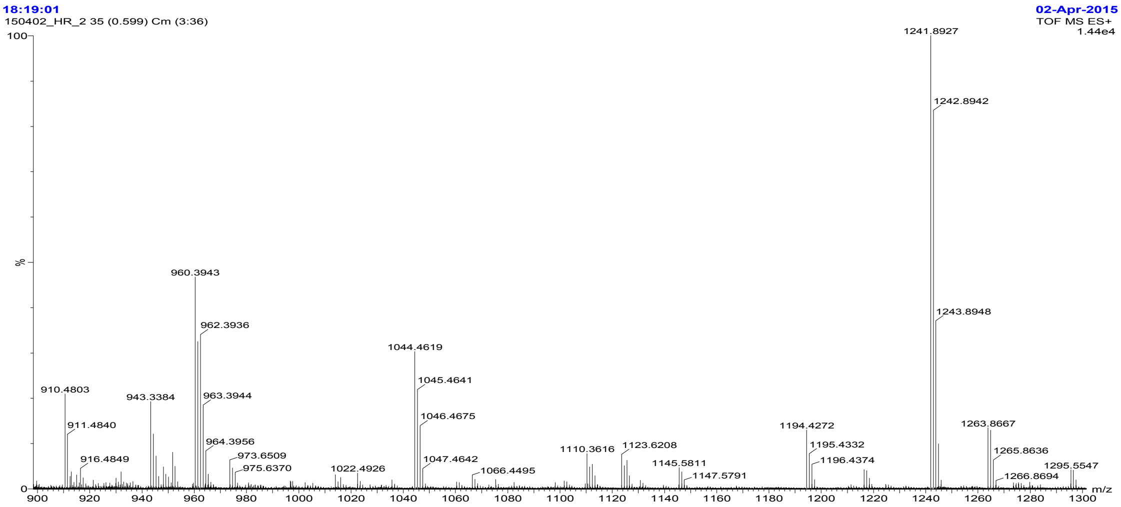

The mixture was prepared from the prepared **L-PrPr$_2$**/Mg(OTf)$_2$ (1.1/1, 0.01 mol), **1a** (0.2 mmol) in toluene (0.2 mL) for 2 h. The peak at 960.3943 was the [L-PrPr$_2$+Mg^{2+}+OTf$^-$+**1a**], (calc. 960.3810).

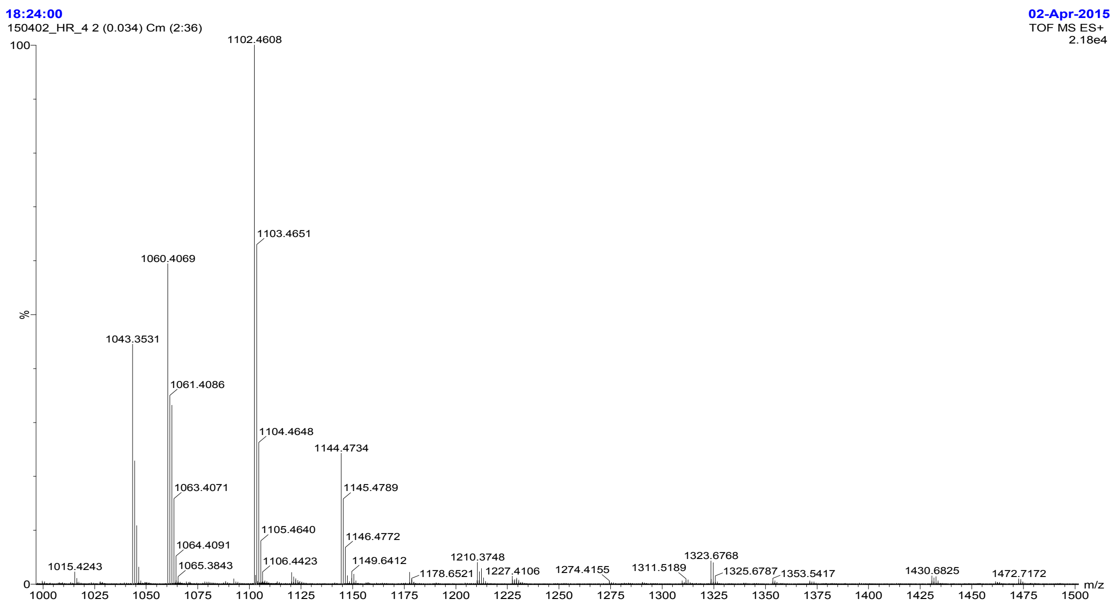

The mixture was prepared from the prepared **L-RaPr₂**/Sc(OTf)₃ (1.1/1, 0.01 mol), **1a** (0.2 mmol) in toluene (0.2 mL) for 2 h. The peak at 1210.3748 was the [L-RaPr$_2$+Sc^{3+}+2OTf$^-$+**1a**], (calc.1210.3665).

5.4 Comparative experiments

a) Synthesis of the Michale intermediate **6aa** and **6aa'**

Under the optimized condition without Na_2CO_3, only trace amount of Michael addition intermediates were detected. After optimizing these condition, the intermediate could be synthesized as follow. Sc(OTf)$_3$ (10 mol%) and **L-PrPr$_2$** (11 mol%) were added in a reaction tube under nitrogen, then, 0.2 mL THF was added with stirring for 30 min at 30 °C. After removing the solvent, **1a** (0.1 mmol), **2a** (0.1 mmol), 4 Å (30 mg), and 0.4 mL THF were added. Finnaly, the mixture was kept stirring at 0 °C for 48 h.

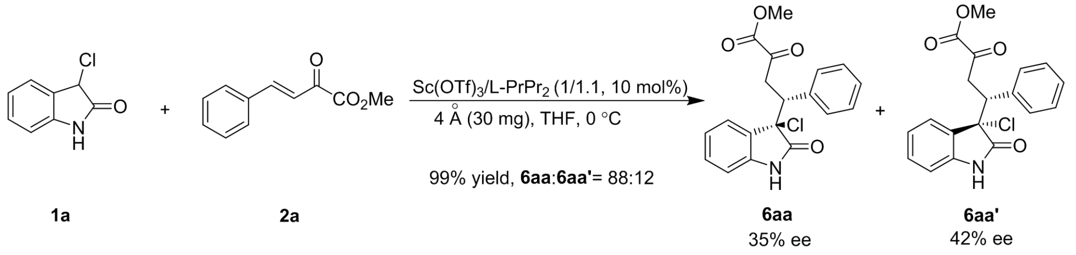

Two diastermers were partly isolated via flash column chromatography (PE : EA = 2:1). The **6aa** was further isolated via flash chromatography using a mixed eluent (PE : CH_2Cl_2 : acetone = 8 : 1 : 1). After recystallation, single diastereomer of **6aa** was isolated with the increasing ee value from 35% ee into 73% ee). Subsequently, the concentrated mother liquid was isolated again by the flash chromatography (PE : Et_2O = 1:2) to give **6aa'**. The racemic Michael intermediates were obtained by using racemic ligand.

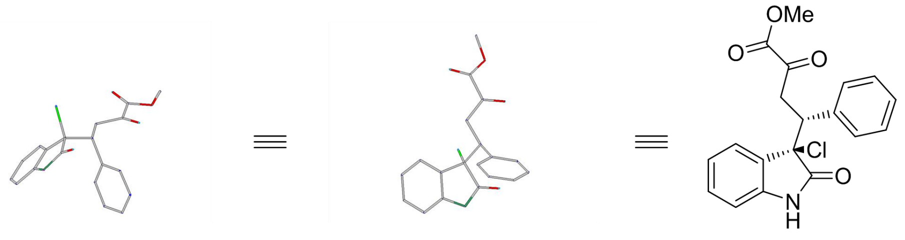

top view front view **6aa**

Additionaly, the relative configuration of **6aa** was also confirmed by X-ray crystallographic analysis. CCDC 1545249 (**6aa)** could be obtained free from the Cambridge Crystallographic Data Centre via www.ccdc.cam.ac.uk/data_requst/cif.

b) Reactivity of *N*-Me 3-Cl oxindole

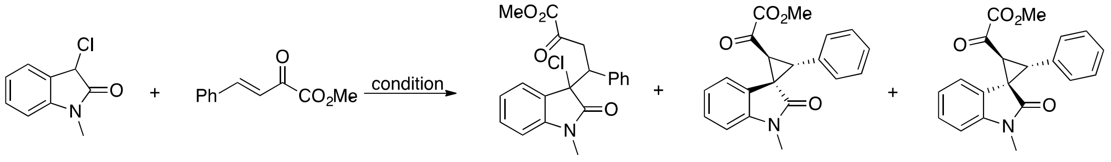

7 **8aa'** **8aa**

Entry	Condition[a]	7 [yield, ee]	8aa'[b] [yield, ee]	8aa[b] [yield, ee]
**1**	**L-RaPr**$_2$/Sc(OTf)$_3$	31%, 52%	—, —	25%, 30%
**2**	**L-PrPr**$_2$/Mg(OTf)$_2$	19%, -11%	40%, -4%	22%, -41%

[a]Unless otherwise noted, reactions were performed with **1** (0.1 mmol), **2** (0.1 mmol), chiral catalyst (10 mol%), Na_2CO_3 in toluene (1.0 mL) for 72 h. **L-PrPr**$_2$/Mg(OTf)$_2$ (1.1/1) and 1.0 eq of Na_2CO_3 were used at 40°C; **L-RaPr**$_2$/Sc(OTf)$_3$ (1.1/1, 10 mol%) and 1.0 eq of Na_2CO_3 were used at 0°C. [b] Isolated yield. ee was determined by HPLC on a chiral stationary.

To check the role of aza-*ortho*-xylylene in this reaction. *N*-Me 3-Cl oxindole was synthesized and subjected into the optimized condition. However, both **L-RaPr**$_2$/Sc(OTf)$_3$ and **L-PrPr**$_2$/Mg(OTf)$_2$ showed very low reactivities and poor ees with the same Michael addition diastereomer detected. The relative configuration was confirmed by *N*-methylation of **3aa'** with an opposite ee. The newly induced methyl group may affect the pKa of the substrate and change the coordinative type. It's also very hard to construct another diastereomer of **7**, even when the central Lewis acid was changed.

6 Characterization of the products

Methyl (1*S*,2*S*,3*R*)-2-oxo-2-(2'-oxo-2-phenylspiro[cyclopropane-1,3'-indoline]-3-yl)acetate:

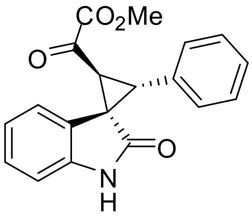

Prepared according to the general procedure. The compound of **3aa'** was purified by silica gel chromatography (petroleum ether: Et_2O = 1:2) to afford light yellow oil in 77% yield. HPLC (chiralcel IA, hexane/*i*-PrOH = 80:20, flow rate 1.0 mL/min, λ = 254 nm) t_r(major) = 12.24 min, t_r(minor) = 10.23 min, *ee* = 95%, dr > 99:1. $[\alpha]^{14.8}_D$ = 253.9 (c = 0.84, in CH_2Cl_2). ^1H NMR (400 MHz, $CDCl_3$) δ = 8.89 (s, 1H), 7.34 (d, *J* = 7.6 Hz, 1H), 7.32 – 7.26 (m, 5H), 7.24 – 7.18 (m, 1H), 7.02 (t, *J* = 7.6 Hz, 1H), 6.76 (d, *J* = 7.7 Hz, 1H), 4.35 (d, *J* = 8.3 Hz, 1H), 3.97 (d, *J* = 8.3 Hz, 1H), 3.86 (s, 3H). ^{13}C NMR (101 MHz, $CDCl_3$) δ = 187.3, 173.3, 160.3, 141.3, 132.5, 129.2, 128.2, 128.2, 127.8, 125.4, 122.8, 122.4, 110.2, 53.5, 44.0, 41.0, 40.7. HRMS (ESI-TOF) calcd for $C_{19}H_{15}NO_4$ (M+Na$^+$) = 344.0899, found 344.0898.

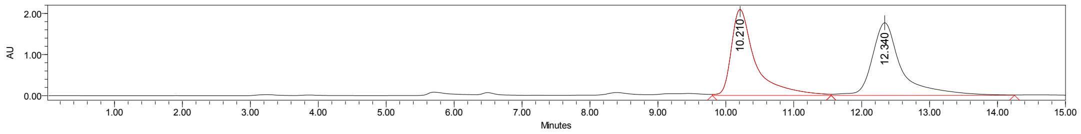

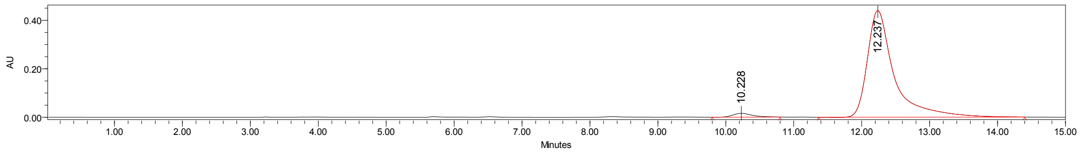

	Retention Time	Area	% Area
1	10.228	313815	2.54
2	12.237	12046603	97.46

Methyl (1*S*,2*S*,3*R*)-2-(2-(2-methoxyphenyl)-2'-oxospiro[cyclopropane-1,3'-indoline]-3-yl)-2-oxoacetate:

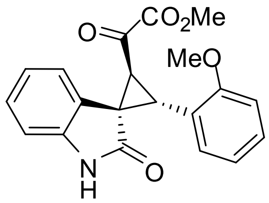

Prepared according to the general procedure. The compound of **3ah'** was purified by silica gel chromatography (petroleum ether: Et_2O = 1:2) to afford white solid in 69% yield. Mp. 188.0 – 190.0 ºC, HPLC (chiralcel IA, hexane/*i*-PrOH = 80:20, flow rate 1.0 mL/min, λ = 254 nm) t_r(major) = 10.61 min, t_r(minor) = 9.15 min, *ee* = 95%, dr > 99:1. $[\alpha]^{11.0}_D$ = 250.0 (c = 0.03, in CH_2Cl_2). ^1H NMR (400 MHz, $CDCl_3$) δ = 8.22 (s, 1H), 7.37 (d, *J* = 7.6 Hz, 1H), 7.31 – 7.26 (m, 2H), 7.26 – 7.19 (m, 1H), 7.03 (t, *J* = 7.6 Hz, 1H), 6.96 (t, *J* = 7.6 Hz, 1H), 6.87 – 6.75 (m, 2H), 4.24 – 4.15 (m, 1H), 3.86 (s, 3H), 3.80 (d, *J* = 8.3 Hz, 1H), 3.59 (s, 3H). ^{13}C NMR (101 MHz, $CDCl_3$) δ = 187.7, 173.3, 160.4, 158.2, 141.0, 130.1, 129.1, 127.8, 125.9, 123.0, 122.3, 121.3, 120.2, 110.2 109.6, 55.3, 53.4, 43.6, 41.0, 36.7. HRMS (ESI-TOF) calcd for $C_{20}H_{17}NO_5$ $(M+Na^+)$ = 374.1004, found 374.1006.

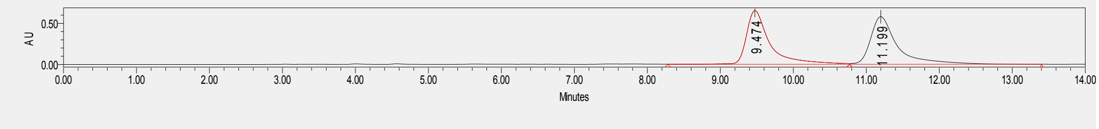

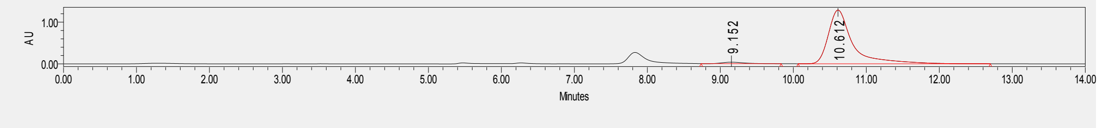

	Retention Time	Area	% Area
1	9.152	739851	2.43
2	10.612	29686935	97.57

Methyl (1*S*,2*S*,3*R*)-2-(2-(3-methoxyphenyl)-2'-oxospiro[cyclopropane-1,3'-indoline]-3-yl)-2-oxoacetate: Prepared according to the general procedure. The compound of **3ai'** was purified by silica gel chromatography (petroleum ether: Et_2O = 1:2) to afford white solid in 70% yield. Mp. 148.0 – 150.0 ºC, HPLC (chiralcel IA, hexane/*i*-PrOH = 80:20, flow rate 1.0 mL/min, λ = 254 nm)

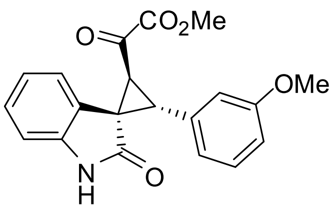

t_r(major) = 11.53 min, t_r(minor) = 9.08 min, *ee* = 92%, dr > 99:1. $[\alpha]^{14.9}_D$ = 281.65 (c = 0.28, in CH_2Cl_2). ^1H NMR (400 MHz, $CDCl_3$) δ = 8.10 (s, 1H), 7.35 (d, *J* = 7.6 Hz, 1H), 7.26 – 7.20 (m, 2H), 7.04 (t, *J* = 7.7 Hz, 1H), 6.93 – 6.78 (m, 4H), 4.35 (d, *J* = 8.3 Hz, 1H), 3.95 (d, *J* = 8.3 Hz, 1H), 3.87 (s, 3H), 3.77 (s, 3H). ^{13}C NMR (101 MHz, $CDCl_3$) δ = 187.3, 172.5, 160.3, 159.4, 141.0, 133.9, 129.2, 128.2, 125.4, 122.9, 122.5, 121.5, 114.9, 113.3, 109.8, 55.2, 53.5, 43.9, 41.0, 40.8. HRMS (ESI-TOF) calcd for $C_{20}H_{17}NO_5$ $(M+Na^+)$ = 374.1004, found 374.1006.

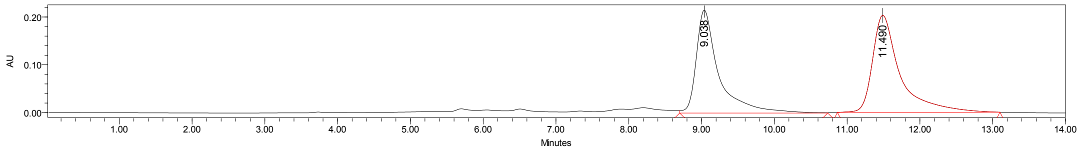

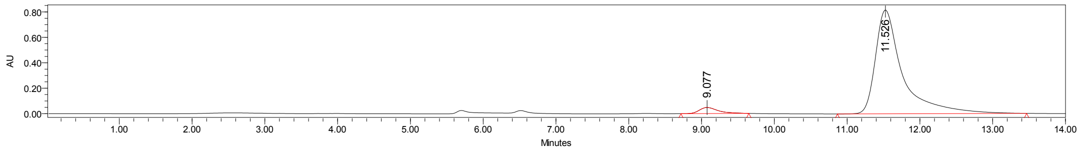

	Retention Time	Area	% Area
1	9.077	855723	3.90
2	11.526	21098002	96.10

Methyl (1*S*,2*S*,3*R*)-2-(2-(4-methoxyphenyl)-2'-oxospiro[cyclopropane-1,3'-indoline]-3-yl)-2-oxoacetate:

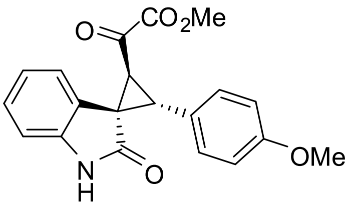

Prepared according to the general procedure. The compound of **3aj'** was purified by silica gel chromatography (petroleum ether: Et_2O = 1:2) to afford colorless oil in 59% yield. HPLC (chiralcel IA, hexane/*i*-PrOH = 80:20, flow rate 1.0 mL/min, λ = 254 nm) t_r(major) = 24.77 min, t_r(minor) = 14.62 min, *ee* = 94%, dr > 99:1. $[\alpha]^{12.9}_D$ = 196.9 (c = 0.10, in CH_2Cl_2). ^1H NMR (400 MHz,$CDCl_3$) δ = 7.75 (s, 1H), 7.36 (d, *J* = 7.7 Hz, 1H), 7.26 – 7.20 (m, 3H), 7.04 (t, *J* = 7.6 Hz, 1H), 6.89 – 6.82 (m, 3H), 4.33 (d, *J* = 8.3 Hz, 1H), 3.92 (d, *J* = 8.3 Hz, 1H), 3.88 (d, *J* = 4.7 Hz, 3H), 3.78 (s, 3H). ^{13}C NMR (101 MHz, $CDCl_3$) δ = 187.4, 172.4, 160.3, 159.1, 140.8, 130.2, 128.1, 125.6, 124.2, 123.0, 122.5, 113.6, 109.7, 55.2, 53.5, 43.9, 40.8, 40.8. HRMS (ESI-TOF) calcd for $C_{20}H_{17}NO_5$ $(M+Na^+)$ = 374.1004, found 374.1001.

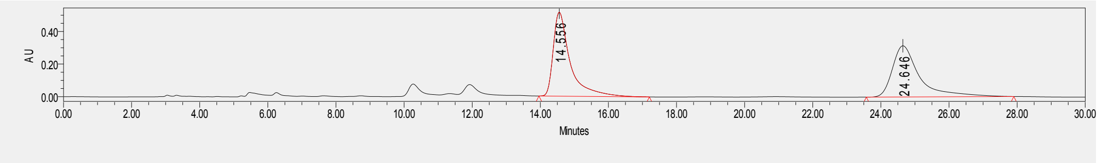

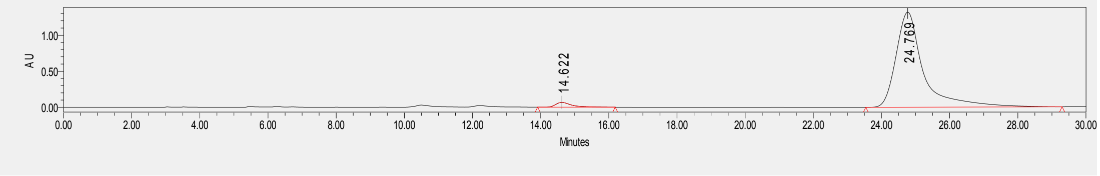

	Retention Time	Area	% Area
1	14.622	2192622	2.89
2	24.769	73779811	97.11

Methyl (1*S*,2*S*,3*R*)-2-oxo-2-(2'-oxo-2-o-tolylspiro[cyclopropane-1,3'-indoline]-3-yl)acetate:

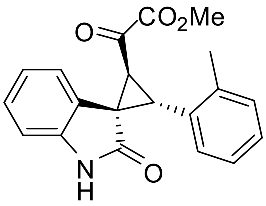

Prepared according to the general procedure. The compound of **3ak'** was purified by silica gel chromatography (petroleum ether: Et_2O = 1:2) to afford yellow solid in 50% yield. Mp. 150.0 – 152.0 ºC, HPLC (chiralcel IA, hexane/*i*-PrOH = 80:20, flow rate 1.0 mL/min, λ = 254 nm) t_r(major) = 10.13 min, t_r(minor) = 7.52 min, *ee* = 91%, dr > 99:1. $[\alpha]^{11.0}_D$ = 247.5 (c = 0.55, in CH_2Cl_2). ^1H NMR (400 MHz, $CDCl_3$) δ = 8.67 (s, 1H), 7.37 (d, *J* = 7.6 Hz, 1H), 7.34 – 7.28 (m, 1H), 7.25 – 7.16 (m, 3H), 7.13 – 7.08 (m, 1H), 7.07 – 7.01 (m, 1H), 6.77 (d, *J* = 7.8 Hz, 1H), 4.34 (d, *J* = 8.3 Hz, 1H), 3.87 (s, 3H), 3.81 (d, *J* = 8.3 Hz, 1H), 1.96 (s, 3H). ^{13}C NMR (101 MHz, $CDCl_3$) δ = 187.5, 173.1, 160.4, 141.1, 137.6, 131.4, 129.9, 129.1, 128.2, 127.9, 125.7, 125.2, 122.7, 122.5, 110.1, 53.5, 43.8, 40.7, 40.0, 19.4. HRMS (ESI-TOF) calcd for $C_{20}H_{17}NO_4$ (M+Na$^+$) = 358.1055, found 358.1055.

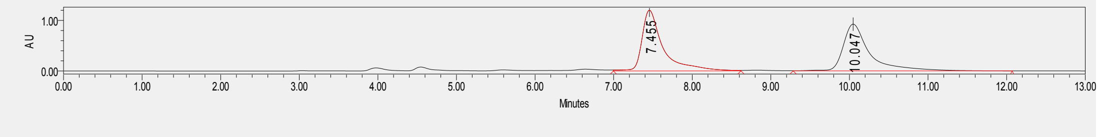

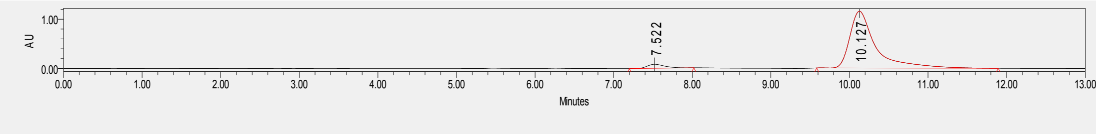

	Retention Time	Area	% Area
1	7.522	1227215	4.45
2	10.127	26323776	95.55

Methyl (1*S*,2*S*,3*R*)-2-oxo-2-(2'-oxo-2-p-tolylspiro[cyclopropane-1,3'-indoline]-3-yl)acetate:

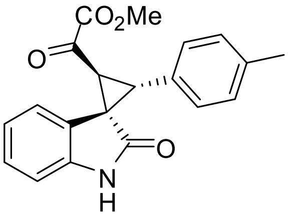

Prepared according to the general procedure. The compound of **3al'** was purified by silica gel chromatography (petroleum ether: Et_2O = 1:2) to afford yellow solid in 77% yield. Mp. 84.0 – 86.0 ºC, HPLC (chiralcel IA, hexane/*i*-PrOH = 80:20, flow rate 1.0 mL/min, λ = 254 nm) t_r(major) = 14.21 min, t_r(minor) = 9.80 min, *ee* = 93%, dr > 99:1. $[\alpha]^{14.0}_D$ = 249.5 (c = 0.42, in CH_2Cl_2). ^1H NMR (400 MHz, $CDCl_3$) δ = 8.43 (s, 1H), 7.35 (d, *J* = 7.6 Hz, 1H), 7.25 – 7.17 (m, 3H), 7.14 – 7.07 (m, 2H), 7.07 – 6.99 (m, 1H), 6.82 (d, *J* = 7.8 Hz, 1H), 4.34 (d, *J* = 8.3 Hz, 1H), 3.94 (d, *J* = 8.3 Hz, 1H), 3.86 (s, 3H), 2.31 (s, 3H). ^{13}C NMR (101 MHz, $CDCl_3$) δ = 187.4, 173.0, 160.4, 141.1, 137.5, 129.3, 129.0, 128.9, 128.1, 125.6, 122.9, 122.4, 109.9, 53.5, 43.9, 40.9, 40.7, 21.2. HRMS (ESI-TOF) calcd for $C_{20}H_{17}NO_4$ (M+Na$^+$) = 358.1055, found 358.1056.

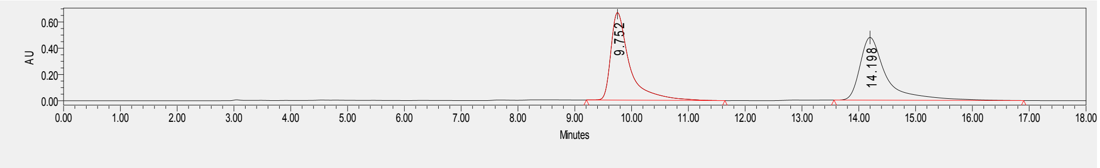

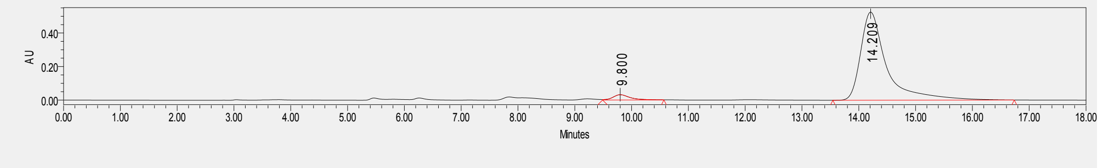

	Retention Time	Area	% Area
1	9.800	650120	3.75
2	14.209	16667191	96.25

Methyl (1*S*,2*S*,3*R*)-2-(-2-(3-chlorophenyl)-2'-oxospiro[cyclopropane-1,3'-indoline]-3-yl)-2-oxoacetate:

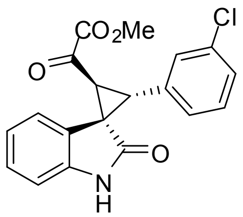

Prepared according to the general procedure. The compound of **3am'** was purified by silica gel chromatography (petroleum ether: Et_2O = 1:2) to afford yellow oil in 77% yield. HPLC (chiralcel IA, hexane/*i*-PrOH = 80:20, flow rate 1.0 mL/min, λ = 254 nm) t_r(major) = 11.53 min, t_r(minor) = 9.08 min, *ee* = 92%, dr > 99:1. $[\alpha]^{14.9}_D$ = 281.7 (c = 0.28, in CH_2Cl_2). ^1H NMR (400 MHz, $CDCl_3$) δ = 8.35 (s, 1H), 7.32 (d, *J* = 7.1 Hz, 2H), 7.26 – 7.16 (m, 4H), 7.04 (td, *J* = 7.7, 0.8 Hz, 1H), 6.86 (d, *J* = 7.7 Hz, 1H), 4.32 (d, *J* = 8.3 Hz, 1H), 3.91 (d, *J* = 8.3 Hz, 1H), 3.88 (s, 3H). ^{13}C NMR (101 MHz, $CDCl_3$) δ = 187.0, 172.5, 160.2, 141.1, 134.4, 134.1, 129.4, 129.3, 128.4, 128.0, 127.4, 125.0, 122.9, 122.6, 110.1, 53.6, 43.6, 40.4, 40.0. HRMS (ESI-TOF) calcd for $C_{19}H_{14}{}^{35}ClNO_4$ $(M+Na^+)$ = 378.0509, found 378.0511; HRMS (ESI-TOF) calcd for $C_{19}H_{14}{}^{37}ClNO_4$ $(M+Na^+)$ = 380.0480, found 380.0490.

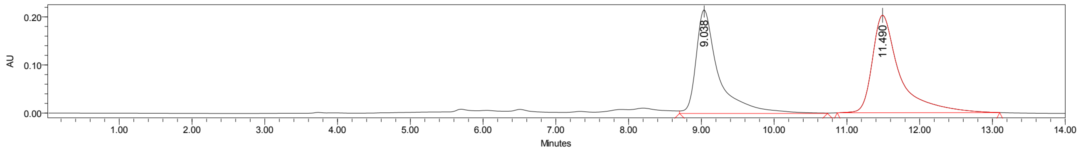

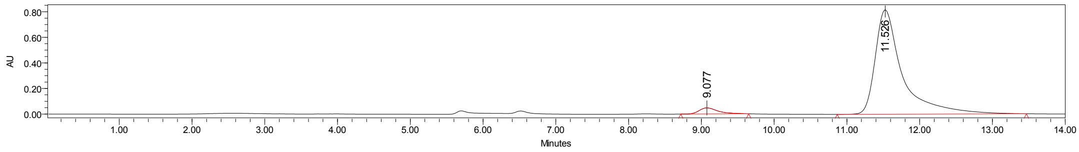

	Retention Time	Area	% Area
1	9.077	855723	3.90
2	11.526	21098002	96.10

Methyl

(1*S*,2*S*,3*R*)-2-(2-(4-chlorophenyl)-2'-oxospiro[cyclopropane-1,3'-indoline]-3-yl)-2-oxoacetate:

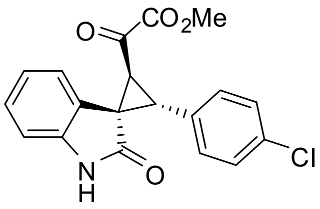

Prepared according to the general procedure. The compound of **3an'** was purified by silica gel chromatography (petroleum ether: Et_2O = 1:2) to afford yellow oil in 63% yield. HPLC (chiralcel IA, hexane/*i*-PrOH = 80:20, flow rate 1.0 mL/min, λ = 254 nm) t_r(major) = 16.19 min, t_r(minor) = 10.66 min, *ee* = 91%, dr > 99:1. $[\alpha]^{14.9}_D$ = 295.5 (c = 0.27, in CH_2Cl_2). ^1H NMR (400 MHz, $CDCl_3$) δ = 8.59 (s, 1H), 7.33 (d, *J* = 7.6 Hz, 1H), 7.31 – 7.22 (m, 5H), 7.03 (t, *J* = 7.6 Hz, 1H), 6.81 (d, *J* = 7.7 Hz, 1H), 4.32 (d, *J* = 8.3 Hz, 1H), 3.91 (d, *J* = 8.3 Hz, 1H), 3.87 (s, 3H). ^{13}C NMR (101 MHz, $CDCl_3$) δ = 187.0, 172.9, 160.3, 141.1, 133.7, 131.0, 130.5, 128.4, 128.4, 125.1, 122.9, 122.6, 110.1, 53.6, 43.7, 40.5, 40.0. HRMS (ESI-TOF) calcd for $C_{19}H_{14}{}^{35}ClNO_4$ (M+Na$^+$) = 378.0509, found 378.0513; HRMS (ESI-TOF) calcd for $C_{19}H_{14}{}^{37}ClNO_4$ (M+Na$^+$) = 380.0480, found 380.0491.

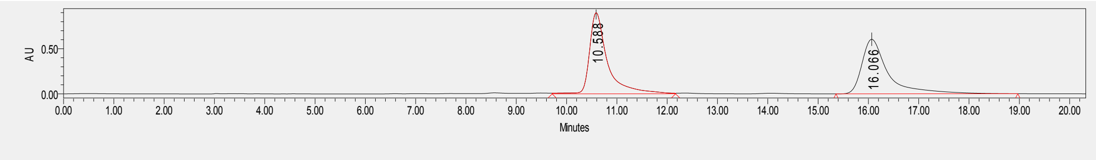

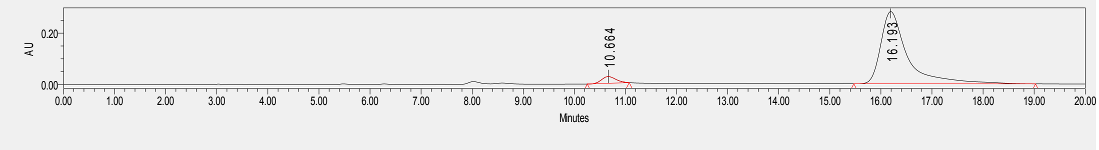

	Retention Time	Area	% Area
1	10.664	511787	4.77
2	16.193	10215126	95.23

Methyl (1*S*,2*S*,3*R*)-2-(2-(2-bromophenyl)-2'-oxospiro[cyclopropane-1,3'-indoline]-3-yl)-2-oxoacetate:

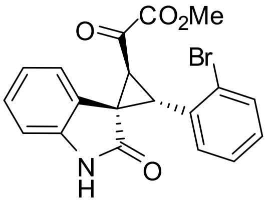

Prepared according to the general procedure. The compound of **3ao'** was purified by silica gel chromatography (petroleum ether: Et_2O = 1:2) to afford white solid in 62% yield. Mp. 176.0 – 178.0 ºC, HPLC (chiralcel IA, hexane/*i*-PrOH = 80:20, flow rate 1.0 mL/min, λ = 254 nm) t_r(major) = 18.59 min, t_r(minor) = 9.69 min, *ee* = 94%, dr > 99:1. $[\alpha]^{11.0}_D$ = 49.5 (c = 0.20, in CH_2Cl_2). ^1H NMR (400 MHz, $CDCl_3$) δ = 8.41 (s, 1H), 7.50 (d, *J* = 7.9 Hz, 1H), 7.35 (m, 3H), 7.26 – 7.21 (m, 1H), 7.20 - 7.14 (m, 1H), 7.08 – 6.98 (m, 1H), 6.82 – 6.76 (m, 1H), 4.28 (d, *J* = 8.2 Hz, 1H), 3.89 – 3.83 (m, 3H). ^{13}C NMR (101 MHz, $CDCl_3$) δ = 187.1, 172.9, 160.3, 141.3, 133.1, 132.5, 131.0, 129.4, 128.2, 127.2, 125.6, 125.1, 123.0, 122.5, 109.9, 53.5, 44.0, 41.2, 40.9. HRMS (ESI-TOF) calcd for $C_{19}H_{14}{}^{79}BrNO_4$ (M+Na$^+$) = 422.0004, found 422.0007; calcd for $C_{19}H_{14}{}^{81}BrNO_4$ (M+Na$^+$) = 423.9983, found 423.9993.

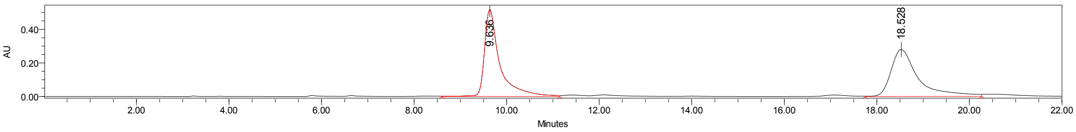

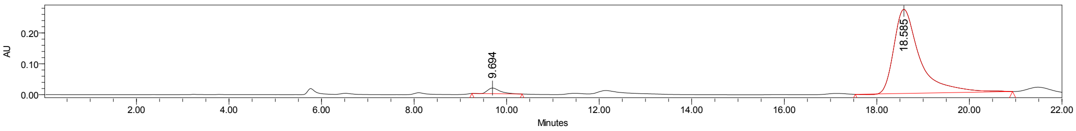

	Retention Time	Area	% Area
1	9.694	343895	3.13
2	18.585	10643433	96.87

Methyl (1*S*,2*S*,3*R*)-2-(2-(4-bromophenyl)-2'-oxospiro[cyclopropane-1,3'-indoline]-3-yl)-2-oxoacetate: Prepared according to the general procedure. The compound of **3ap'** was purified by silica gel chromatography (petroleum ether: Et_2O = 1:2) to afford light yellow solid in 81% yield. Mp. 66.0 – 68.0 ºC, HPLC (chiralcel IA, hexane/*i*-PrOH = 80:20, flow rate 1.0 mL/min, λ = 254 nm) t_r(major) = 17.11 min, t_r(minor) = 11.22 min, *ee* = 94%, dr > 99:1. $[\alpha]^{10.9}_D$ = 296.3 (c = 0.40, in CH_2Cl_2). ^1H NMR (400 MHz, $CDCl_3$) δ = 8.55 (s, 1H), 7.43 (d, *J* = 8.4 Hz, 1H), 7.33 (d, *J* = 7.6 Hz, 1H), 7.30 – 7.25 (m, 2H), 7.23 – 7.17 (m, 2H), 7.04 (t, *J* = 7.6 Hz, 1H), 6.81 (d, *J* = 7.8 Hz, 1H), 4.32 (d, *J* = 8.3 Hz, 1H), 3.89 (d, *J* = 8.3 Hz, 1H), 3.87 (s, 3H). ^{13}C NMR (101 MHz, $CDCl_3$) δ = 187.0 172.9, 160.3, 141.1, 131.5, 131.4, 130.9, 128.4, 125.1, 122.9, 122.6, 121.9, 110.1, 53.6, 43.7, 40.4, 40.0. HRMS (ESI-TOF) calcd for $C_{19}H_{14}{}^{79}BrNO_4$ (M+Na$^+$) = 422.0004, found 422.0009; calcd for $C_{19}H_{14}{}^{81}BrNO_4$ (M+Na$^+$) = 423.9983, found 423.9989.

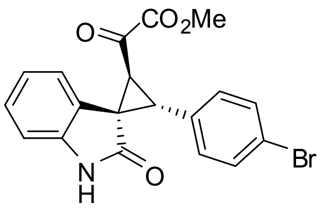

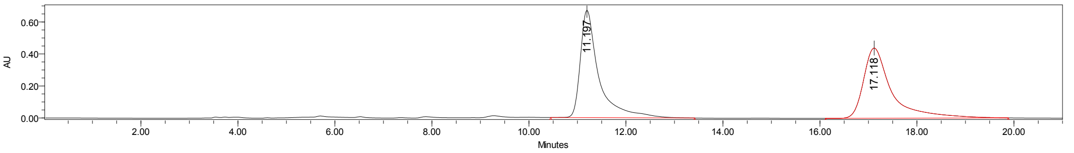

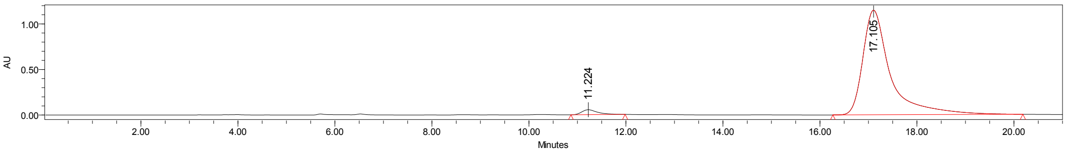

	Retention Time	Area	% Area
1	11.224	1233355	2.66
2	17.105	45173061	97.34

Methyl (1*S*,2*S*,3*R*)-2-(2-(biphenyl-4-yl)-2'-oxospiro[cyclopropane-1,3'-indoline]-3-yl)-2-oxoacetate: Prepared according to the general procedure. The compound of **3aq'** was purified by silica gel chromatography (petroleum ether: Et_2O = 1:2) to afford white solid in 74% yield. Mp. 88.0 – 90.0

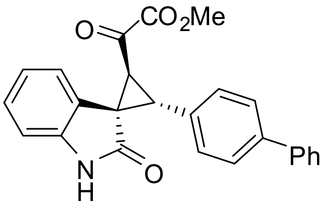

ºC, HPLC (chiralcel IA, hexane/*i*-PrOH = 80:20, flow rate 1.0 mL/min, λ = 254 nm) t_r(major) = 27.46 min, t_r(minor) = 14.54 min, *ee* = 98%, dr > 99:1. $[\alpha]^{15.1}_{D}$ = 307.8 (c = 0.42, in CH_2Cl_2). ^1H NMR (400 MHz, $CDCl_3$) δ = 8.72 (s, 1H), 7.57 – 7.50 (m, 4H), 7.48 – 7.40 (m, 2H), 7.39 – 7.30 (m, 4H), 7.11 (t, *J* = 7.4 Hz, 1H), 7.00 (t, *J* = 7.5 Hz, 1H), 6.74 (d, *J* = 7.7 Hz, 1H), 4.40 (d, *J* = 8.3 Hz, 1H), 3.98 (d, *J* = 8.3 Hz, 1H), 3.86 (s, 3H). ^{13}C NMR (101 MHz, $CDCl_3$) δ = 187.3, 173.2, 160.3, 141.2, 140.6, 131.5, 129.6, 128.8, 128.2, 127.4, 127.1, 126.9, 125.4, 122.9, 122.4, 110.1, 53.5, 44.0, 40.8, 40.6. HRMS (ESI-TOF) calcd for $C_{25}H_{19}NO_4$ $(M+Na^+)$ = 420.1212, found 420.1210.

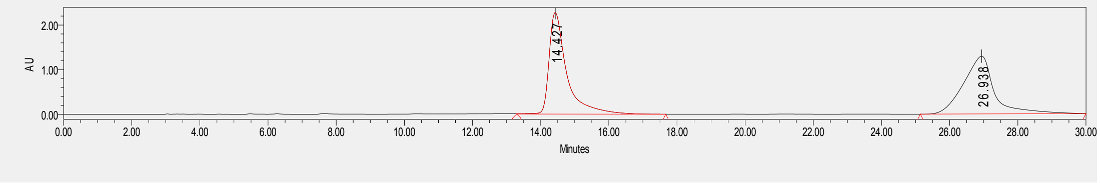

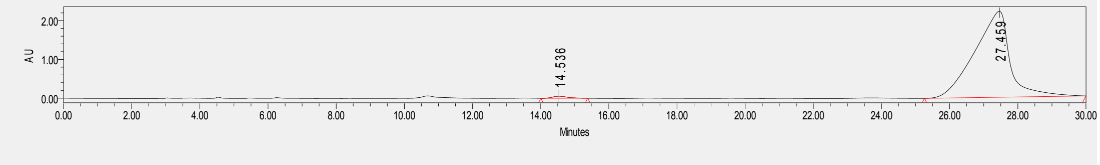

	Retention Time	Area	% Area
1	14.536	1564552	0.97
2	27.459	160521023	99.03

Methyl (1*S*,2*S*,3*R*)-2-(2-(naphthalen-2-yl)-2'-oxospiro[cyclopropane-1,3'-indoline]-3-yl)-2-oxoacetate:

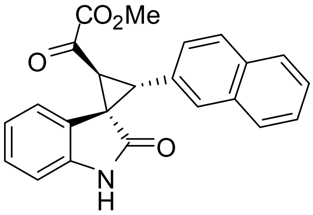

Prepared according to the general procedure. The compound of **3ar'** was purified by silica gel chromatography (petroleum ether: Et_2O = 1:2) to afford light yellow oil in 71% yield. HPLC (chiralcel IA, hexane/*i*-PrOH = 80:20, flow rate 1.0 mL/min, λ = 254 nm) t_r(major) = 20.26 min, t_r(minor) = 14.27 min, *ee* = 93%, dr > 99:1. $[\alpha]^{13.7}_{D}$ = 485.4 (c = 0.21, in CH_2Cl_2). ^1H NMR (400 MHz, $CDCl_3$) δ = 8.51 (s, 1H), 7.85 – 7.72 (m, 4H), 7.50 – 7.42 (m, 2H), 7.40 – 7.33 (m, 2H), 7.14 (t, *J* = 7.8 Hz, 1H), 7.02 (t, *J* = 7.8 Hz, 1H), 6.61 (d, *J* = 7.7 Hz, 1H), 4.48 (d, *J* = 8.3 Hz, 1H), 4.11 (d, *J* = 8.3 Hz, 1H), 3.88 (s, 3H). ^{13}C NMR (101 MHz, $CDCl_3$) δ = 187.3, 173.0, 160.4, 141.1, 133.0, 132.9, 129.9, 128.2, 128.2, 127.9, 127.9, 127.7, 126.9, 126.3, 126.1, 125.4, 122.9, 122.4, 110.0, 53.6, 44.0, 41.1, 40.7. HRMS (ESI-TOF) calcd for $C_{23}H_{17}NO_4$ $(M+Na^+)$ = 394.1055, found 394.1060.

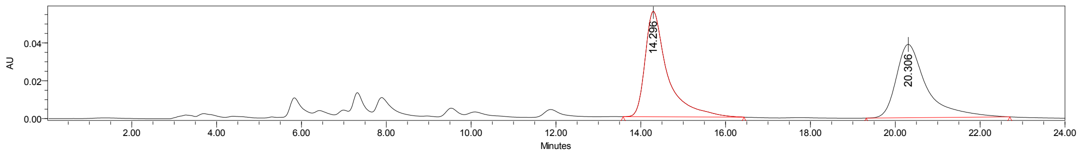

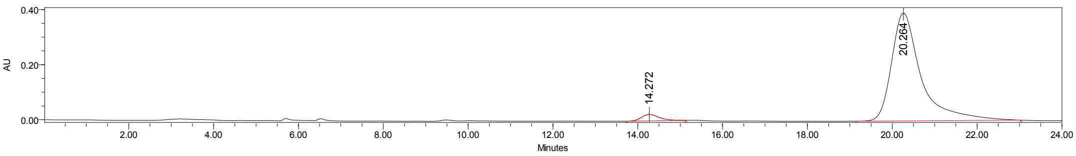

	Retention Time	Area	% Area
1	14.272	670529	3.42
2	20.264	18913427	96.58

Methyl (1*S*,2*S*,3*R*)-2-oxo-2-(2'-oxo-2-(thiophen-2-yl)spiro[cyclopropane-1,3'-indoline]-3-yl)acetate:

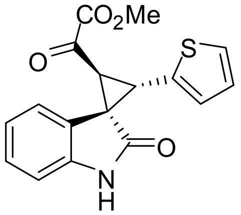

Prepared according to the general procedure. The compound of **3as'** was purified by silica gel chromatography (petroleum ether: Et_2O = 1:2) to afford yellow oil in 52% yield. HPLC (chiralcel IA, hexane/*i*-PrOH = 80:20, flow rate 1.0 mL/min, λ = 254 nm) t_r(major) = 15.08 min, t_r(minor) = 10.51 min, *ee* = 95%, dr > 99:1. $[\alpha]^{13.7}_D$ = 150.6 (c = 0.53, in CH_2Cl_2). ^1H NMR (400 MHz, $CDCl_3$) δ = 8.72 (s, 1H), 7.30 (d, *J* = 7.6 Hz, 1H), 7.26 – 7.23 (m, 1H), 7.21 (d, *J* = 5.6 Hz, 1H), 7.08 (d, *J* = 3.4 Hz, 1H), 7.03 (t, *J* = 7.6 Hz, 1H), 6.98 – 6.93 (m, 1H), 6.87 (d, *J* = 7.8 Hz, 1H), 4.35 (d, *J* = 8.1 Hz, 1H), 3.99 (d, *J* = 8.1 Hz, 1H), 3.86 (s, 3H). ^{13}C NMR (101 MHz, $CDCl_3$) δ = 186.8, 172.5, 160.1, 141.2, 135.0, 128.3, 127.8, 126.8, 125.5, 124.9, 122.8, 122.5, 110.2, 53.6, 44.0, 41.9, 35.6. HRMS (ESI-TOF) calcd for $C_{17}H_{13}NO_4S$ $(M+Na^+)$ = 350.0463, found 350.0464.

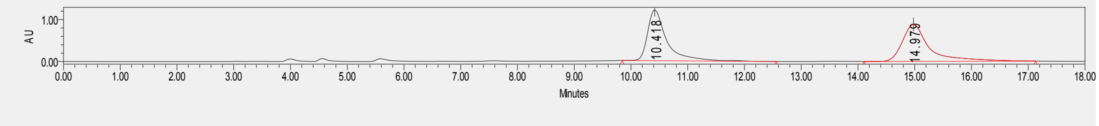

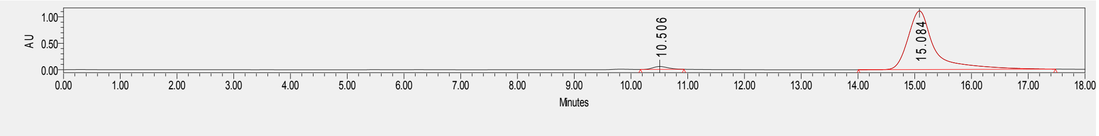

	Retention Time	Area	% Area
1	10.506	1023037	2.75
2	15.084	36154409	97.25

Methyl (1*S*,2*S*,3*R*)-2-(2-butyl-2'-oxospiro[cyclopropane-1,3'-indoline]-3-yl)-2-oxoacetate:

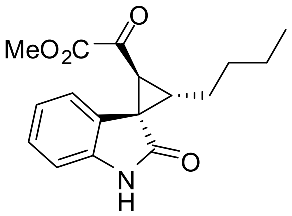

Prepared according to the general procedure. The compound of **3at'** was purified by silica gel chromatography (petroleum ether: Et_2O = 1:2) to afford yellow oil in 71% yield. HPLC (chiralcel IA, hexane/*i*-PrOH = 80:20, flow rate 1.0 mL/min, λ = 254 nm) t_r(major) = 11.32 min, t_r(minor) = 10.11 min, *ee* = 93%, dr > 99:1. $[\alpha]^{12.3}_D$ = 174.5 (c = 0.43, in CH_2Cl_2). ^1H NMR (400 MHz, $CDCl_3$) δ = 9.07 (s, 1H), 7.27 – 7.22 (m, 1H), 7.06 – 7.02 (m, 2H), 7.00 (d, *J* = 7.8 Hz, 1H), 3.77 (s, 3H), 3.00 (d, *J* = 8.4 Hz, 1H), 2.71 (q, *J* = 7.5 Hz, 1H), 1.85 (dd, *J* = 13.9, 7.6 Hz, 1H), 1.70 (tt, *J* = 13.5, 6.8 Hz,

1H), 1.43 – 1.25 (m, 4H), 0.85 (t, J = 6.9 Hz, 3H). ^{13}C NMR (101 MHz, $CDCl_3$) δ = 187.0, 175.8, 160.8, 141.5, 127.9, 126.3, 122.1, 121.3, 110.6, 53.1, 41.9, 40.1, 35.4, 30.8, 26.5, 22.1, 13.9. HRMS (ESI-TOF) calcd for $C_{17}H_{20}NO_4$ (M+H$^+$) = 302.1392, found 302.1385.

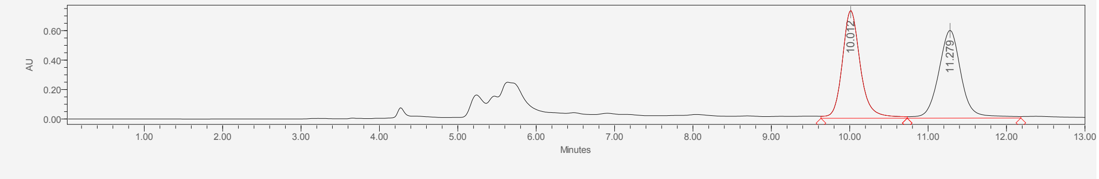

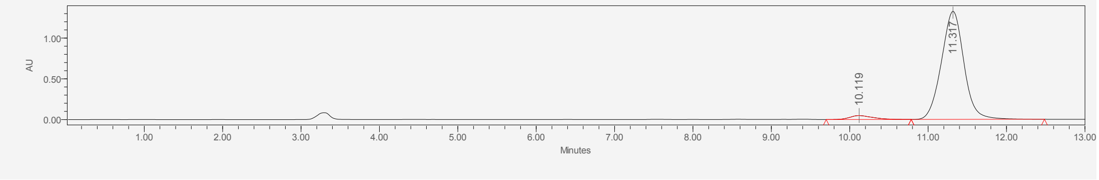

	Retention Time	Area	% Area
1	10.119	974803	3.55
2	11.317	26446603	96.45

Methyl (1S,2S,3R)-2-(4'-fluoro-2'-oxo-2-phenylspiro[cyclopropane-1,3'-indoline]-3-yl)-2-oxoacetate:

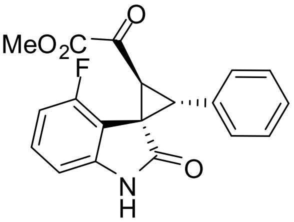

Prepared according to the general procedure. The compound of **3ca'** was purified by silica gel chromatography (petroleum ether: Et_2O= 1:2) to afford white oil in 84% yield. HPLC (chiralcel IA, hexane/i-PrOH = 80:20, flow rate 1.0 mL/min, λ = 254 nm) t_r(major) = 19.60 min, t_r(minor) = 10.21 min, ee = 96%, dr > 99:1. $[\alpha]^{14.6}_D$ = 213.2 (c = 0.34, in CH_2Cl_2). ^{1}H NMR (400 MHz, $CDCl_3$) δ = 8.66 (s, 1H), 7.39 – 7.27 (m, 5H), 7.22 – 7.14 (m, 1H), 6.76 – 6.67 (m, 1H), 6.61 (d, J = 7.8 Hz, 1H), 4.27 (d, J = 8.9 Hz, 1H), 3.96 (d, J = 8.9 Hz, 1H), 3.86 (s, 3H). ^{13}C NMR (101 MHz, $CDCl_3$) δ = 186.8, 172.9, 160.5, 157.4 (d, J = 247.4 Hz), 143.2 (d, J = 8.2 Hz), 132.5, 129.7 (d, J = 8.8 Hz), 129.2, 128.2, 127.8, 111.6 (d, J = 17.5 Hz), 110.1 (d, J = 21.3 Hz), 106.5 (d, J = 3.2 Hz), 53.4, 40.5, 37.0, 37.0. HRMS (ESI-TOF) calcd for $C_{19}H_{14}FNO_4$ (M+Na$^+$) = 362.0805, found 362.0807.

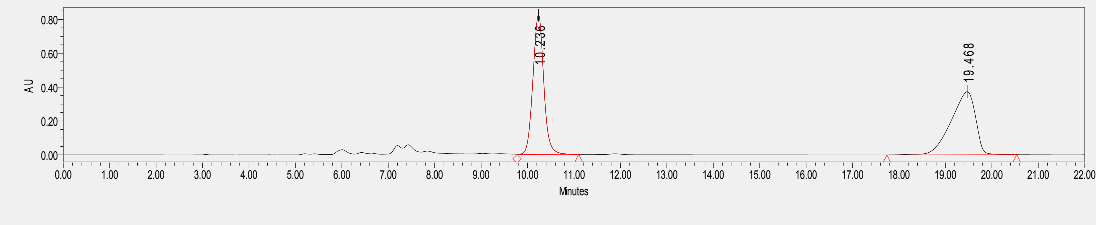

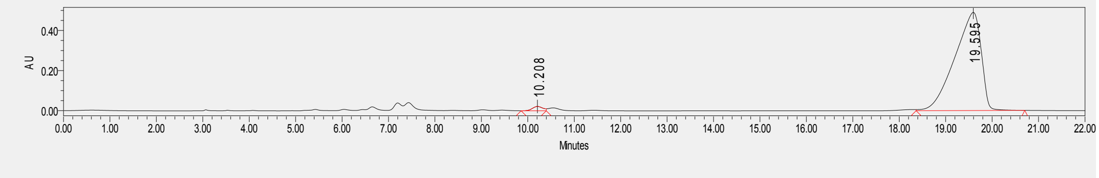

	Retention Time	Area	% Area
1	10.208	338532	1.71

2	19.595	19409542	98.29

Methyl (1*S*,2*S*,3*R*)-2-(5'-methyl-2'-oxo-2-phenylspiro[cyclopropane-1,3'-indoline]-3-yl)-2-oxoacetate:

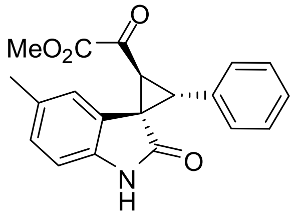

Prepared according to the general procedure. The compound of **3da'** was purified by silica gel chromatography (petroleum ether: Et_2O = 1:2) to afford light yellow oil in 72% yield. HPLC (chiralcel ID, hexane/*i*-PrOH = 90:10, flow rate 1.0 mL/min, λ = 254 nm) t_r(major) = 20.49 min, t_r(minor) = 24.2 9min, *ee* = 96%, dr > 99:1. $[\alpha]^{14.0}_D$ = 210.6 (c = 0.55, in CH_2Cl_2). ^1H NMR (400 MHz, $CDCl_3$) δ = 8.57 (s, 1H), 7.35 – 7.26 (m, 5H), 7.17 (s, 1H), 7.02 (d, *J* = 7.8 Hz, 1H), 6.68 (d, *J* = 7.9 Hz, 1H), 4.34 (d, *J* = 8.3 Hz, 1H), 3.94 (d, *J* = 8.3 Hz, 1H), 3.87 (s, 3H), 2.34 (s, 3H). ^{13}C NMR (101 MHz, $CDCl_3$) δ = 187.4, 173.1, 160.3, 138.8, 132.6, 132.0, 129.1, 128.5, 128.2, 127.8, 125.5, 123.6, 109.8, 53.5, 44.1, 41.0, 40.6, 21.3. HRMS (ESI-TOF) calcd for $C_{20}H_{17}NO_4$ $(M+Na^+)$ = 358.1055, found 358.1055.

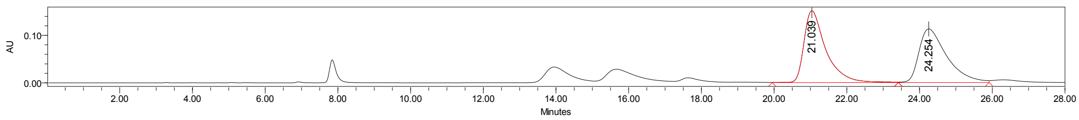

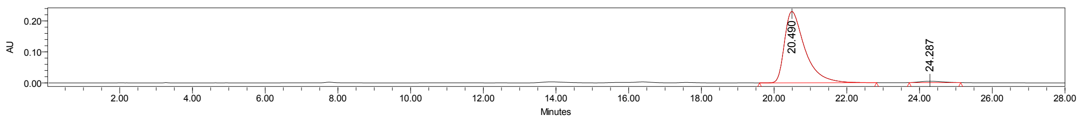

	Retention Time	Area	% Area
1	20.490	9003766	97.94
2	24.287	189526	2.06

Methyl (1*S*,2*S*,3*R*)-2-(5'-fluoro-2'-oxo-2-phenylspiro[cyclopropane-1,3'-indoline]-3-yl)-2-oxoacetate:

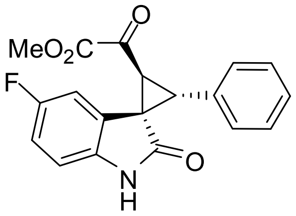

Prepared according to the general procedure. The compound of **3ea'** was purified by silica gel chromatography (petroleum ether: Et_2O = 1:2) to afford yellow oil in 67% yield. HPLC (chiralcel IA, hexane/*i*-PrOH = 80:20, flow rate 1.0 mL/min, λ = 254 nm) t_r(major) = 9.87 min, t_r(minor) = 7.92 min, *ee* = 92%, dr > 99:1. $[\alpha]^{13.4}_D$ = 235.2 (c = 0.78, in CH_2Cl_2). ^1H NMR (400 MHz, $CDCl_3$) δ = 9.09 (s, 1H), 7.40 – 7.26 (m, 5H), 7.18 – 7.12 (m, 1H), 6.95 – 6.88 (m, 1H), 6.66 – 6.60 (m, 1H), 4.36 (d, *J* = 8.3 Hz, 1H), 3.92 (d, *J* = 8.4 Hz, 1H), 3.88 (s, 3H). ^{13}C NMR (101 MHz, $CDCl_3$) δ = 187.3, 173.3, 158.8 (d, *J* = 238.5 Hz), 157.6, 137.2 (d, *J* = 2.0 Hz), 132.2, 129.2, 128.3, 127.9, 126.9 (d, *J* = 9.3 Hz), 114.6 (d, *J* = 23.5 Hz), 111.1 (d, *J* = 26.5 Hz), 110.7 (d, *J* = 6.5 Hz), 53.6, 44.12 (d, *J* = 2.0 Hz), 41.6, 40.5. HRMS (ESI-TOF) calcd for $C_{19}H_{14}FNO_4$ $(M+H^+)$ = 340.0985, found 340.0982.

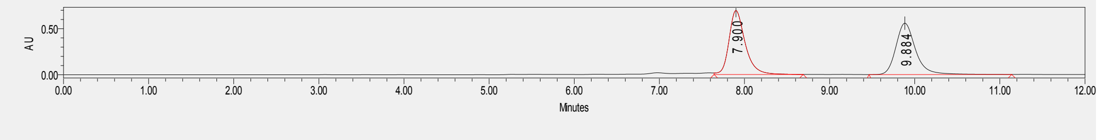

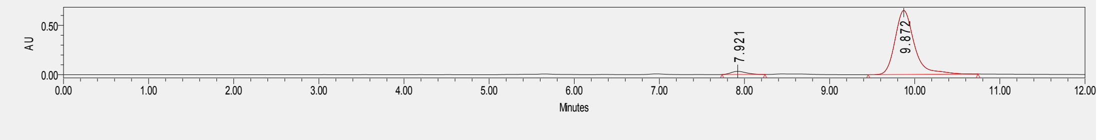

	Retention Time	Area	% Area
1	7.921	396567	3.74
2	9.872	10213204	96.26

Methyl (1*S*,2*S*,3*R*)-2-(5'-chloro-2'-oxo-2-phenylspiro[cyclopropane-1,3'-indoline]-3-yl)-2-oxoacetate:

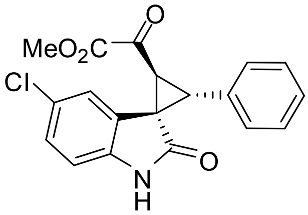

Prepared according to the general procedure. The compound of **3fa'** was purified by silica gel chromatography (petroleum ether: Et_2O = 1:2) to afford light yellow solid in 61% yield. Mp. 172.0 – 174.0 ºC, HPLC (chiralcel IA, hexane/*i*-PrOH = 80:20, flow rate 1.0 mL/min, λ = 254 nm) t_r(major) = 9.03 min, t_r(minor) = 7.94 min, *ee* = 88%, dr > 99:1. ^{1}H NMR (400 MHz, $CDCl_3$) δ = 8.74 (s, 1H), 7.39 (d, *J* = 1.5 Hz, 1H), 7.35 – 7.27 (m, 5H), 7.24 – 7.16 (m, 1H), 6.70 (d, *J* = 8.3 Hz, 1H), 4.37 (d, *J* = 8.4 Hz, 1H), 3.93 (d, *J* = 8.4 Hz, 1H), 3.89 (s, 3H). ^{13}C NMR (101 MHz, $CDCl_3$) δ = 187.2, 172.7, 160.3, 139.7, 132.0, 129.1, 128.3, 128.2, 128.0, 127.9, 127.1, 123.4, 111.9, 53.6, 43.8, 41.7, 40.5. HRMS (ESI-TOF) calcd for $C_{19}H_{14}{}^{35}ClNO_4$ $(M+H^+)$ = 356.0690, found 356.0687; HRMS (ESI-TOF) calcd for $C_{19}H_{14}{}^{37}ClNO_4$ $(M+H^+)$ = 358.0660, found 358.0651.

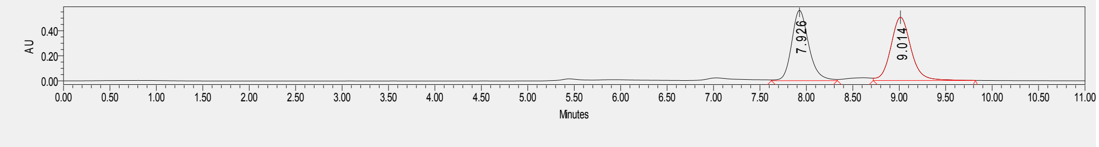

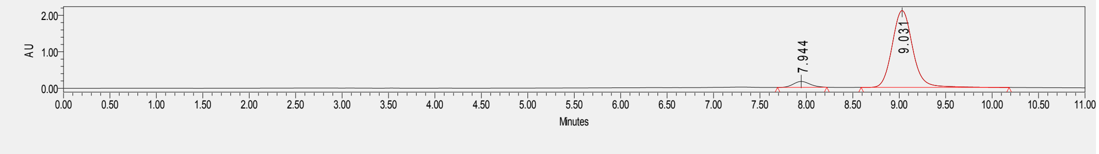

	Retention Time	Area	% Area
1	7.944	2004181	5.84
2	9.031	32337164	94.16

Methyl (1*S*,2*S*,3*R*)-2-(6'-fluoro-2'-oxo-2-phenylspiro[cyclopropane-1,3'-indoline]-3-yl)-2-oxoacetate: Prepared according to the general procedure. The compound of **3ga'** was purified by silica gel chromatography (petroleum ether: Et_2O = 1:2) to afford a light yellow oil in 70% yield. HPLC (chiralcel IA, hexane/*i*-PrOH = 80:20, flow rate 1.0 mL/min, λ = 254 nm) t_r(major) = 10.16 min, t_r(minor) = 8.45 min, *ee* = 89%, dr > 99:1. $[\alpha]^{14.5}_D$ = 234.5 (c = 0.68, in CH_2Cl_2). ^{1}H NMR (400

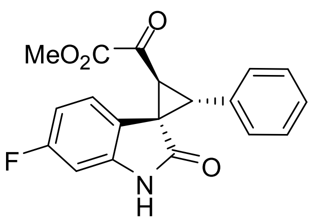

MHz, $CDCl_3$) δ = 9.12 (s, 1H), 7.35 – 7.26 (m, 6H), 6.75 – 6.66 (m, 1H), 6.48 (dd, *J* = 8.6, 2.3 Hz, 1H), 4.33 (d, *J* = 8.3 Hz, 1H), 3.94 (d, *J* = 8.3 Hz, 1H), 3.87 (s, 3H). ^{13}C NMR (101 MHz, $CDCl_3$) δ = 187.4, 173.8, 162.9 (d, *J* = 244.5 Hz), 160.3, 142.5 (d, *J* = 12.0 Hz), 132.3, 129.1, 128.3, 128.0, 124.1 (d, *J* = 9.7 Hz), 120.7 (d, *J* = 2.8 Hz), 108.8 (d, *J* = 12.4 Hz), 99.0 (d, *J* = 27.2 Hz), 53.6, 43.6, 41.0, 40.6. HRMS (ESI-TOF) calcd for $C_{19}H_{14}FNO_4$ (M+Na^+) = 362.0805, found 362.0807.

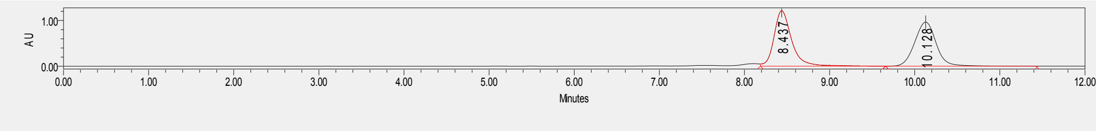

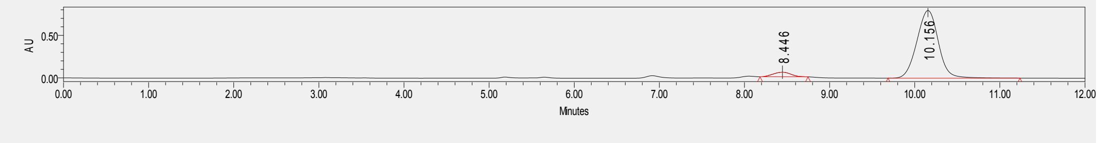

	Retention Time	Area	% Area
1	8.446	786510	5.34
2	10.156	13929911	94.66

Methyl (1*S*,2*S*,3*R*)-2-(-6'-chloro-2'-oxo-2-phenylspiro[cyclopropane-1,3'-indoline]-3-yl)-2-oxoacetate:

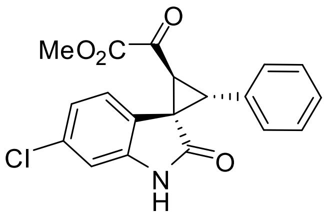

Prepared according to the general procedure. The compound of **3ha'** was purified by silica gel chromatography (petroleum ether: Et_2O = 2:1) to afford a colorless solid in 63% yield. HPLC (chiralcel IA, hexane/*i*-PrOH = 80:20, flow rate 1.0 mL/min, λ = 254 nm) t_r(major) = 11.90 min, t_r(minor) = 9.87 min, *ee* = 84%, dr > 99:1. $[\alpha]^{14.5}_D$ = 222.6 (c = 0.38, in CH_2Cl_2). ^1H NMR (400 MHz, $CDCl_3$) δ = 8.83 (s, 1H), 7.39 – 7.26 (m, 6H), 7.00 (dd, *J* = 8.2, 1.7 Hz, 1H), 6.78 (d, *J* = 1.6 Hz, 1H), 4.36 (d, *J* = 8.3 Hz, 1H), 3.95 (d, *J* = 8.3 Hz, 1H), 3.88 (s, 3H). ^{13}C NMR (101 MHz, $CDCl_3$) δ = 187.3, 173.2, 160.3, 142.2, 134.1, 132.0, 129.1, 128.3, 128.0, 123.8, 123.8 122.4, 110.8, 53.6, 43.6, 41.2, 40.6. HRMS (ESI-TOF) calcd for $C_{19}H_{14}{}^{35}ClNO_4$ (M+Na^+) = 356.0690, found 356.0690; HRMS (ESI-TOF) calcd for $C_{19}H_{14}{}^{37}ClNO_4$ (M+Na^+) = 358.0660, found 358.0663.

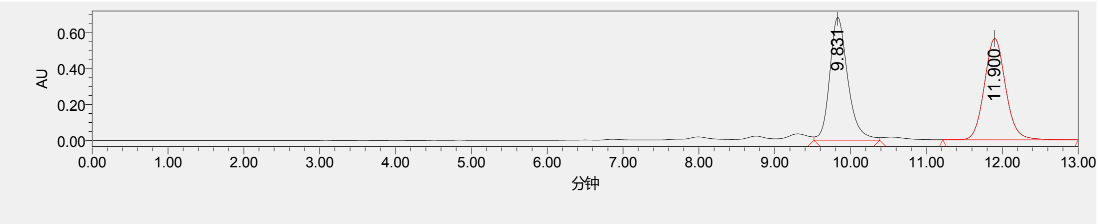

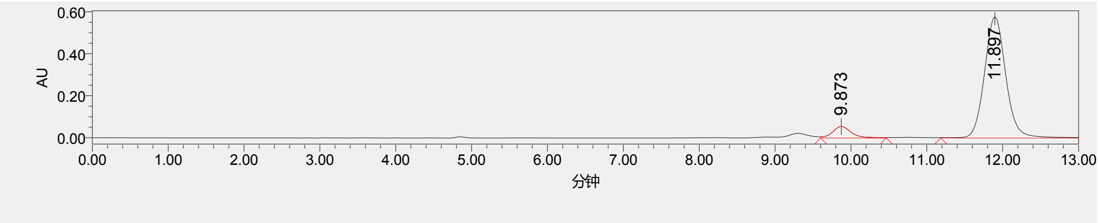

	Retention time	Area	% Area
1	9.873	955517	7.78
2	11.897	11325546	92.22

Methyl (1*R*,2*S*,3*R*) 2-oxo-2-(-2'-oxo-2-phenylspiro[cyclopropane-1,3'-indoline]-3-yl)acetate:

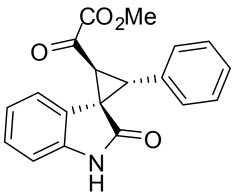

Prepared according to the general procedure. The compound of **3aa** was purified by silica gel chromatography (petroleum ether: EA = 1:1) to afford a colorless solid in 99% yield. Mp. 70.0 – 72.0 ºC. HPLC (chiralcel IE, hexane/*i*-PrOH = 80:20, flow rate 1.0 mL/min, λ = 254 nm) t_r(major) = 13.58 min, t_r(minor) = 16.68 min, *ee* = 92%, dr > 99:1. $[\alpha]^{28.0}_{D}$ = 169.7 (c = 0.35, in CH_2Cl_2). ^1H NMR (400 MHz, DMSO) δ = 10.73 (s, 1H), 7.41 – 7.26 (m, 5H), 7.11 (d, *J* = 7.7, 1H), 6.88 (d, *J* = 7.7 Hz, 1H), 6.66 (t, *J* = 7.5 Hz, 1H), 6.11 (d, *J* = 7.5 Hz, 1H), 3.93 (d, *J* = 8.4 Hz, 1H), 3.72 (s, 3H), 3.61 (d, *J* = 8.4 Hz, 1H). ^{13}C NMR (101 MHz, DMSO) δ = 186.4, 174.0, 159.8, 142.4, 133.0, 129.5, 128.4, 127.7, 127.5, 125.6, 120.9, 120.8, 109.6, 52.7, 40.1, 38.6, 36.8. HRMS (ESI-TOF) calcd for $C_{19}H_{15}NO_4$ ($M+Na^+$) = 344.0899, found 344.0898.

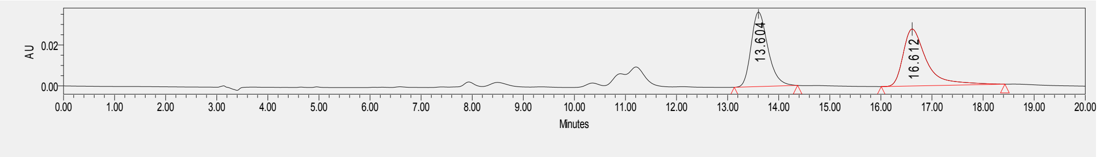

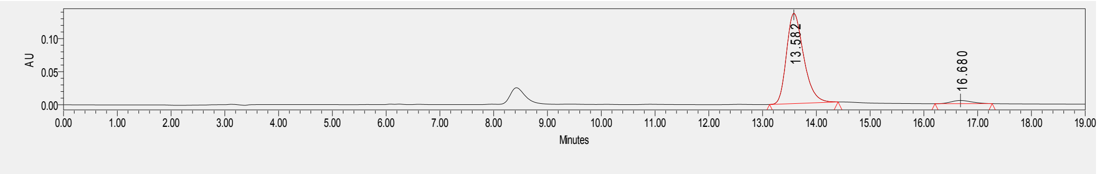

	Retention Time	Area	% Area
1	13.582	3026150	95.91
2	16.680	129163	4.09

Methyl (1*R*,2*S*,3*R*)-2-(4'-methyl-2'-oxo-2-phenylspiro[cyclopropane-1,3'-indoline]-3-yl)-2-oxoacetate:

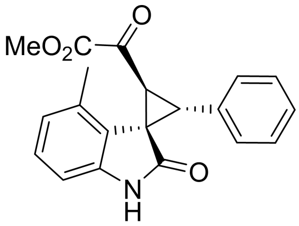

Prepared according to the general procedure. The compound of **3ba** was purified by silica gel chromatography (petroleum ether: EA = 1:1) in 67% yield. ^1H NMR (400 MHz, DMSO) δ = 10.77 (s, 1H), 7.34 – 7.26 (m, 3H), 7.20 (d, *J* = 5.3 Hz, 2H), 7.06 (t, *J* = 7.7 Hz, 1H), 6.79 (d, *J* = 7.7 Hz, 1H), 6.54 (d, *J* = 7.7 Hz, 1H), 4.16 (d, *J* = 8.6 Hz, 1H), 3.73 (s, 3H), 3.40 (d, *J* = 8.6 Hz, 1H), 1.21 (s, 3H). ^{13}C NMR (101 MHz, DMSO) δ = 187.7, 174.8, 159.8, 143.2, 135.4, 133.6, 129.6, 128.5, 127.8, 127.5, 124.3,

122.4, 107.9, 52.6, 40.6, 37.8, 35.5, 18.1. HRMS (ESI-TOF) calcd for $C_{20}H_{17}NO_4$ ($M+H^+$) = 336.1230, found 336.1230.

Methyl (1*R*,2*S*,3*R*)-2-(4'-fluoro-2'-oxo-2-phenylspiro[cyclopropane-1,3'-indoline]-3-yl)-2-oxoacetate:

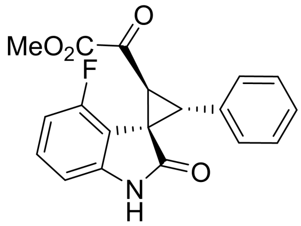

Prepared according to the general procedure. The compound of **3ca** was purified by silica gel chromatography (petroleum ether: EA = 1:1) in 76% yield. HRMS (ESI-TOF) calcd for $C_{19}H_{14}FNO_4$ ($M+H^+$) = 340.0980, found 340.0981.

Methyl (1*R*,2*S*,3*R*)-2-(5'-methyl-2'-oxo-2-phenylspiro[cyclopropane-1,3'-indoline]-3-yl)-2-oxoacetate:

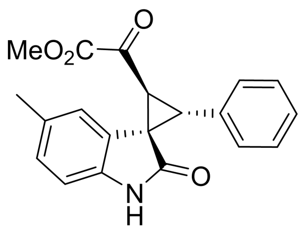

Prepared according to the general procedure. The compound of **3da** was purified by silica gel chromatography (petroleum ether: EA = 1:1) in 96% yield. ^{1}H NMR (400 MHz, DMSO) δ 10.61 (s, 1H), 7.42 – 7.27 (m, 5H), 6.92 (d, J = 7.8 Hz, 1H), 6.76 (d, J = 7.8 Hz, 1H), 5.92 (s, 1H), 3.87 (d, J = 8.4 Hz, 1H), 3.73 (d, J = 8.4 Hz, 3H), 3.58 (d, J = 8.4 Hz, 1H), 1.97 (s, 3H). ^{13}C NMR (101 MHz, DMSO) δ = 186.5, 174.0, 159.8, 140.0, 130.0, 129.5, 129.4, 128.3, 127.8, 127.6, 125.7, 121.7, 109.3, 52.7, 40.1, 38.5, 36.7, 20.6. HRMS (ESI-TOF) calcd for $C_{20}H_{17}NO_4$ ($M+H^+$) = 336.1230, found 336.1234.

Methyl (1*R*,2*S*,3*R*)-2-(5'-fluoro-2'-oxo-2-phenylspiro[cyclopropane-1,3'-indoline]-3-yl)-2-oxoacetate:

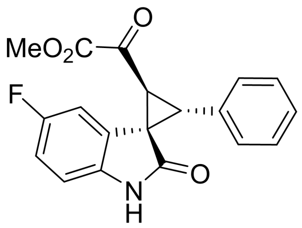

Prepared according to the general procedure. The compound of **3ea** was purified by silica gel chromatography (petroleum ether: EA = 1:1) in 96% yield. HRMS (ESI-TOF) calcd for $C_{19}H_{14}FNO_4$ ($M+H^+$) = 340.0980, found 340.0980.

Methyl (1*R*,2*S*,3*R*)-2-(6'-fluoro-2'-oxo-2-phenylspiro[cyclopropane-1,3'-indoline]-3-yl)-2-oxoacetate:

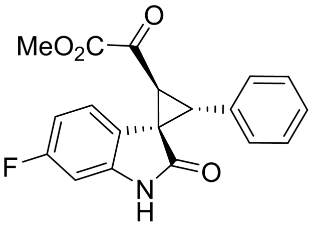

Prepared according to the general procedure. The compound of **3ga** was purified by silica gel chromatography (petroleum ether: EA = 1:1) in 91% yield. HRMS (ESI-TOF) calcd for $C_{19}H_{14}FNO_4$ ($M+H^+$) = 340.0980, found 340.0985.

Methyl (1*R*,2*S*,3*R*)-2-(-6'-chloro-2'-oxo-2-phenylspiro[cyclopropane-1,3'-indoline]-3-yl)-2-oxoacetate:

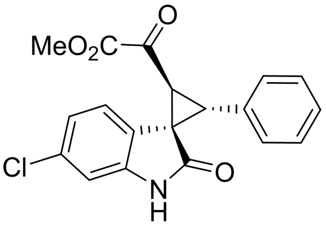

Prepared according to the general procedure. The compound of **3ha** was purified by silica gel chromatography (petroleum ether: EA = 1:1) in 98% yield. ^{1}H NMR (400 MHz, DMSO-d_6) δ = 10.89 (s, 1H), 7.40 -7.25 (m, 5H), 6.91 – 6.85 (m, 1H), 6.75 (d, *J* = 8.1, 1H), 6.07 (d, *J* = 8.1 Hz, 1H), 3.98 (d, *J* = 8.4 Hz, 1H), 3.73 (s, 3H), 3.62 (d, *J* = 8.4 Hz, 1H). ^{13}C NMR (101 MHz, DMSO-d_6) δ = 186.1, 173.9, 159.8, 143.8, 132.7, 132.0, 129.5, 128.5, 127.8, 124.6, 122.2, 120.5, 109.7, 52.8, 39.8, 38.7, 37.0.HRMS (ESI-TOF) calcd for $C_{19}H_{14}{}^{35}ClNO_4$ (M+H$^+$) = 356.0684, found 356.0682; HRMS (ESI-TOF) calcd for $C_{19}H_{14}{}^{37}ClNO_4$ (M+H$^+$) = 358.0655, found 358.0649.

Methyl (1*R*,2*S*,3*R*)-2-(-6'-bromo-2'-oxo-2-phenylspiro[cyclopropane-1,3'-indoline]-3-yl)-2-oxoacetate:

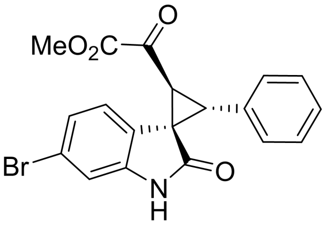

Prepared according to the general procedure. The compound of **3ia** was purified by silica gel chromatography (petroleum ether: EA = 1:1) in 83% yield. ^{1}H NMR (400 MHz, DMSO-d_6) δ 10.88 (s, 1H), 7.38 – 7.26 (m, 5H), 7.03 (s, 1H), 6.92 – 6.84 (m, 1H), 6.02 (d, *J* = 8.4 Hz, 1H), 3.98 (d, *J* = 8.4 Hz, 1H), 3.73 (s, 3H), 3.62 (d, *J* = 8.5 Hz, 1H). ^{13}C NMR (101 MHz, DMSO-d_6) δ 186.1, 173.8, 159.8, 144.0, 132.7, 129.5, 128.5, 127.8, 125.0, 123.4, 122.5, 120.2, 112.5, 52.8, 39.8, 38.7, 37.0. HRMS (ESI-TOF) calcd for $C_{19}H_{14}{}^{79}BrNO_4$ (M+H$^+$) = 400.0179, found 400.0184; HRMS (ESI-TOF) calcd for $C_{19}H_{14}{}^{81}BrNO_4$ (M+H$^+$) = 402.0159, found 402.0163.

Methyl (1*R*,2*S*,3*R*)-2-(2-(2-methoxyphenyl)-2'-oxospiro[cyclopropane-1,3'-indoline]-3-yl)-2-oxoacetate:

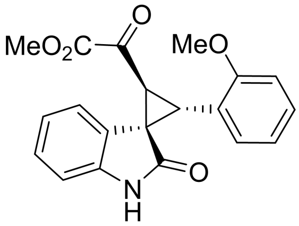

Prepared according to the general procedure. The compound of **3ah** was purified by silica gel chromatography (petroleum ether: EA = 1:1) in 71% yield. HRMS (ESI-TOF) calcd for $C_{20}H_{17}NO_5$ (M+H$^+$) = 352.1179, found 352.1182.

Methyl (1*R*,2*S*,3*R*)-2-(2-(3-methoxyphenyl)-2'-oxospiro[cyclopropane-1,3'-indoline]-3-yl)-2-oxoacetate:

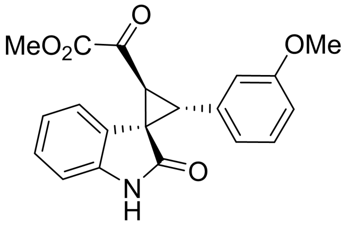

Prepared according to the general procedure. The compound of **3ai** was purified by silica gel chromatography (petroleum ether: EA = 1:1) in 98% yield. HRMS (ESI-TOF) calcd for $C_{20}H_{17}NO_5$ (M+H$^+$) = 352.1179, found 352.1176.

Methyl

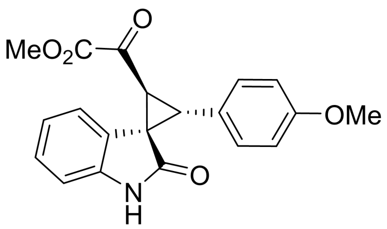

(1*R*,2*S*,3*R*)-2-(2-(4-methoxyphenyl)-2'-oxospiro[cyclopropane-1,3'-indoline]-3-yl)-2-oxoacetate: Prepared according to the general procedure. The compound of **3aj** was purified by silica gel chromatography (petroleum ether: EA = 1:1) in 96% yield. HRMS (ESI-TOF) calcd for $C_{20}H_{17}NO_5$ (M+H$^+$) =

352.1179, found 352.1176.

Methyl (1*R*,2*S*,3*R*)-2-oxo-2-(2'-oxo-2-o-tolylspiro[cyclopropane-1,3'-indoline]-3-yl)acetate:

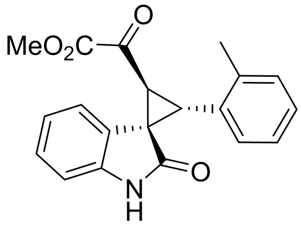

Prepared according to the general procedure. The compound of **3ak** was purified by silica gel chromatography (petroleum ether: EA = 1:1) in 76% yield. HRMS (ESI-TOF) calcd for $C_{20}H_{17}NO_4$ ($M+H^+$) = 336.1230, found 336.1233.

Methyl (1*R*,2*S*,3*R*)-2-oxo-2-(2'-oxo-2-p-tolylspiro[cyclopropane-1,3'-indoline]-3-yl)acetate:

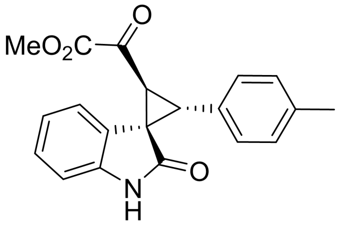

Prepared according to the general procedure. The compound of **3al** was purified by silica gel chromatography (petroleum ether: EA = 1:1) in 93% yield. HRMS (ESI-TOF) calcd for $C_{20}H_{17}NO_4$ ($M+H^+$) = 336.1230, found 336.1230.

Methyl (1*R*,2*S*,3*R*)-2-(-2-(3-chlorophenyl)-2'-oxospiro[cyclopropane-1,3'-indoline]-3-yl)-2-oxoacetate:

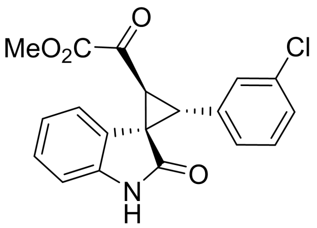

The compound of **3am** was purified by silica gel chromatography (petroleum ether: EA = 1:1) in 76% yield. HRMS (ESI-TOF) calcd for $C_{19}H_{14}{}^{35}ClNO_4$ ($M+H^+$) = 356.0684, found 356.0684; HRMS (ESI-TOF) calcd for $C_{19}H_{14}{}^{37}ClNO_4$ ($M+H^+$) = 358.0655, found 358.0651.

Methyl (1*R*,2*S*,3*R*)-2-(2-(4-chlorophenyl)-2'-oxospiro[cyclopropane-1,3'-indoline]-3-yl)-2-oxoacetate: The

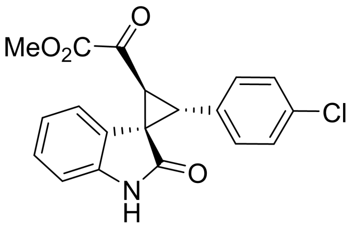

compound of **3an** was purified by silica gel chromatography (petroleum ether: EA = 1:1) in 76% yield. HRMS (ESI-TOF) calcd for $C_{19}H_{14}{}^{35}ClNO_4$ ($M+H^+$) = 356.0684, found 356.0682; HRMS (ESI-TOF) calcd for $C_{19}H_{14}{}^{37}ClNO_4$ ($M+H^+$) = 358.0655, found 358.0648.

Methyl

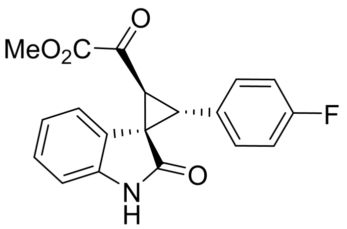

(1*R*,2*S*,3*R*)-2-(2-(4-fluorophenyl)-2'-oxospiro[cyclopropane-1,3'-indoline]-3-yl)-2-oxoacetate: The compound of **3av** was purified by silica gel chromatography (petroleum ether: EA = 1:1) in 96% yield. HRMS (ESI-TOF) calcd for $C_{19}H_{14}FNO_4$ ($M+H^+$) = 340.0980, found 340.0978.

Methyl (1*R*,2*S*,3*R*)-2-(2-(4-bromophenyl)-2'-oxospiro[cyclopropane-1,3'-indoline]-3-yl)-2-oxoacetate:

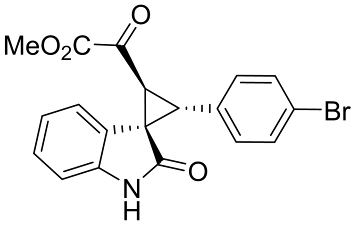

Prepared according to the general procedure. The compound of **3ap** was purified by silica gel chromatography (petroleum ether: EA = 1:1) in 83% yield. HRMS (ESI-TOF) calcd for $C_{19}H_{14}{}^{79}BrNO_4$ (M+H$^+$) = 400.0179, found 400.0179; HRMS (ESI-TOF) calcd for $C_{19}H_{14}{}^{81}BrNO_4$ (M+H$^+$) = 402.0159, found 402.0158.

Methyl (1*R*,2*S*,3*R*)-2-(2-(biphenyl-4-yl)-2'-oxospiro[cyclopropane-1,3'-indoline]-3-yl)-2-oxoacetate:

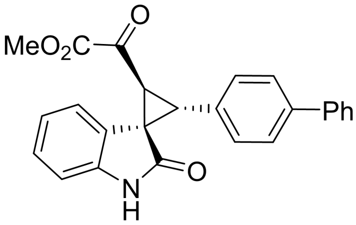

Prepared according to the general procedure. The compound of **3aq** was purified by silica gel chromatography (petroleum ether: EA = 1:1) in 96% yield. HRMS (ESI-TOF) calcd for $C_{25}H_{19}NO_4$ (M+H$^+$) = 398.1387, found 398.1392.

Methyl (1*R*,2*S*,3*R*)-2-(2-(naphthalen-2-yl)-2'-oxospiro[cyclopropane-1,3'-indoline]-3-yl)-2-oxoacetate:

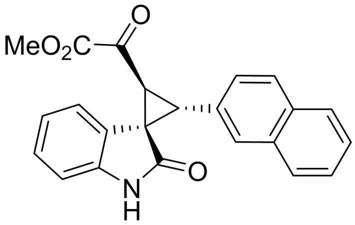

Prepared according to the general procedure. The compound of **3ar** was purified by silica gel chromatography (petroleum ether: EA = 1:1) in 91% yield. HRMS (ESI-TOF) calcd for $C_{23}H_{17}NO_4$ (M+H$^+$) = 372.1230, found 372.1232.

Methyl (1*R*,2*S*,3*R*)3-butyl-2'-oxo-3H-spiro[furan-2,3'-indoline]-5-carboxylate:

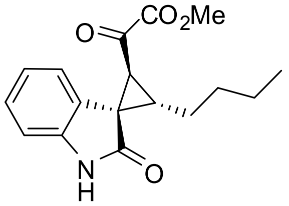

Prepared according to the general procedure. The compound of **3at** was purified by silica gel chromatography (petroleum ether: EA = 1:1) to afford a light yellow oil in 99% yield. HPLC (chiralcel IB, hexane/*i*-PrOH = 80:20, flow rate 1.0 mL/min, λ = 254 nm) t_r(major) = 10.69 min, t_r(minor) = 12.99 min, *ee* = 99%, dr > 99:1. $[\alpha]^{12.5}_D$ = 124.4 (c = 0.62, in CH_2Cl_2). ^{1}H NMR (400 MHz, $CDCl_3$) δ = 8.82 (s, 1H), 7.26 – 7.18 (m, 2H), 7.04 – 6.96 (m, 1H), 6.94 (d, *J* = 7.7 Hz, 1H), 3.82 (s, 3H), 3.75 (d, *J* = 8.0 Hz, 1H), 2.68 (q, *J* = 7.5 Hz, 1H), 2.08 – 1.96 (m, 2H), 1.38 – 1.25 (m, 4H), 0.86 (t, *J* = 6.9 Hz, 3H). ^{13}C NMR (101 MHz, $CDCl_3$) δ = 187.7, 174.8, 160.4, 140.7, 127.8, 126.0, 122.8, 122.4, 110.0, 53.3, 43.0, 42.9, 38.1, 31.3, 24.4, 22.1, 14.0. HRMS (ESI-TOF) calcd for $C_{17}H_{19}NO_4$ (M+H$^+$) = 324.1212, found 324.1219.

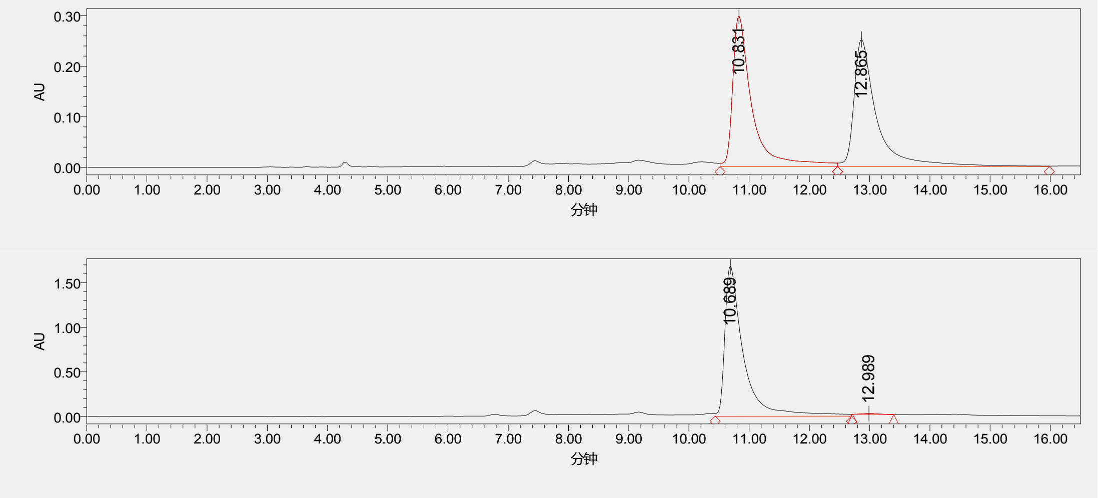

	Retention time	Area	% Area
1	10.689	36241524	99.51
2	12.989	176745	0.49

Methyl (1*R*,2*S*,3*R*)3-cyclohexyl-2'-oxo-3H-spiro[furan-2,3'-indoline]-5-carboxylate:

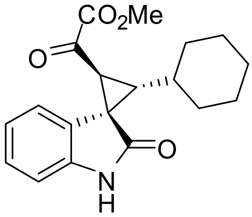

Prepared according to the general procedure. The compound of **3au** was purified by silica gel chromatography (petroleum ether: EA = 1:1) to afford colorless oil in 99% yield. HPLC (chiralcel ID, hexane/*i*-PrOH = 80:20, flow rate 1.0 mL/min, λ = 254 nm) t_r(major) = 8.71 min, t_r(minor) = 10.32 min, *ee* = 99%, dr > 99:1. $[\alpha]^{11.9}_D$ = 158.8 (c = 0.41, in CH_2Cl_2). ^{1}H NMR (400 MHz, $CDCl_3$) δ = 8.86 (s, 1H), 7.24 (s, 1H), 7.11 – 7.03 (m, 2H), 6.98 (d, *J* = 7.8 Hz, 1H), 3.78 (s, 3H), 3.01 (d, *J* = 8.4 Hz, 1H), 2.62 – 2.51 (m, 1H), 1.99 – 1.90 (m, 1H), 1.88 – 1.81 (m, 1H), 1.65 – 1.48 (m, 3H), 1.41 – 1.19 (m, 4H), 1.10 – 0.95 (m, 2H). ^{13}C NMR (101 MHz, $CDCl_3$) δ = 187.0, 175.6, 160.9, 141.3, 127.9, 126.3, 122.2, 121.0, 110.5, 53.0, 41.5, 41.3, 40.0, 36.3, 32.5, 31.9, 26.0, 25.9, 25.5. HRMS (ESI-TOF) calcd for $C_{19}H_{22}NO_4$ $(M+H^+)$ = 328.1549, found 328.1546.

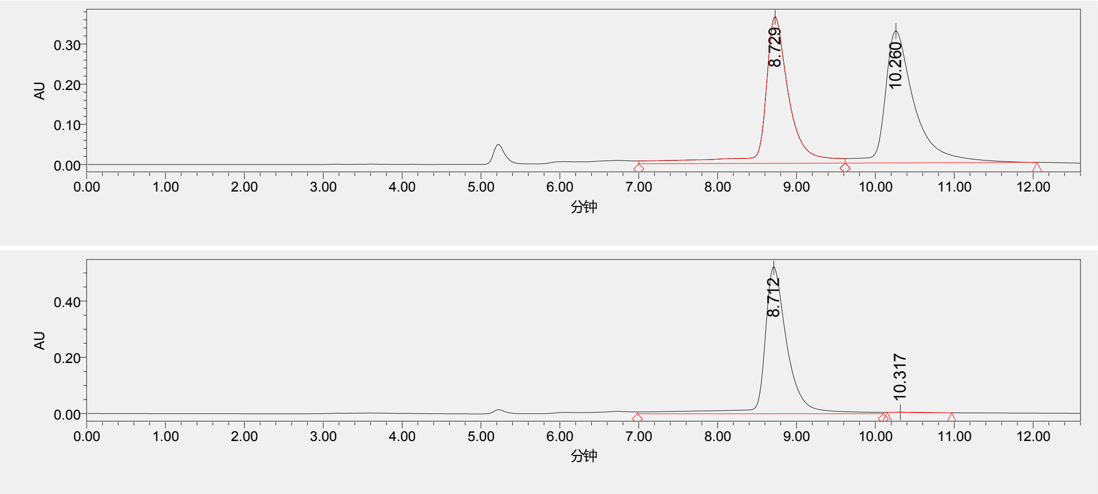

	Retention Time	Area	% Area
1	8.712	10803175	99.86
2	10.317	15507	0.14

1'-isopropyl (1*R*,2*S*,3*R*)-3'-phenylspiro[indoline-3,2'-piperidine]-2,5',6'-trione:

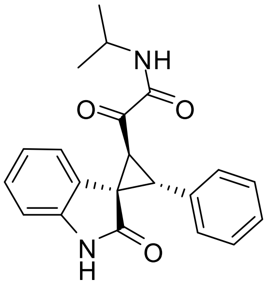

Prepared according to the general procedure. The compound of **4aa** was purified by silica gel chromatography (petroleum ether: EA= 1:1) to afford light yellow oil in 68% yield. HPLC (chiralcel IE, hexane/*i*-PrOH = 70:30, flow rate 1.0 mL/min, λ = 254 nm) t_r(major) = 7.33 min, t_r(minor) = 14.03 min, *ee* = 91%, dr > 99:1. ^{1}H NMR (400 MHz, $CDCl_3$) δ = 8.50 (s, 1H), 7.35 – 7.28 (m, 3H), 7.24 – 7.18 (m, 2H), 7.15 – 7.06 (m, 1H), 6.96 – 6.87 (m, 2H), 6.73 – 6.66 (m, 1H), 6.07 (d, *J* = 7.5 Hz, 1H), 4.07 – 3.96 (m, 2H), 3.94 (d, *J* = 8.6 Hz, 1H), 1.24 – 1.15 (m, 6H). ^{13}C NMR (101 MHz, $CDCl_3$) δ = 190.6, 174.4, 159.0, 141.0, 133.0, 129.8, 128.6, 127.9, 127.7, 125.7, 121.9, 121.7, 109.9, 41.9, 40.8, 38.5, 37.8, 22.3, 22.3. HRMS (ESI-TOF) calcd for $C_{21}H_{20}N_2O_3$ (M+H$^+$) = 349.1552, found 349.1554.

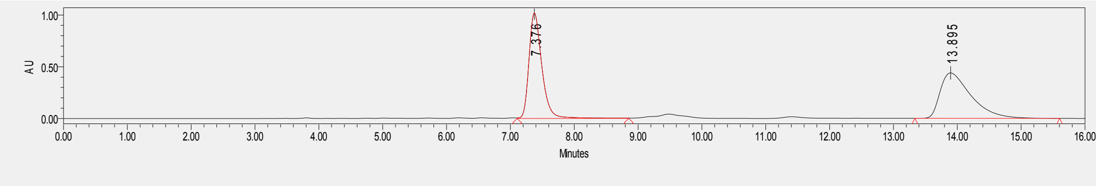

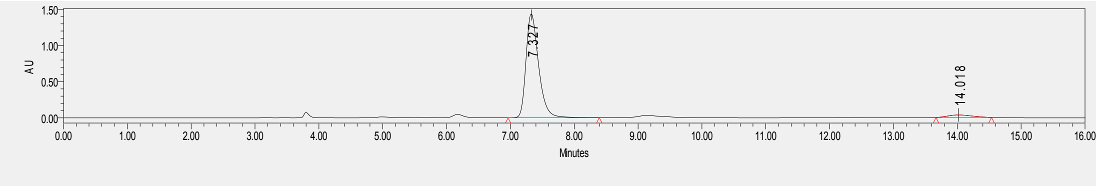

	Retention Time	Area	% Area
1	7.327	19650794	95.59
2	14.018	906620	4.41

3'-phenyl (1*R*,2*S*,3*R*)-1'-propylspiro[indoline-3,2'-piperidine]-2,5',6'-trione:

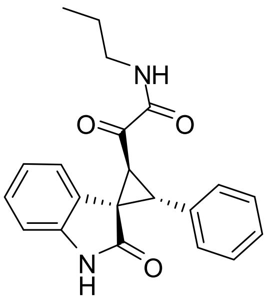

Prepared according to the general procedure. The compound of **4aa′** was purified by silica gel chromatography (petroleum ether: EA= 1:1) to afford light yellow oil in 71% yield. HPLC (chiralcel IE, hexane/*i*-PrOH = 70:30, flow rate 1.0 mL/min, λ = 254 nm) t_r(major) = 9.06 min, t_r(minor) = 16.07 min, *ee* = 92%, dr > 99:1. $[\alpha]^{28.6}_D$ = 236.5 (c = 0.36, in CH_2Cl_2). ^{1}H NMR (400 MHz, $CDCl_3$) δ = 8.56 (s, 1H), 7.36 – 7.27 (m, 3H), 7.24 – 7.18 (m, 2H), 7.14 – 7.06 (m, 2H), 6.87 (d, *J* = 7.7 Hz, 1H), 6.68 (td, *J* = 7.6, 0.9 Hz, 1H), 6.07 (d, *J* = 7.6 Hz, 1H), 4.01 (d, *J* = 8.6 Hz, 1H), 3.93 (d, *J* = 8.6 Hz, 1H), 3.36 – 3.12 (m, 2H), 1.62 – 1.54 (m, 2H), 0.92 (t, *J* = 7.4 Hz, 3H). ^{13}C NMR (101 MHz, $CDCl_3$) δ = 190.6, 174.4, 159.9, 141.1, 133.0, 129.8, 128.6, 128.0, 127.8, 125.7, 121.9, 121.7, 110.0, 41.2, 40.7, 38.6, 37.7, 22.5, 11.3. HRMS (ESI-TOF) calcd for $C_{21}H_{20}N_2O_3$ (M+Na$^+$) = 371.1372, found 371.1364.

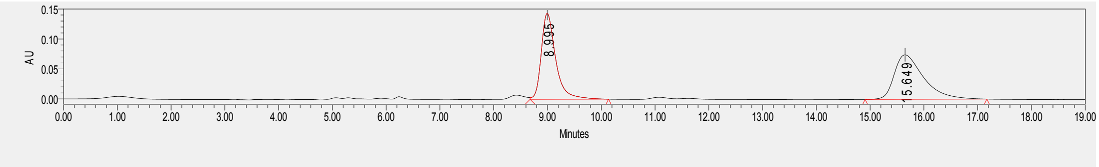

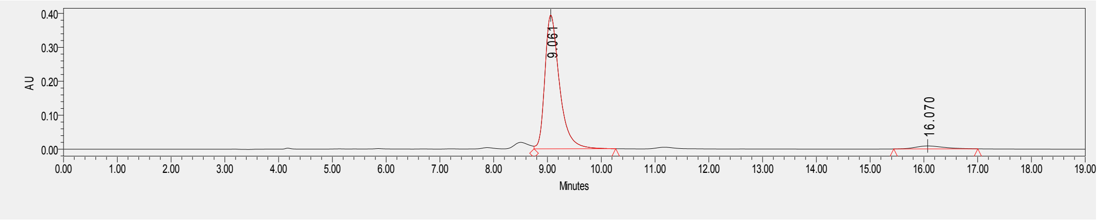

	Retention Time	Area	% Area
1	9.061	7437063	95.87
2	16.070	320587	4.13

1'-isopropyl (1*R*,2*S*,3*R*)-3'-(2-methoxyphenyl)spiro[indoline-3,2'-piperidine]-2,5',6'-trione:

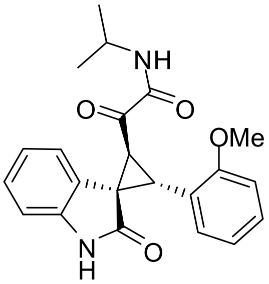

Prepared according to the general procedure. The compound of **4ah** was purified by silica gel chromatography (petroleum ether: EA= 1:1) to afford red oil in 59% yield. HPLC (chiralcel IE, hexane/*i*-PrOH = 70:30, flow rate 1.0 mL/min, λ = 254 nm) t_r(major) = 9.67 min, t_r(minor) = 14.38 min, *ee* = 91%, dr = 94:6. $[\alpha]^{14.1}_D$ = 183.9 (c = 1.36, in CH_2Cl_2). ^1H NMR (400 MHz, $CDCl_3$) δ = 7.96 (s, 1H), 7.35 – 7.25 (m, 2H), 7.10 – 7.03 (m, 1H), 7.02 – 6.95 (m, 1H), 6.94 – 6.82 (m, 2H), 6.74 – 6.68 (m, 1H), 6.67 – 6.61 (m, 1H), 6.00 (d, *J* = 8.0 Hz, 1H), 4.07 – 3.95 (m, 1H), 3.91 (d, *J* = 8.0 Hz, 1H), 3.81 (d, *J* = 8.0 Hz, 1H), 3.36 (s, 3H), 1.24 – 1.15 (m, 6H). ^{13}C NMR (101 MHz, $CDCl_3$) δ = 190.9, 174.5, 159.1, 158.8, 140.7, 129.8, 129.4, 127.3, 126.6, 122.1, 121.6, 120.6, 120.3, 110.7, 109.3, 55.2, 41.8, 40.4, 38.3, 34.7, 22.4, 22.3. HRMS (ESI-TOF) calcd for $C_{22}H_{22}N_2O_4$ (M+H$^+$) = 379.1658, found 379.1653.

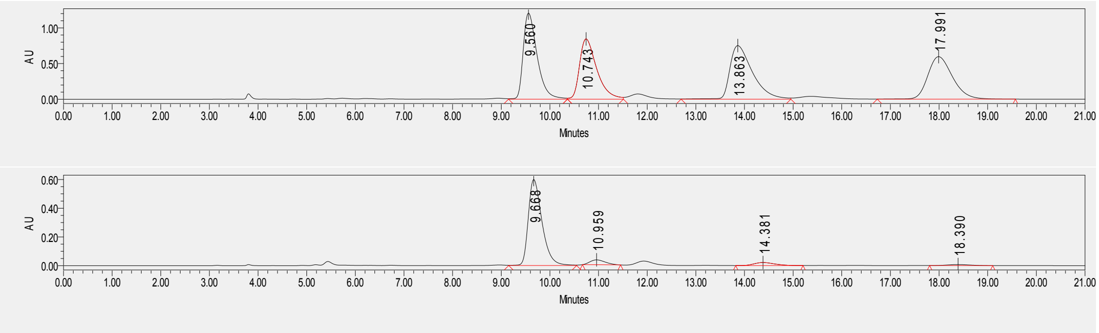

	Retention Time	Area	% Area
1	9.668	11725047	88.03
2	10.959	749804	5.63
3	14.381	618224	4.64
4	18.390	226498	1.70

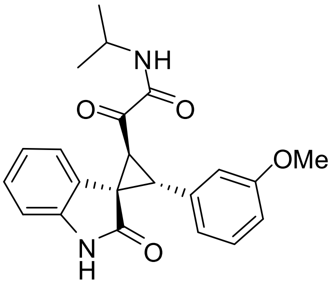

1'-isopropyl (1*R*,2*S*,3*R*)-3'-(3-methoxyphenyl)spiro[indoline-3,2'-piperidine]-2,5',6'-trione:

Prepared according to the general procedure. The compound of **4ai** was purified by silica gel chromatography (petroleum ether: EA= 1:1) to afford colorless oil in 62% yield. HPLC (chiralcel IE,

hexane/*i*-PrOH = 70:30, flow rate 1.0 mL/min, λ = 254 nm) t_r(major) = 9.36 min, t_r(minor) = 17.99 min, *ee* = 95%, dr > 99:1. $[\alpha]^{14.4}_D$ = 189.5 (c = 0.26, in CH_2Cl_2). ^{1}H NMR (400 MHz, $CDCl_3$) δ = 8.18 (s, 1H), 7.26 – 7.18 (m, 1H), 7.15 – 7.06 (m, 1H), 6.93 – 6.77 (m, 4H), 6.77 – 6.66 (m, 2H), 6.17 (d, *J* = 7.6 Hz, 1H), 4.17 – 3.96 (m, 2H), 3.93 (d, *J* = 8.6 Hz, 1H), 3.74 (s, 3H), 1.25 – 1.13 (m, 6H). ^{13}C NMR (101 MHz, $CDCl_3$) δ = 190.5, 174.1, 159.7, 159.0, 140.9, 134.5, 129.6, 127.8 125.7, 122.0, 122.0, 121.8, 115.3, 113.6, 109.8, 55.3, 41.9, 40.8, 38.5, 37.7, 22.4, 22.3. HRMS (ESI-TOF) calcd for $C_{22}H_{22}N_2O_4$ (M+H$^+$) = 379.1658, found 379.1652.

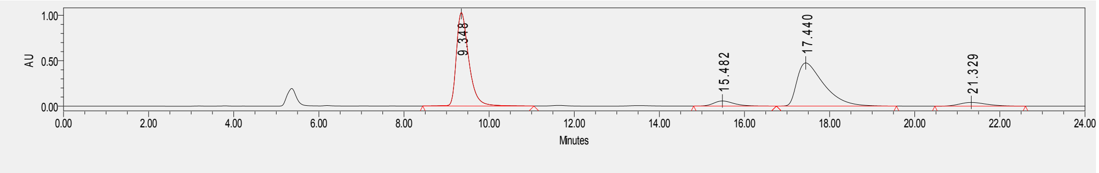

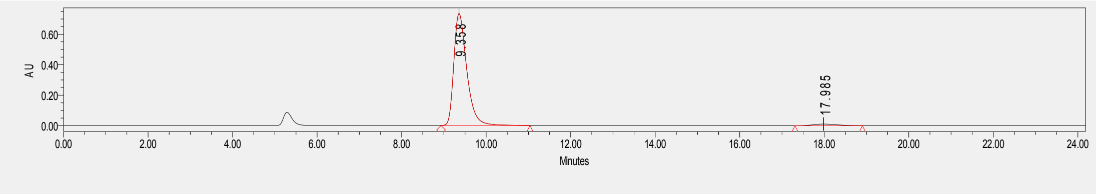

	Retention Time	Area	% Area
1	9.358	15657627	97.44
2	17.985	410938	2.56

1'-isopropyl (1*R*,2*S*,3*R*)-3'-(4-methoxyphenyl)spiro[indoline-3,2'-piperidine]-2,5',6'-trione:

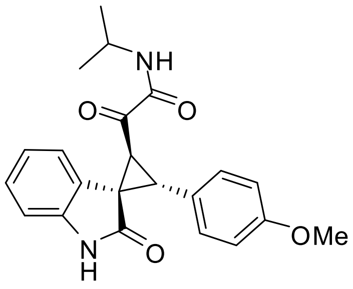

Prepared according to the general procedure. The compound of **4aj** was purified by silica gel chromatography (petroleum ether: EA= 1:1) to afford light yellow oil in 63% yield. HPLC (chiralcel IE, hexane/*i*-PrOH = 70:30, flow rate 1.0 mL/min, λ = 254 nm) t_r(major) = 10.47 min, t_r(minor) = 21.24 min, *ee* = 72%, dr > 99:1. $[\alpha]^{14.4}_D$ = 136.7 (c = 0.22, in CH_2Cl_2). ^{1}H NMR (400 MHz, $CDCl_3$) δ = 8.39 (s, 1H), 7.18 – 7.05 (m, 3H), 6.94 – 6.79 (m, 4H), 6.71 (t, *J* = 7.6 Hz, 1H), 6.11 (d, *J* = 7.6 Hz, 1H), 4.06 – 3.93 (m, 2H), 3.90 (dd, *J* = 8.6, 3.7 Hz, 1H), 3.80 (s, 3H), 1.24 – 1.15 (m, 6H). ^{13}C NMR (101 MHz, $CDCl_3$) δ = 190.7, 174.4, 159.2, 159.0, 141.0, 130.9, 127.7, 125.8, 125.0, 121.9, 121.9, 113.9, 109.8, 55.3, 41.9, 40.9, 38.8, 37.3, 22.3, 22.3. HRMS (ESI-TOF) calcd for $C_{22}H_{22}N_2O_4$ (M+Na$^+$) = 401.1477, found 401.1476.

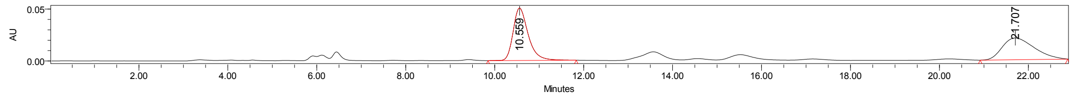

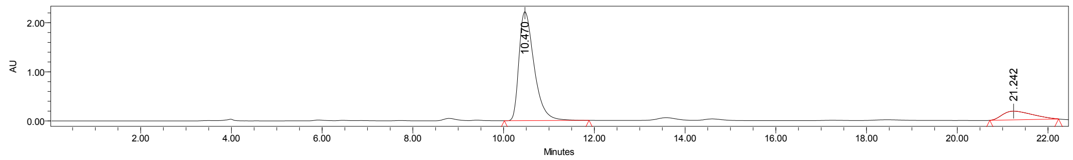

	Retention Time	Area	% Area
1	10.470	51302885	86.29

2	21.242	8148746	13.71

1'-isopropyl (1*R*,2*S*,3*R*)-3'-o-tolylspiro[indoline-3,2'-piperidine]-2,5',6'-trione:

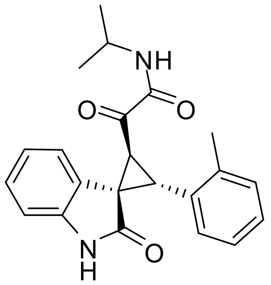

repared according to the general procedure. The compound of **4ak** was purified by silica gel chromatography (petroleum ether: EA= 1:1) to afford light yellow oil in 47% yield. HPLC (chiralcel IE, hexane/*i*-PrOH = 70:30, flow rate 1.0 mL/min, λ = 254 nm) t_r(major) = 7.25 min, t_r(minor) = 10.56 min, *ee* = 96%, dr > 99:1. $[\alpha]^{14.1}_D$ = 180.0 (c = 0.87, in CH_2Cl_2). ^{1}H NMR (400 MHz, $CDCl_3$) δ = 8.84 (s, 1H), 7.39 – 7.33 (m, 1H), 7.26 – 7.18 (m, 2H), 7.10 – 7.03 (m, 2H), 6.94 (d, *J* = 8.1 Hz, 1H), 6.86 (d, *J* = 7.7 Hz, 1H), 6.64 (t, *J* = 7.6 Hz, 1H), 5.99 (d, *J* = 7.5 Hz, 1H), 4.10 – 3.96 (m, 2H), 3.85 (d, *J* = 8.6 Hz, 1H), 1.85 (s, 3H), 1.24 – 1.13 (m, 6H). ^{13}C NMR (101 MHz, $CDCl_3$) δ = 190.7, 174.5, 159.0, 140.9, 138.9, 131.8, 130.1, 128.7, 128.2, 127.8, 126.0, 125.7, 121.9, 120.9, 110.0, 41.9, 40.7, 38.4, 37.2, 22.3, 22.3, 19.2. HRMS (ESI-TOF) calcd for $C_{22}H_{22}N_2O_3$ (M+H$^+$) = 363.1709, found 363.1703.

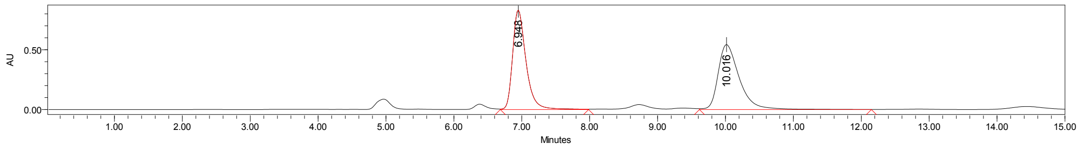

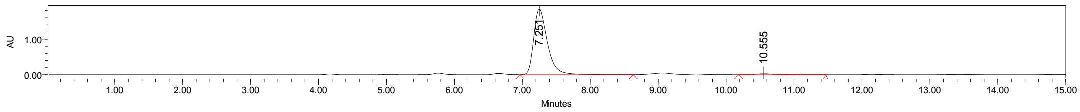

	Retention Time	Area	% Area
1	7.251	26008087	97.95
2	10.555	543636	2.05

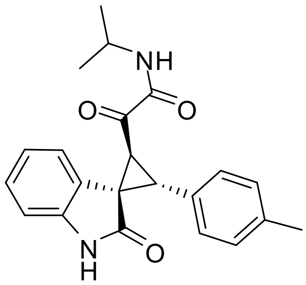

1'-isopropyl (1*R*,2*S*,3*R*)-3'-p-tolylspiro[indoline-3,2'-piperidine]-2,5',6'-trione: Prepared according to the general procedure. The compound of **4al** was purified by silica gel chromatography (petroleum ether: EA= 1:1) to afford white solid in 60% yield. Mp. 172.0 – 174.0 °C, HPLC (chiralcel IE, hexane/*i*-PrOH = 70:30, flow rate 1.0 mL/min, λ = 254 nm) t_r(major) = 8.04 min, t_r(minor) = 16.07 min, *ee* = 87%, dr > 99:1. $[\alpha]^{14.9}_D$ = 169.0 (c = 0.43, in CH_2Cl_2). ^{1}H NMR (400 MHz, $CDCl_3$) δ = 8.39 (s, 1H), 7.15 – 7.05 (m, 5H), 6.95 – 6.85 (m, 2H), 6.75 – 6.65 (m, 1H), 6.11 (d, *J* = 7.5 Hz, 1H), 4.07 – 3.95 (m, 2H), 3.92 (dd, *J* = 8.6, 3.5 Hz, 1H), 2.34 (d, *J* = 6.0 Hz, 3H), 1.20 (d, *J* = 6.5 Hz, 3H), 1.16 (d, *J* = 6.7 Hz, 3H). ^{13}C NMR (101 MHz, $CDCl_3$) δ = 190.7, 174.4, 159.0, 141.0, 137.7, 129.9, 129.6, 129.3, 127.7, 125.8, 121.9, 121.8, 109.8 , 41.9, 40.9, 38.6, 37.7, 22.3, 22.3, 21.2. HRMS (ESI-TOF) calcd for $C_{22}H_{22}N_2O_3$ (M+H$^+$) = 385.1258, found 385.1527.

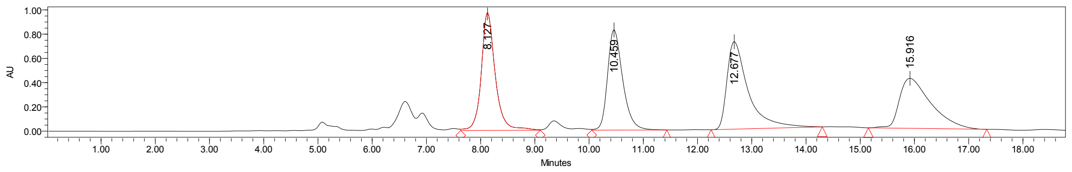

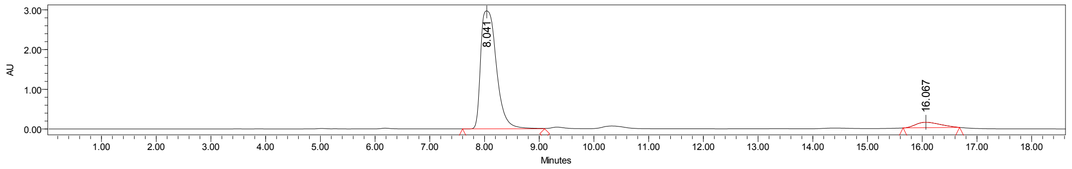

	Retention Time	Area	% Area
1	8.041	59605466	93.27
2	16.067	4302187	6.73

(1*R*,2*S*,3*R*)-3'-(4-fluorophenyl)-1'-isopropylspiro[indoline-3,2'-piperidine]-2,5',6'-trione:

Prepared according to the general procedure. The compound of **4av** was purified by silica gel chromatography (petroleum ether: EA= 1:1) to afford colorless oil in 46% yield. HPLC (chiralcel IE, hexane/*i*-PrOH = 70:30, flow rate 1.0 mL/min, λ = 254 nm) t_r(major) = 6.576 min, t_r(minor) = 12.844 min, *ee* = 90%, dr > 99:1. $[\alpha]^{14.4}_D$ = 157.7 (c = 0.26, in CH_2Cl_2). ^1H NMR (400 MHz, $CDCl_3$) ^1H NMR (400 MHz, $CDCl_3$) δ = 8.35 (s, 1H), 7.22 – 7.15 (m, 2H), 7.15 – 7.09 (m, 1H), 7.05 – 6.96 (m, 2H), 6.92 – 6.85 (m, 2H), 6.76 – 6.67 (m, 1H), 6.06 (d, *J* = 7.6 Hz, 1H), 4.10 – 3.91 (m, 2H), 3.90 – 3.82 (m, 1H), 1.25 – 1.12 (m, 6H). ^{13}C NMR (101 MHz, $CDCl_3$) δ = 190.5, 174.2, 162.3 (d, *J* = 243.6 Hz), 158.9, 141.0, 131.5 (d, *J* = 8.2 Hz), 128.9 (d, *J* = 3.3 Hz), 127.8, 125.5, 122.0, 121.7, 115.7, 115.5, 110.0, 41.9, 40.6, 38.75, 37.0, 22.3, 22.3. HRMS (ESI-TOF) calcd for $C_{21}H_{19}FN_2O_3$ $(M+H^+)$ = 389.1277, found 389.1272.

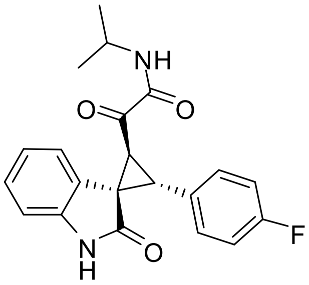

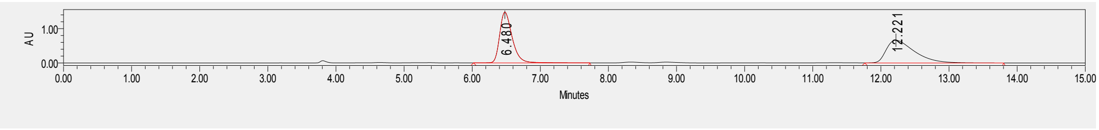

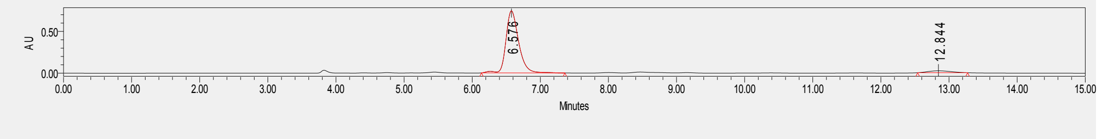

	Retention Time	Area	% Area
1	6.576	9588612	94.76
2	12.844	529960	5.24

(1*R*,2*S*,3*R*)-3'-(3-chlorophenyl)-1'-isopropylspiro[indoline-3,2'-piperidine]-2,5',6'-trione：

Prepared according to the general procedure. The compound of **4am** was purified by silica gel chromatography (petroleum ether: EA= 1:1) to afford colorless oil in 50% yield. HPLC (chiralcel IE, hexane/*i*-PrOH = 70:30, flow rate 1.0 mL/min, λ = 254 nm) t_r(major) = 6.68 min, t_r(minor) =

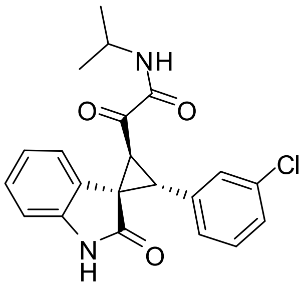

12.05 min, *ee* = 85%, dr > 99:1. $[\alpha]^{13.5}_{D}$ = 144.4 (c = 0.47, in CH_2Cl_2). ^{1}H NMR (400 MHz, $CDCl_3$) δ = 8.45 (d, *J* = 14.1 Hz, 1H), 7.28 – 7.24 (m, 3H), 7.17 – 7.02 (m, 2H), 6.95 – 6.85 (m, 2H), 6.78 – 6.69 (m, 1H), 6.11 (d, *J* = 7.6 Hz, 1H), 4.01 (dd, *J* = 14.3, 6.8 Hz, 1H), 3.96 (d, *J* = 8.5 Hz, 1H), 3.90 (t, *J* = 6.8 Hz, 1H), 1.22 – 1.15 (m, 6H). ^{13}C NMR (101 MHz, $CDCl_3$) δ = 190.3, 174.0, 158.9, 141.1, 135.2, 134.5, 129.9, 129.8, 128.3, 128.1, 128.0, 125.2, 122.1, 121.7, 110.1, 41.9, 40.6, 38.3, 37.0, 22.3, 22.3. HRMS (ESI-TOF) calcd for $C_{21}H_{19}{}^{35}ClN_2O_3$ $(M+H^+)$ = 383.1162, found 383.1154; HRMS (ESI-TOF) calcd for $C_{21}H_{19}{}^{37}ClN_2O_3$ $(M+H^+)$ = 385.1133, found 385.1129.

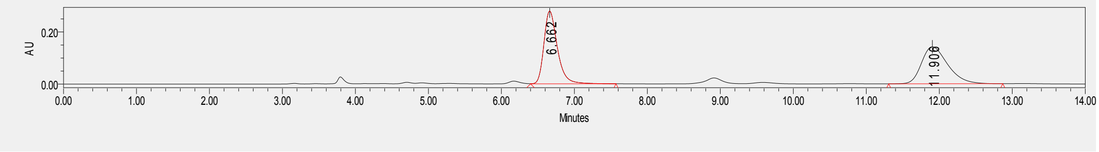

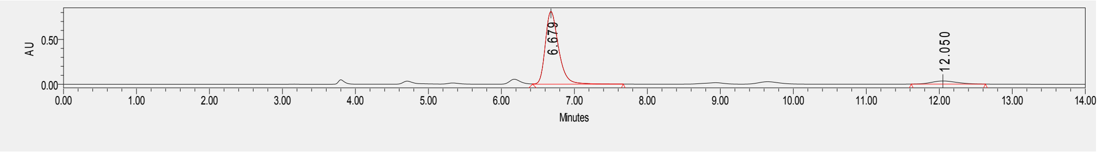

	Retention Time	Area	% Area
1	6.679	10183774	92.42
2	12.050	835828	7.58

(1*R*,2*S*,3*R*)-3'-(4-chlorophenyl)-1'-isopropylspiro[indoline-3,2'-piperidine]-2,5',6'-trione:

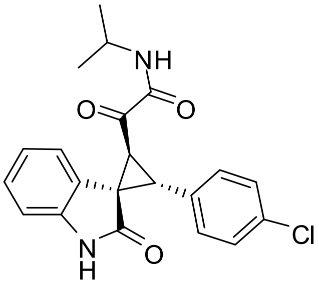

Prepared according to the general procedure. The compound of **4an** was purified by silica gel chromatography (petroleum ether: EA= 1:1) to afford white solid in 44% yield. Mp. 108.0 – 110.0 ºC, HPLC (chiralcel IE, hexane/*i*-PrOH = 70:30, flow rate 1.0 mL/min, λ = 254 nm) t_r(major) = 9.27 min, t_r(minor) = 21.00 min, *ee* = 93%, dr > 99:1. $[\alpha]^{14.0}_{D}$ = 192.8 (c = 0.77, in CH_2Cl_2). ^{1}H NMR (400 MHz, $CDCl_3$) δ = 8.80 (s, 1H), 7.29 (s, 2H), 7.20 – 7.06 (m, 3H), 6.97 – 6.85 (m, 2H), 6.76 – 6.89 (m, 1H), 6.08 (d, *J* = 7.5 Hz, 1H), 4.00 (m, 1H), 3.94 (d, *J* = 8.5 Hz, 1H), 3.87 (d, *J* = 8.6 Hz, 1H), 1.24 – 1.14 (m, 6H). ^{13}C NMR (101 MHz, $CDCl_3$) δ = 190.4, 174.3, 159.0, 141.2, 133.9, 131.7, 131.1, 128.8, 128.0, 125.3, 122.0, 121.7, 110.2, 42.0, 40.6, 38.5, 37.0, 22.3, 22.3. HRMS (ESI-TOF) calcd for $C_{21}H_{19}{}^{35}ClN_2O_3$ $(M+Na^+)$ = 405.0982, found 405.0980; calcd for $C_{21}H_{19}{}^{37}ClN_2O_3$ $(M+Na^+)$ = 407.0952, found 407.0966.

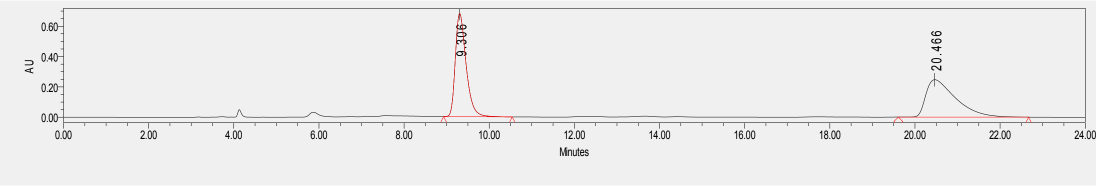

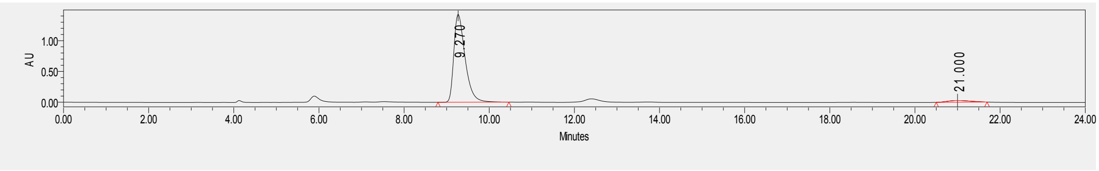

	Retention Time	Area	% Area
1	9.270	26209774	96.21
2	21.000	1033455	3.79

(1*R*,2*S*,3*R*)-3'-(4-bromophenyl)-1'-isopropylspiro[indoline-3,2'-piperidine]-2,5',6'-trione:

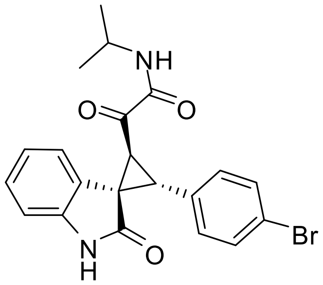

Prepared according to the general procedure. The compound of **4ap** was purified by silica gel chromatography (petroleum ether: EA= 1:1) to afford white solid in 42% yield. Mp. 108.0 – 110.0 ºC, HPLC (chiralcel IE, hexane/*i*-PrOH = 70:30, flow rate 1.0 mL/min, λ = 254 nm) t_r(major) = 7.05 min, t_r(minor) = 14.06 min, *ee* = 94%, dr > 99:1. $[\alpha]^{14.4}_D$ = 214.9 (c = 0.40, in CH_2Cl_2). ^1H NMR (400 MHz, $CDCl_3$) δ = 8.58 (s, 1H), 7.48 – 7.42 (m, 2H), 7.18 – 7.01 (m, 3H), 6.95 – 6.86 (m, 2H), 6.76 – 6.70 (m, 1H), 6.08 (d, *J* = 7.5 Hz, 1H), 4.06 – 3.95 (m, 1H), 3.91 (d, *J* = 8.4 Hz, 1H), 3.86 (d, *J* = 8.4 Hz, 1H), 1.24 – 1.15 (m, 6H). ^{13}C NMR (101 MHz, $CDCl_3$) δ = 190.4, 174.2, 158.9, 141.1, 132.2, 131.8, 131.5, 128.0, 125.3, 122.1, 122.1, 121.7, 110.1, 41.9, 40.5, 38.5, 37.0, 22.3, 22.3. HRMS (ESI-TOF) calcd for $C_{21}H_{19}{}^{79}BrN_2O_3$ (M+Na$^+$) = 449.0477, found 405.0486; calcd for $C_{21}H_{19}{}^{81}BrN_2O_3$ (M+Na$^+$) = 451.0456, found 451.0459.

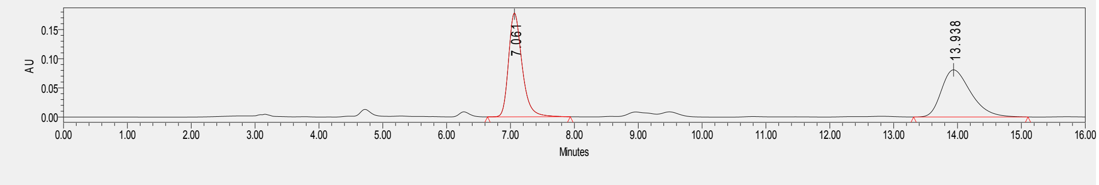

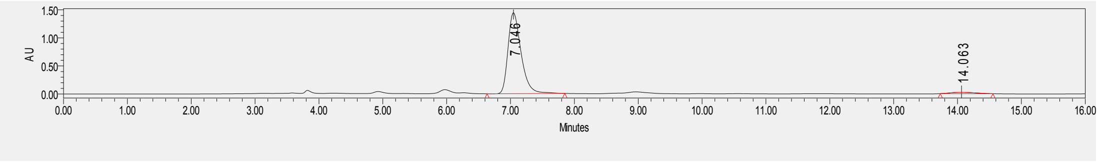

	Retention Time	Area	% Area
1	7.046	20562028	96.84
2	14.063	670147	3.16

(1*R*,2*S*,3*R*)-3'-(biphenyl-4-yl)-1'-isopropylspiro[indoline-3,2'-piperidine]-2,5',6'-trione:

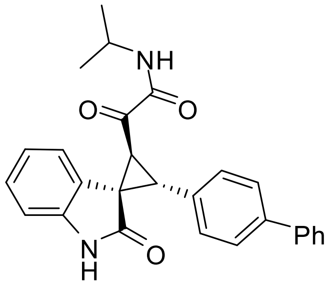

Prepared according to the general procedure. The compound of **4aq** was purified by silica gel chromatography (petroleum ether: EA= 1:1) to afford yellow solid in 51% yield. Mp. 120.0 – 122.0 ºC, HPLC (chiralcel IE, hexane/*i*-PrOH = 70:30, flow rate 1.0 mL/min, λ = 254 nm) t_r(major) = 9.54 min, t_r(minor) = 18.27 min, *ee* = 92%, dr > 99:1. $[\alpha]^{13.4}_D$ = 259.2 (c = 0.37, in CH_2Cl_2). ^1H NMR (400 MHz, $CDCl_3$) δ = 8.56 (s, 1H), 7.63 – 7.53 (m, 4H), 7.47 – 7.40 (m, 2H), 7.37 – 7.31 (t, *J* = 7.3 Hz, 1H), 7.31 – 7.24 (m, 2H), 7.14 – 7.08 (m, 1H), 6.96 – 6.86 (m, 2H), 6.70 (t, *J* = 7.6 Hz, 1H), 6.17 (d, *J* = 7.6 Hz, 1H), 4.09 – 3.95 (m, 3H), 1.24 – 1.16 (m, 6H). ^{13}C NMR (101 MHz, $CDCl_3$) δ = 190.6, 174.4, 159.0, 141.1, 140.7, 140.3, 132.1, 130.2, 128.8, 127.8, 127.5, 127.2, 127.0, 125.7, 122.0, 121.8, 110.0, 41.9, 40.9, 38.6, 37.6, 22.4, 22.3. HRMS (ESI-TOF) calcd for $C_{27}H_{24}N_2O_3$ (M+Na$^+$) = 447.1685, found 447.1688.

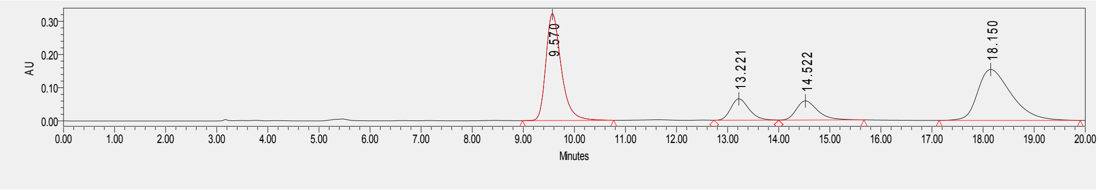

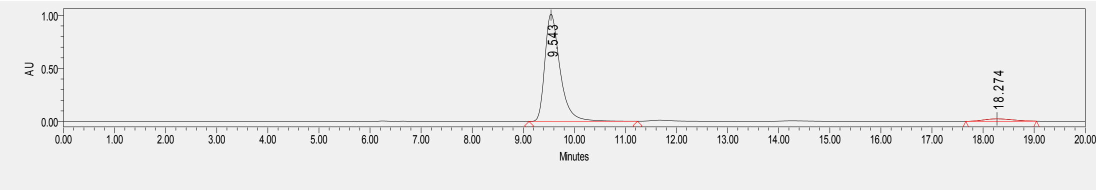

	Retention Time	Area	% Area
1	9.543	20935396	95.90
2	18.274	894005	4.10

1'-isopropyl (1*R*,2*S*,3*R*)-3'-(naphthalen-2-yl)spiro[indoline-3,2'-piperidine]-2,5',6'-trione:

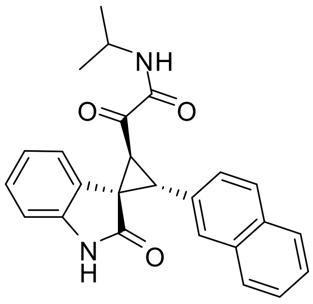

Prepared according to the general procedure. The compound of **4ar** was purified by silica gel chromatography (petroleum ether: EA= 1:1) to afford white solid in 56% yield. Mp. 115.0 – 117.0 ºC, HPLC (chiralcel IE, hexane/*i*-PrOH = 70:30, flow rate 1.0 mL/min, λ = 254 nm) t_r(major) = 8.37 min, t_r(minor) = 15.68 min, *ee* = 93%, dr > 99:1. $[\alpha]^{14.1}_D$ = 264.7 (c = 0.27, in CH_2Cl_2). ^{1}H NMR (400 MHz, $CDCl_3$) δ = 8.27 (s, 1H), 7.87 – 7.76 (m, 3H), 7.74 (d, *J* = 8.5 Hz, 1H), 7.54 – 7.45 (m, 2H), 7.22 (d, *J* = 8.4 Hz, 1H), 7.06 (t, *J* = 7.6 Hz, 1H), 6.96 – 6.85 (m, 2H), 6.65 – 6.55 (m, 1H), 6.06 (d, *J* = 7.6 Hz, 1H), 4.17 (d, *J* = 8.6 Hz, 1H), 4.09 (d, *J* = 8.6 Hz, 1H), 4.04 (m, 1H), 1.25 – 1.17 (m, 6H). ^{13}C NMR (101 MHz, $CDCl_3$) δ = 190.6, 174.2, 159.0, 140.9, 133.2, 132.9, 132.9, 130.7, 128.4, 128.4, 127.9, 127.8, 127.7, 126.4, 126.3, 125.6, 122.1, 121.7, 109.9, 41.9, 40.9, 38.6, 38.0, 22.4, 22.3. HRMS (ESI-TOF) calcd for $C_{25}H_{22}N_2O_3$ (M+Na$^+$) = 421.1528, found 421.1527.

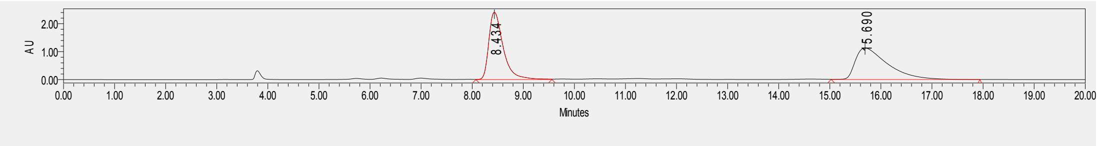

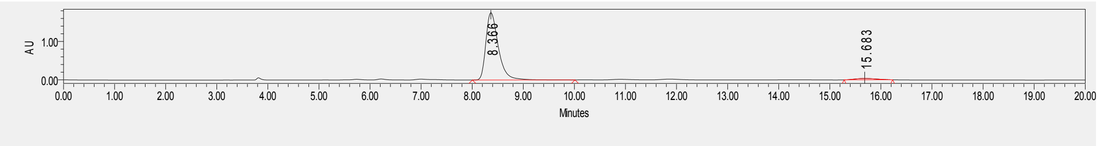

	Retention Time	Area	% Area
1	8.366	29749442	96.54
2	15.683	1066202	3.46

N-isopropyl (1*R*,2*S*,3*R*)-2-(-4'-methyl-2'-oxo-2-phenylspiro[cyclopropane-1,3'-indoline]-3-yl)-2-oxoacetamide: Prepared according to the general procedure. The compound of **4ba** was purified by silica gel chromatography (petroleum ether: EA= 1:1) to afford white oil in 43% yield. HPLC (chiralcel IE,

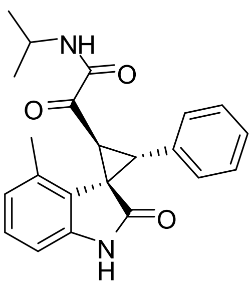

hexane/*i*-PrOH = 70:30, flow rate 1.0 mL/min, λ = 254 nm) t_r(major) = 7.29 min, t_r(minor) = 17.41 min, *ee* = 86%, dr > 99:1. $[\alpha]^{13.7}_{D}$ = 103.2 (c = 0.22, in CH_2Cl_2). ^1H NMR (400 MHz, $CDCl_3$) δ = 8.03 (s, 1H), 7.26 – 7.18 (m, 5H), 7.05 (t, *J* = 7.8 Hz, 1H), 6.85 (d, *J* = 7.9 Hz, 1H), 6.77 (d, *J* = 7.7 Hz, 1H), 6.58 (d, *J* = 7.8 Hz, 1H), 4.17 (d, *J* = 8.8 Hz, 1H), 4.10 – 3.96 (m, 1H), 3.80 (d, *J* = 8.8 Hz, 1H), 1.34 (s, 3H), 1.24 – 1.16 (m, 6H). ^{13}C NMR (101 MHz, $CDCl_3$) δ = 192.3, 175.0, 159.3, 141.6, 135.3, 134.8, 129.7, 128.6, 127.9, 127.7, 125.6, 122.4, 108.0, 41.8, 41.1, 38.8, 35.1, 22.4, 22.3, 18.7. HRMS (ESI-TOF) calcd for $C_{22}H_{22}N_2O_3$ (M+Na$^+$) = 385.1528, found 385.1535.

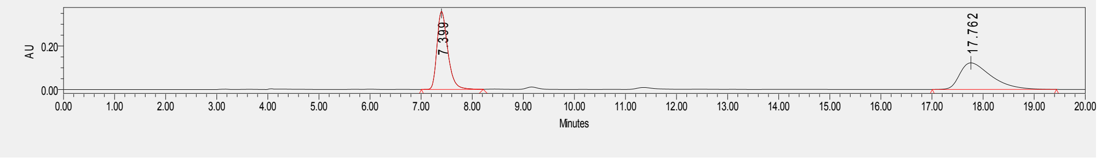

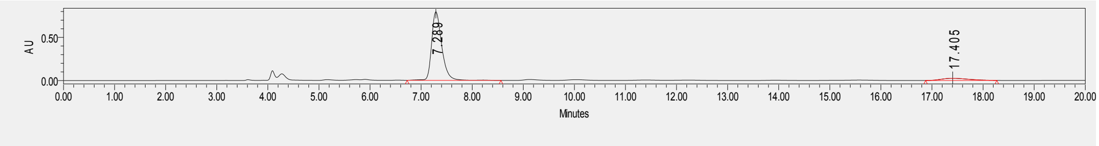

	Retention Time	Area	% Area
1	7.289	10959386	92.25
2	17.405	920811	7.75

(1*R*,2*S*,3*R*)-4-fluoro-1'-isopropyl-3'-phenylspiro[indoline-3,2'-piperidine]-2,5',6'-trione:

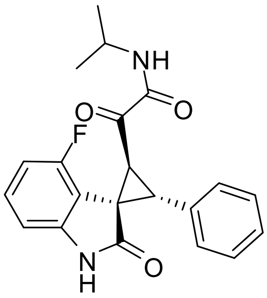

Prepared according to the general procedure. The compound of **4ca** was purified by silica gel chromatography (petroleum ether: EA= 1:1) to afford white solid in 49% yield. Mp. 118.0 – 120.0 ºC, HPLC (chiralcel IE, hexane/*i*-PrOH = 70:30, flow rate 1.0 mL/min, λ = 254 nm) t_r(major) = 6.57 min, t_r(minor) = 13.86 min, *ee* = 86%, dr > 99:1. $[\alpha]^{14.4}_{D}$ = 137.2 (c = 0.24, in CH_2Cl_2). ^1H NMR (400 MHz, $CDCl_3$) δ = 8.45 (s, 1H), 7.32 – 7.26 (m, 2H), 7.25 – 7.17 (m, 2H), 7.13 – 7.02 (m, 1H), 6.89 (d, *J* = 8.0 Hz, 1H), 6.71 (t, *J* = 8.2 Hz, 1H), 6.49 – 6.39 (m, 1H), 4.25 (t, *J* = 8.5 Hz, 1H), 4.08 – 3.95 (m, 1H), 3.88 (t, *J* = 7.0 Hz, 1H), 1.25 – 1.17 (m, 6H). ^{13}C NMR (101 MHz, $CDCl_3$) δ = 191.4, 174.2, 158.9, 157.5 (d, *J* = 247.8 Hz), 143.09 (d, *J* = 8.4 Hz), 134.5, 129.5 (d, *J* = 8.7 Hz), 129.3, 128.3, 127.8, 111.7 (d, *J* = 17.7 Hz), 110.0 (d, *J* = 21.0 Hz), 106.3 (d, *J* = 2.8 Hz), 41.9, 40.3, 38.3, 35.9, 22.4, 22.3. HRMS (ESI-TOF) calcd for $C_{21}H_{19}FN_2O_3$ (M+H$^+$) = 367.1458, found 367.1466.

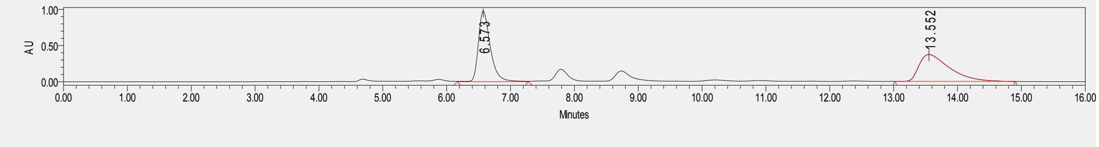

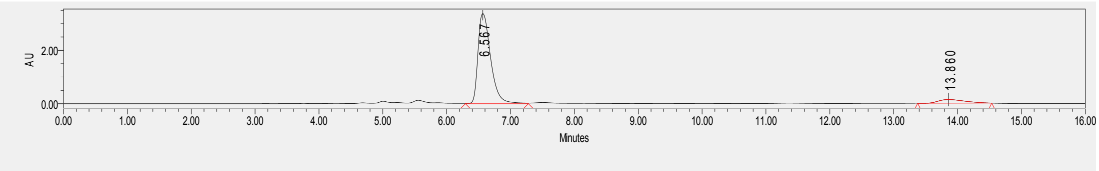

	Retention Time	Area	% Area
1	6.567	46541977	92.25
2	13.860	3909245	7.75

(1*R*,2*S*,3*R*)-1'-isopropyl-5-methyl-3'-phenylspiro[indoline-3,2'-piperidine]-2,5',6'-trione:

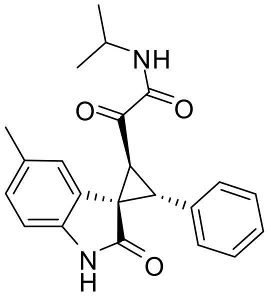

Prepared according to the general procedure. The compound of **4da** was purified by silica gel chromatography (petroleum ether: EA= 1:1) to afford white solid in 64% yield. Mp. 178.0 – 180.0 ºC, HPLC (chiralcel IE, hexane/*i*-PrOH = 70:30, flow rate 1.0 mL/min, λ = 254 nm) t_r(major) = 7.37 min, t_r(minor) = 16.77 min, *ee* = 89%, dr > 99:1. $[\alpha]^{13.1}_D$ = 194.9 (c = 0.39, in CH_2Cl_2). ^{1}H NMR (400 MHz, $CDCl_3$) δ = 8.39 (s, 1H), 7.36 – 7.28 (m, 3H), 7.24 – 7.17 (m, 2H), 6.93 – 6.85 (m, 2H), 6.76 (d, *J* = 7.9 Hz, 1H), 6.76 (d, *J* = 7.9 Hz, 1H), 5.86 (s, 1H), 4.06 – 3.95 (m, 2H), 3.91 (d, *J* = 8.6 Hz, 1H), 2.01 (s, 3H), 1.24 – 1.15 (m, 6H). ^{13}C NMR (101 MHz, $CDCl_3$) δ = 190.6, 174.4, 159.0, 138.6, 133.1, 131.3, 129.8, 128.5, 128.1, 127.9, 125.7, 122.6, 109.6, 41.9, 40.9, 38.5, 37.7, 22.3, 22.3, 21.0. HRMS (ESI-TOF) calcd for $C_{22}H_{22}N_2O_3$ (M+H$^+$) = 363.1709, found 363.1704.

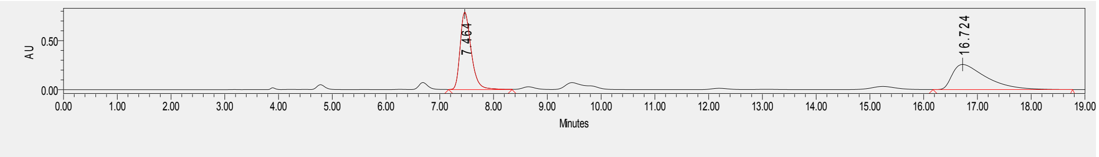

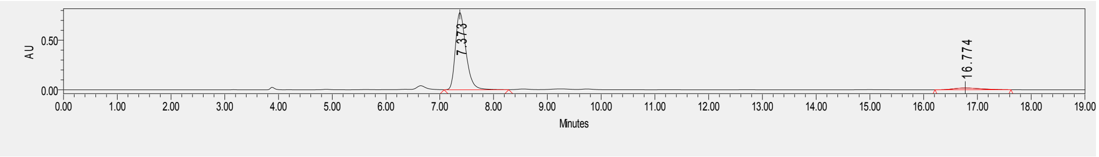

	Retention Time	Area	% Area
1	7.373	10583683	94.41
2	16.774	626430	5.59

(1*R*,2*S*,3*R*)-5-fluoro-1'-isopropyl-3'-phenylspiro[indoline-3,2'-piperidine]-2,5',6'-trione:

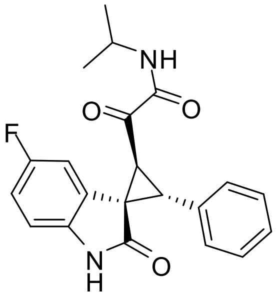

Prepared according to the general procedure. The compound of **4ea** was purified by silica gel chromatography (petroleum ether: EA= 1:1) to afford light yellow solid in 58% yield. Mp. 190.0 – 192.0 ºC, HPLC (chiralcel IE, hexane/*i*-PrOH = 70:30, flow rate 1.0 mL/min, λ = 254 nm) t_r(major) = 6.06 min, t_r(minor) = 12.86 min, *ee* = 81%, dr > 99:1. $[\alpha]^{13.7}_D$ = 165.6 (c = 0.68, in CH_2Cl_2). ^{1}H NMR (400 MHz, $CDCl_3$) δ = 8.54 (s, 1H), 7.38 – 7.30 (m, 3H), 7.25 – 7.15 (m, 2H), 6.90 (d, *J* = 8.1 Hz, 1H), 6.84 – 6.76 (m, 2H), 5.80 (d, *J* = 8.3 Hz, 1H), 4.06 – 3.95 (m, 2H), 3.86 (d, *J* = 8.7 Hz, 1H), 1.25 – 1.13 (m, 6H). ^{13}C NMR (101 MHz, $CDCl_3$) δ = 190.5, 174.4, 158.9, 158.4 (d, *J* = 238.3 Hz), 137.0, 132.5, 129.6, 128.8, 128.3, 127.5 (d, *J* = 9.2 Hz), 114.2 (d, *J* = 23.7 Hz), 110.41 (d, *J* = 8.3 Hz), 109.7 (d, *J* = 25.9 Hz), 42.0, 40.8, 38.7, 37.9, 22.4, 22.3. HRMS (ESI-TOF) calcd for $C_{21}H_{19}FN_2O_3$ (M+H$^+$) = 367.1458, found 367.1460.

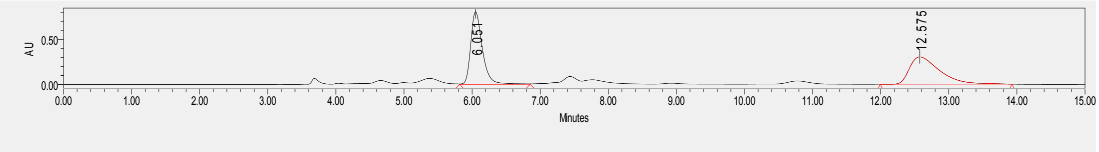

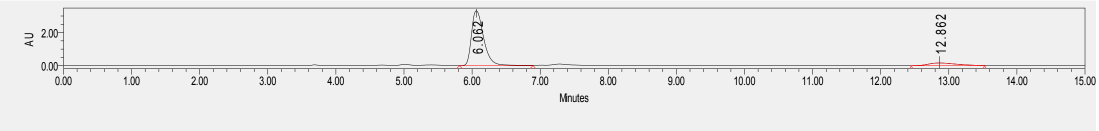

	Retention Time	Area	% Area
1	6.062	41500565	90.47
2	12.862	4370091	9.53

(1*R*,2*S*,3*R*)-6-fluoro-1'-isopropyl-3'-phenylspiro[indoline-3,2'-piperidine]-2,5',6'-trione:

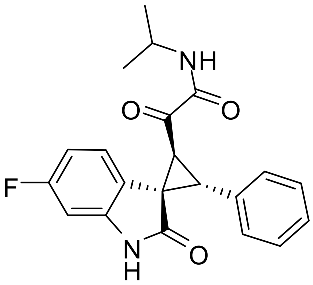

Prepared according to the general procedure. The compound of **4ga** was purified by silica gel chromatography (petroleum ether: EA= 1:1) to afford white solid in 58% yield. Mp. 106.0 – 108.0 ºC, HPLC (chiralcel IE, hexane/*i*-PrOH = 70:30, flow rate 1.0 mL/min, λ = 254 nm) t_r(major) = 5.89 min, t_r(minor) = 10.23 min, *ee* = 90%, dr > 99:1. $[\alpha]^{14.7}_D$ = 156.2 (c = 0.68, in CH_2Cl_2). ^{1}H NMR (400 MHz, $CDCl_3$) δ = 8.99 (s, 1H), 7.35 – 7.28 (m, 3H), 7.24 – 7.15 (m, 2H), 6.97 (d, *J* = 8.1 Hz, 1H), 6.64 – 6.56 (m, 1H), 6.41 – 6.30 (m, 1H), 5.96 (dd, *J* = 8.4, 5.2 Hz, 1H), 4.09 – 3.99 (m, 1H), 3.96 (d, *J* = 8.6 Hz, 1H), 3.87 (d, *J* = 8.6 Hz, 1H), 1.24 – 1.15 (m, 6H). ^{13}C NMR (101 MHz, $CDCl_3$) δ = 190.7, 174.9, 163.6 (d, *J* = 243.3 Hz), 158.9, 142.5 (d, *J* = 12.0 Hz), 132.9, 129.7, 128.6, 128.1, 122.5 (d, *J* = 9.7 Hz), 121.03 (d, *J* = 2.7 Hz), 108.3 (d, *J* = 23.7 Hz), 98.8 (d, *J* = 27.3 Hz), 42.0, 40.3, 38.3, 37.5, 22.3. HRMS (ESI-TOF) calcd for $C_{21}H_{19}FN_2O_3$ (M+H$^+$) = 367.1458, found 367.1460.

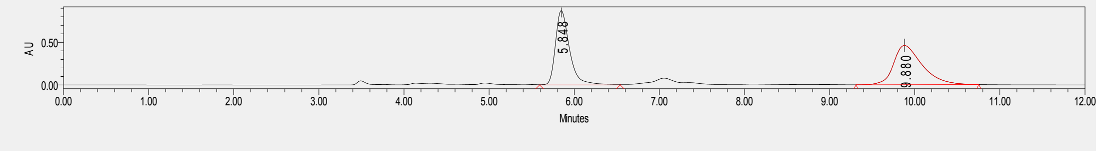

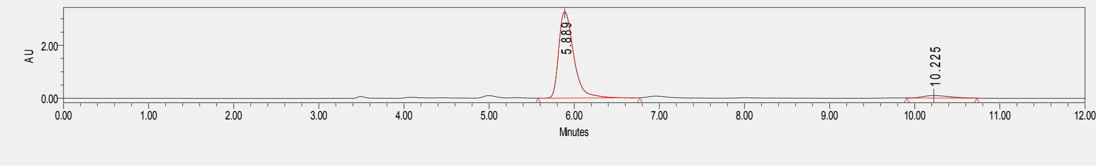

	Retention Time	Area	% Area
1	5.889	39631859	95.20
2	10.225	1996316	4.80

(1*R*,2*S*,3*R*)-6-chloro-1'-isopropyl-3'-phenylspiro[indoline-3,2'-piperidine]-2,5',6'-trione：

Prepared according to the general procedure. The compound of **4ha** was purified by silica gel chromatography (petroleum ether: EA= 1:1) to afford light yellow solid in 67% yield. Mp. 108.0 – 110.0 ºC, HPLC (chiralcel IB, hexane/*i*-PrOH = 80:20, flow rate 1.0 mL/min, λ = 254 nm) t_r(major) = 6.92 min, t_r(minor) = 5.78 min, *ee* = 88%, dr = 98:2. $[\alpha]^{14.6}_D$ = 204.8 (c = 0.42, in CH_2Cl_2). ^{1}H

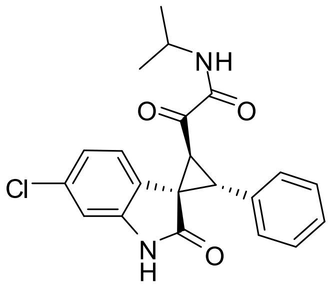

NMR (400 MHz, $CDCl_3$) δ = 8.81 (s, 1H), 7.35 – 7.29 (m, 3H), 7.22 – 7.16 (m, 2H), 6.95 (d, *J* = 8.3 Hz, 1H), 6.85 (d, *J* = 1.6 Hz, 1H), 6.64 (dd, *J* = 8.1, 1.7 Hz, 1H), 5.94 (d, *J* = 8.1 Hz, 1H), 4.08 – 3.96 (m, 1H), 3.97 (d, *J* = 8.5 Hz, 1H), 3.86 (d, *J* = 8.6 Hz, 1H), 1.24 – 1.17 (m, 6H). ^{13}C NMR (101 MHz, $CDCl_3$) δ = 190.6, 174.4, 158.9, 142.2, 133.5, 132.7, 129.7, 128.7, 128.1, 124.1, 122.4, 122.0, 110.6, 42.0, 40.3, 38.4, 37.7, 22.3. HRMS (ESI-TOF) calcd for $C_{21}H_{19}{}^{35}ClN_2O_3$ (M+H$^+$) = 383.1162, found 383.1156; HRMS (ESI-TOF) calcd for $C_{21}H_{19}{}^{37}ClN_2O_3$ (M+H$^+$) = 385.1133, found 385.1142.

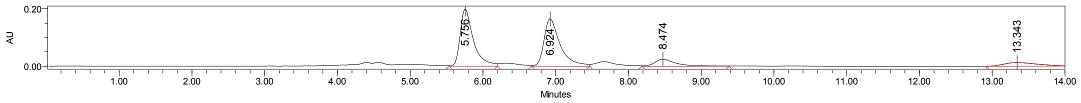

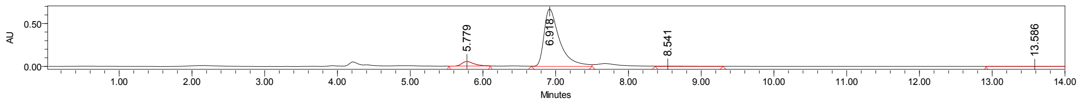

	Retention Time	Area	% Area
1	5.779	605520	5.64
2	6.918	9956371	92.76
3	8.541	142033	1.32
4	13.586	29232	0.27

(1*R*,2*S*,3*R*)-6-bromo-1'-isopropyl-3'-phenylspiro[indoline-3,2'-piperidine]-2,5',6'-trione：

Prepared according to the general procedure. The compound of **4ia** was purified by silica gel

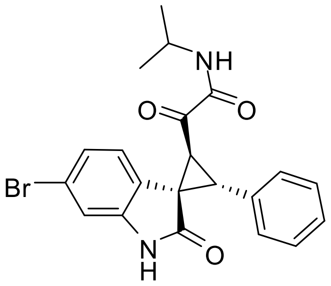

chromatography (petroleum ether: EA= 1:1) to afford light yellow solid in 66% yield. Mp. 116.0 – 118.0 ºC, HPLC (chiralcel IB, hexane/*i*-PrOH = 80:20, flow rate 1.0 mL/min, λ = 254 nm) t_r(major) = 7.58 min, t_r(minor) = 6.18 min, *ee* = 84%, dr > 99:1. $[\alpha]^{14.8}_D$ = 130.5 (c = 0.44, in CH_2Cl_2). ^1H NMR (400 MHz, $CDCl_3$) δ = 8.79 (s, 1H), 7.36 – 7.26 (m, 3H), 7.21 – 7.16 (m, 2H), 7.00 (d, *J* = 1.2 Hz, 1H), 6.94 (d, *J* = 8.2 Hz, 1H), 6.80 (dd, *J* = 8.1, 1.4 Hz, 1H), 5.88 (d, *J* = 8.1 Hz, 1H), 4.09 – 3.99 (m, 1H), 3.97 (d, *J* = 8.5 Hz, 1H), 3.86 (d, *J* = 8.6 Hz, 1H), 1.24 – 1.17 (m, 6H). ^{13}C NMR (101 MHz, $CDCl_3$) δ = 190.6, 174.3, 158.9, 142.3, 132.7, 129.7, 128.7, 128.2, 124.9, 124.6, 122.7, 121.4, 113.4, 42.0, 40.3, 38.4, 37.7, 22.3. HRMS (ESI-TOF) calcd for $C_{21}H_{19}{}^{79}BrN_2O_3$ (M+H$^+$) = 427.0657, found 427.0658; HRMS (ESI-TOF) calcd for $C_{21}H_{19}{}^{81}BrN_2O_3$ (M+H$^+$) = 429.0637, found 429.0638.

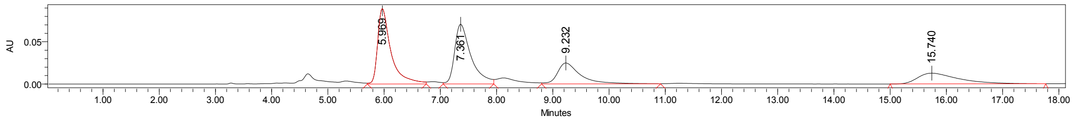

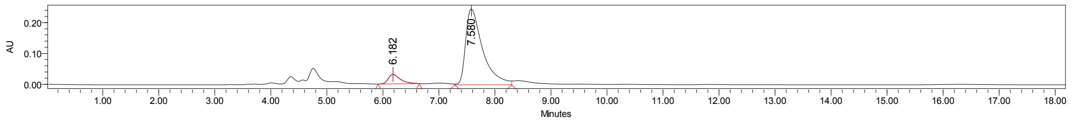

	Retention Time	Area	% Area

1	6.182	457160	8.08
2	7.580	5202782	91.92

(1*R*,2*S*,3*R*)-3'-cyclohexyl-1'-isopropylspiro[indoline-3,2'-piperidine]-2,5',6'-trione:

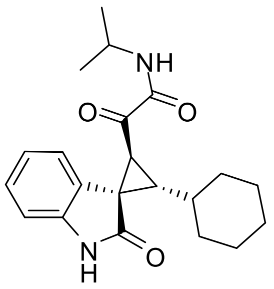

Prepared according to the general procedure. The compound of **4au** was purified by silica gel chromatography (petroleum ether: EA= 1:1) to afford white solid in 60% yield. Mp. 96.0 – 98.0 ºC, HPLC (chiralcel IB, hexane/*i*-PrOH = 90:10, flow rate 1.0 mL/min, λ = 254 nm) t_r(major) = 7.38 min, t_r(minor) = 6.79 min, *ee* = 99%, dr = 72:28. $[\alpha]^{11.7}_D$ = 169.2 (c = 0.48, in CH_2Cl_2). ^1H NMR (400 MHz, $CDCl_3$) δ = 8.69 (s, 1H), 7.21 (t, *J* = 7.6 Hz, 1H), 7.11 (d, *J* = 7.5 Hz, 1H), 7.01 (t, *J* = 7.5 Hz, 1H), 6.93 (d, *J* = 7.7 Hz, 1H), 6.84 (d, *J* = 8.1 Hz, 1H), 3.98 – 3.90 (m, 1H), 3.46 (d, *J* = 8.6 Hz, 1H), 2.51 (dd, *J* = 10.1, 9.0 Hz, 1H), 1.92 – 1.88 (d, *J* = 4.9 Hz, 2H), 1.84 – 1.75 (m, 1H), 1.68 – 1.52 (m, 3H), 1.36 – 1.26 (m, 3H), 1.17 (d, *J* = 6.6 Hz, 3H), 1.13 (d, *J* = 6.6 Hz, 3H), 1.08 – 0.93 (m, 2H). ^{13}C NMR (101 MHz, $CDCl_3$) δ = 190.7, 175.0, 159.1, 141.2, 127.7, 126.6, 122.1, 121.1, 110.2, 41.7, 41.6, 40.0, 39.5, 36.2, 32.5, 32.0, 26.0, 25.9, 25.6, 22.3, 22.3. HRMS (ESI-TOF) calcd for $C_{21}H_{26}N_2O_3$ (M+H⁺) = 355.2022, found 355.2021.

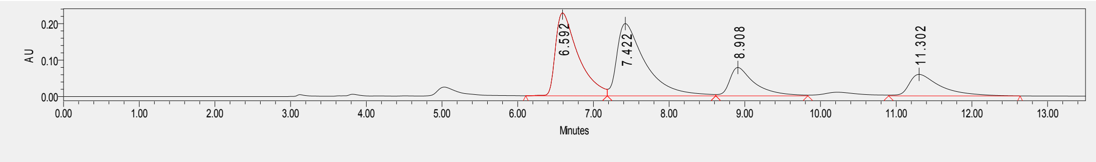

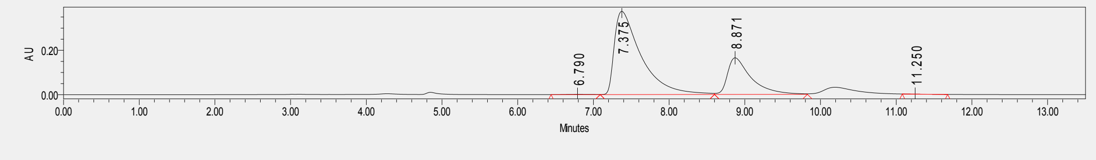

	Retention Time	Area	% Area
1	6.790	24456	0.19
2	7.375	9267899	71.90
3	8.871	3591929	27.87
4	11.250	5775	0.04

(1*R*,2*S*,3*R*)-3'-butyl-1'-isopropylspiro[indoline-3,2'-piperidine]-2,5',6'-trione:

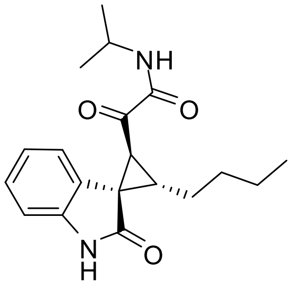

Prepared according to the general procedure. The compound of **4at** was purified by silica gel chromatography (petroleum ether: EA= 1:1) to afford light yellow oil in 68% yield. HPLC (chiralcel IE, hexane/*i*-PrOH = 80:20, flow rate 1.0 mL/min, λ = 254 nm) t_r(major) = 9.51 min, t_r(minor) = 19.01 min, *ee* = 99%, dr = 76:24. $[\alpha]^{11.5}_D$ = 143.3 (c = 0.54, in CH_2Cl_2). ^1H NMR (400 MHz, $CDCl_3$) δ = 8.51 (s, 1H), 7.21 (t, *J* = 7.6 Hz, 1H), 7.08 (d, *J* = 7.3 Hz, 1H), 7.02 (t, *J* = 7.5 Hz, 1H), 6.92 (d, *J* = 7.7 Hz, 1H), 6.84 (d, *J* = 8.0 Hz, 1H), 4.01 – 3.89 (m, 1H), 3.43 (d, *J* = 8.6 Hz, 1H), 2.70 – 2.60 (m, 1H), 1.83 -1.79 (m, 1H), 1.78 – 1.65 (m, 1H), 1.35 – 1.24 (m, 4H), 1.17 (d, *J* = 6.5 Hz, 3H), 1.13 (d, *J* = 6.6 Hz, 3H), 0.84 (t, *J* = 7.0 Hz, 3H). ^{13}C NMR (101 MHz, $CDCl_3$) δ = 190.9, 175.0, 159.0, 141.2, 127.7, 126.6, 122.0,

121.5, 110.2, 41.8, 40.1, 40.1, 35.4, 30.9, 26.4, 22.3, 22.3, 22.1, 13.9. HRMS (ESI-TOF) calcd for $C_{19}H_{24}N_2O_3$ (M+H$^+$) = 329.1865, found 329.1861.

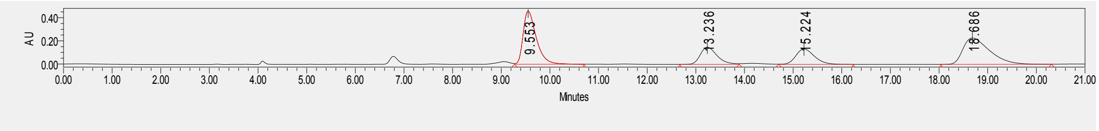

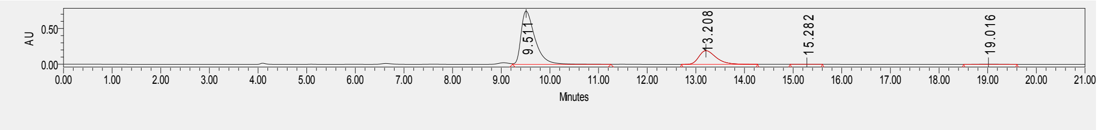

	Retention Time	Area	% Area
1	9.511	14857016	75.56
2	19.011	4700481	23.90
3	15.282	20146	0.10
4	19.016	85714	0.44

(1*R*,2*S*,3*R*) tert-butyl 2-(2-methoxy-2-oxoacetyl)-2'-oxo-3-phenylspiro[cyclopropane-1,3'-indoline]-1'-carboxylate:

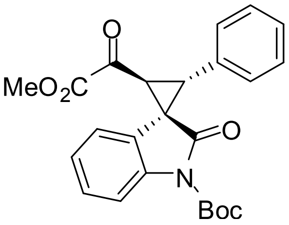

Prepared according to the general procedure. The compound of **5aa** was purified by silica gel chromatography (petroleum ether: EA= 1:1) to afford white solid in 60% yield. Mp. 168.0 – 170.0 ºC, HPLC (chiralcel IE, hexane/*i*-PrOH = 80:20, flow rate 1.0 mL/min, λ = 254 nm) t_r(major) = 11.67 min, t_r(minor) = 16.77 min, *ee* = 93%, dr > 99:1. $[\alpha]^{12.6}_D$ = 176.0 (c = 0.52, in CH_2Cl_2). ^{1}H NMR (400 MHz, $CDCl_3$) δ = 7.88 (d, *J* = 8.2 Hz, 1H), 7.34 – 7.28 (m, 3H), 7.26 – 7.19 (m, 1H), 7.20 – 7.14 (m, 2H), 6.82 (t, *J* = 7.6 Hz, 1H), 6.03 (d, *J* = 7.6 Hz, 1H), 4.10 (d, *J* = 8.6 Hz, 1H), 3.86 (s, 3H), 3.53 (d, *J* = 8.6 Hz, 1H), 1.65 (s, 9H). ^{13}C NMR (101 MHz, $CDCl_3$) δ = 185.8, 171.3, 160.6, 149.0, 140.1, 132.2, 129.8, 128.7, 128.2, 128.2, 123.9, 121.1, 115.0, 84.8, 53.3, 41.0, 40.8, 39.6, 28.1. HRMS (ESI-TOF) calcd for $C_{24}H_{23}NO_6$ (M+H$^+$) = 444.1423, found 444.1418.

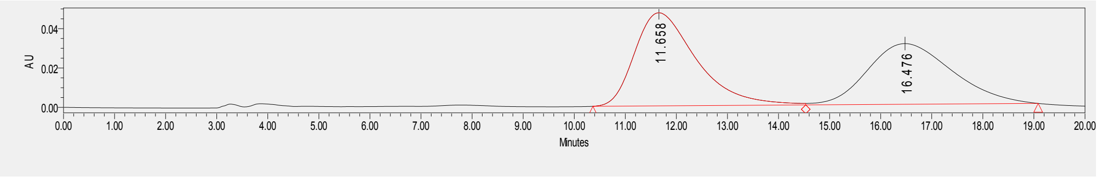

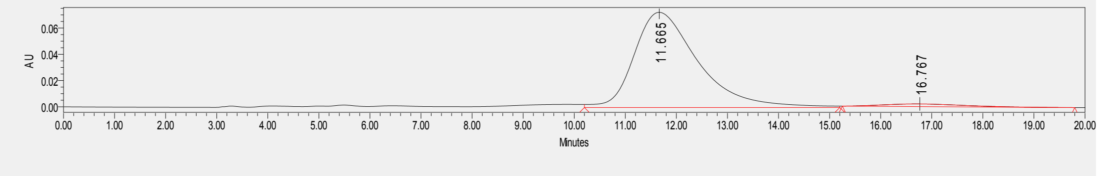

	Retention Time	Area	% Area
1	11.665	6322824	96.69
2	16.767	216148	3.31

Methyl 4-(-3-chloro-2-oxoindolin-3-yl)-2-oxo-4-phenylbutanoate:

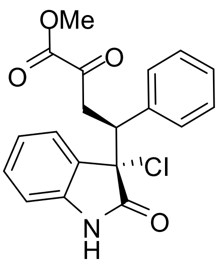

Prepared according to the above procedure. The product was purified by silica gel chromatography (petroleum ether: EA= 2:1) to afford white solid in 80% yield, and then recrystallization. The crude product after isolation, HPLC (chiralcel IB, hexane/*i*-PrOH = 90:10, flow rate 1.0 mL/min, λ = 254 nm) t_r(major) = 10.92 min, t_r(minor) = 15.08 min, *ee* = 35%, dr = 88:12. Mp. 159.0 – 160.0 ºC, $[\alpha]^{28.4}_D$ = 38.8 (c = 0.36, in CH_2Cl_2). And then the mixture was further purified to give a pure diastereomer. ^1H NMR (400 MHz, $CDCl_3$) δ = 8.05 (s, 1H), 7.30 (d, *J* = 7.7 Hz, 1H), 7.22 – 7.12 (m, 2H), 7.12 – 7.02 (m, 3H), 6.96 – 6.88 (m, 2H), 6.77 (d, *J* = 7.8 Hz, 1H), 4.12 – 4.02 (m, 2H), 3.83 (s, 3H), 3.73 (dd, *J* = 18.7, 10.6 Hz, 1H). ^{13}C NMR (101 MHz, $CDCl_3$) δ = 191.0, 174.8, 160.9, 140.0, 135.8, 130.6, 129.5, 128.1, 128.1, 128.0, 126.0, 123.1, 110.5, 67.7, 53.2, 47.8, 40.0. HRMS (ESI-TOF) calcd for $C_{19}H_{16}{}^{35}ClNO_4$ ($M+Na^+$) = 380.0666, found 358.0667; HRMS (ESI-TOF) calcd for $C_{19}H_{16}{}^{37}ClNO_4$ ($M+Na^+$) = 382.0636, found 382.0618.

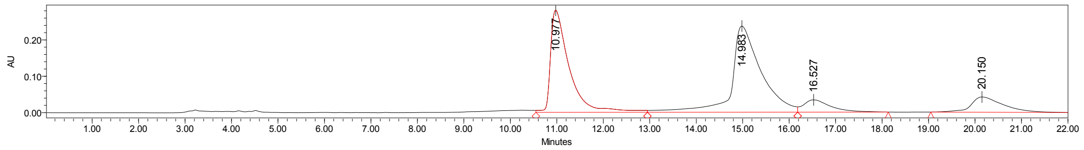

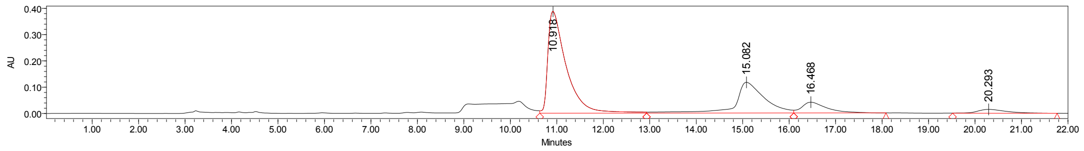

	Retention Time	Area	% Area
1	10.918	10661340	59.45
2	15.082	5103528	28.46
3	16.468	1543662	8.61
4	20.293	625570	3.49

Methyl 4-(1-methyl-3-chloro-2-oxoindolin-3-yl)-2-oxo-4-phenylbutanoate:

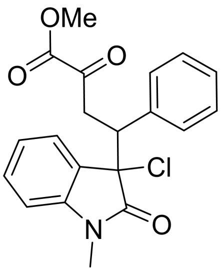

Prepared according to the above procedure of synthesis of the Michale intermediate. The product **7** was purified by silica gel chromatography (petroleum ether: EA= 2:1) to afford white solid in 21% yield. HPLC (chiralcel IE, hexane/*i*-PrOH = 70:30, flow rate 1.0 mL/min, λ = 254 nm) t_r(major) = 19.82 min, t_r(minor) = 21.54 min, *ee* = 77%. $[\alpha]^{22.6}_D$ = 32.4 (c = 0.03, in CH_2Cl_2). ^1H NMR (400 MHz, $CDCl_3$) δ = 7.36 – 7.29 (m, 1H), 7.21 – 7.11 (m, 2H), 7.11 – 7.04 (m, 3H), 6.92 – 6.84 (m, 2H), 6.65 (d, *J* = 7.8 Hz, 1H), 4.16 – 4.02 (m, 2H), 3.85 (s, 3H), 3.71 (dd, *J* = 19.2, 11.2 Hz, 1H), 2.88 (s, 3H). ^{13}C NMR (101 MHz, $CDCl_3$) δ = 191.0, 173.0, 161.0, 142.9, 135.9, 130.5, 129.3, 128.0, 127.9, 127.6, 125.7, 123.0, 108.5, 67.6, 53.1, 48.0, 39.87, 26.26. HRMS (ESI-TOF) calcd for $C_{20}H_{18}{}^{35}ClNO_4$ ($M+H^+$) = 372.0997, found 372.0996; HRMS (ESI-TOF) calcd for $C_{20}H_{18}{}^{37}ClNO_4$ ($M+H^+$) = 374.0968, found 374.0964.

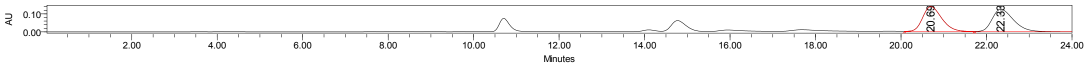

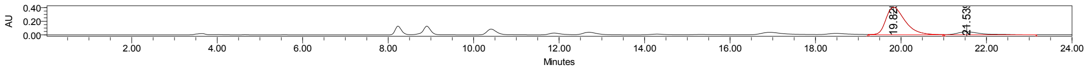

	Retention Time	Area	% Area
1	19.825	12684011	88.25
2	21.539	1688716	11.75

methyl 2-(-1'-methyl-2'-oxo-2-phenylspiro[cyclopropane-1,3'-indolin]-3-yl)-2-oxoacetate:

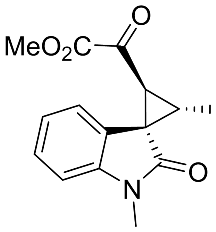

Prepared according to the procedure of the Synthesis of *rel*-(1*S*,2*S*,3*R*)-**3'**. The product **8aa'** was purified by silica gel chromatography (petroleum ether: EA= 1:1) to afford white solid in 40% yield. HPLC (chiralcel IE, hexane/*i*-PrOH = 70:30, flow rate 1.0 mL/min, λ = 254 nm) t_r(major) = 14.52 min, t_r(minor) = 10.52 min, *ee* = -4%. $[\alpha]^{21.8}_D$ = 8.2 (c = 0.24, in CH_2Cl_2).^{1}H NMR (400 MHz, $CDCl_3$) δ = 7.40 (d, *J* = 7.6 Hz, 1H), 7.34 – 7.26 (m, 6H), 7.07 (t, *J* = 7.6 Hz, 1H), 6.88 (d, *J* = 7.6 Hz, 1H), 4.39 (d, *J* = 8.4 Hz, 1H), 3.97 (d, *J* = 8.4 Hz, 1H), 3.87 (s, 3H), 3.19 (s, 3H). ^{13}C NMR (101 MHz, $CDCl_3$) δ = 187.4, 170.9, 160.4, 144.0, 132.3, 129.1, 128.2, 128.2, 127.8, 125.0, 122.6, 122.5, 108.1, 53.4, 43.8, 40.8, 40.5, 26.7. HRMS (ESI-TOF) calcd for $C_{20}H_{17}NO_4$ (M+H$^+$) = 336.1230, found 336.1229.

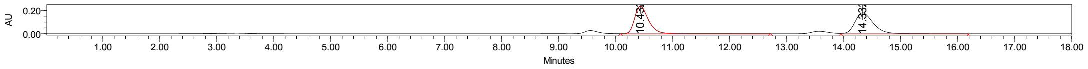

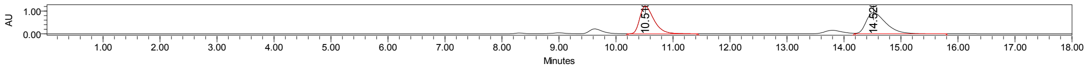

	Retention Time	Area	% Area
1	10.516	20464614	47.97
2	14.520	22195430	52.03

methyl 2-(-1'-methyl-2'-oxo-2-phenylspiro[cyclopropane-1,3'-indolin]-3-yl)-2-oxoacetate:

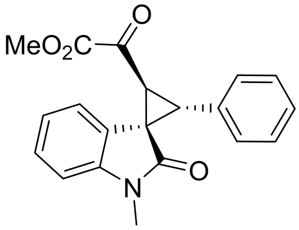

Prepared according to the procedure of the Synthesis of *rel*-(1*S*,2*S*,3*R*)-**3'**. The product **8aa** was purified by silica gel chromatography (petroleum ether: EA= 1:1) to afford white solid in 19% yield. HPLC (chiralcel IE, hexane/*i*-PrOH = 70:30, flow rate 1.0 mL/min, λ = 254 nm) t_r(major) = 20.39 min, t_r(minor) = 18.52 min, *ee* = -41%. $[\alpha]^{21.2}_D$ = -47.9 (c = 0.15, in CH_2Cl_2). ^{1}H NMR (400 MHz, $CDCl_3$) δ = 7.34 – 7.28 (m, 3H), 7.22 – 7.14 (m, 3H), 6.87 (d, *J* = 7.8 Hz, 1H), 6.74 (t, *J* = 7.6 Hz, 1H), 6.05 (d, *J* = 7.6 Hz, 1H), 4.10 (d, *J* = 8.4 Hz, 1H), 3.86 (s, 3H), 3.53 (d, *J* = 8.4 Hz, 1H), 3.28 (s, 3H). ^{13}C NMR (101 MHz, $CDCl_3$) δ = 186.6, 172.7, 160.8,

144.0, 132.9, 129.7, 128.6, 128.0, 127.9, 125.0 122.0, 121.4, 108.3, 53.1, 40.7, 39.8, 37.8, 26.7. HRMS (ESI-TOF) calcd for $C_{20}H_{17}NO_4$ ($M+H^+$) = 336.1230, found 336.1231.

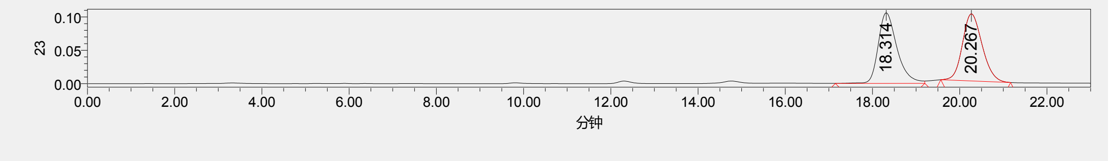

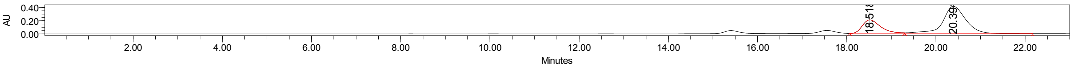

	Retention Time	Area	% Area
1	18.518	6181214	29.20
2	20.391	14990866	70.80

7 Reference

[1] J. Guillaumel, P. Demerseman, J.-M. Clavel, R. J. Royer, *Heterocyclic Chem.* **1980**, *17*, 1531.
[2] J. H. Feng, X. Fu, Z. L. Chen, L. L. Lin, X. H. Liu, X. M. Feng, *Org. Lett.* **2013**, *15*, 2640.
[3] Y. H. Wen, X. Huang, J. L. Huang, Y. Xiong, B. Qin, X. M. Feng, *Synlett.* **2005**, *16*, 2445.
[4] a) X. H. Liu, L. L. Lin, X. M. Feng, *Acc. Chem. Res.* **2011**, *44*, 574; b) W. Li, X. H. Liu, X. Y. Hao, Y. F. Cai, L. Lin, X. M. Feng, *Angew. Chem.* **2012**, *124*, 8772; *Angew. Chem. Int. Ed.* **2012**, *51*, 8644; c) Y. L. Liu, D. J. Shang, X. Zhou, X. H. Liu, X. M. Feng, *Chem. Eur. J.* **2009**, *15*, 2055.

8 Copies of the CD Spectra

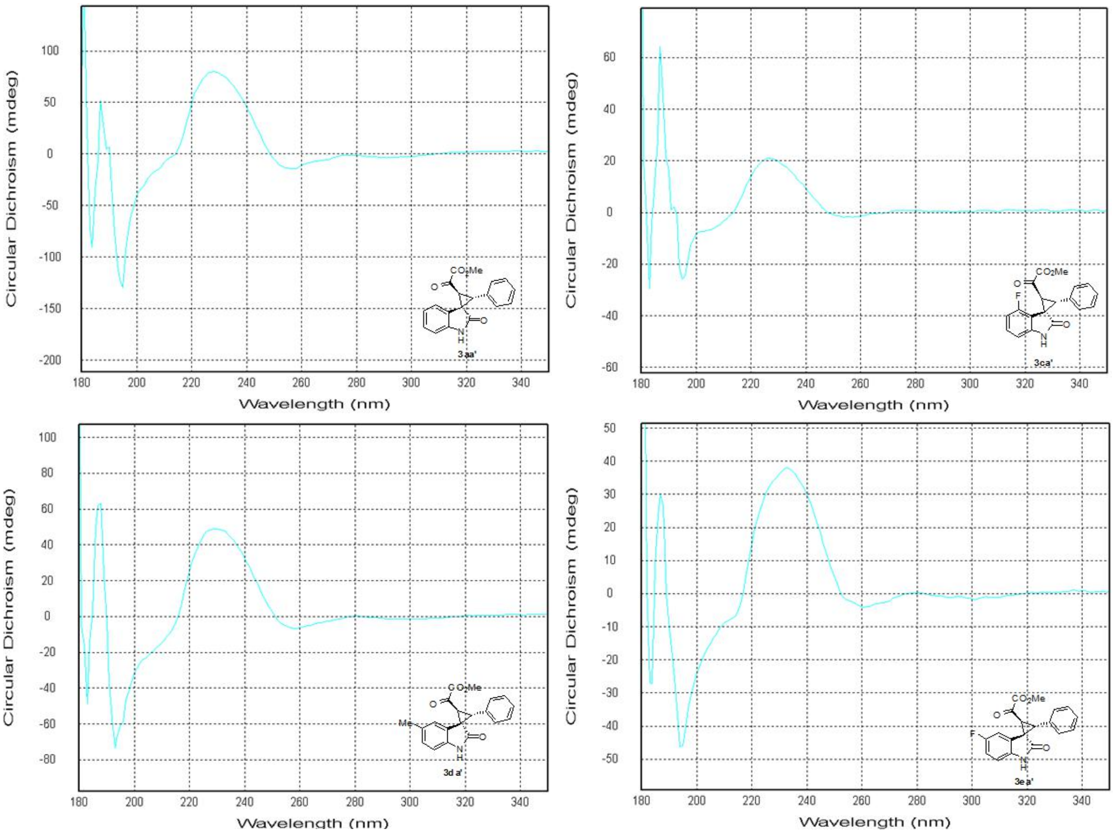

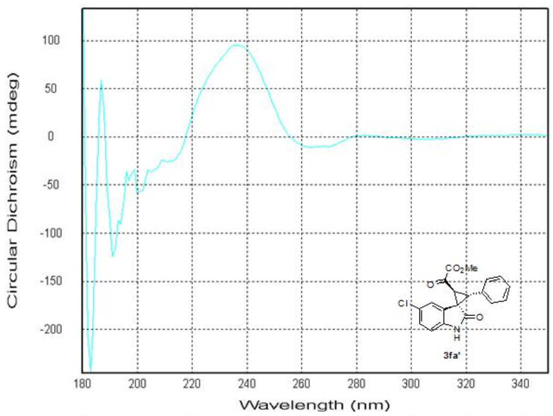
Circular Dichroism (mdeg)
Wavelength (nm)
3fa'

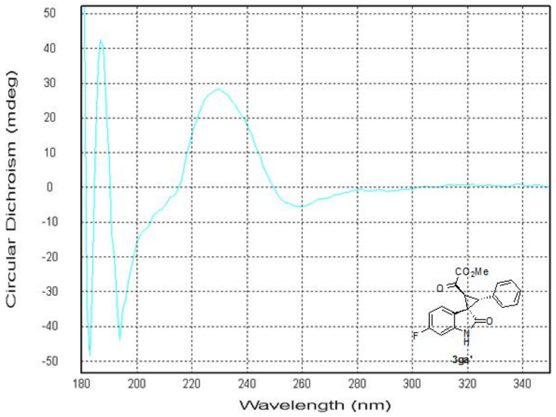
Circular Dichroism (mdeg)
Wavelength (nm)
3ga'

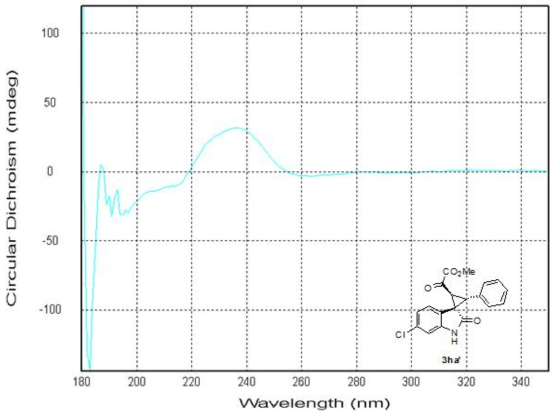
Circular Dichroism (mdeg)
Wavelength (nm)
3ha'

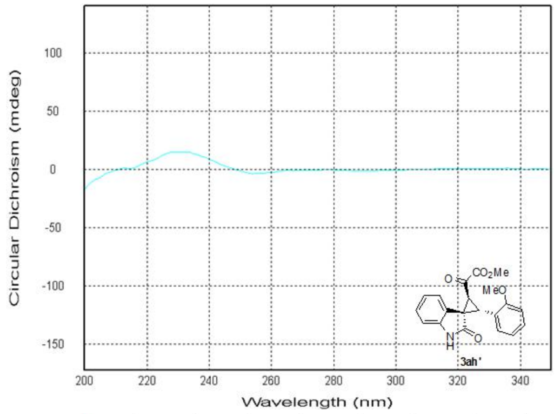
Circular Dichroism (mdeg)
Wavelength (nm)
3ah'

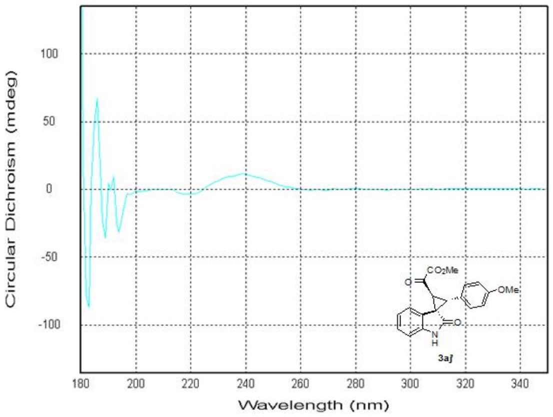
Circular Dichroism (mdeg)
Wavelength (nm)
3aj'

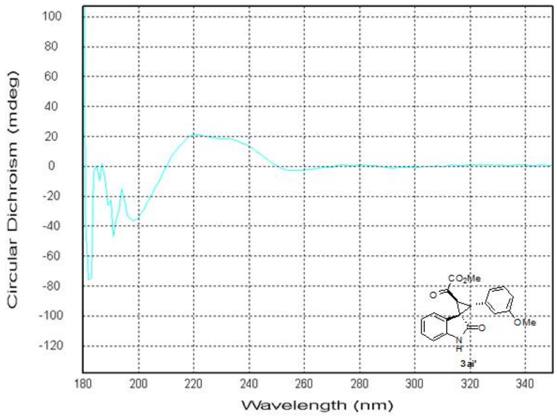
Circular Dichroism (mdeg)
Wavelength (nm)
3ai'

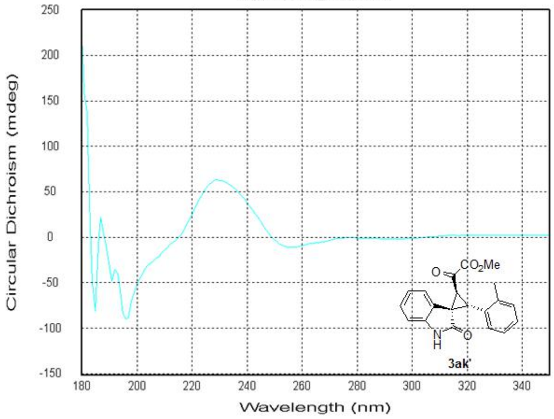
Circular Dichroism (mdeg)
Wavelength (nm)
3ak'

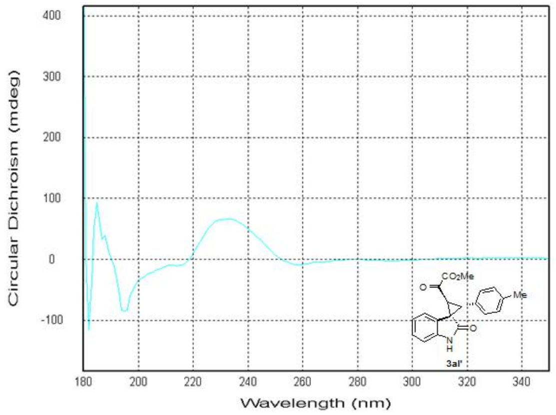
Circular Dichroism (mdeg)
Wavelength (nm)
3al'

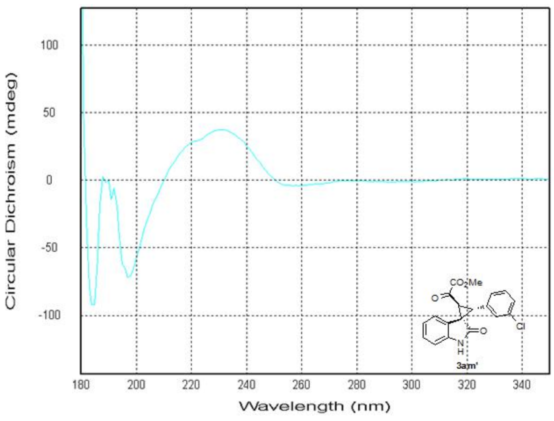
Circular Dichroism (mdeg)
Wavelength (nm)
CO2Me
Cl
3am'
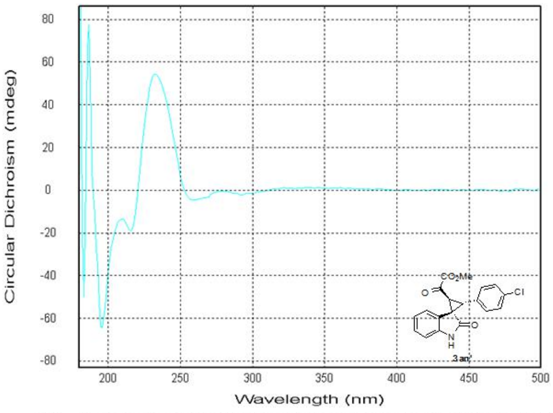
Circular Dichroism (mdeg)
Wavelength (nm)
CO2Me
Cl
3an'
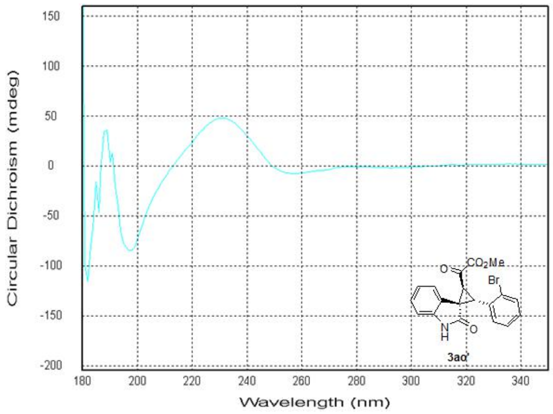
Circular Dichroism (mdeg)
Wavelength (nm)
CO2Me
Br
3ao'
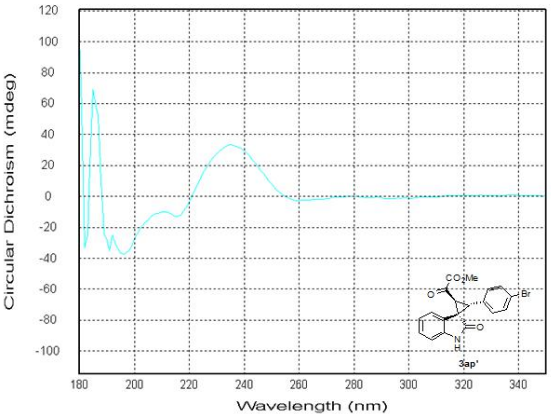
Circular Dichroism (mdeg)
Wavelength (nm)
CO2Me
Br
3ap'
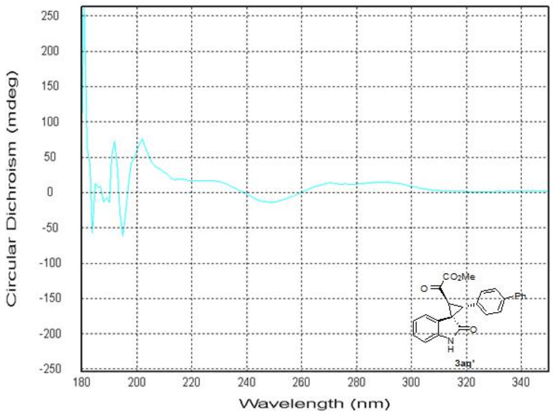
Circular Dichroism (mdeg)
Wavelength (nm)
CO2Me
Ph
3aq'
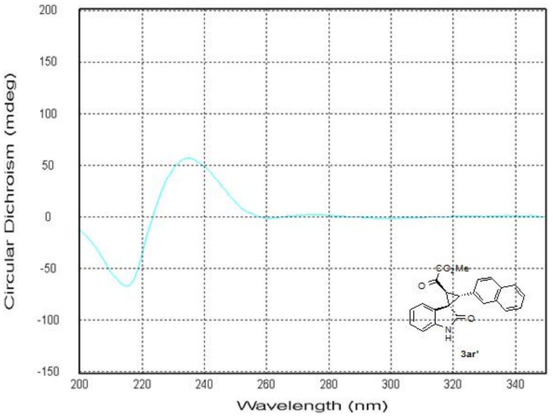
Circular Dichroism (mdeg)
Wavelength (nm)
CO2Me
3ar'
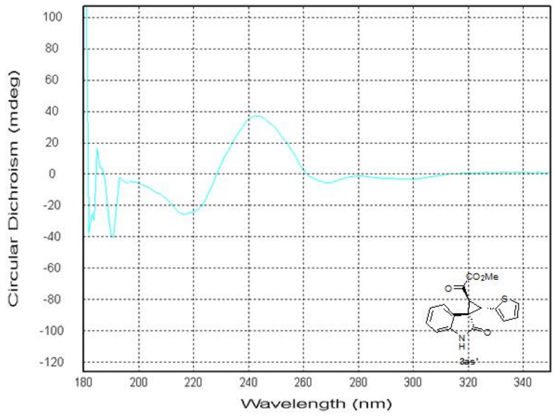
Circular Dichroism (mdeg)
Wavelength (nm)
CO2Me
3as'
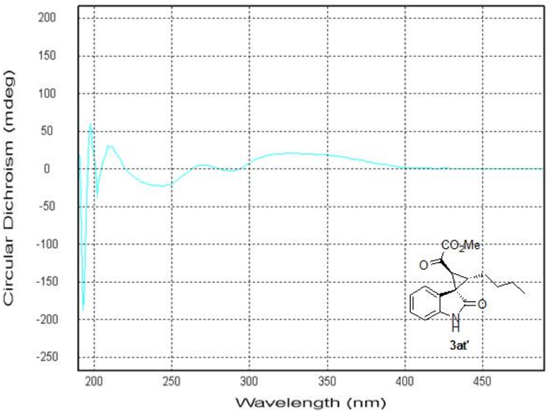
Circular Dichroism (mdeg)
Wavelength (nm)
CO2Me
3at'

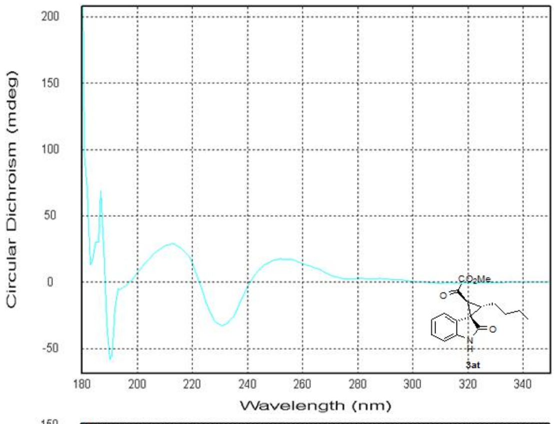
Circular Dichroism (mdeg)
Wavelength (nm)
3at

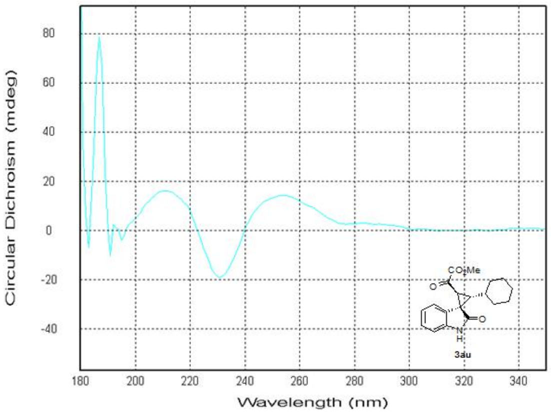
Circular Dichroism (mdeg)
Wavelength (nm)
3au

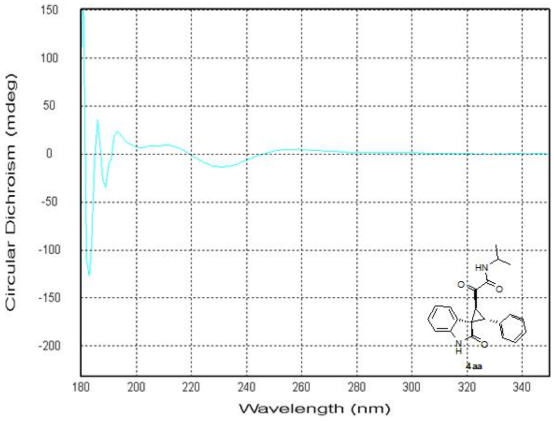
Circular Dichroism (mdeg)
Wavelength (nm)
4aa

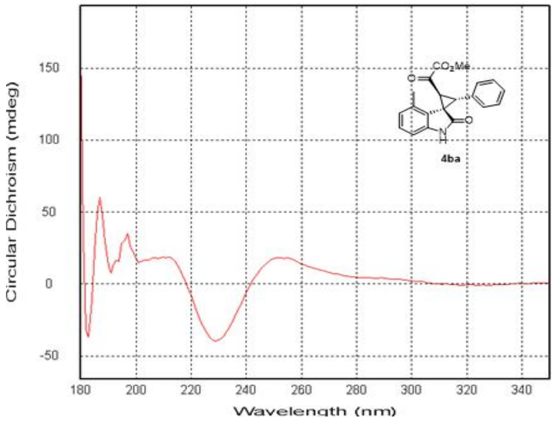
Circular Dichroism (mdeg)
Wavelength (nm)
4ba

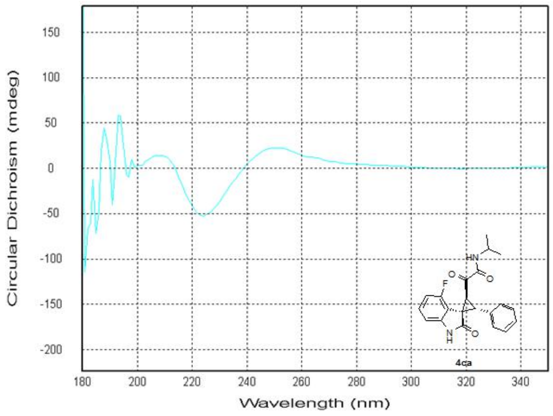
Circular Dichroism (mdeg)
Wavelength (nm)
4ca

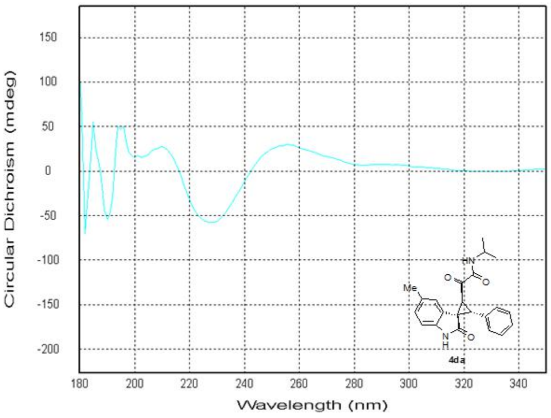
Circular Dichroism (mdeg)
Wavelength (nm)
4da

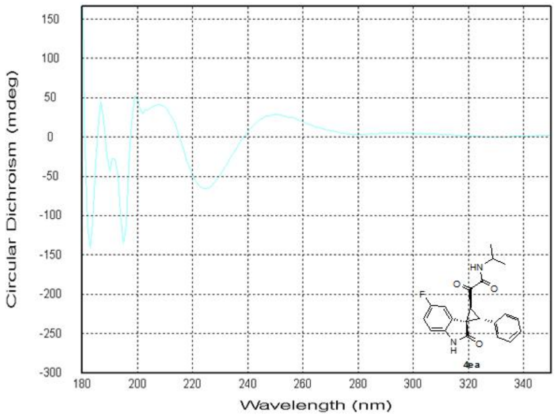
Circular Dichroism (mdeg)
Wavelength (nm)
4ea

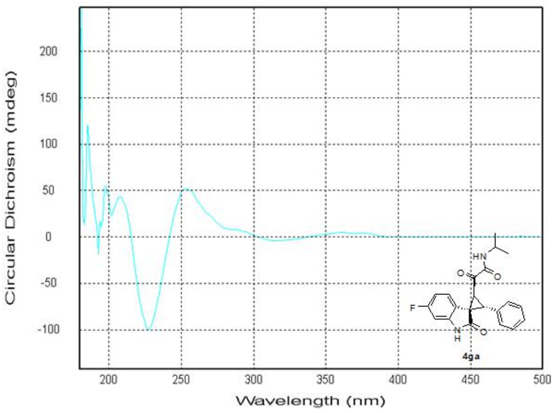
Circular Dichroism (mdeg)
Wavelength (nm)
4ga

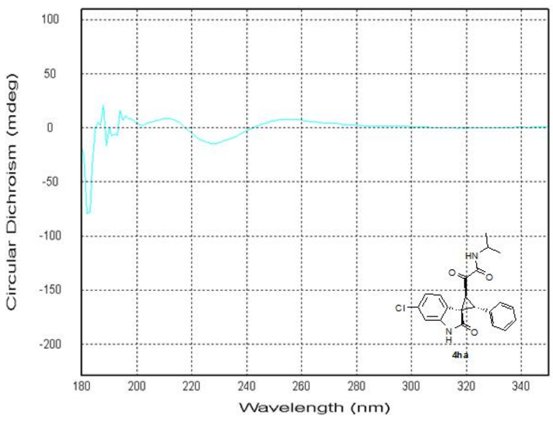
Circular Dichroism (mdeg)
Wavelength (nm)
4ha

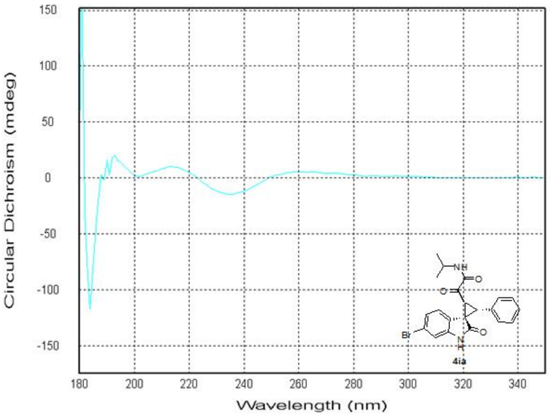
Circular Dichroism (mdeg)
Wavelength (nm)
4ia

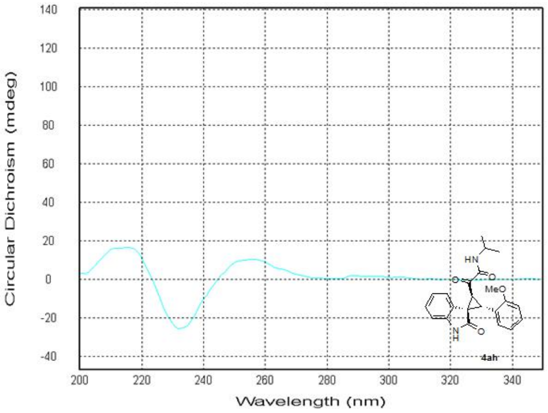
Circular Dichroism (mdeg)
Wavelength (nm)
4ah

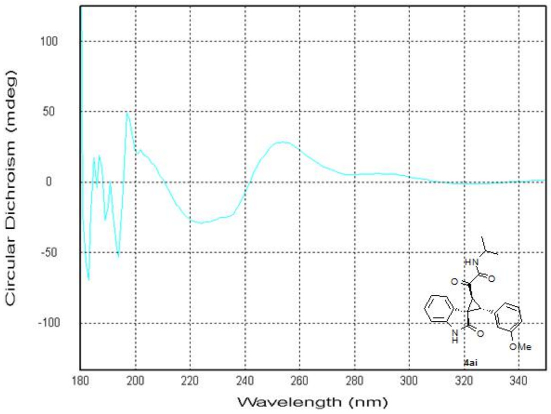
Circular Dichroism (mdeg)
Wavelength (nm)
4ai

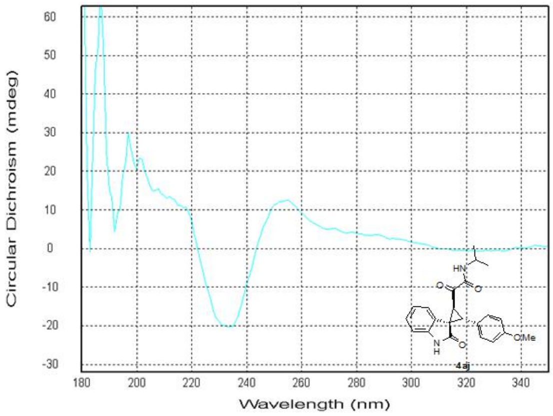
Circular Dichroism (mdeg)
Wavelength (nm)
4aj

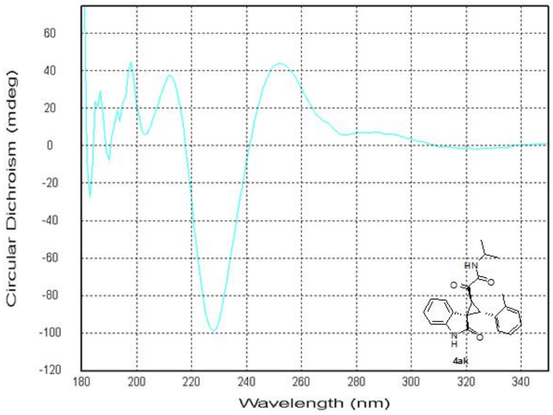
Circular Dichroism (mdeg)
Wavelength (nm)
4ak

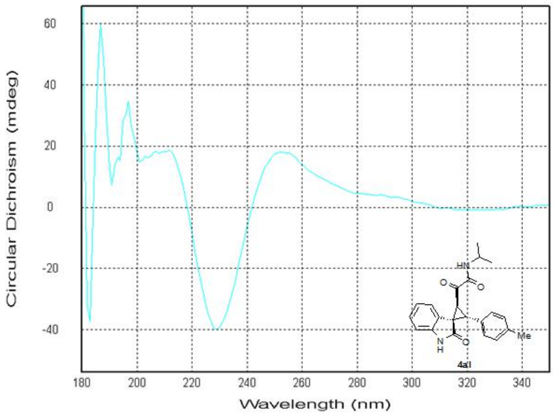
Circular Dichroism (mdeg)
Wavelength (nm)
4al

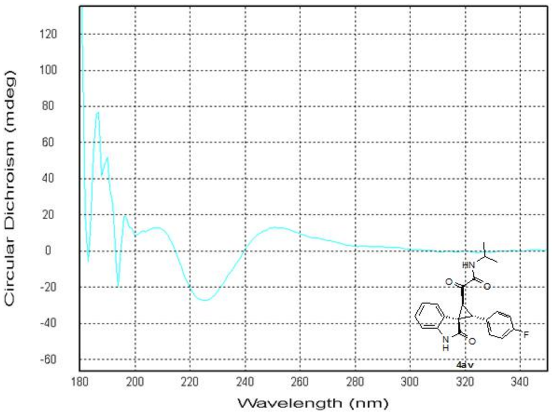
Circular Dichroism (mdeg)
Wavelength (nm)
4av

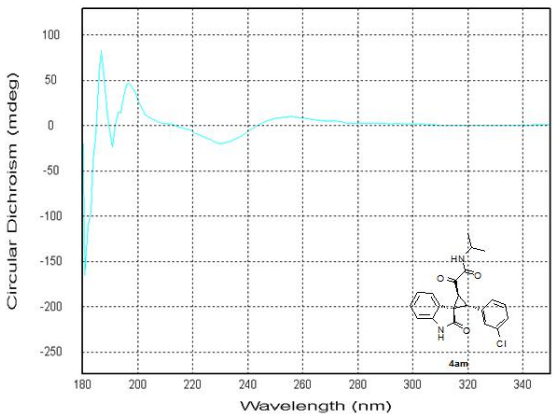
Circular Dichroism (mdeg)
Wavelength (nm)
4am

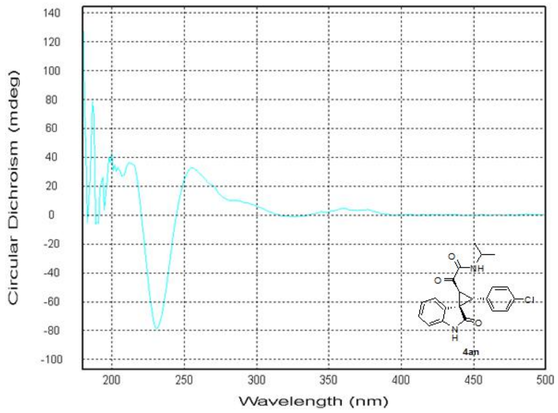
Circular Dichroism (mdeg)
Wavelength (nm)
4an

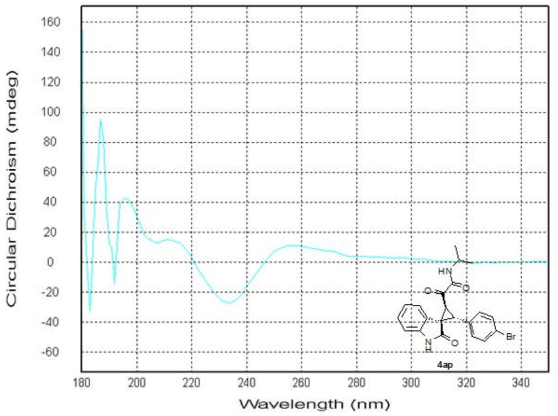
Circular Dichroism (mdeg)
Wavelength (nm)
4ap

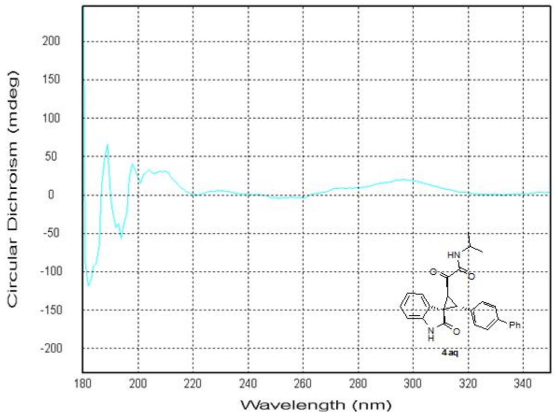
Circular Dichroism (mdeg)
Wavelength (nm)
4aq

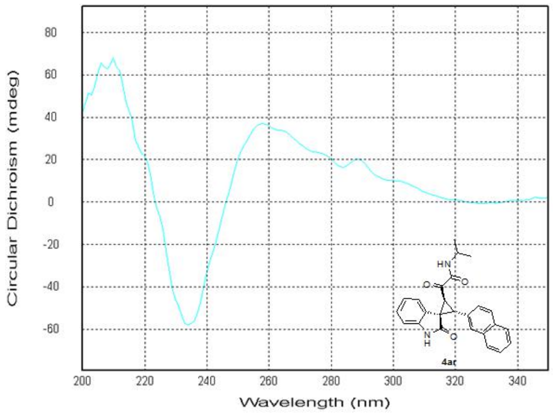
Circular Dichroism (mdeg)
Wavelength (nm)
4ar

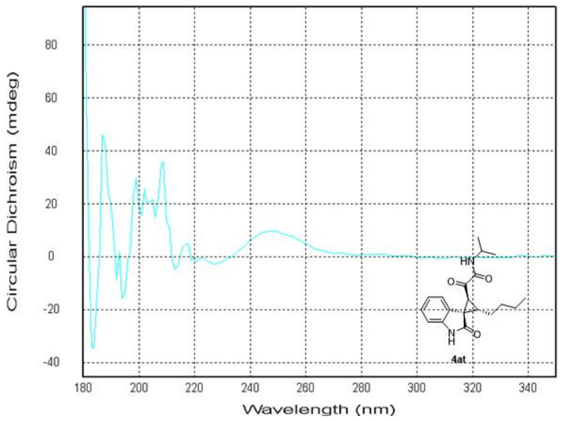
Circular Dichroism (mdeg)
Wavelength (nm)
4at

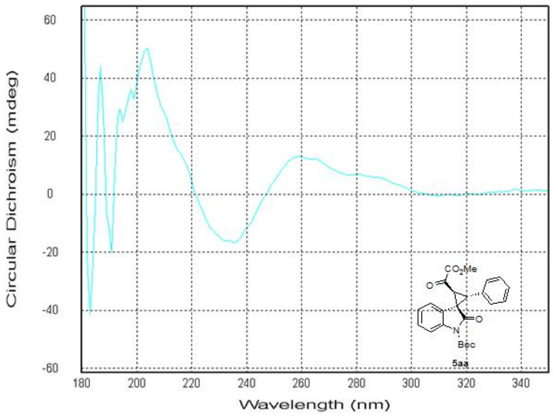
Circular Dichroism (mdeg)
Wavelength (nm)
5aa

9 Copies of the NMR Spectra

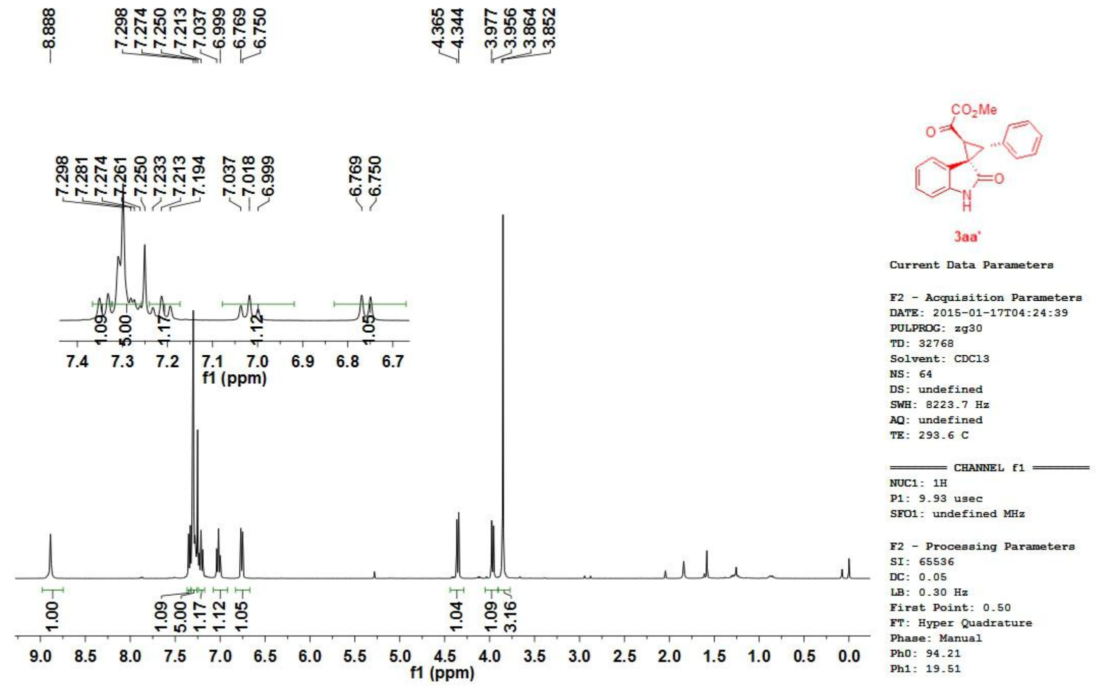

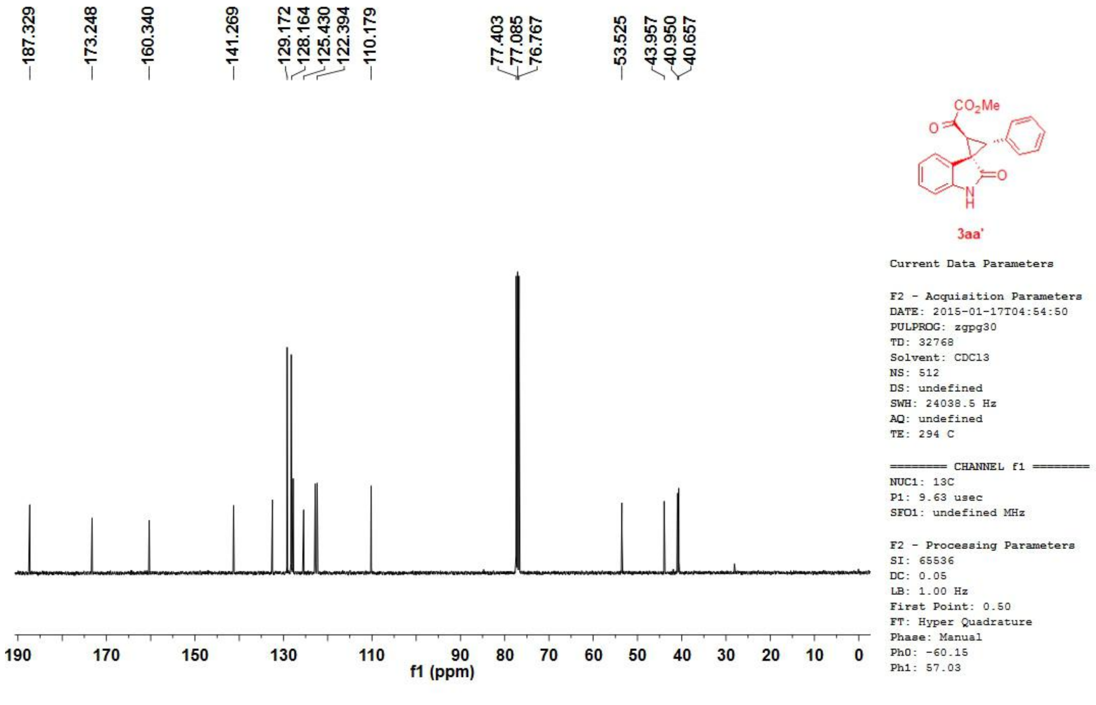

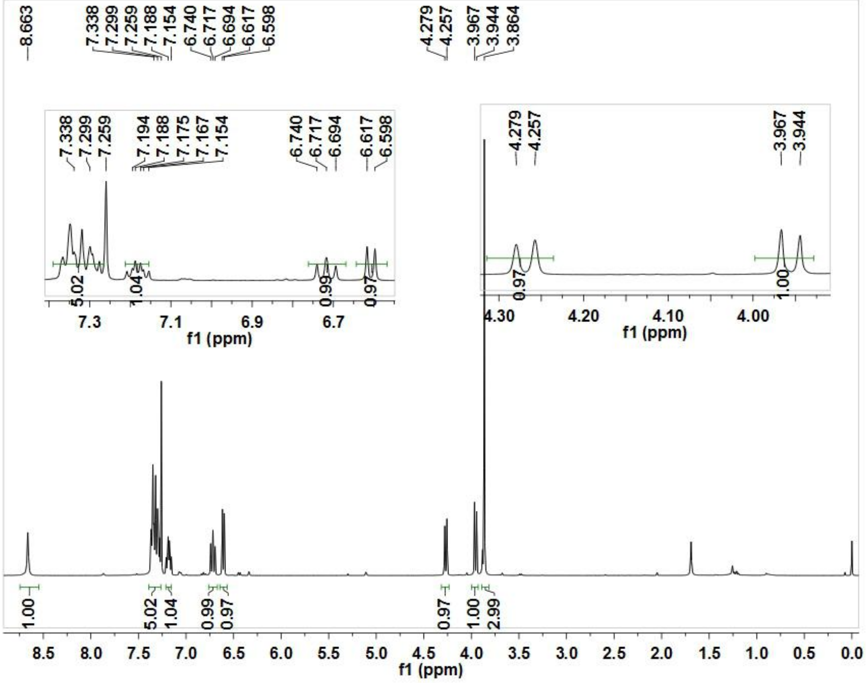

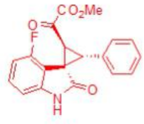

3ca'

Current Data Parameters

F2 - Acquisition Parameters
DATE: 2015-02-25T21:45:15
PULPROG: zg30
TD: 32768
Solvent: CDCl3
NS: 128
DS: undefined
SWH: 8223.7 Hz
AQ: undefined
TE: 293.6 C

======== CHANNEL f1 ========
NUC1: 1H
P1: 9.93 usec
SFO1: undefined MHz

F2 - Processing Parameters
SI: 65536
DC: 0.05
LB: 0.30 Hz
First Point: 0.50
FT: Hyper Quadrature
Phase: Manual
Ph0: 97.34
Ph1: 14.52

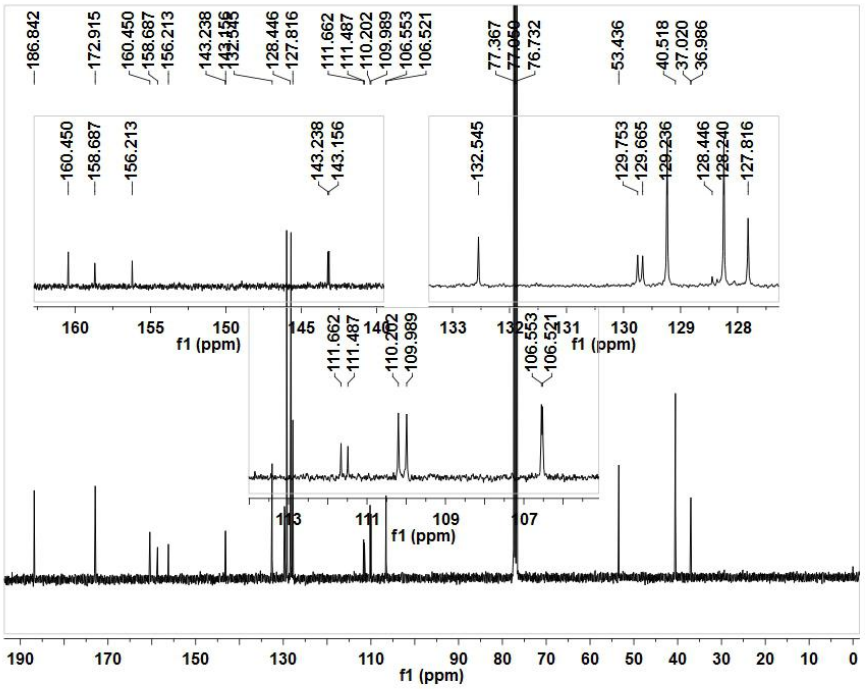

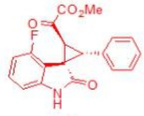

3ca'

Current Data Parameters

F2 - Acquisition Parameters
DATE: 2015-02-25T22:44:41
PULPROG: zgpg30
TD: 32768
Solvent: CDCl3
NS: 1024
DS: undefined
SWH: 24038.5 Hz
AQ: undefined
TE: 294 C

======== CHANNEL f1 ========
NUC1: 13C
P1: 9.63 usec
SFO1: undefined MHz

F2 - Processing Parameters
SI: 65536
DC: 0.05
LB: 1.00 Hz
First Point: 0.50
FT: Hyper Quadrature
Phase: Manual
Ph0: -60.32
Ph1: 52.94

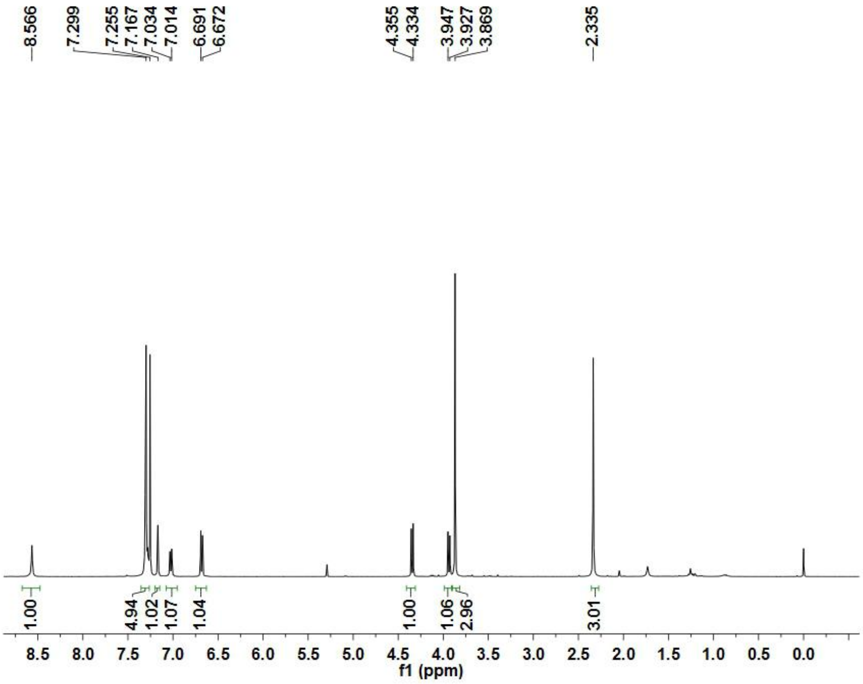

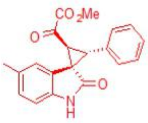

3da'

```
Current Data Parameters

F2 - Acquisition Parameters
DATE: 2014-12-23T20:08:04
PULPROG: zg30
TD: 32768
Solvent: CDCl3
NS: 64
DS: undefined
SWH: 8223.7 Hz
AQ: undefined
TE: 293.8 C

======== CHANNEL f1 ========
NUC1: 1H
P1: 9.93 usec
SFO1: undefined MHz

F2 - Processing Parameters
SI: 65536
DC: 0.05
LB: 0.30 Hz
First Point: 0.50
FT: Hyper Quadrature
Phase: Manual
Ph0: 94.13
Ph1: 17.57
```

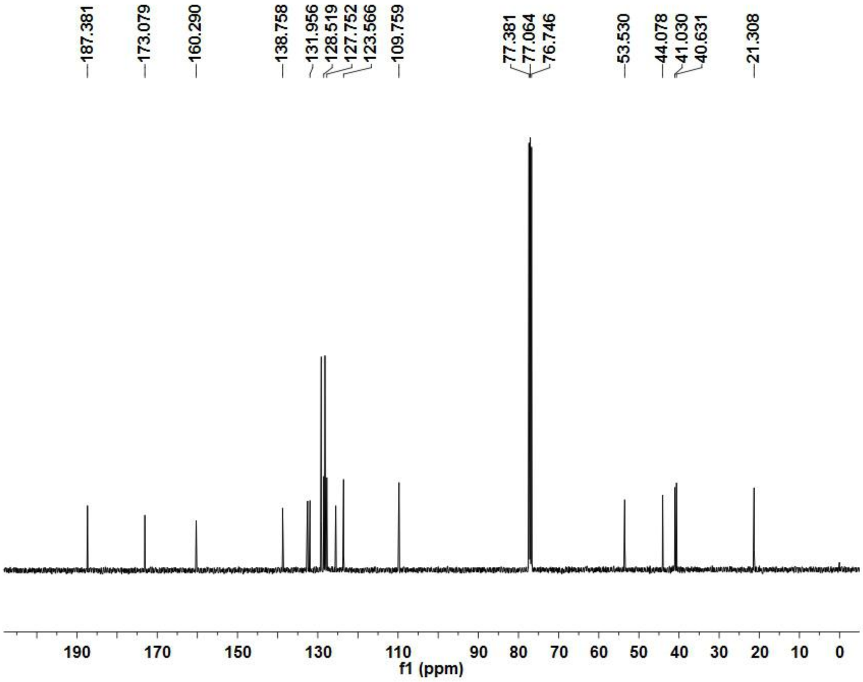

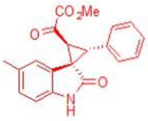

3da'

```
Current Data Parameters

F2 - Acquisition Parameters
DATE: 2014-12-23T20:38:16
PULPROG: zgpg30
TD: 32768
Solvent: CDCl3
NS: 512
DS: undefined
SWH: 24038.5 Hz
AQ: undefined
TE: 294.2 C

======== CHANNEL f1 ========
NUC1: 13C
P1: 9.63 usec
SFO1: undefined MHz

F2 - Processing Parameters
SI: 65536
DC: 0.05
LB: 1.00 Hz
First Point: 0.50
FT: Hyper Quadrature
Phase: Manual
Ph0: -75.29
Ph1: 70.59
```

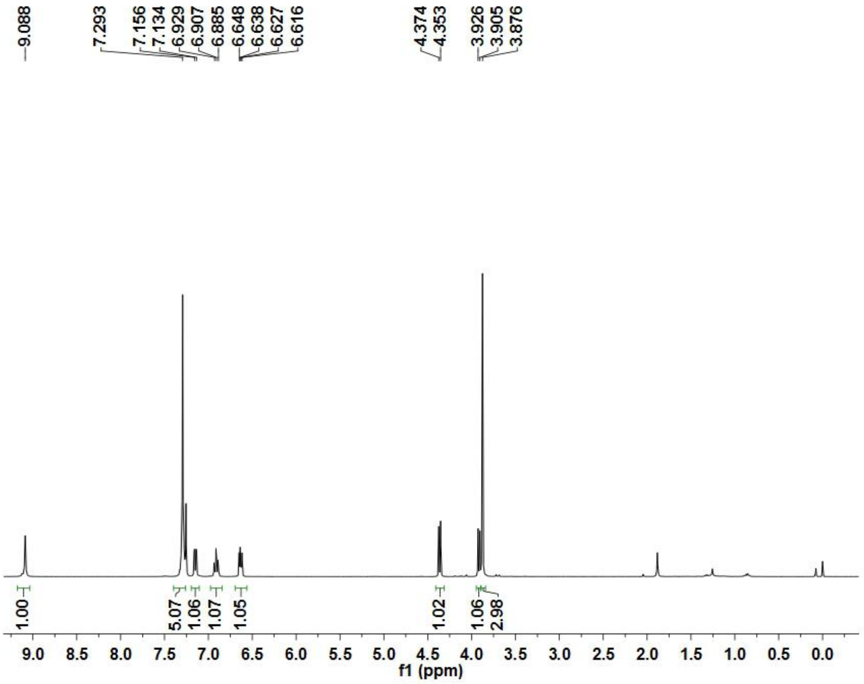

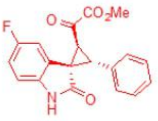

3ea'

Current Data Parameters

F2 - Acquisition Parameters
DATE: 2015-01-16T23:32:09
PULPROG: zg30
TD: 32768
Solvent: CDCl3
NS: 64
DS: undefined
SWH: 8223.7 Hz
AQ: undefined
TE: 293.7 C

======== CHANNEL f1 ========
NUC1: 1H
P1: 9.93 usec
SFO1: undefined MHz

F2 - Processing Parameters
SI: 65536
DC: 0.05
LB: 0.30 Hz
First Point: 0.50
FT: Hyper Quadrature
Phase: Manual
Ph0: 94.88
Ph1: 16.64

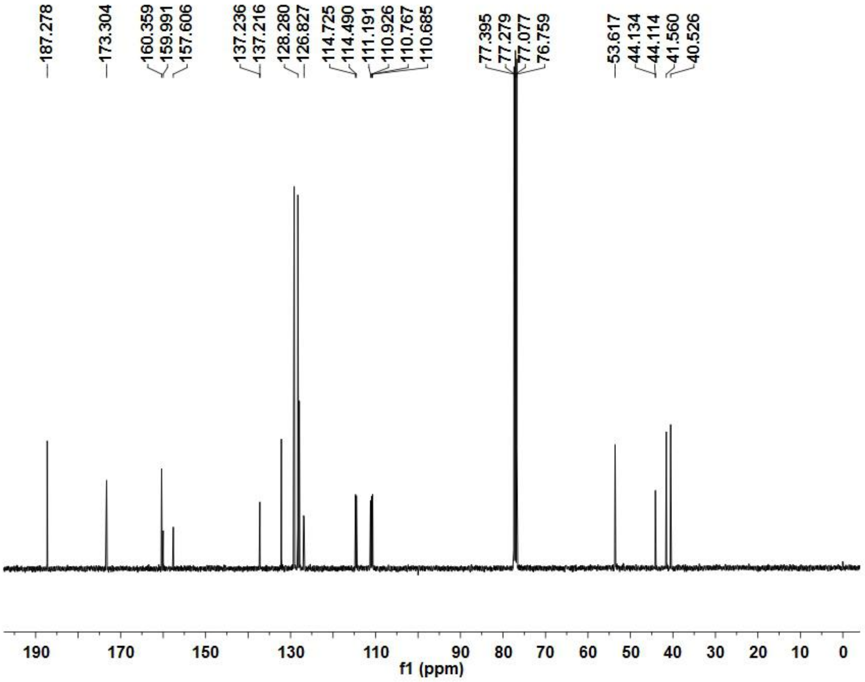

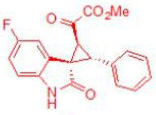

3ea'

Current Data Parameters

F2 - Acquisition Parameters
DATE: 2015-01-17T00:02:20
PULPROG: zgpg30
TD: 32768
Solvent: CDCl3
NS: 512
DS: undefined
SWH: 24038.5 Hz
AQ: undefined
TE: 294 C

======== CHANNEL f1 ========
NUC1: 13C
P1: 9.63 usec
SFO1: undefined MHz

F2 - Processing Parameters
SI: 65536
DC: 0.05
LB: 1.00 Hz
First Point: 0.50
FT: Hyper Quadrature
Phase: Manual
Ph0: -62.29
Ph1: 61.74

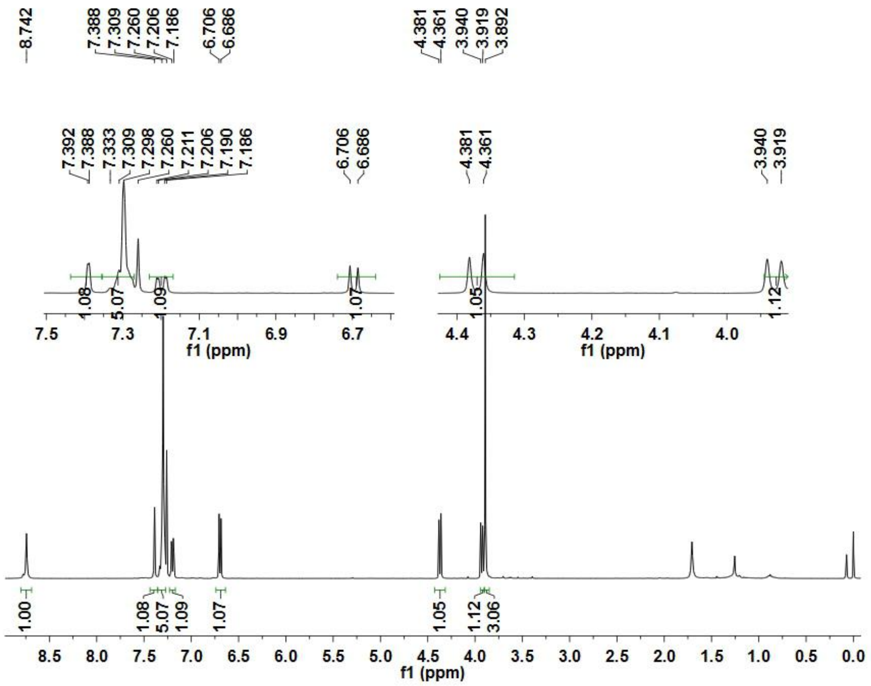

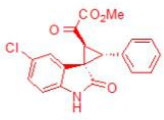

3fa'

Current Data Parameters

F2 - Acquisition Parameters
DATE: 2015-01-16T03:45:17
PULPROG: zg30
TD: 32768
Solvent: CDCl3
NS: 64
DS: undefined
SWH: 8223.7 Hz
AQ: undefined
TE: 294.5 C

======== CHANNEL f1 ========
NUC1: 1H
P1: 9.93 usec
SFO1: undefined MHz

F2 - Processing Parameters
SI: 65536
DC: 0.05
LB: 0.30 Hz
First Point: 0.50
FT: Hyper Quadrature
Phase: Manual
Ph0: -264.73
Ph1: 13.22

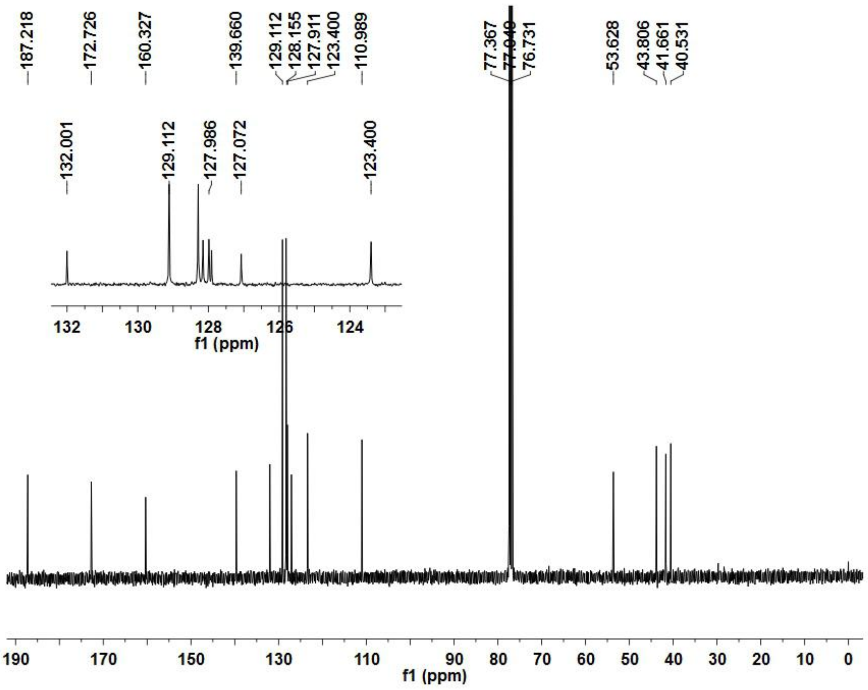

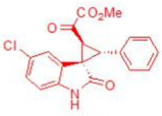

3fa'

Current Data Parameters

F2 - Acquisition Parameters
DATE: 2015-01-16T04:15:29
PULPROG: zgpg30
TD: 32768
Solvent: CDCl3
NS: 512
DS: undefined
SWH: 24038.5 Hz
AQ: undefined
TE: 294.8 C

======== CHANNEL f1 ========
NUC1: 13C
P1: 9.63 usec
SFO1: undefined MHz

F2 - Processing Parameters
SI: 65536
DC: 0.05
LB: 1.00 Hz
First Point: 0.50
FT: Hyper Quadrature
Phase: Manual
Ph0: -57.04
Ph1: 55.25

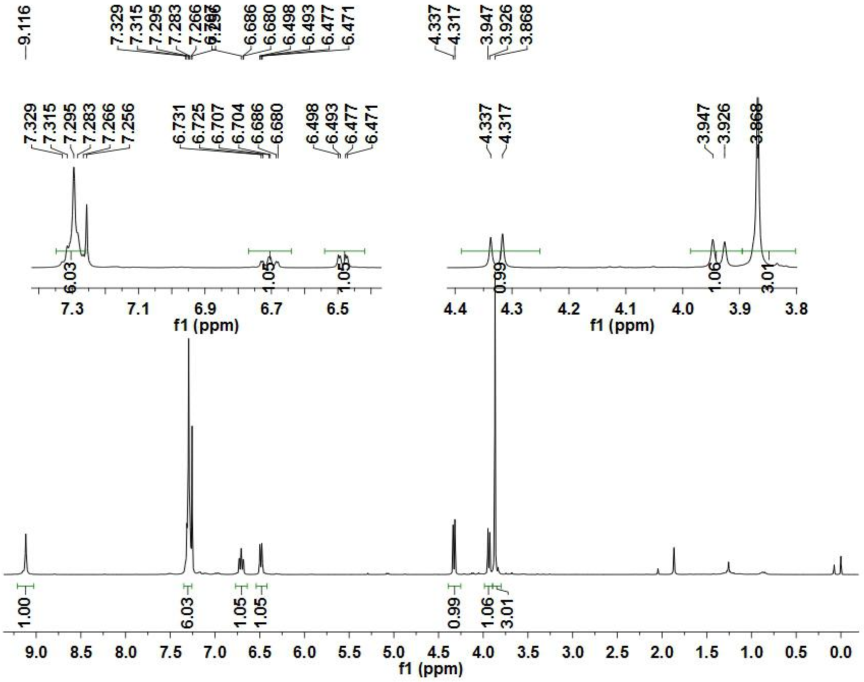

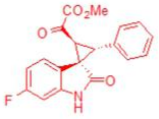

3ga'

Current Data Parameters

F2 - Acquisition Parameters
DATE: 2015-01-17T02:29:14
PULPROG: zg30
TD: 32768
Solvent: CDCl3
NS: 64
DS: undefined
SWH: 8223.7 Hz
AQ: undefined
TE: 293.7 C

======== CHANNEL f1 ========
NUC1: 1H
P1: 9.93 usec
SFO1: undefined MHz

F2 - Processing Parameters
SI: 65536
DC: 0.05
LB: 0.30 Hz
First Point: 0.50
FT: Hyper Quadrature
Phase: Manual
Ph0: 92.44
Ph1: 20.95

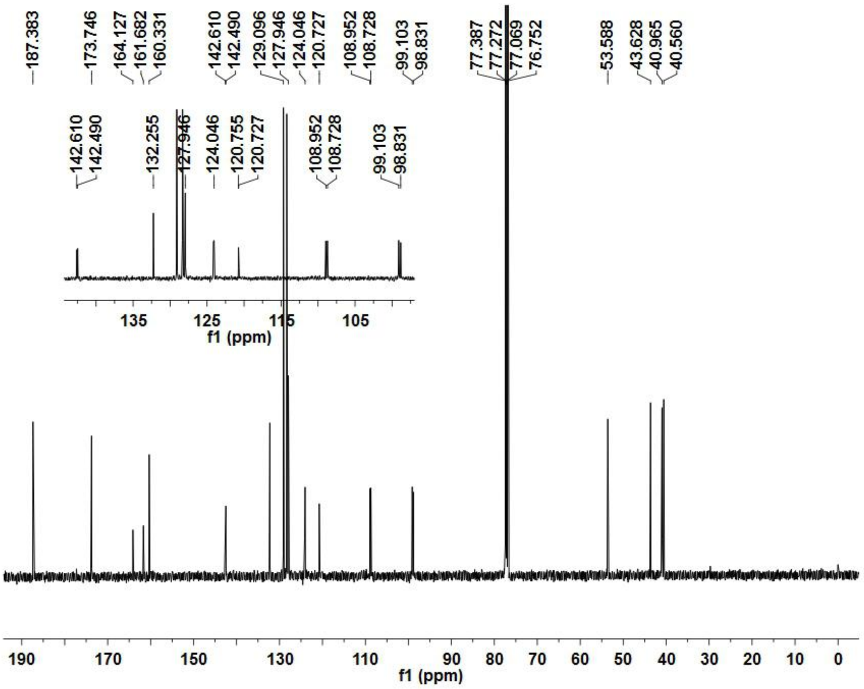

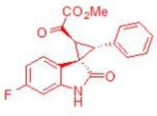

3g a'

Current Data Parameters

F2 - Acquisition Parameters
DATE: 2015-01-17T02:59:26
PULPROG: zgpg30
TD: 32768
Solvent: CDCl3
NS: 512
DS: undefined
SWH: 24038.5 Hz
AQ: undefined
TE: 294 C

======== CHANNEL f1 ========
NUC1: 13C
P1: 9.63 usec
SFO1: undefined MHz

F2 - Processing Parameters
SI: 65536
DC: 0.05
LB: 1.00 Hz
First Point: 0.50
FT: Hyper Quadrature
Phase: Manual
Ph0: -79.51
Ph1: 86.92

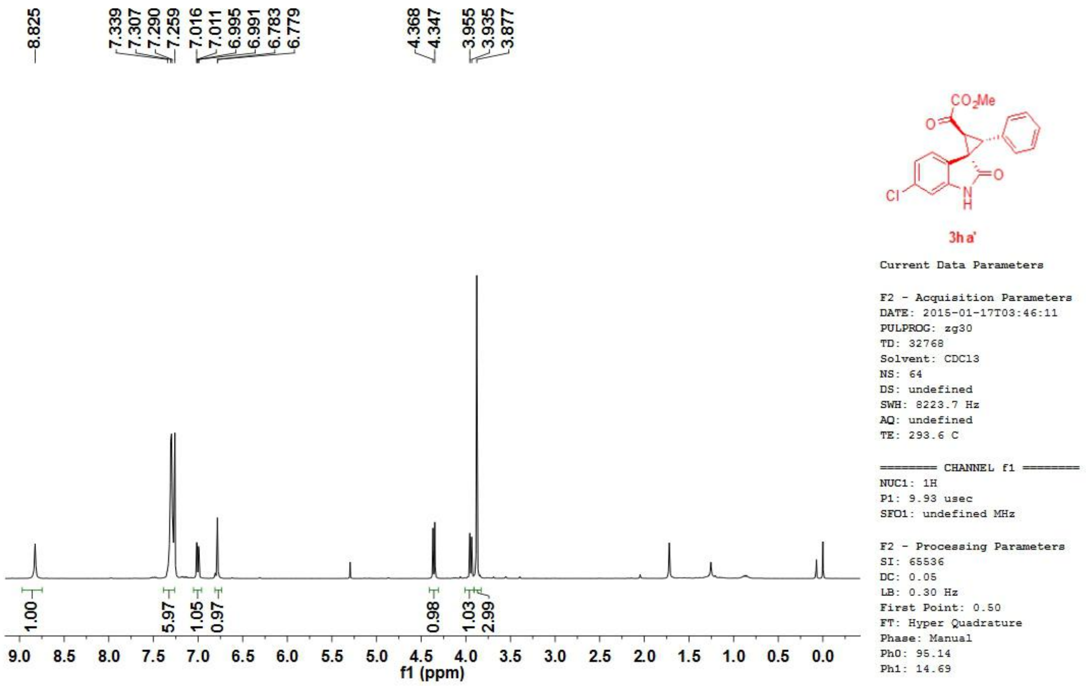
8.825
7.339
7.307
7.290
7.259
7.016
7.011
6.995
6.991
6.783
6.779
4.368
4.347
3.955
3.935
3.877
1.00
5.97
1.05
0.97
0.98
1.03
2.99
9.0 8.5 8.0 7.5 7.0 6.5 6.0 5.5 5.0 4.5 4.0 3.5 3.0 2.5 2.0 1.5 1.0 0.5 0.0
f1 (ppm)
CO_2Me
O
Cl
N
H
3ha'
Current Data Parameters
F2 - Acquisition Parameters
DATE: 2015-01-17T03:46:11
PULPROG: zg30
TD: 32768
Solvent: CDCl3
NS: 64
DS: undefined
SWH: 8223.7 Hz
AQ: undefined
TE: 293.6 C
======== CHANNEL f1 ========
NUC1: 1H
P1: 9.93 usec
SFO1: undefined MHz
F2 - Processing Parameters
SI: 65536
DC: 0.05
LB: 0.30 Hz
First Point: 0.50
FT: Hyper Quadrature
Phase: Manual
Ph0: 95.14
Ph1: 14.69

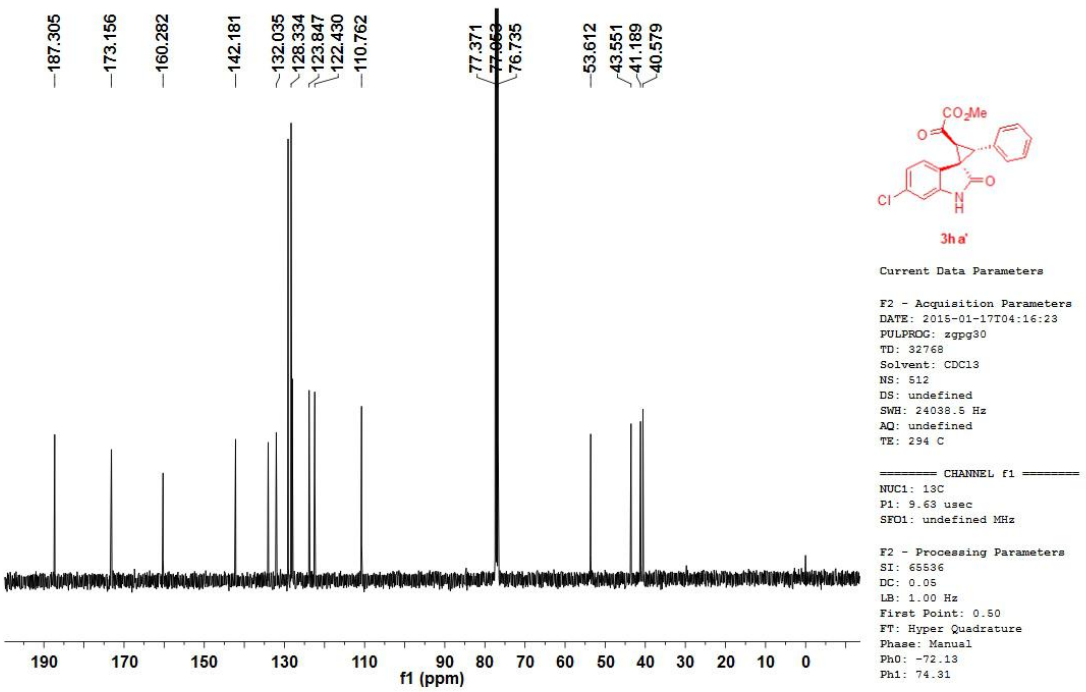
187.305
173.156
160.282
142.181
132.035
128.334
123.847
122.430
110.762
77.371
77.053
76.735
53.612
43.551
41.189
40.579
190 170 150 130 110 90 80 70 60 50 40 30 20 10 0
f1 (ppm)
CO_2Me
O
Cl
N
H
3ha'
Current Data Parameters
F2 - Acquisition Parameters
DATE: 2015-01-17T04:16:23
PULPROG: zgpg30
TD: 32768
Solvent: CDCl3
NS: 512
DS: undefined
SWH: 24038.5 Hz
AQ: undefined
TE: 294 C
======== CHANNEL f1 ========
NUC1: 13C
P1: 9.63 usec
SFO1: undefined MHz
F2 - Processing Parameters
SI: 65536
DC: 0.05
LB: 1.00 Hz
First Point: 0.50
FT: Hyper Quadrature
Phase: Manual
Ph0: -72.13
Ph1: 74.31

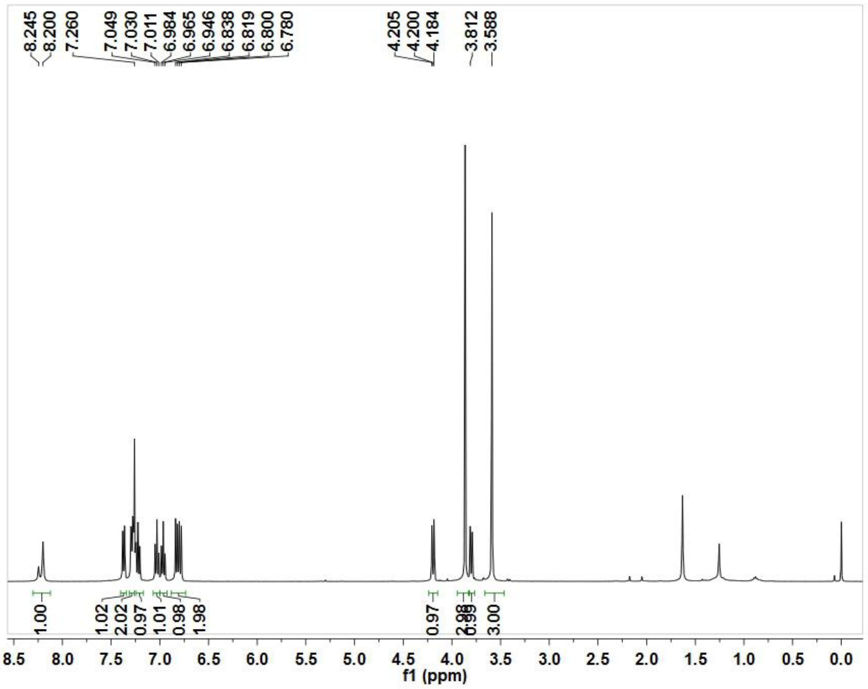

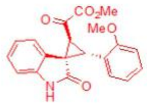

3ah'

Current Data Parameters

F2 - Acquisition Parameters
DATE: 2015-03-03T08:38:05
PULPROG: zg30
TD: 32768
Solvent: CDCl3
NS: 128
DS: undefined
SWH: 8223.7 Hz
AQ: undefined
TE: 293.7 C

======== CHANNEL f1 ========
NUC1: 1H
P1: 9.93 usec
SFO1: undefined MHz

F2 - Processing Parameters
SI: 65536
DC: 0.05
LB: 0.30 Hz
First Point: 0.50
FT: Hyper Quadrature
Phase: Manual
Ph0: 96.54
Ph1: 13.81

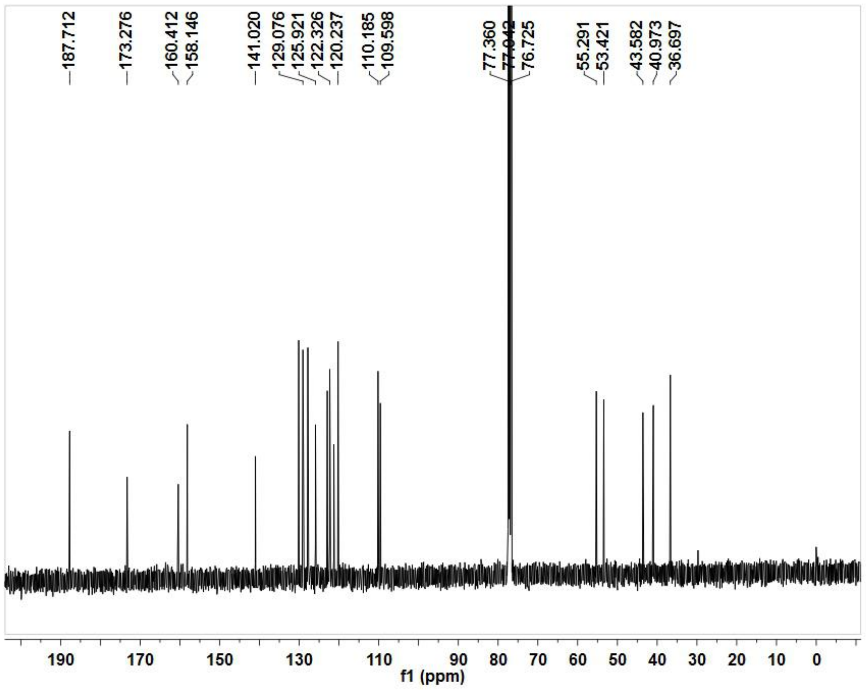

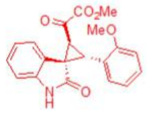

3ah'

Current Data Parameters

F2 - Acquisition Parameters
DATE: 2015-03-03T09:37:31
PULPROG: zgpg30
TD: 32768
Solvent: CDCl3
NS: 1024
DS: undefined
SWH: 24038.5 Hz
AQ: undefined
TE: 294 C

======== CHANNEL f1 ========
NUC1: 13C
P1: 9.63 usec
SFO1: undefined MHz

F2 - Processing Parameters
SI: 65536
DC: 0.05
LB: 1.00 Hz
First Point: 0.50
FT: Hyper Quadrature
Phase: Manual
Ph0: -69.75
Ph1: 65.51

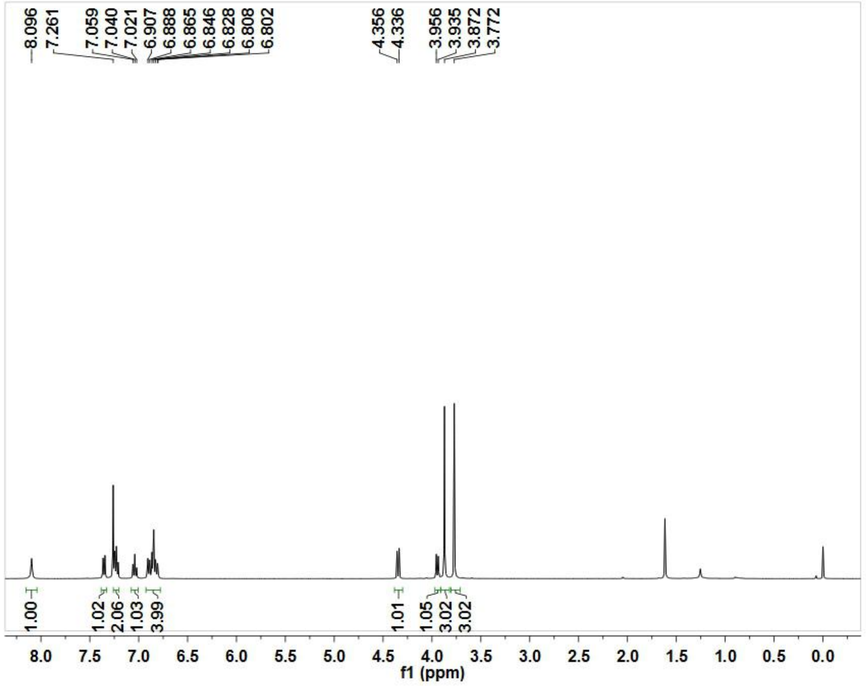

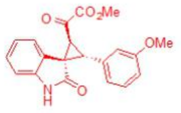

3ai'

Current Data Parameters

F2 - Acquisition Parameters
DATE: 2015-02-25T18:06:21
PULPROG: zg30
TD: 32768
Solvent: CDCl3
NS: 128
DS: undefined
SWH: 8223.7 Hz
AQ: undefined
TE: 293.6 C

======== CHANNEL f1 ========
NUC1: 1H
P1: 9.93 usec
SFO1: undefined MHz

F2 - Processing Parameters
SI: 65536
DC: 0.05
LB: 0.30 Hz
First Point: 0.50
FT: Hyper Quadrature
Phase: Manual
Ph0: 99.83
Ph1: 9.25

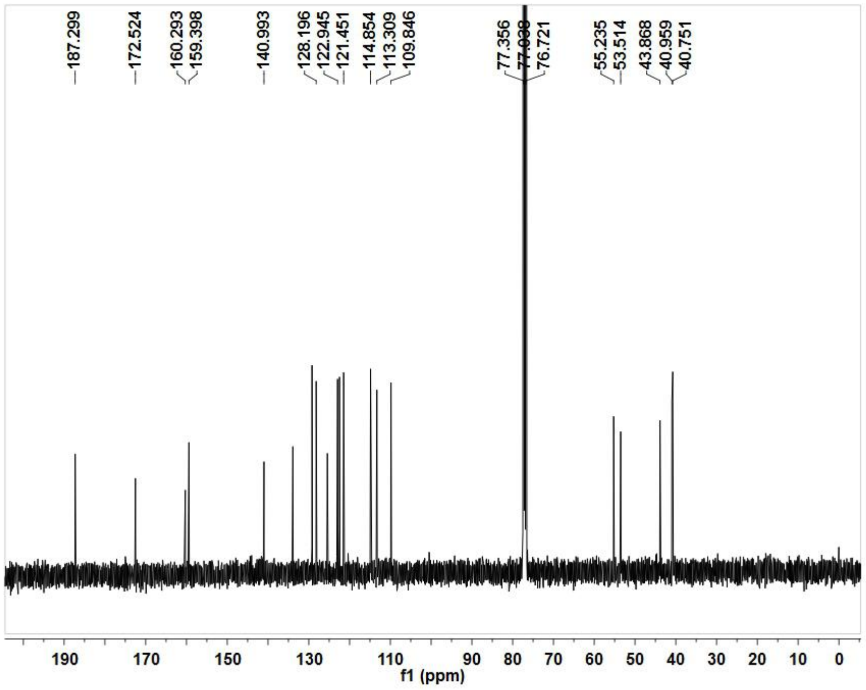

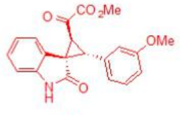

3ai'

Current Data Parameters

F2 - Acquisition Parameters
DATE: 2015-02-25T19:05:46
PULPROG: zgpg30
TD: 32768
Solvent: CDCl3
NS: 1024
DS: undefined
SWH: 24038.5 Hz
AQ: undefined
TE: 294.1 C

======== CHANNEL f1 ========
NUC1: 13C
P1: 9.63 usec
SFO1: undefined MHz

F2 - Processing Parameters
SI: 65536
DC: 0.05
LB: 1.00 Hz
First Point: 0.50
FT: Hyper Quadrature
Phase: Manual
Ph0: -59.00
Ph1: 41.32

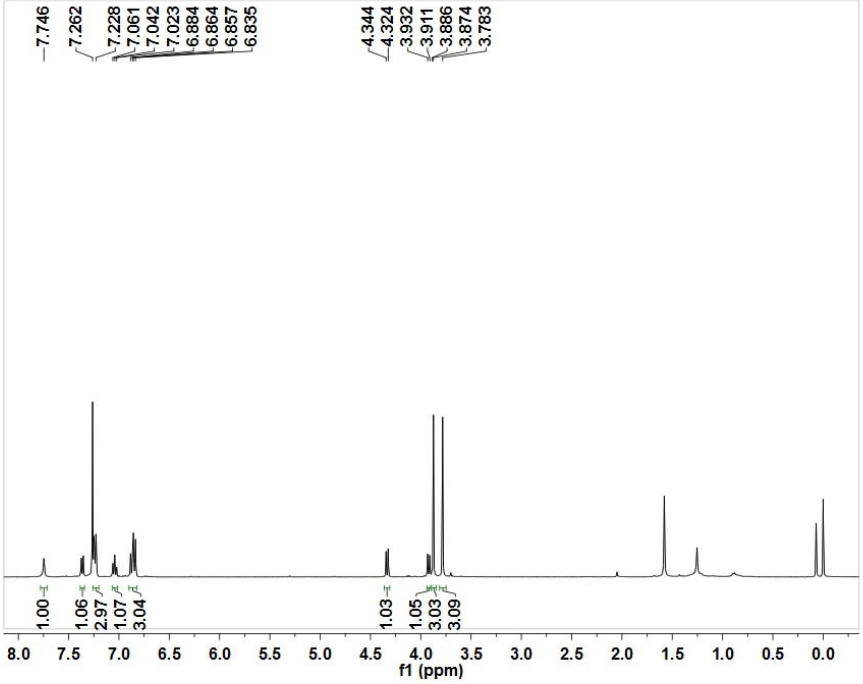

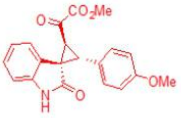

3aj'

Current Data Parameters

F2 - Acquisition Parameters
DATE: 2015-02-25T19:19:10
PULPROG: zg30
TD: 32768
Solvent: CDCl3
NS: 128
DS: undefined
SWH: 8223.7 Hz
AQ: undefined
TE: 293.7 C

======== CHANNEL f1 ========
NUC1: 1H
P1: 9.93 usec
SFO1: undefined MHz

F2 - Processing Parameters
SI: 65536
DC: 0.05
LB: 0.30 Hz
First Point: 0.50
FT: Hyper Quadrature
Phase: Manual
Ph0: 101.09
Ph1: 5.57

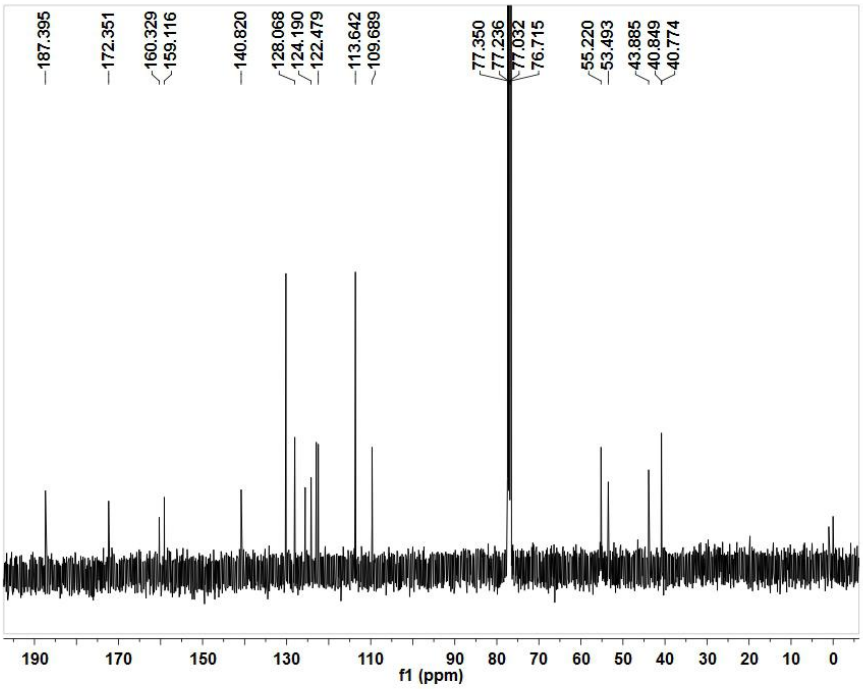

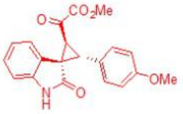

3aj'

Current Data Parameters

F2 - Acquisition Parameters
DATE: 2015-02-25T20:18:36
PULPROG: zgpg30
TD: 32768
Solvent: CDCl3
NS: 1024
DS: undefined
SWH: 24038.5 Hz
AQ: undefined
TE: 294.1 C

======== CHANNEL f1 ========
NUC1: 13C
P1: 9.63 usec
SFO1: undefined MHz

F2 - Processing Parameters
SI: 65536
DC: 0.05
LB: 1.00 Hz
First Point: 0.50
FT: Hyper Quadrature
Phase: Manual
Ph0: -63.99
Ph1: 50.40

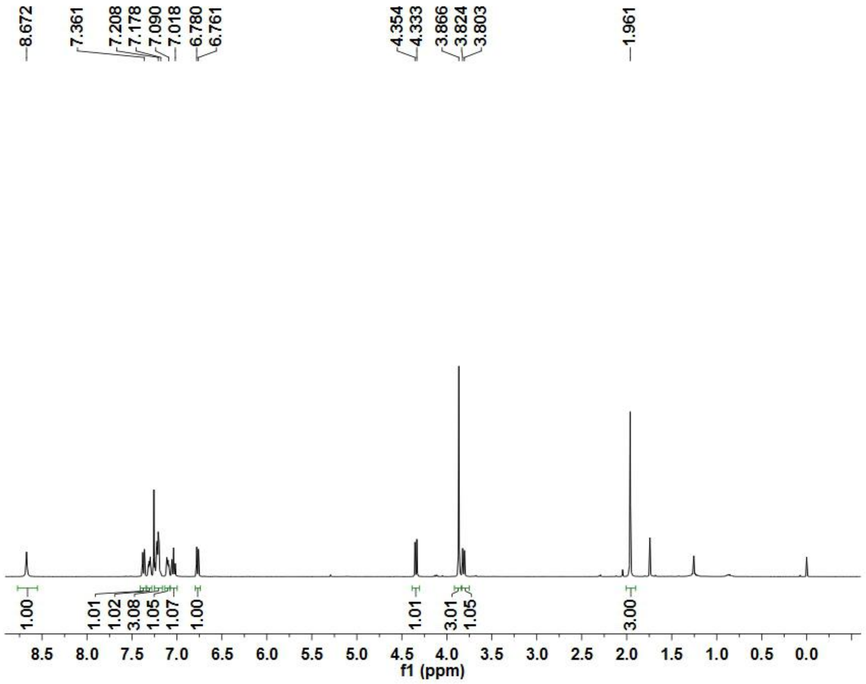

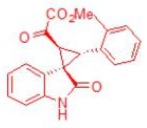

3ak'

Current Data Parameters

F2 - Acquisition Parameters
DATE: 2015-01-29T01:43:23
PULPROG: zg30
TD: 32768
Solvent: CDCl3
NS: 64
DS: undefined
SWH: 8223.7 Hz
AQ: undefined
TE: 293.8 C

======== CHANNEL f1 ========
NUC1: 1H
P1: 9.93 usec
SFO1: undefined MHz

F2 - Processing Parameters
SI: 65536
DC: 0.05
LB: 0.30 Hz
First Point: 0.50
FT: Hyper Quadrature
Phase: Manual
Ph0: -265.85
Ph1: 18.77

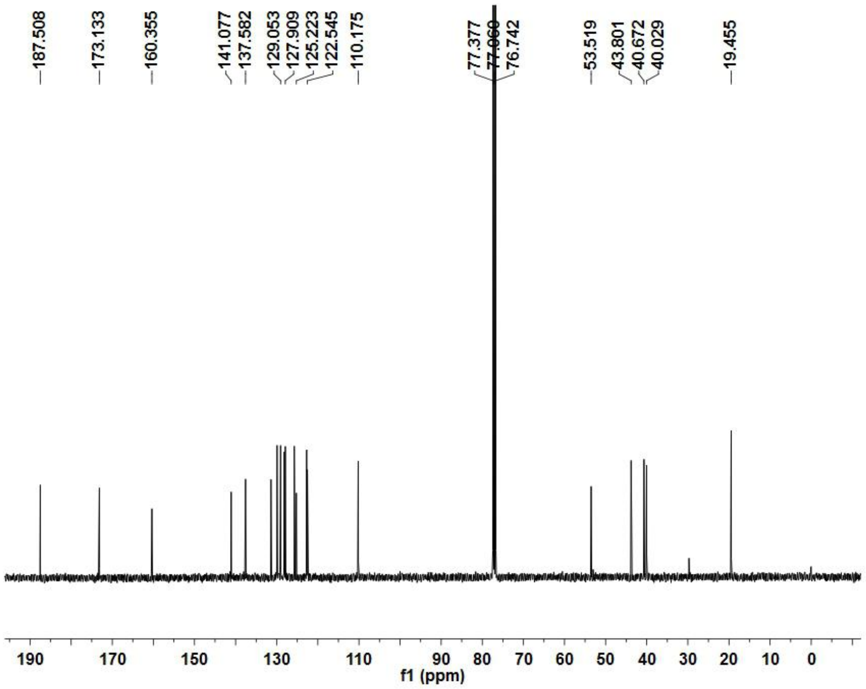

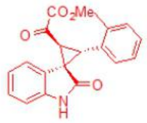

3ak'

Current Data Parameters

F2 - Acquisition Parameters
DATE: 2015-01-29T02:13:35
PULPROG: zgpg30
TD: 32768
Solvent: CDCl3
NS: 512
DS: undefined
SWH: 24038.5 Hz
AQ: undefined
TE: 294.3 C

======== CHANNEL f1 ========
NUC1: 13C
P1: 9.63 usec
SFO1: undefined MHz

F2 - Processing Parameters
SI: 65536
DC: 0.05
LB: 1.00 Hz
First Point: 0.50
FT: Hyper Quadrature
Phase: Manual
Ph0: -62.33
Ph1: 66.05

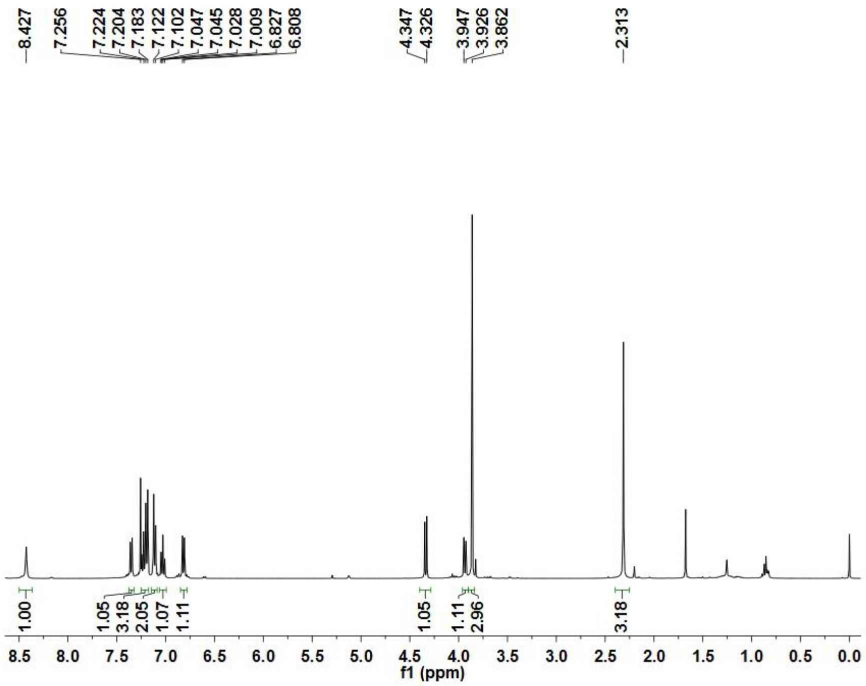

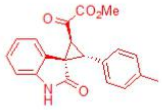

3af'

Current Data Parameters

F2 - Acquisition Parameters
DATE: 2014-11-05T19:55:38
PULPROG: zg30
TD: 32768
Solvent: CDCl3
NS: 32
DS: undefined
SWH: 8223.7 Hz
AQ: undefined
TE: 295.7 C

======== CHANNEL f1 ========
NUC1: 1H
P1: 9.93 usec
SFO1: undefined MHz

F2 - Processing Parameters
SI: 65536
DC: 0.05
LB: 0.30 Hz
First Point: 0.50
FT: Hyper Quadrature
Phase: Manual
Ph0: 91.85
Ph1: 13.50

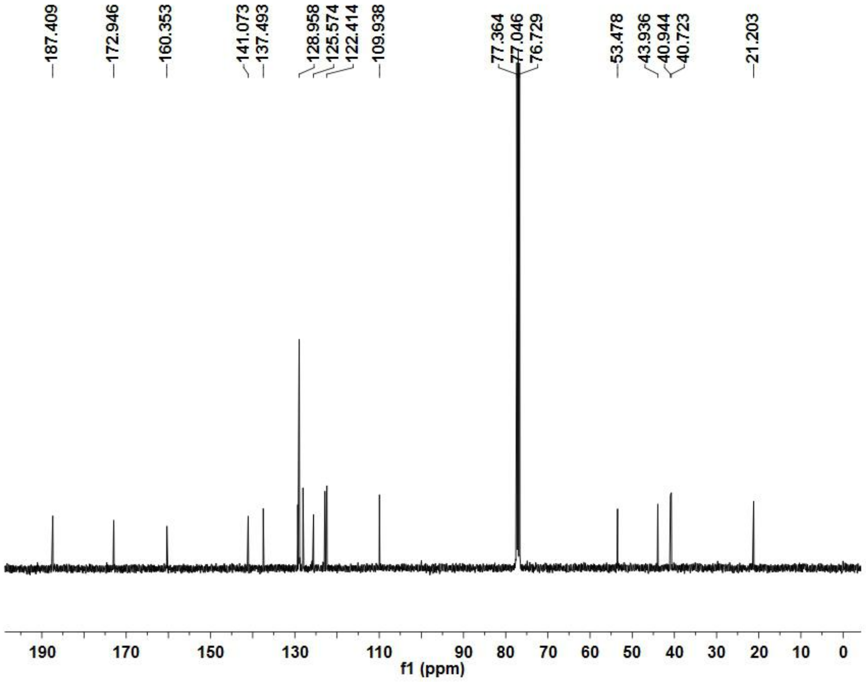

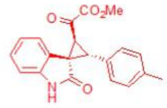

3af'

Current Data Parameters

F2 - Acquisition Parameters
DATE: 2014-11-05T20:25:49
PULPROG: zgpg30
TD: 32768
Solvent: CDCl3
NS: 512
DS: undefined
SWH: 24038.5 Hz
AQ: undefined
TE: 296 C

======== CHANNEL f1 ========
NUC1: 13C
P1: 9.63 usec
SFO1: undefined MHz

F2 - Processing Parameters
SI: 65536
DC: 0.05
LB: 1.00 Hz
First Point: 0.50
FT: Hyper Quadrature
Phase: Manual
Ph0: -76.40
Ph1: 71.00

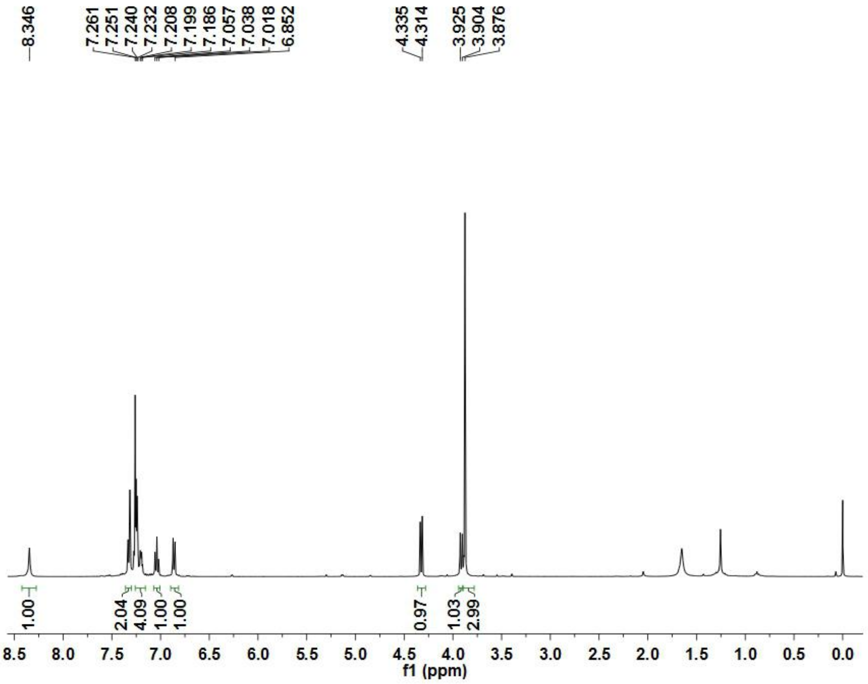

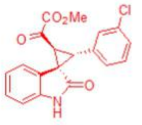

3am'

Current Data Parameters

F2 - Acquisition Parameters
DATE: 2015-07-07T15:53:17
PULPROG: zg30
TD: 32768
Solvent: CDCl3
NS: 64
DS: undefined
SWH: 8223.7 Hz
AQ: undefined
TE: 296.5 C

======== CHANNEL f1 ========
NUC1: 1H
P1: 9.93 usec
SFO1: undefined MHz

F2 - Processing Parameters
SI: 65536
DC: 0.05
LB: 0.30 Hz
First Point: 0.50
FT: Hyper Quadrature
Phase: Manual
Ph0: 92.45
Ph1: 10.24

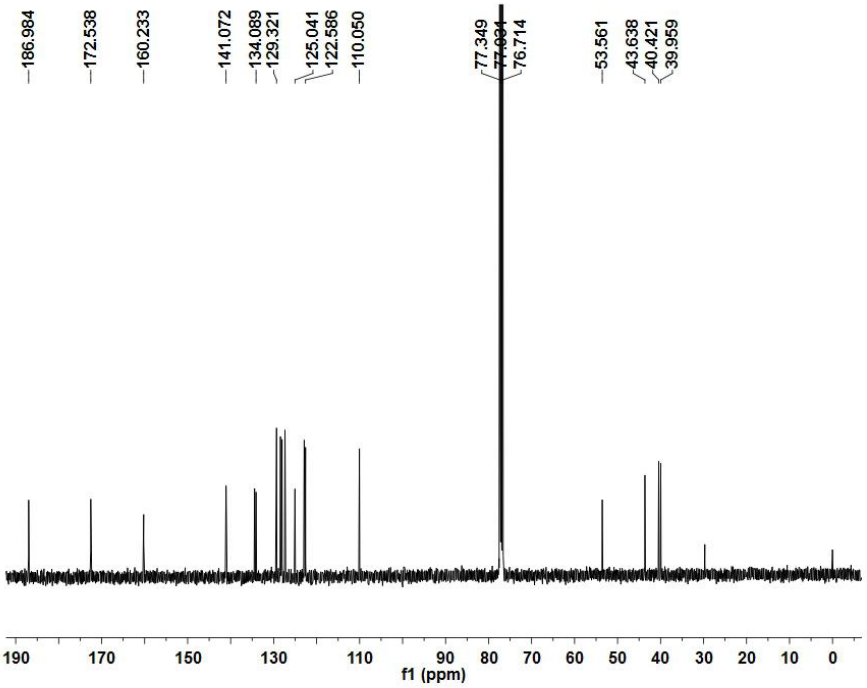

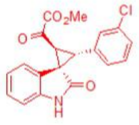

3am'

Current Data Parameters

F2 - Acquisition Parameters
DATE: 2015-07-07T16:52:58
PULPROG: zgpg30
TD: 32768
Solvent: CDCl3
NS: 1024
DS: undefined
SWH: 24038.5 Hz
AQ: undefined
TE: 296.9 C

======== CHANNEL f1 ========
NUC1: 13C
P1: 9.63 usec
SFO1: undefined MHz

F2 - Processing Parameters
SI: 65536
DC: 0.05
LB: 1.00 Hz
First Point: 0.50
FT: Hyper Quadrature
Phase: Manual
Ph0: -74.79
Ph1: 67.84

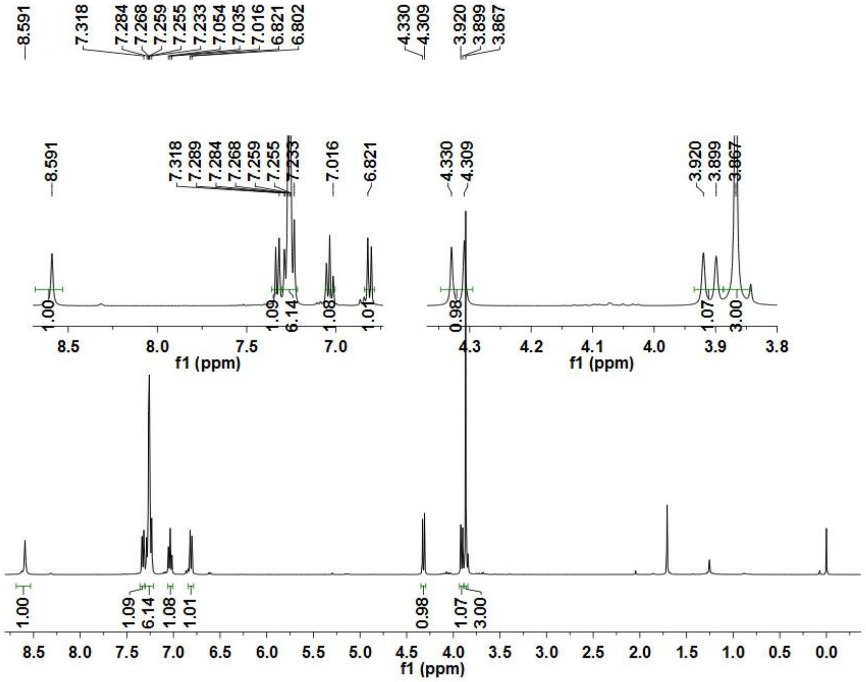

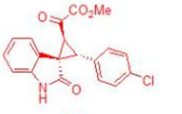

3an'

Current Data Parameters

F2 - Acquisition Parameters
DATE: 2014-11-15T17:27:55
PULPROG: zg30
TD: 32768
Solvent: CDCl3
NS: 64
DS: undefined
SWH: 8223.7 Hz
AQ: undefined
TE: 295.5 C

======== CHANNEL f1 ========
NUC1: 1H
P1: 9.93 usec
SFO1: undefined MHz

F2 - Processing Parameters
SI: 65536
DC: 0.05
LB: 0.30 Hz
First Point: 0.50
FT: Hyper Quadrature
Phase: Manual
Ph0: 91.48
Ph1: 16.56

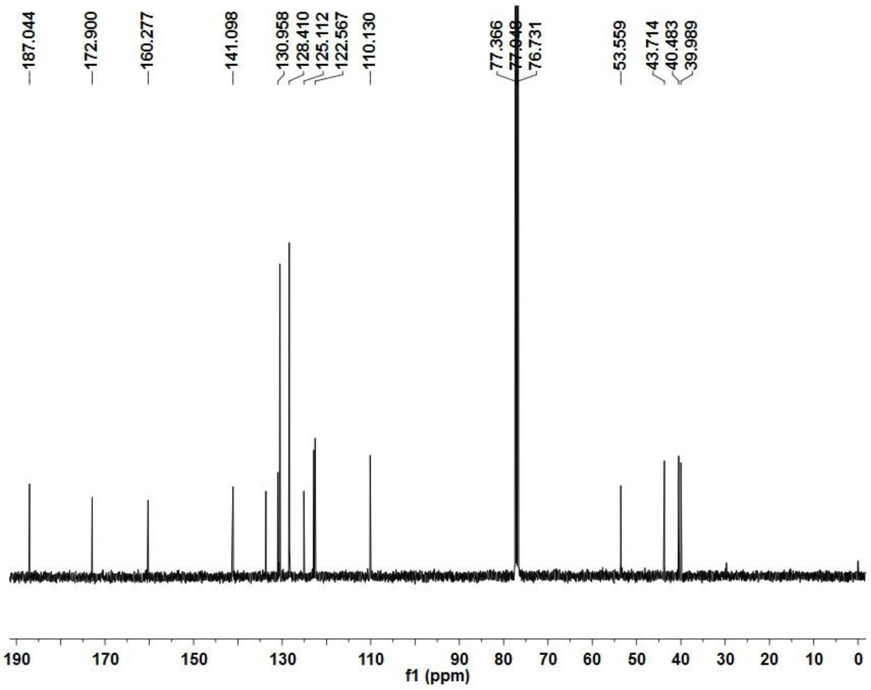

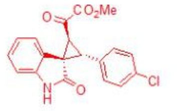

3an'

Current Data Parameters

F2 - Acquisition Parameters
DATE: 2014-11-15T17:58:07
PULPROG: zgpg30
TD: 32768
Solvent: CDCl3
NS: 512
DS: undefined
SWH: 24038.5 Hz
AQ: undefined
TE: 296.1 C

======== CHANNEL f1 ========
NUC1: 13C
P1: 9.63 usec
SFO1: undefined MHz

F2 - Processing Parameters
SI: 65536
DC: 0.05
LB: 1.00 Hz
First Point: 0.50
FT: Hyper Quadrature
Phase: Manual
Ph0: -69.78
Ph1: 62.71

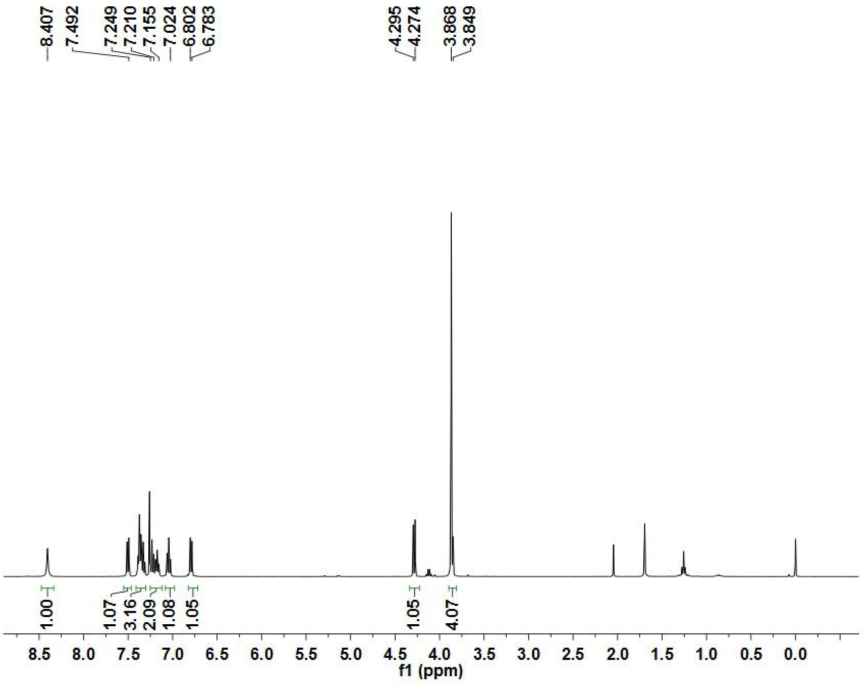

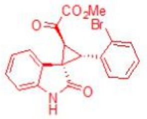

3ao'

Current Data Parameters

F2 - Acquisition Parameters
DATE: 2014-11-15T17:20:12
PULPROG: zg30
TD: 32768
Solvent: CDCl3
NS: 64
DS: undefined
SWH: 8223.7 Hz
AQ: undefined
TE: 295.7 C

======== CHANNEL f1 ========
NUC1: 1H
P1: 9.93 usec
SFO1: undefined MHz

F2 - Processing Parameters
SI: 65536
DC: 0.05
LB: 0.30 Hz
First Point: 0.50
FT: Hyper Quadrature
Phase: Manual
Ph0: 90.19
Ph1: 17.13

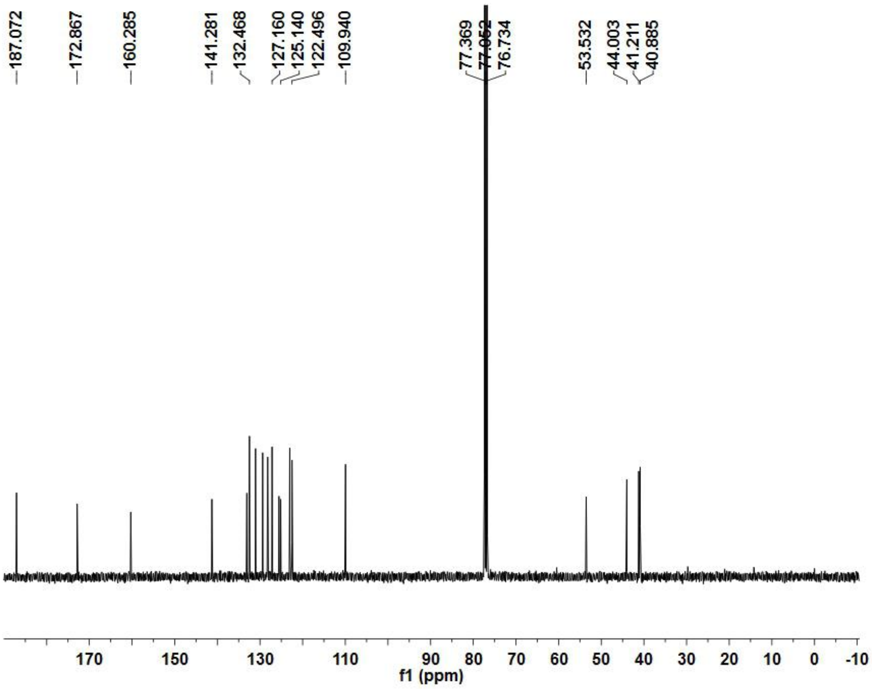

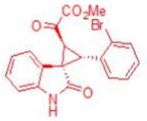

3ao'

Current Data Parameters

F2 - Acquisition Parameters
DATE: 2014-11-15T17:14:07
PULPROG: zgpg30
TD: 32768
Solvent: CDCl3
NS: 512
DS: undefined
SWH: 24038.5 Hz
AQ: undefined
TE: 296 C

======== CHANNEL f1 ========
NUC1: 13C
P1: 9.63 usec
SFO1: undefined MHz

F2 - Processing Parameters
SI: 65536
DC: 0.05
LB: 1.00 Hz
First Point: 0.50
FT: Hyper Quadrature
Phase: Manual
Ph0: -70.77
Ph1: 62.72

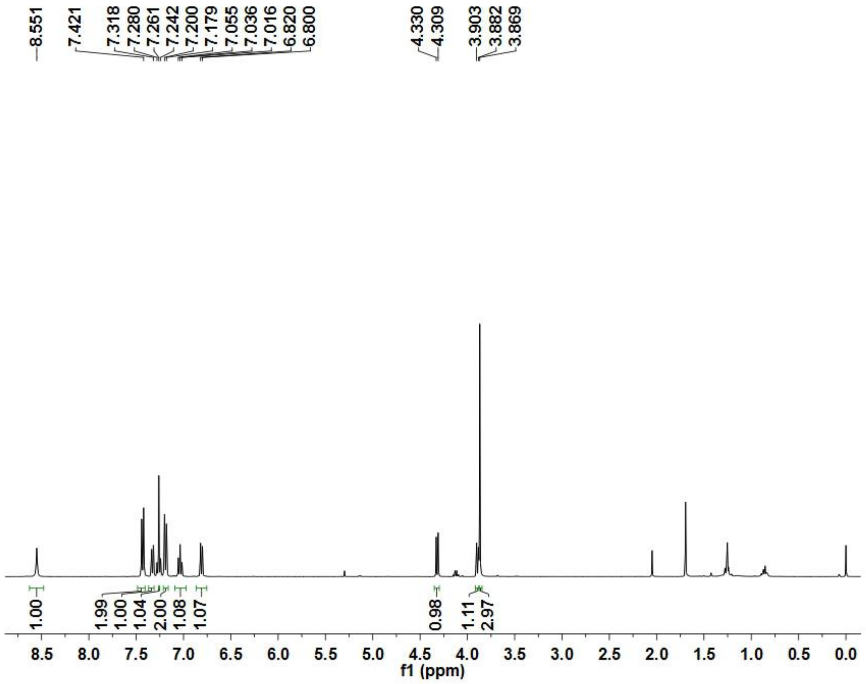

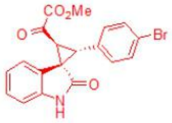

3ap'

Current Data Parameters

F2 - Acquisition Parameters
DATE: 2015-01-29T04:55:35
PULPROG: zg30
TD: 32768
Solvent: CDCl3
NS: 64
DS: undefined
SWH: 8223.7 Hz
AQ: undefined
TE: 293.9 C

======== CHANNEL f1 ========
NUC1: 1H
P1: 9.93 usec
SFO1: undefined MHz

F2 - Processing Parameters
SI: 65536
DC: 0.05
LB: 0.30 Hz
First Point: 0.50
FT: Hyper Quadrature
Phase: Manual
Ph0: 93.33
Ph1: 16.76

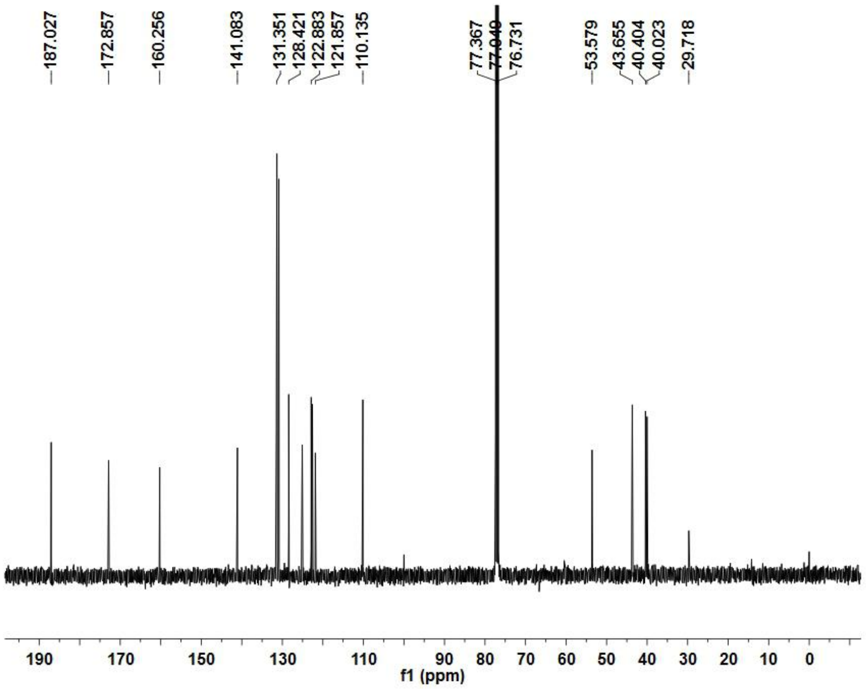

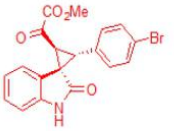

3ap'

Current Data Parameters

F2 - Acquisition Parameters
DATE: 2015-01-29T05:25:47
PULPROG: zgpg30
TD: 32768
Solvent: CDCl3
NS: 512
DS: undefined
SWH: 24038.5 Hz
AQ: undefined
TE: 294.4 C

======== CHANNEL f1 ========
NUC1: 13C
P1: 9.63 usec
SFO1: undefined MHz

F2 - Processing Parameters
SI: 65536
DC: 0.05
LB: 1.00 Hz
First Point: 0.50
FT: Hyper Quadrature
Phase: Manual
Ph0: -42.11
Ph1: 23.69

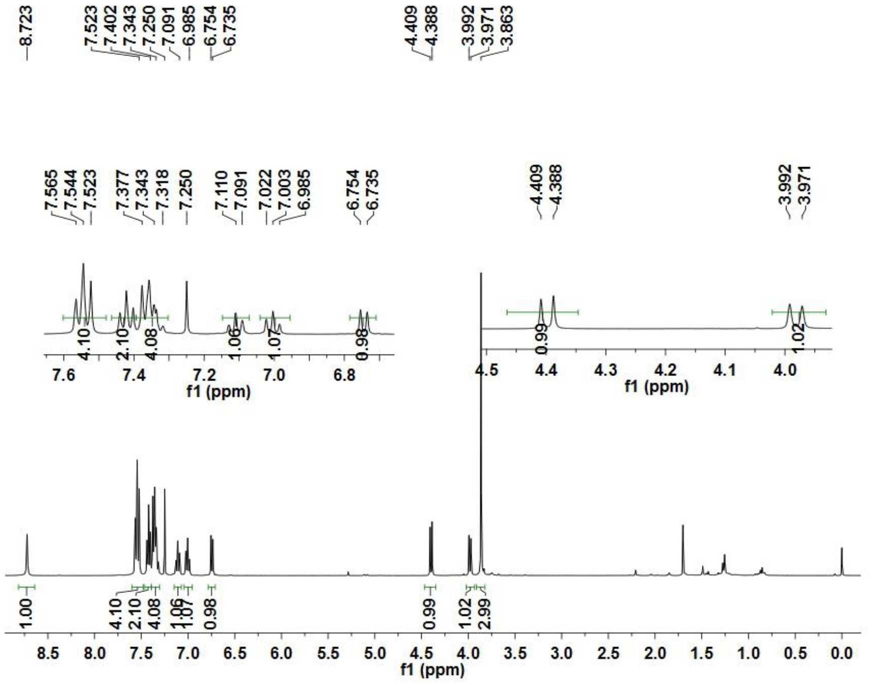

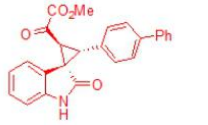

3aq'

Current Data Parameters

F2 - Acquisition Parameters
DATE: 2014-11-05T19:19:53
PULPROG: zg30
TD: 32768
Solvent: CDCl3
NS: 32
DS: undefined
SWH: 8223.7 Hz
AQ: undefined
TE: 295.6 C

======== CHANNEL f1 ========
NUC1: 1H
P1: 9.93 usec
SFO1: undefined MHz

F2 - Processing Parameters
SI: 65536
DC: 0.05
LB: 0.30 Hz
First Point: 0.50
FT: Hyper Quadrature
Phase: Manual
Ph0: 95.23
Ph1: 9.39

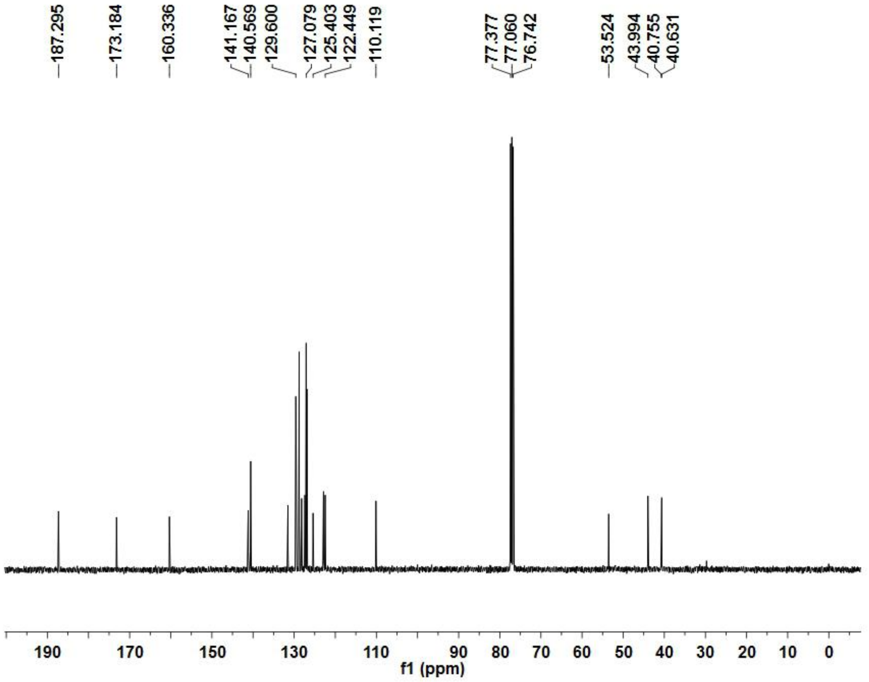

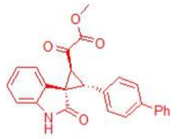

3aq'

Current Data Parameters

F2 - Acquisition Parameters
DATE: 2014-11-05T19:50:05
PULPROG: zgpg30
TD: 32768
Solvent: CDCl3
NS: 512
DS: undefined
SWH: 24038.5 Hz
AQ: undefined
TE: 296 C

======== CHANNEL f1 ========
NUC1: 13C
P1: 9.63 usec
SFO1: undefined MHz

F2 - Processing Parameters
SI: 65536
DC: 0.05
LB: 1.00 Hz
First Point: 0.50
FT: Hyper Quadrature
Phase: Manual
Ph0: -74.15
Ph1: 70.21

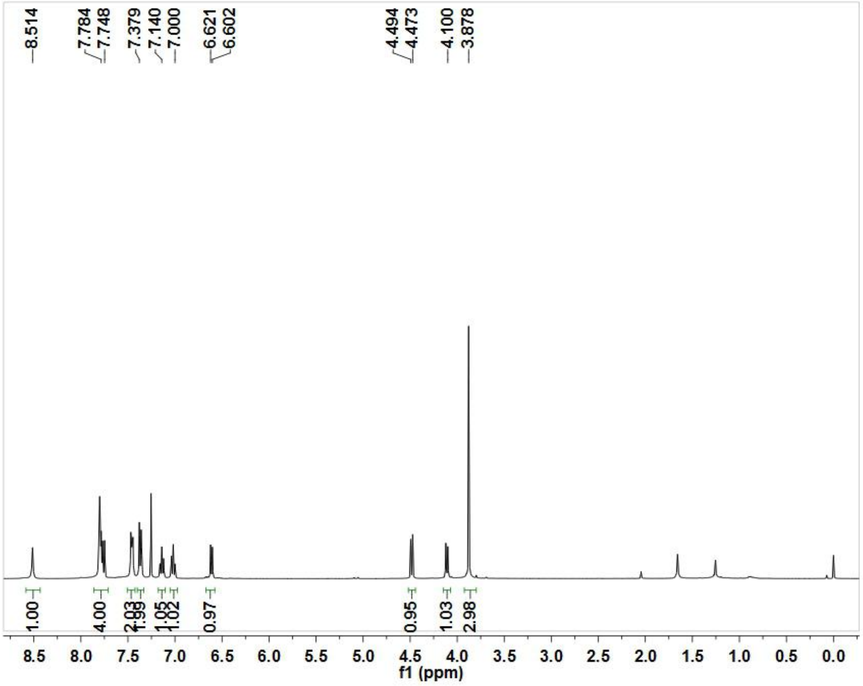

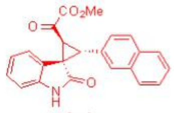

3ar'

Current Data Parameters

F2 - Acquisition Parameters
DATE: 2015-02-25T20:32:24
PULPROG: zg30
TD: 32768
Solvent: CDCl3
NS: 128
DS: undefined
SWH: 8223.7 Hz
AQ: undefined
TE: 293.6 C

======== CHANNEL f1 ========
NUC1: 1H
P1: 9.93 usec
SFO1: undefined MHz

F2 - Processing Parameters
SI: 65536
DC: 0.05
LB: 0.30 Hz
First Point: 0.50
FT: Hyper Quadrature
Phase: Manual
Ph0: 99.66
Ph1: 6.08

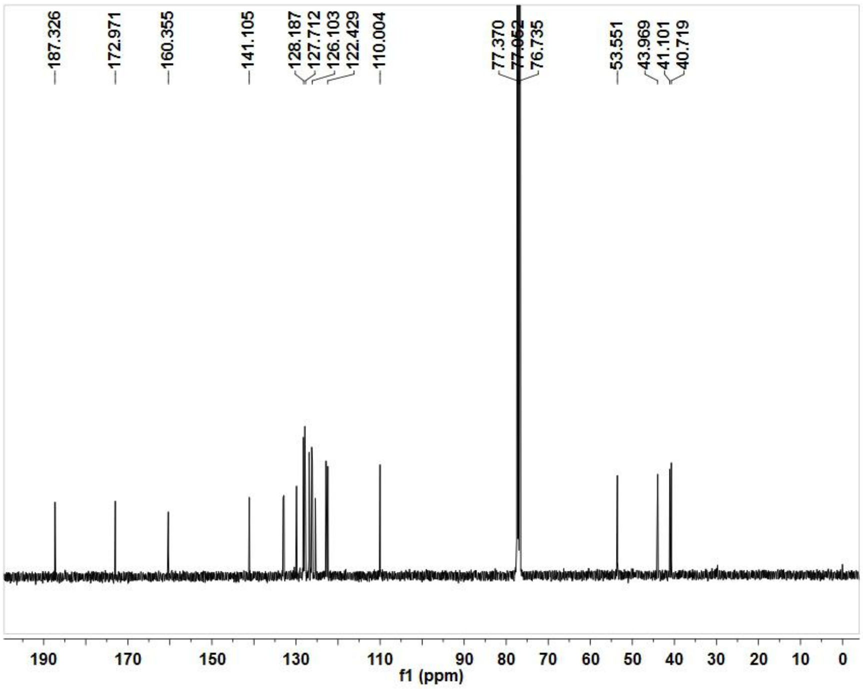

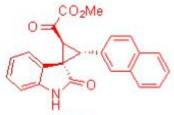

3ar'

Current Data Parameters

F2 - Acquisition Parameters
DATE: 2015-02-25T21:31:51
PULPROG: zgpg30
TD: 32768
Solvent: CDCl3
NS: 1024
DS: undefined
SWH: 24038.5 Hz
AQ: undefined
TE: 294 C

======== CHANNEL f1 ========
NUC1: 13C
P1: 9.63 usec
SFO1: undefined MHz

F2 - Processing Parameters
SI: 65536
DC: 0.05
LB: 1.00 Hz
First Point: 0.50
FT: Hyper Quadrature
Phase: Manual
Ph0: -63.13
Ph1: 49.12

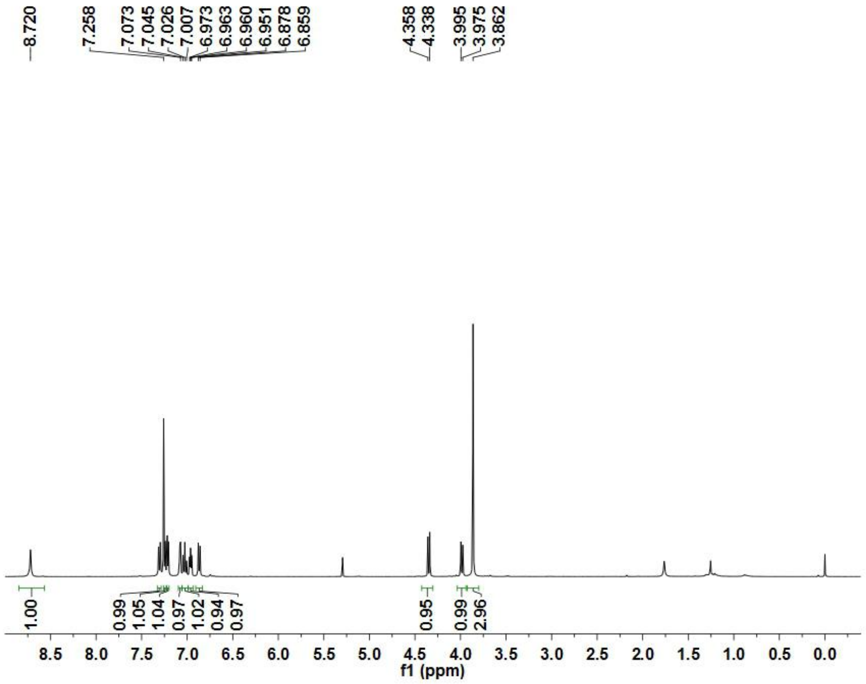

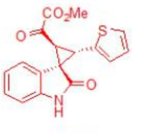

3as'

Current Data Parameters

F2 - Acquisition Parameters
DATE: 2015-01-22T23:57:42
PULPROG: zg30
TD: 32768
Solvent: CDCl3
NS: 64
DS: undefined
SWH: 8223.7 Hz
AQ: undefined
TE: 293.8 C

======== CHANNEL f1 ========
NUC1: 1H
P1: 9.93 usec
SFO1: undefined MHz

F2 - Processing Parameters
SI: 65536
DC: 0.05
LB: 0.30 Hz
First Point: 0.50
FT: Hyper Quadrature
Phase: Manual
Ph0: 97.83
Ph1: 15.30

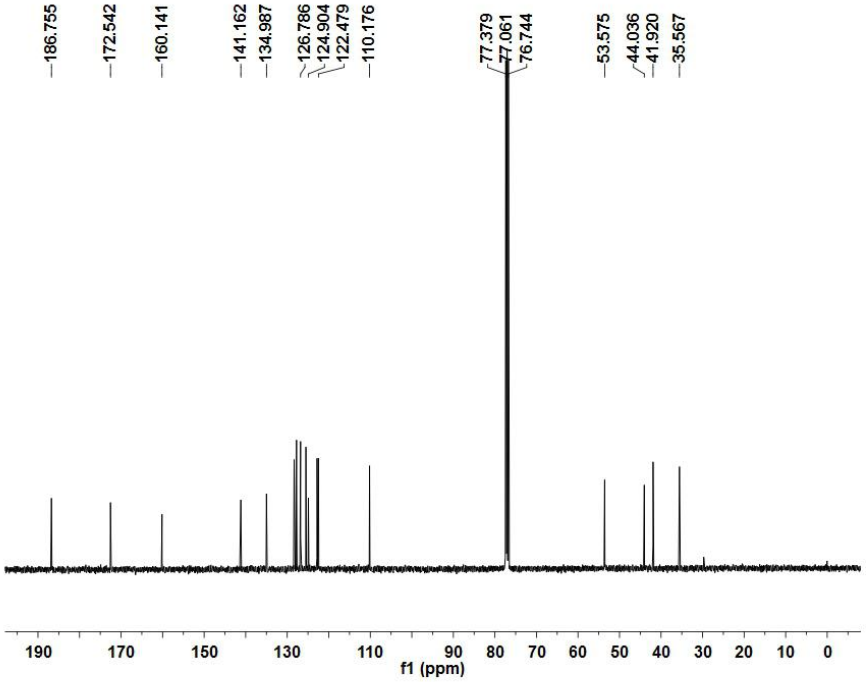

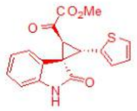

3as'

Current Data Parameters

F2 - Acquisition Parameters
DATE: 2015-01-23T00:27:54
PULPROG: zgpg30
TD: 32768
Solvent: CDCl3
NS: 512
DS: undefined
SWH: 24038.5 Hz
AQ: undefined
TE: 294.2 C

======== CHANNEL f1 ========
NUC1: 13C
P1: 9.63 usec
SFO1: undefined MHz

F2 - Processing Parameters
SI: 65536
DC: 0.05
LB: 1.00 Hz
First Point: 0.50
FT: Hyper Quadrature
Phase: Manual
Ph0: -74.89
Ph1: 81.37

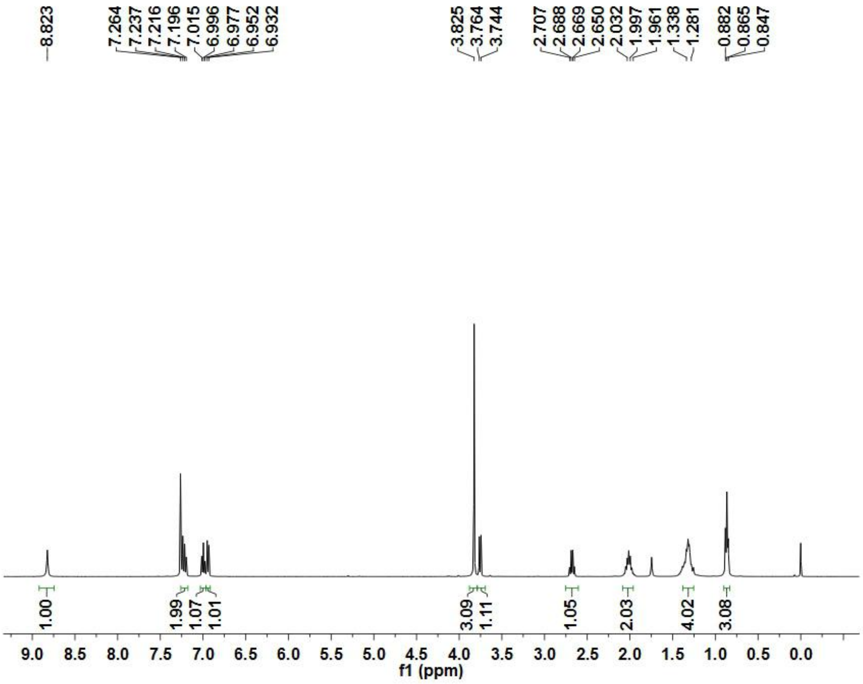

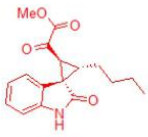

3at'

Current Data Parameters

F2 - Acquisition Parameters
DATE: 2015-02-03T03:24:17
PULPROG: zg30
TD: 32768
Solvent: CDCl3
NS: 64
DS: undefined
SWH: 8223.7 Hz
AQ: undefined
TE: 294 C

======== CHANNEL f1 ========
NUC1: 1H
P1: 9.93 usec
SFO1: undefined MHz

F2 - Processing Parameters
SI: 65536
DC: 0.05
LB: 0.30 Hz
First Point: 0.50
FT: Hyper Quadrature
Phase: Manual
Ph0: 91.87
Ph1: 20.29

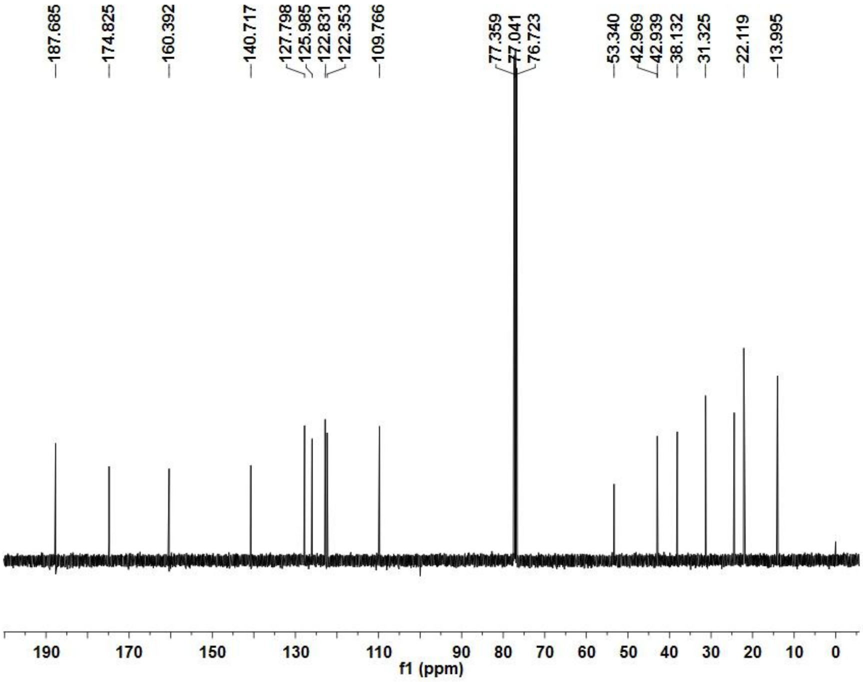

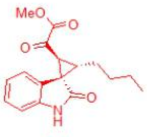

3at'

Current Data Parameters

F2 - Acquisition Parameters
DATE: 2015-02-03T03:54:29
PULPROG: zgpg30
TD: 32768
Solvent: CDCl3
NS: 512
DS: undefined
SWH: 24038.5 Hz
AQ: undefined
TE: 294.4 C

======== CHANNEL f1 ========
NUC1: 13C
P1: 9.63 usec
SFO1: undefined MHz

F2 - Processing Parameters
SI: 65536
DC: 0.05
First Point: 0.50
FT: Hyper Quadrature
Phase: Global

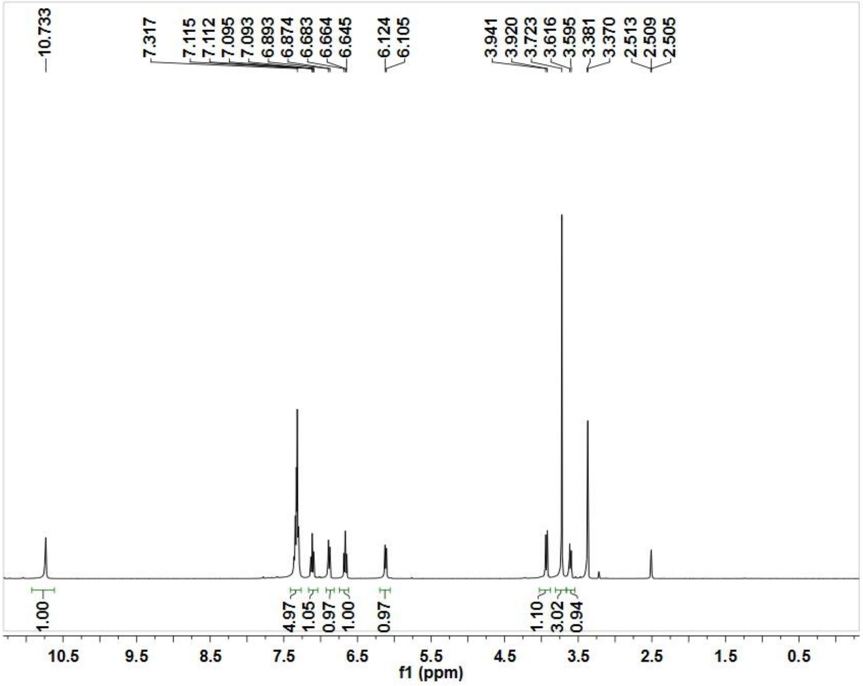

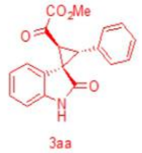
3aa
Current Data Parameters
F2 - Acquisition Parameters
DATE: 2015-04-01T23:15:58
PULPROG: zg30
TD: 32768
Solvent: DMSO
NS: 64
DS: undefined
SWH: 8223.7 Hz
AQ: undefined
TE: 295.4 C
======== CHANNEL f1 ========
NUC1: 1H
P1: 9.93 usec
SFO1: undefined MHz
F2 - Processing Parameters
SI: 65536
DC: 0.05
LB: 0.30 Hz
First Point: 0.50
FT: Hyper Quadrature
Phase: Manual
Ph0: -263.67
Ph1: 22.35

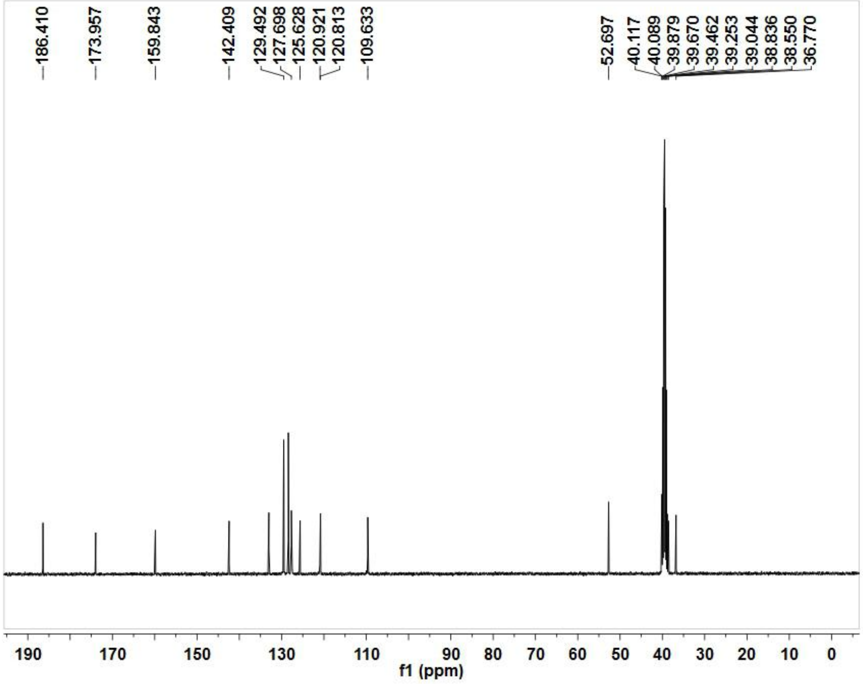

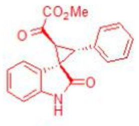
3aa
Current Data Parameters
F2 - Acquisition Parameters
DATE: 2015-04-01T23:46:10
PULPROG: zgpg30
TD: 32768
Solvent: DMSO
NS: 512
DS: undefined
SWH: 24038.5 Hz
AQ: undefined
TE: 295.8 C
======== CHANNEL f1 ========
NUC1: 13C
P1: 9.63 usec
SFO1: undefined MHz
F2 - Processing Parameters
SI: 65536
DC: 0.05
LB: 1.00 Hz
First Point: 0.50
FT: Hyper Quadrature
Phase: Manual
Ph0: -66.42
Ph1: 58.90

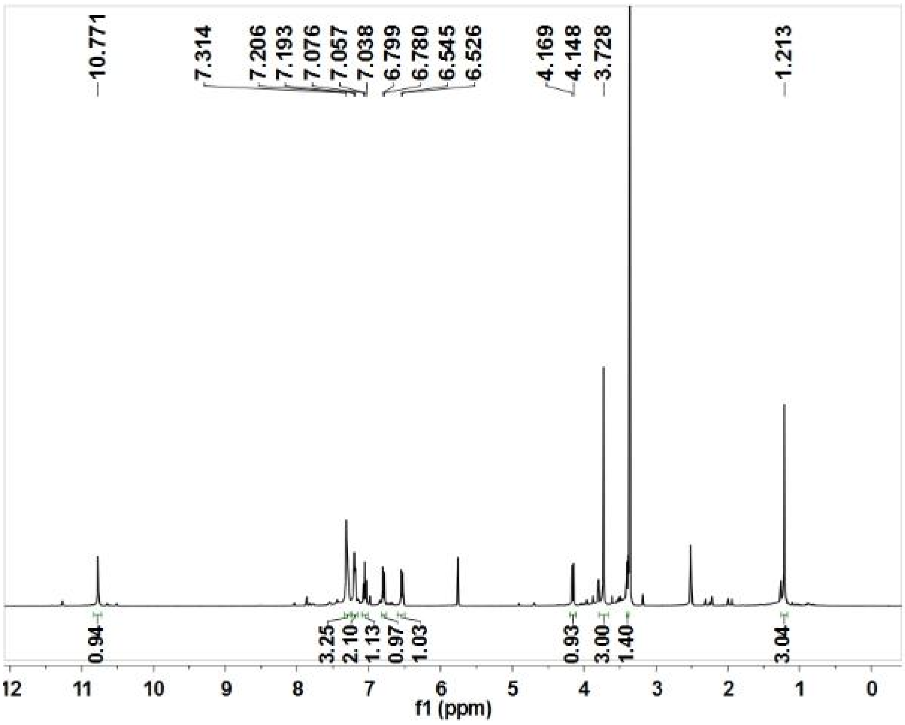

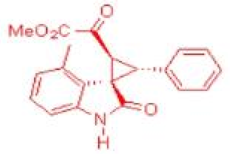

3ba

Current Data Parameters

F2 - Acquisition Parameters
DATE: 2017-10-11T21:56:41
PULPROG: zg30
TD: 32768
Solvent: DMSO
NS: 32
DS: undefined
SWH: 8223.7 Hz
AQ: undefined
TE: 296.9 C

======== CHANNEL f1 ========
NUC1: 1H
P1: 9.93 usec
SFO1: undefined MHz

F2 - Processing Parameters
SI: 65536
DC: 0.05
LB: 0.30 Hz
First Point: 0.50
FT: Hyper Quadrature
Phase: Manual
Ph0: 93.33
Ph1: 20.01

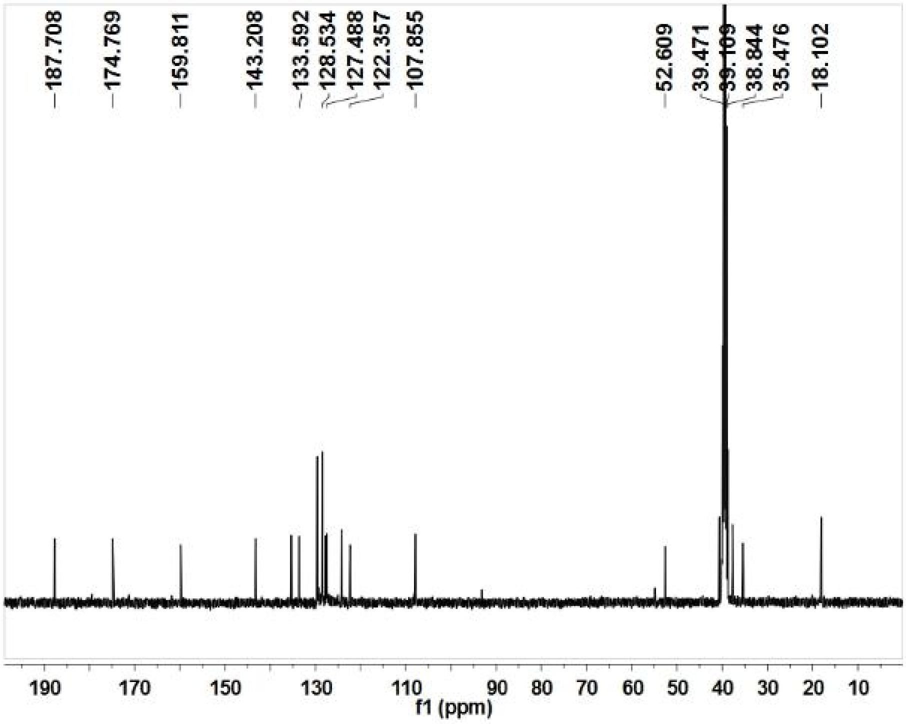

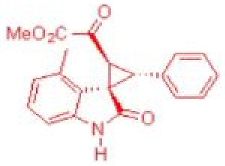

3ba

Current Data Parameters

F2 - Acquisition Parameters
DATE: 2017-10-11T22:26:59
PULPROG: zgpg30
TD: 32768
Solvent: DMSO
NS: 512
DS: undefined
SWH: 24038.5 Hz
AQ: undefined
TE: 297.3 C

======== CHANNEL f1 ========
NUC1: 13C
P1: 9.63 usec
SFO1: undefined MHz

F2 - Processing Parameters
SI: 65536
DC: 0.05
LB: 1.00 Hz
First Point: 0.50
FT: Hyper Quadrature
Phase: Manual
Ph0: -78.08
Ph1: 80.20

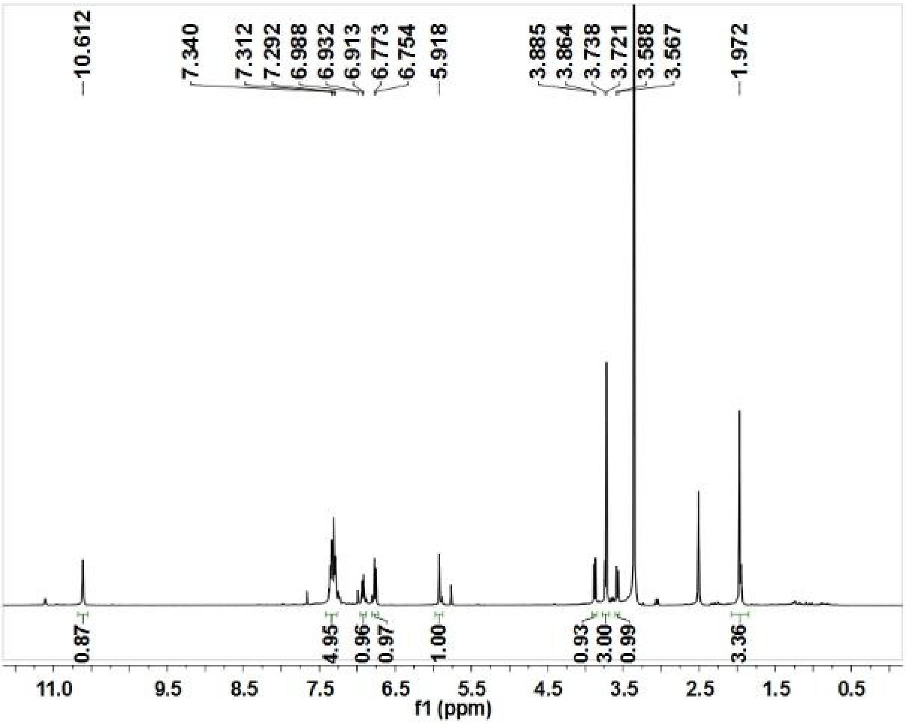

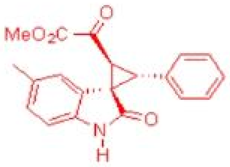

3da

Current Data Parameters

F2 - Acquisition Parameters
DATE: 2017-10-11T22:31:33
PULPROG: zg30
TD: 32768
Solvent: DMSO
NS: 32
DS: undefined
SWH: 8223.7 Hz
AQ: undefined
TE: 297.2 C

======== CHANNEL f1 ========
NUC1: 1H
P1: 9.93 usec
SFO1: undefined MHz

F2 - Processing Parameters
SI: 65536
DC: 0.05
LB: 0.30 Hz
First Point: 0.50
FT: Hyper Quadrature
Phase: Manual
Ph0: 96.00
Ph1: 16.93

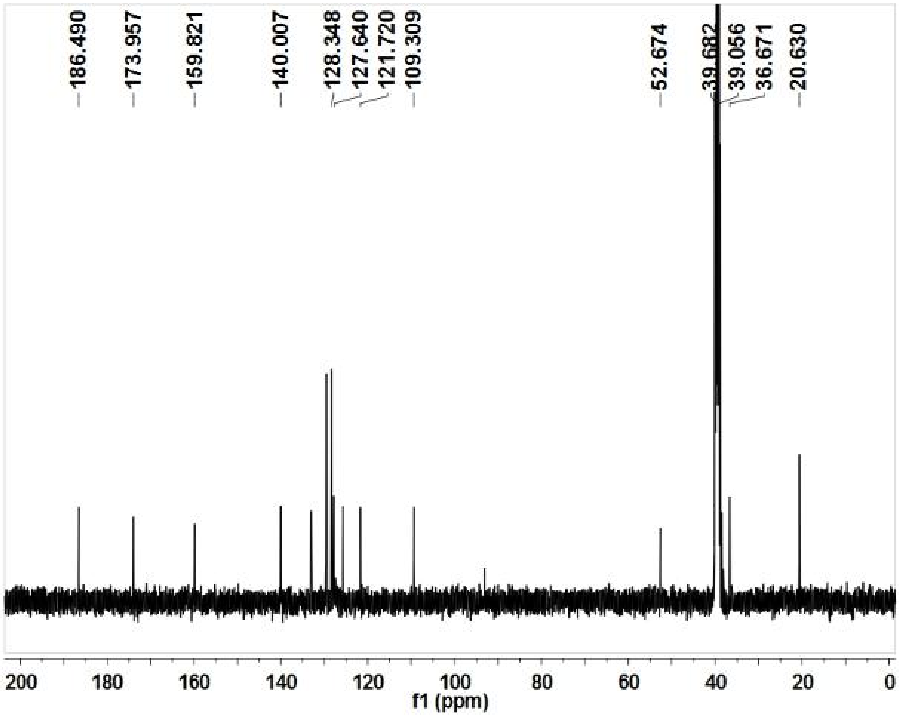

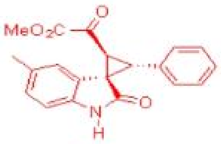

3da

Current Data Parameters

F2 - Acquisition Parameters
DATE: 2017-10-11T23:01:45
PULPROG: zgpg30
TD: 32768
Solvent: DMSO
NS: 512
DS: undefined
SWH: 24038.5 Hz
AQ: undefined
TE: 297.4 C

======== CHANNEL f1 ========
NUC1: 13C
P1: 9.63 usec
SFO1: undefined MHz

F2 - Processing Parameters
SI: 65536
DC: 0.05
LB: 1.00 Hz
First Point: 0.50
FT: Hyper Quadrature
Phase: Manual
Ph0: -59.00
Ph1: 58.75

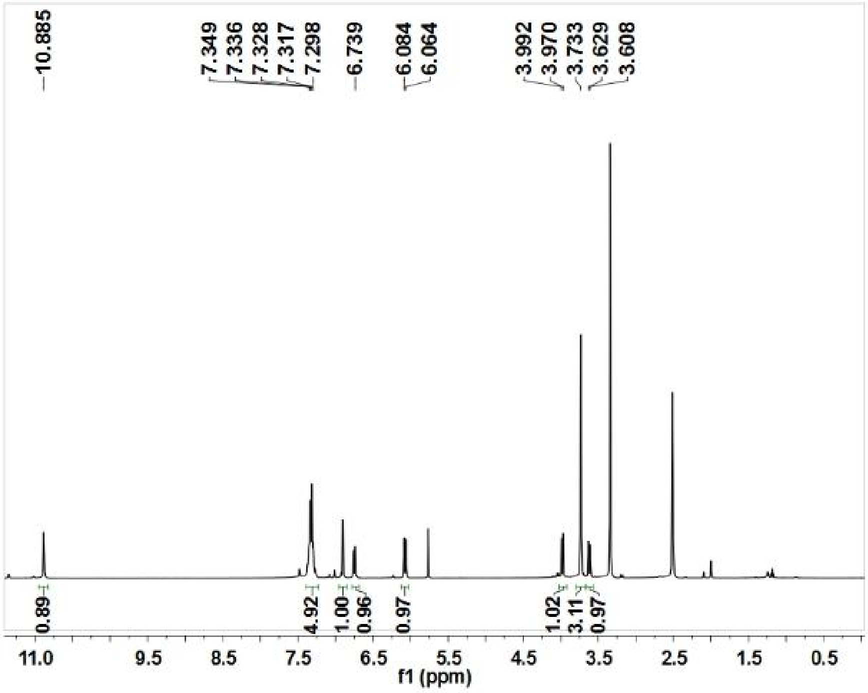

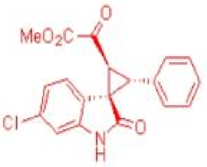

3ha

Current Data Parameters

F2 - Acquisition Parameters
DATE: 2017-10-17T13:29:11
PULPROG: zg30
TD: 32768
Solvent: DMSO
NS: 32
DS: undefined
SWH: 8223.7 Hz
AQ: undefined
TE: 297.1 C

======= CHANNEL f1 =======
NUC1: 1H
P1: 9.93 usec
SFO1: undefined MHz

F2 - Processing Parameters
SI: 65536
DC: 0.05
LB: 0.30 Hz
First Point: 0.50
FT: Hyper Quadrature
Phase: Manual
Ph0: 88.01
Ph1: 20.77

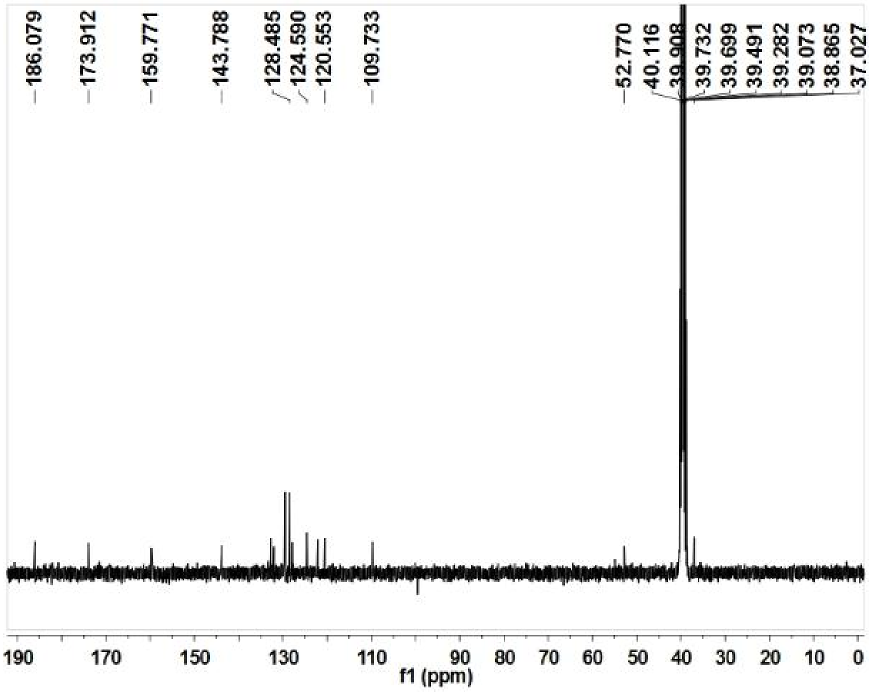

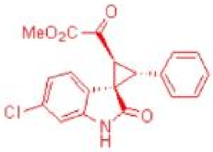

3ha

Current Data Parameters

F2 - Acquisition Parameters
DATE: 2017-10-17T13:59:24
PULPROG: zgpg30
TD: 32768
Solvent: DMSO
NS: 512
DS: undefined
SWH: 24038.5 Hz
AQ: undefined
TE: 297.5 C

======= CHANNEL f1 =======
NUC1: 13C
P1: 9.63 usec
SFO1: undefined MHz

F2 - Processing Parameters
SI: 65536
DC: 0.05
LB: 1.00 Hz
First Point: 0.50
FT: Hyper Quadrature
Phase: Manual
Ph0: -30.76
Ph1: 12.62

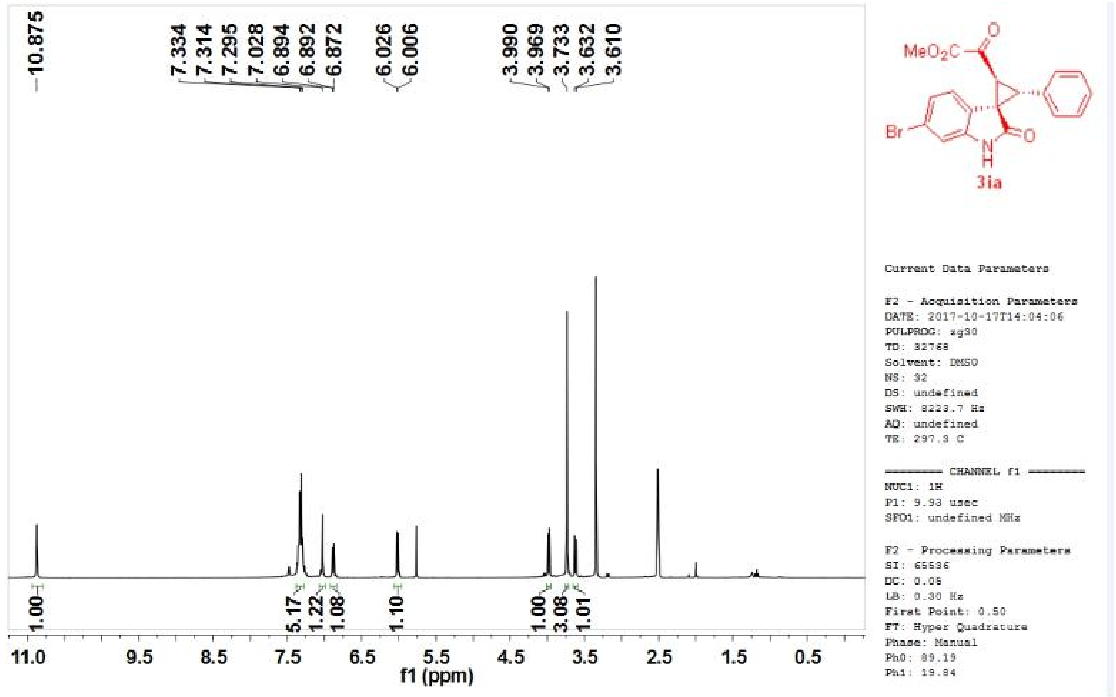

10.875
7.334
7.314
7.295
7.028
6.894
6.892
6.872
6.026
6.006
3.990
3.969
3.733
3.632
3.610
1.00
5.17
1.22
1.08
1.10
1.00
3.08
1.01
11.0
9.5
8.5
7.5
6.5
5.5
4.5
3.5
2.5
1.5
0.5
f1 (ppm)
MeO2C
O
Br
N
H
O
3ia
Current Data Parameters
F2 - Acquisition Parameters
DATE: 2017-10-17T14:04:06
PULPROG: zg30
TD: 32768
Solvent: DMSO
NS: 32
DS: undefined
SWH: 8223.7 Hz
AQ: undefined
TE: 297.3 C
CHANNEL f1
NUC1: 1H
P1: 9.93 usec
SFO1: undefined MHz
F2 - Processing Parameters
SI: 65536
DC: 0.05
LB: 0.30 Hz
First Point: 0.50
FT: Hyper Quadrature
Phase: Manual
Ph0: 89.19
Ph1: 19.84

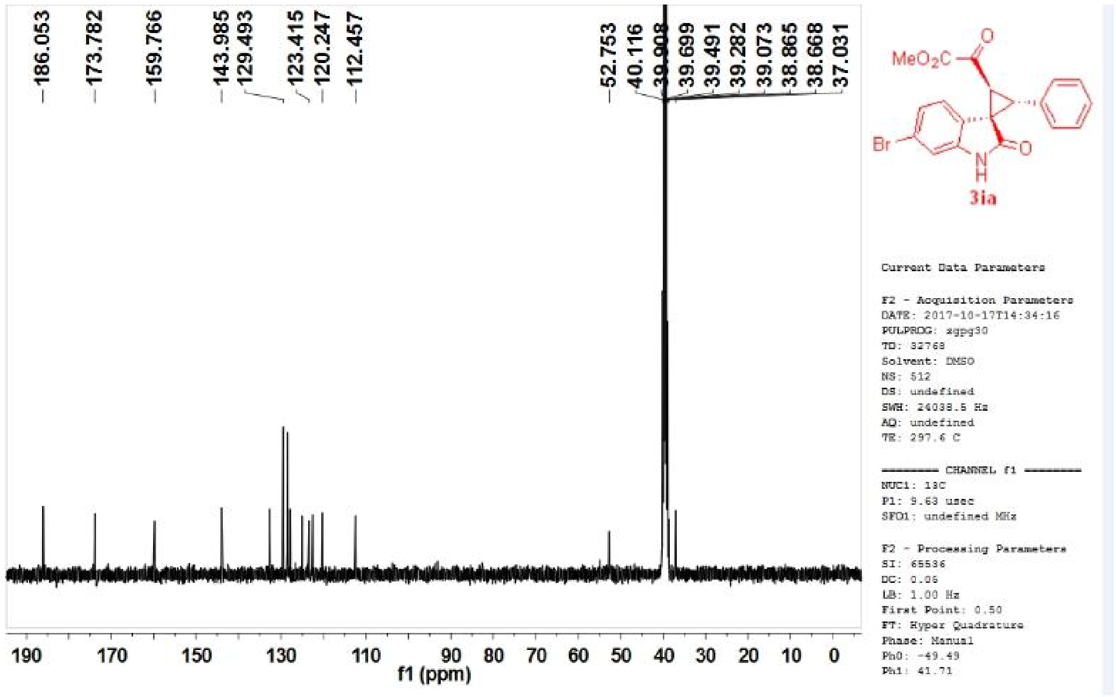

186.053
173.782
159.766
143.985
129.493
123.415
120.247
112.457
52.753
40.116
39.908
39.699
39.491
39.282
39.073
38.865
38.668
37.031
190
170
150
130
110
90
80
70
60
50
40
30
20
10
0
f1 (ppm)
MeO2C
O
Br
N
H
O
3ia
Current Data Parameters
F2 - Acquisition Parameters
DATE: 2017-10-17T14:34:16
PULPROG: zgpg30
TD: 32768
Solvent: DMSO
NS: 512
DS: undefined
SWH: 24038.5 Hz
AQ: undefined
TE: 297.6 C
CHANNEL f1
NUC1: 13C
P1: 9.63 usec
SFO1: undefined MHz
F2 - Processing Parameters
SI: 65536
DC: 0.05
LB: 1.00 Hz
First Point: 0.50
FT: Hyper Quadrature
Phase: Manual
Ph0: -49.49
Ph1: 41.71

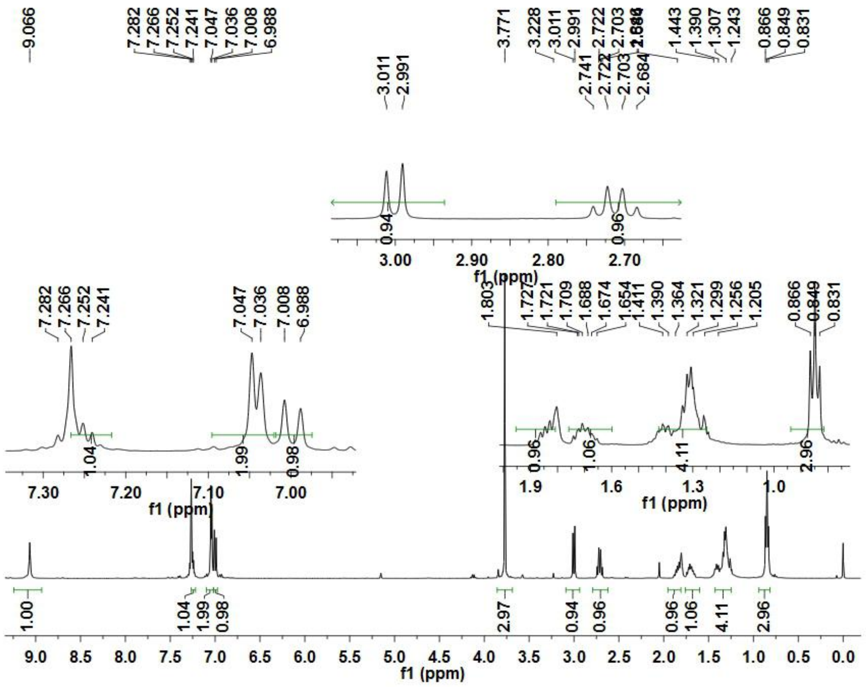

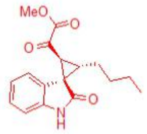

3at

Current Data Parameters

F2 - Acquisition Parameters
DATE: 2015-02-03T15:13:13
PULPROG: zg30
TD: 32768
Solvent: CDCl3
NS: 64
DS: undefined
SWH: 8223.7 Hz
AQ: undefined
TE: 293.8 C

======== CHANNEL f1 ========
NUC1: 1H
P1: 9.93 usec
SFO1: undefined MHz

F2 - Processing Parameters
SI: 65536
DC: 0.05
LB: 0.30 Hz
First Point: 0.50
FT: Hyper Quadrature
Phase: Manual
Ph0: -264.88
Ph1: 19.32

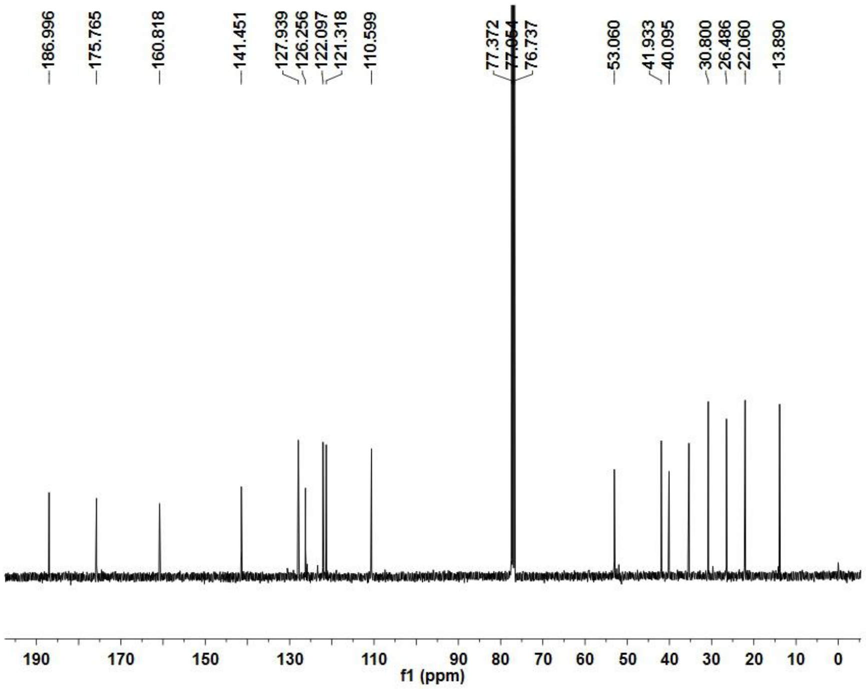

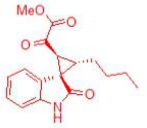

3at

Current Data Parameters

F2 - Acquisition Parameters
DATE: 2015-02-03T15:43:25
PULPROG: zgpg30
TD: 32768
Solvent: CDCl3
NS: 512
DS: undefined
SWH: 24038.5 Hz
AQ: undefined
TE: 294.3 C

======== CHANNEL f1 ========
NUC1: 13C
P1: 9.63 usec
SFO1: undefined MHz

F2 - Processing Parameters
SI: 65536
DC: 0.05
LB: 1.00 Hz
First Point: 0.50
FT: Hyper Quadrature
Phase: Manual
Ph0: -63.24
Ph1: 68.79

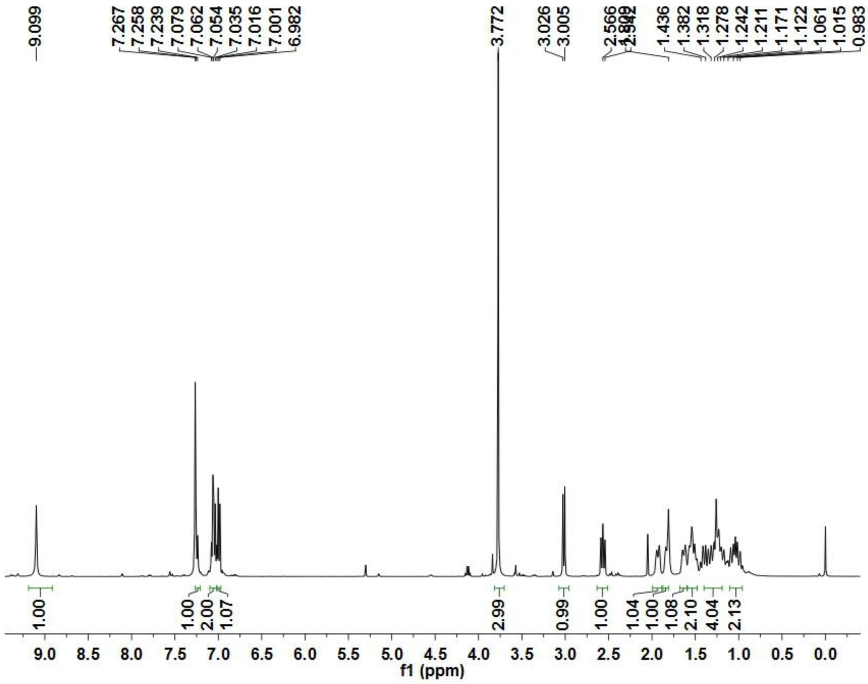

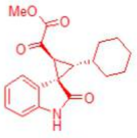

3au

Current Data Parameters

F2 - Acquisition Parameters
DATE: 2015-02-03T14:34:38
PULPROG: zg30
TD: 32768
Solvent: CDCl3
NS: 64
DS: undefined
SWH: 8223.7 Hz
AQ: undefined
TE: 293.8 C

======== CHANNEL f1 ========
NUC1: 1H
P1: 9.93 usec
SFO1: undefined MHz

F2 - Processing Parameters
SI: 65536
DC: 0.05
LB: 0.30 Hz
First Point: 0.50
FT: Hyper Quadrature
Phase: Manual
Ph0: -265.44
Ph1: 17.76

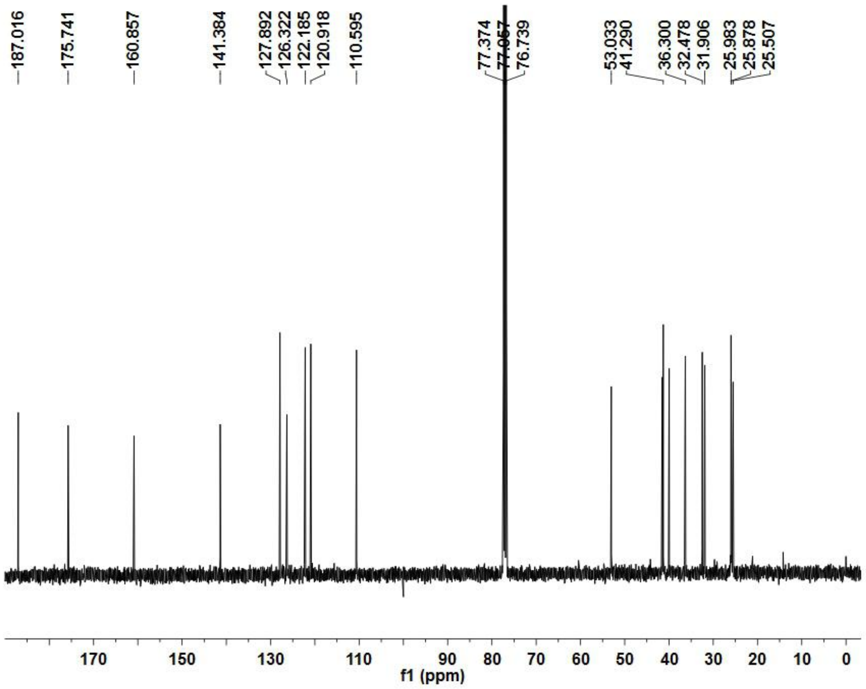

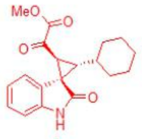

3au

Current Data Parameters

F2 - Acquisition Parameters
DATE: 2015-02-03T15:04:50
PULPROG: zgpg30
TD: 32768
Solvent: CDCl3
NS: 512
DS: undefined
SWH: 24038.5 Hz
AQ: undefined
TE: 294.1 C

======== CHANNEL f1 ========
NUC1: 13C
P1: 9.63 usec
SFO1: undefined MHz

F2 - Processing Parameters
SI: 65536
DC: 0.05
LB: 1.00 Hz
First Point: 0.50
FT: Hyper Quadrature
Phase: Manual
Ph0: -67.65
Ph1: 72.10

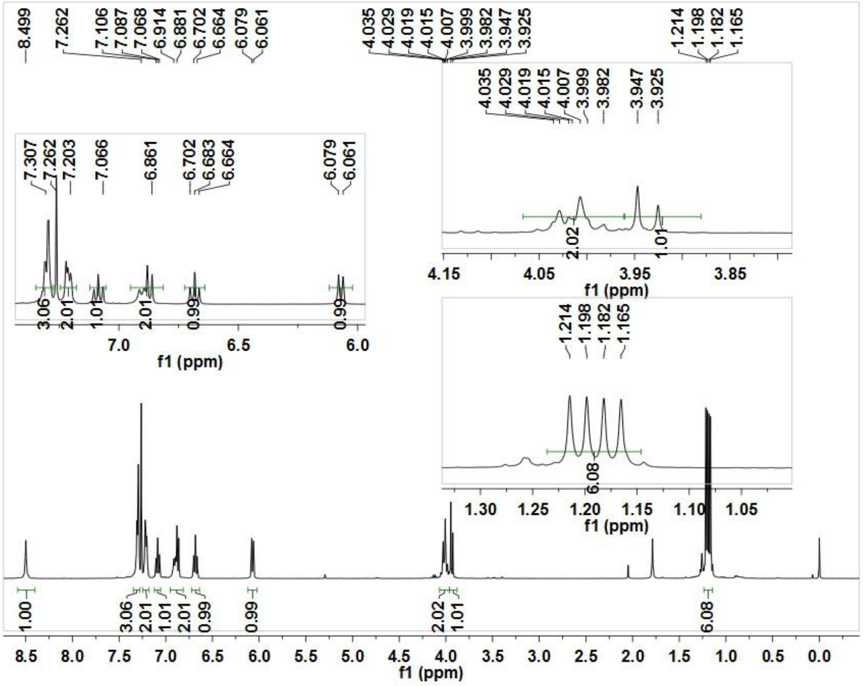

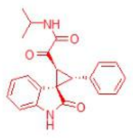

4aa

Current Data Parameters

F2 - Acquisition Parameters
DATE: 2015-04-02T01:09:52
PULPROG: zg30
TD: 32768
Solvent: CDCl3
NS: 64
DS: undefined
SWH: 8223.7 Hz
AQ: undefined
TE: 295.4 C

======== CHANNEL f1 ========
NUC1: 1H
P1: 9.93 usec
SFO1: undefined MHz

F2 - Processing Parameters
SI: 65536
DC: 0.05
LB: 0.30 Hz
First Point: 0.50
FT: Hyper Quadrature
Phase: Manual
Ph0: 94.99
Ph1: 16.29

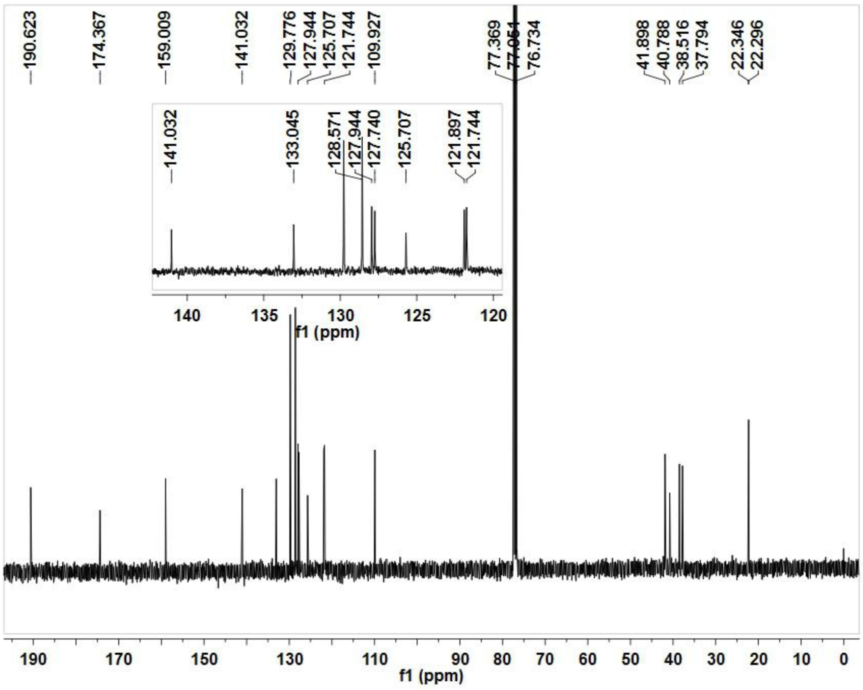

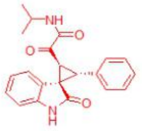

4aa

Current Data Parameters

F2 - Acquisition Parameters
DATE: 2015-04-02T01:18:15
PULPROG: zgpg30
TD: 32768
Solvent: CDCl3
NS: 128
DS: undefined
SWH: 24038.5 Hz
AQ: undefined
TE: 295.7 C

======== CHANNEL f1 ========
NUC1: 13C
P1: 9.63 usec
SFO1: undefined MHz

F2 - Processing Parameters
SI: 65536
DC: 0.05
LB: 1.00 Hz
First Point: 0.50
FT: Hyper Quadrature
Phase: Manual
Ph0: -96.01
Ph1: 103.51

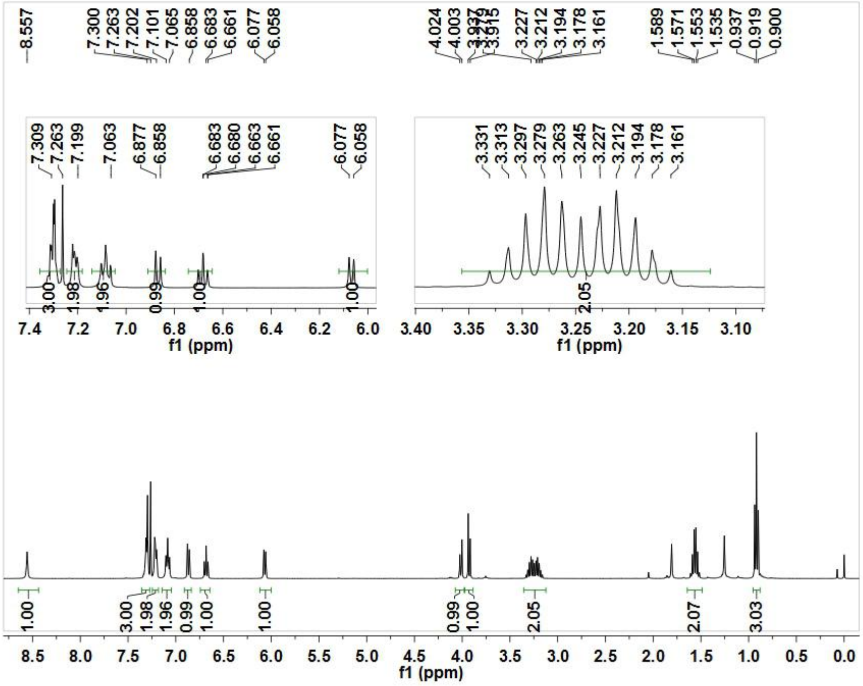

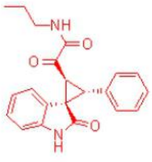

4ab

Current Data Parameters

F2 - Acquisition Parameters
DATE: 2015-04-03T22:14:29
PULPROG: zg30
TD: 32768
Solvent: CDCl3
NS: 16
DS: undefined
SWH: 8223.7 Hz
AQ: undefined
TE: 294 C

======== CHANNEL f1 ========
NUC1: 1H
P1: 9.93 usec
SFO1: undefined MHz

F2 - Processing Parameters
SI: 65536
DC: 0.05
LB: 0.30 Hz
First Point: 0.50
FT: Hyper Quadrature
Phase: Manual
Ph0: -262.16
Ph1: 17.77

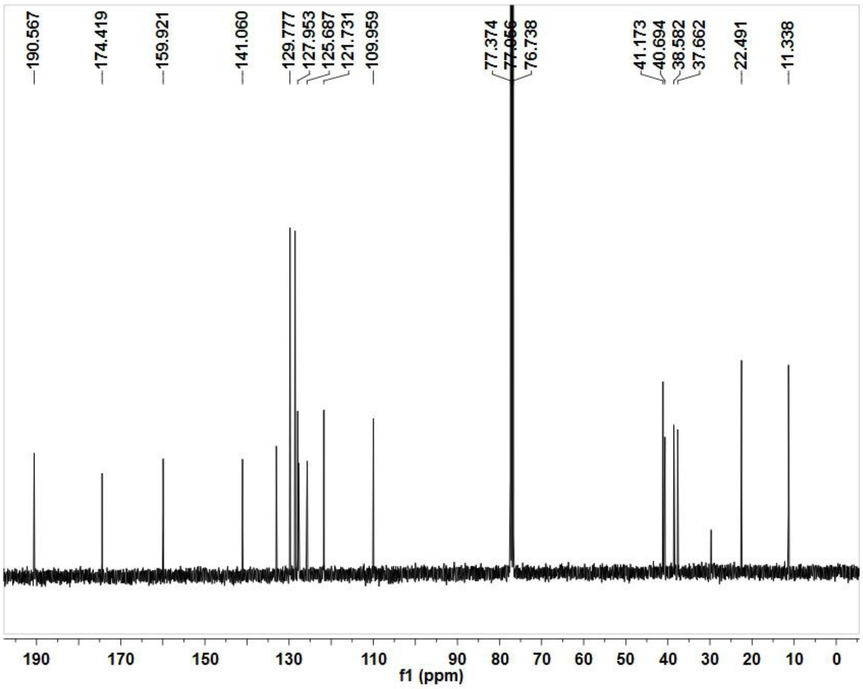

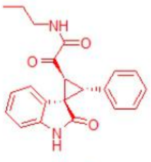

4aa'

Current Data Parameters

F2 - Acquisition Parameters
DATE: 2015-04-03T22:30:07
PULPROG: zgpg30
TD: 32768
Solvent: CDCl3
NS: 256
DS: undefined
SWH: 24038.5 Hz
AQ: undefined
TE: 294.4 C

======== CHANNEL f1 ========
NUC1: 13C
P1: 9.63 usec
SFO1: undefined MHz

F2 - Processing Parameters
SI: 65536
DC: 0.05
LB: 1.00 Hz
First Point: 0.50
FT: Hyper Quadrature
Phase: Manual
Ph0: -65.26
Ph1: 62.70

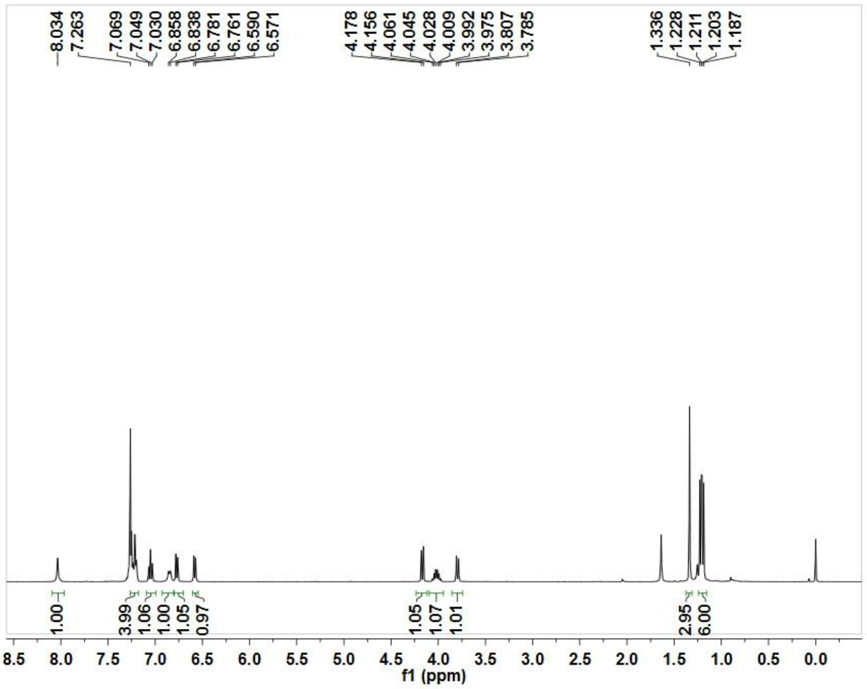

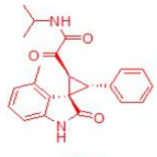

4ba

Current Data Parameters

F2 - Acquisition Parameters
DATE: 2015-02-25T22:58:01
PULPROG: zg30
TD: 32768
Solvent: CDCl3
NS: 128
DS: undefined
SWH: 8223.7 Hz
AQ: undefined
TE: 293.8 C

======== CHANNEL f1 ========
NUC1: 1H
P1: 9.93 usec
SFO1: undefined MHz

F2 - Processing Parameters
SI: 65536
DC: 0.05
LB: 0.30 Hz
First Point: 0.50
FT: Hyper Quadrature
Phase: Manual
Ph0: 95.09
Ph1: 17.05

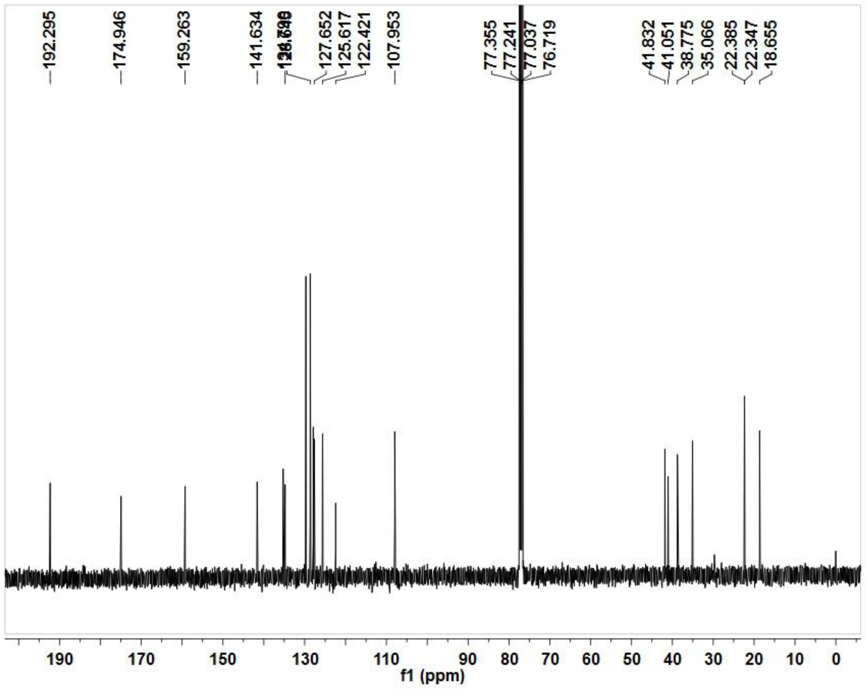

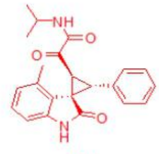

4ba

Current Data Parameters

F2 - Acquisition Parameters
DATE: 2015-02-25T23:57:27
PULPROG: zgpg30
TD: 32768
Solvent: CDCl3
NS: 1024
DS: undefined
SWH: 24038.5 Hz
AQ: undefined
TE: 294.1 C

======== CHANNEL f1 ========
NUC1: 13C
P1: 9.63 usec
SFO1: undefined MHz

F2 - Processing Parameters
SI: 65536
DC: 0.05
LB: 1.00 Hz
First Point: 0.50
FT: Hyper Quadrature
Phase: Manual
Ph0: -53.83
Ph1: 36.10

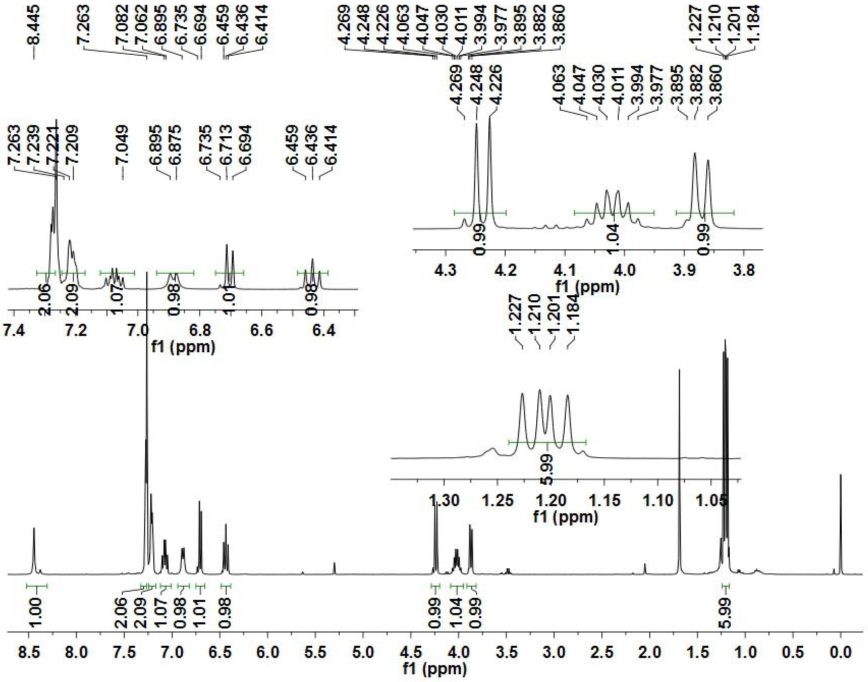

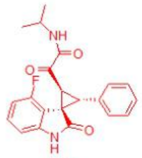

4ca

Current Data Parameters

F2 - Acquisition Parameters
DATE: 2015-01-29T02:21:53
PULPROG: zg30
TD: 32768
Solvent: CDCl3
NS: 64
DS: undefined
SWH: 8223.7 Hz
AQ: undefined
TE: 294.1 C

======== CHANNEL f1 ========
NUC1: 1H
P1: 9.93 usec
SFO1: undefined MHz

F2 - Processing Parameters
SI: 65536
DC: 0.05
LB: 0.30 Hz
First Point: 0.50
FT: Hyper Quadrature
Phase: Manual
Ph0: -267.64
Ph1: 18.03

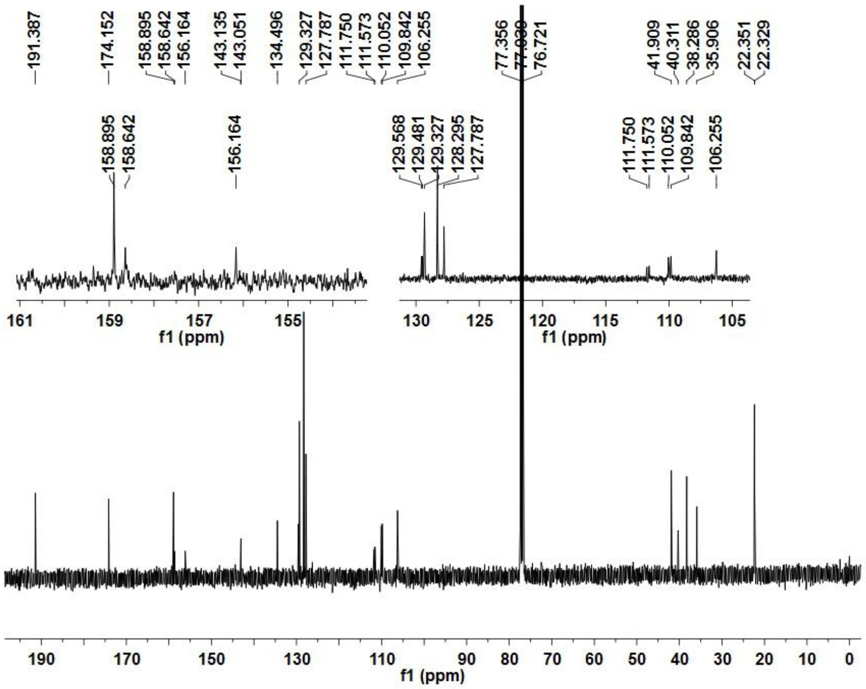

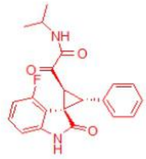

4ca

Current Data Parameters

F2 - Acquisition Parameters
DATE: 2015-01-29T02:52:05
PULPROG: zgpg30
TD: 32768
Solvent: CDCl3
NS: 512
DS: undefined
SWH: 24038.5 Hz
AQ: undefined
TE: 294.3 C

======== CHANNEL f1 ========
NUC1: 13C
P1: 9.63 usec
SFO1: undefined MHz

F2 - Processing Parameters
SI: 65536
DC: 0.05
LB: 1.00 Hz
First Point: 0.50
FT: Hyper Quadrature
Phase: Manual
Ph0: -61.49
Ph1: 58.44

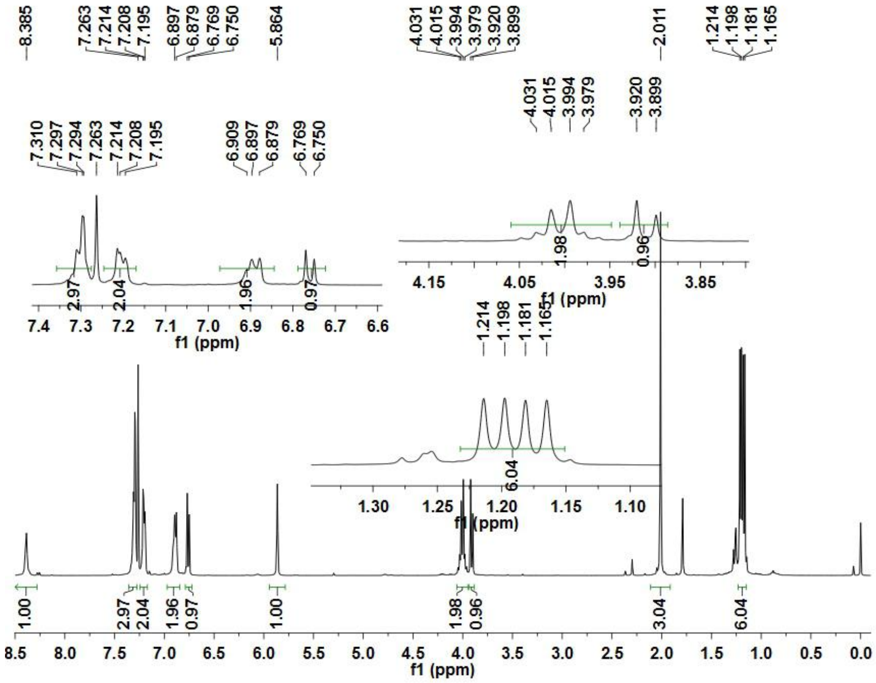

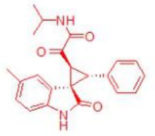

4da

Current Data Parameters

F2 - Acquisition Parameters
DATE: 2015-01-15T11:52:55
PULPROG: zg30
TD: 32768
Solvent: CDCl3
NS: 64
DS: undefined
SWH: 8223.7 Hz
AQ: undefined
TE: 293.6 C

======== CHANNEL f1 ========
NUC1: 1H
P1: 9.93 usec
SFO1: undefined MHz

F2 - Processing Parameters
SI: 65536
DC: 0.05
LB: 0.30 Hz
First Point: 0.50
FT: Hyper Quadrature
Phase: Manual
Ph0: 93.50
Ph1: 19.14

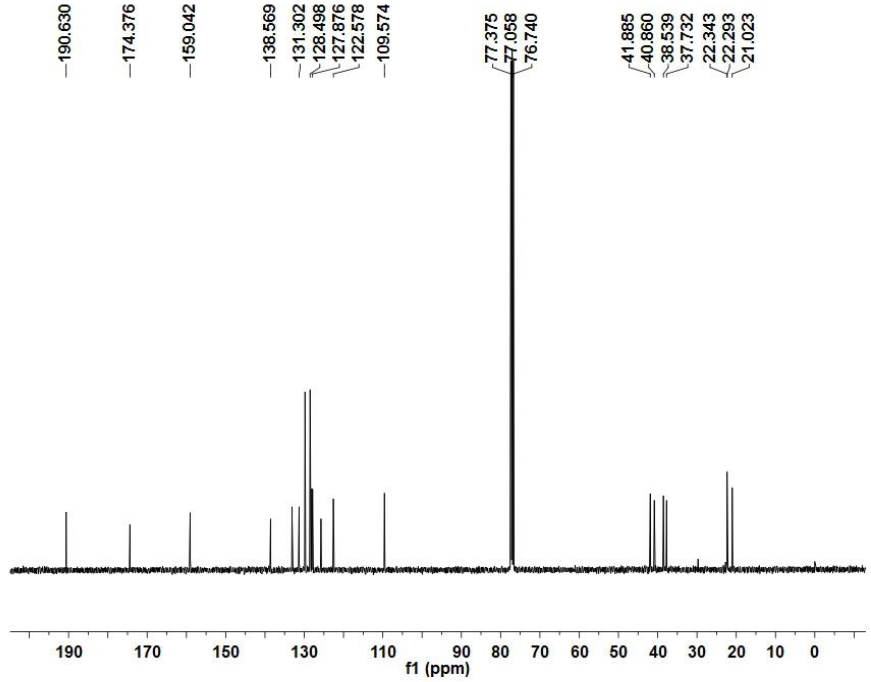

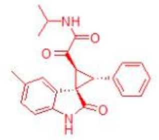

4da

Current Data Parameters

F2 - Acquisition Parameters
DATE: 2015-01-15T12:23:07
PULPROG: zgpg30
TD: 32768
Solvent: CDCl3
NS: 512
DS: undefined
SWH: 24038.5 Hz
AQ: undefined
TE: 294.2 C

======== CHANNEL f1 ========
NUC1: 13C
P1: 9.63 usec
SFO1: undefined MHz

F2 - Processing Parameters
SI: 65536
DC: 0.05
LB: 1.00 Hz
First Point: 0.50
FT: Hyper Quadrature
Phase: Manual
Ph0: -58.54
Ph1: 49.49

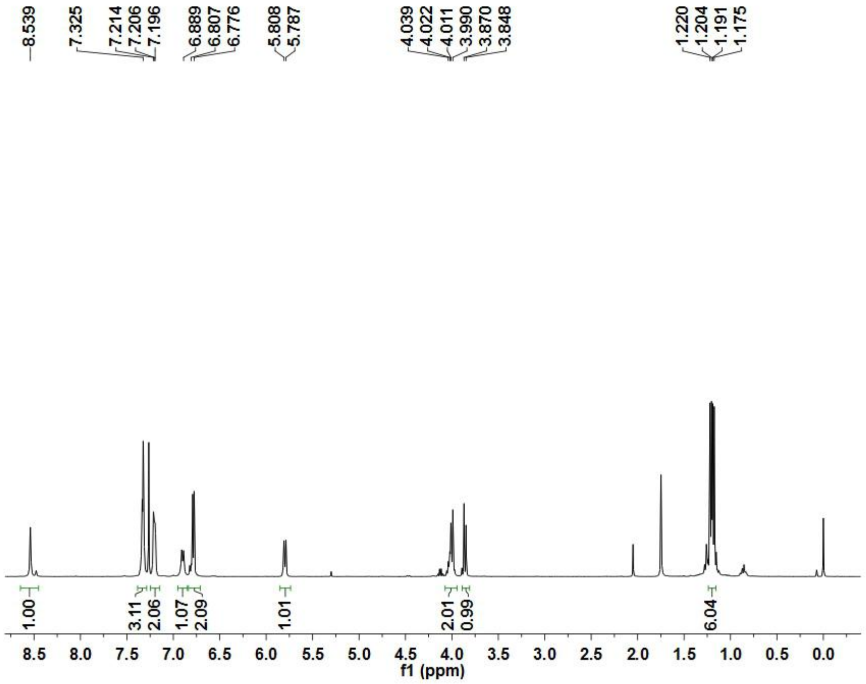

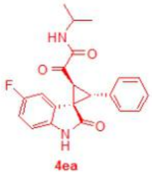

4ea

Current Data Parameters

F2 - Acquisition Parameters
DATE: 2015-01-29T06:58:14
PULPROG: zg30
TD: 32768
Solvent: CDCl3
NS: 64
DS: undefined
SWH: 8223.7 Hz
AQ: undefined
TE: 294.1 C

======== CHANNEL f1 ========
NUC1: 1H
P1: 9.93 usec
SFO1: undefined MHz

F2 - Processing Parameters
SI: 65536
DC: 0.05
LB: 0.30 Hz
First Point: 0.50
FT: Hyper Quadrature
Phase: Manual
Ph0: 92.24
Ph1: 18.80

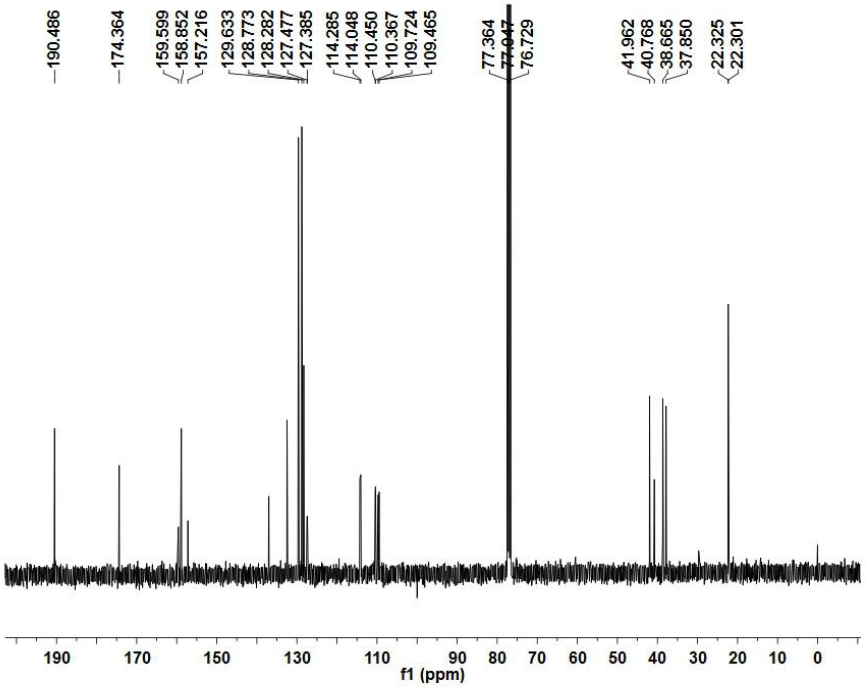

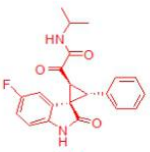

4ea

Current Data Parameters

F2 - Acquisition Parameters
DATE: 2015-01-29T07:28:25
PULPROG: zgpg30
TD: 32768
Solvent: CDCl3
NS: 512
DS: undefined
SWH: 24038.5 Hz
AQ: undefined
TE: 294.3 C

======== CHANNEL f1 ========
NUC1: 13C
P1: 9.63 usec
SFO1: undefined MHz

F2 - Processing Parameters
SI: 65536
DC: 0.05
LB: 1.00 Hz
First Point: 0.50
FT: Hyper Quadrature
Phase: Manual
Ph0: -53.58
Ph1: 41.31

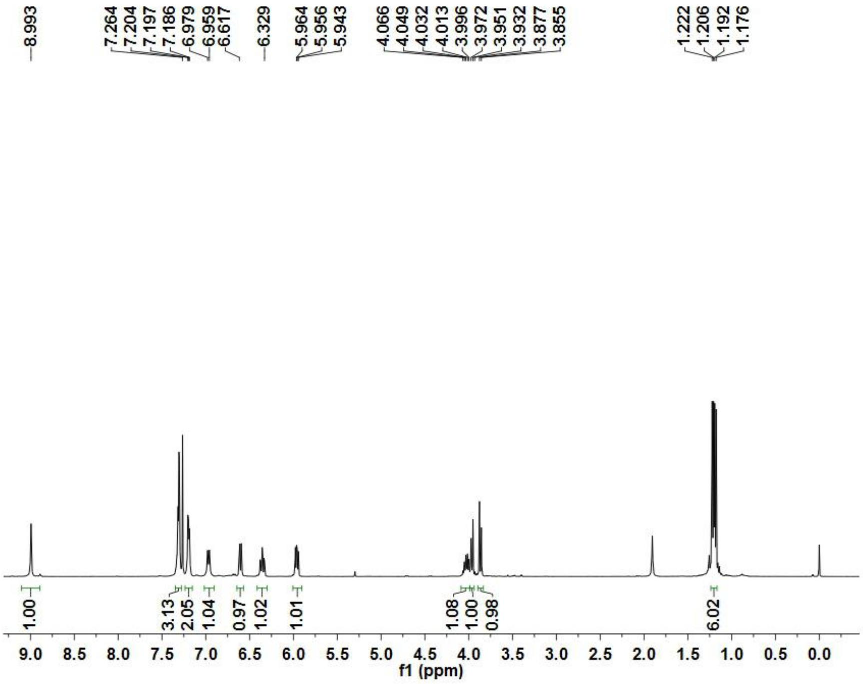

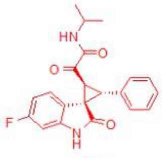

4ga

Current Data Parameters

F2 - Acquisition Parameters
DATE: 2015-01-14T04:52:38
PULPROG: zg30
TD: 32768
Solvent: CDCl3
NS: 64
DS: undefined
SWH: 8223.7 Hz
AQ: undefined
TE: 293.7 C

======== CHANNEL f1 ========
NUC1: 1H
P1: 9.93 usec
SFO1: undefined MHz

F2 - Processing Parameters
SI: 65536
DC: 0.05
LB: 0.30 Hz
First Point: 0.50
FT: Hyper Quadrature
Phase: Manual
Ph0: -264.29
Ph1: 19.74

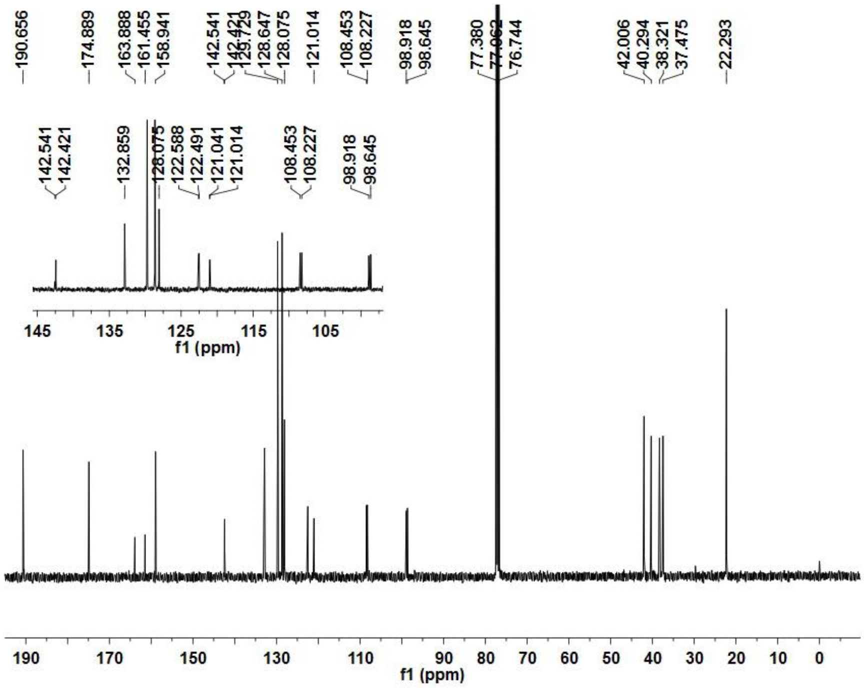

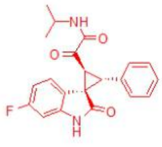

4ga

Current Data Parameters

F2 - Acquisition Parameters
DATE: 2015-01-14T05:22:49
PULPROG: zgpg30
TD: 32768
Solvent: CDCl3
NS: 512
DS: undefined
SWH: 24038.5 Hz
AQ: undefined
TE: 294.2 C

======== CHANNEL f1 ========
NUC1: 13C
P1: 9.63 usec
SFO1: undefined MHz

F2 - Processing Parameters
SI: 65536
DC: 0.05
LB: 1.00 Hz
First Point: 0.50
FT: Hyper Quadrature
Phase: Manual
Ph0: -60.14
Ph1: 60.48

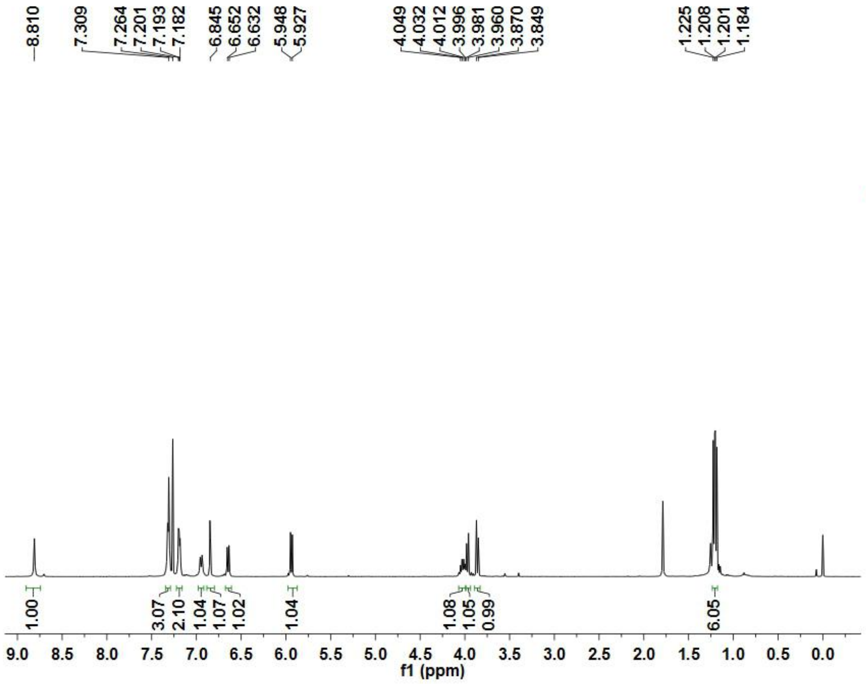

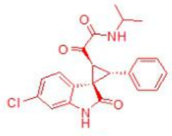

4ha

```
Current Data Parameters

F2 - Acquisition Parameters
DATE: 2015-01-15T10:17:08
PULPROG: zg30
TD: 32768
Solvent: CDCl3
NS: 64
DS: undefined
SWH: 8223.7 Hz
AQ: undefined
TE: 293.6 C

======== CHANNEL f1 ========
NUC1: 1H
P1: 9.93 usec
SFO1: undefined MHz

F2 - Processing Parameters
SI: 65536
DC: 0.05
LB: 0.30 Hz
First Point: 0.50
FT: Hyper Quadrature
Phase: Manual
Ph0: 94.32
Ph1: 19.06
```

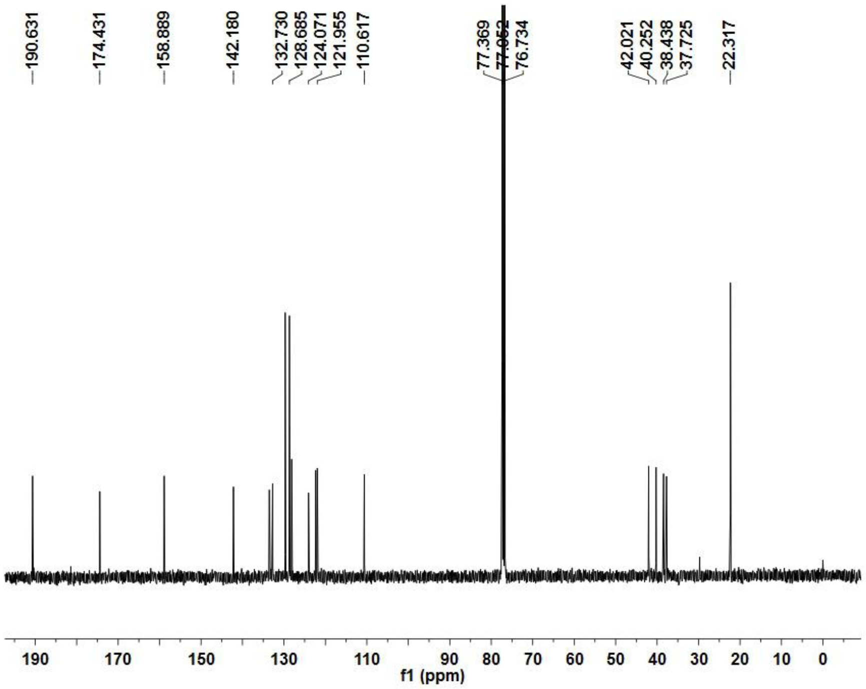

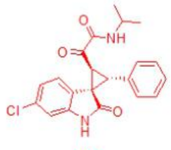

4ha

```
Current Data Parameters

F2 - Acquisition Parameters
DATE: 2015-01-15T10:47:30
PULPROG: zgpg30
TD: 32768
Solvent: CDCl3
NS: 512
DS: undefined
SWH: 24038.5 Hz
AQ: undefined
TE: 294 C

======== CHANNEL f1 ========
NUC1: 13C
P1: 9.63 usec
SFO1: undefined MHz

F2 - Processing Parameters
SI: 65536
DC: 0.05
LB: 1.00 Hz
First Point: 0.50
FT: Hyper Quadrature
Phase: Manual
Ph0: -69.13
Ph1: 67.55
```

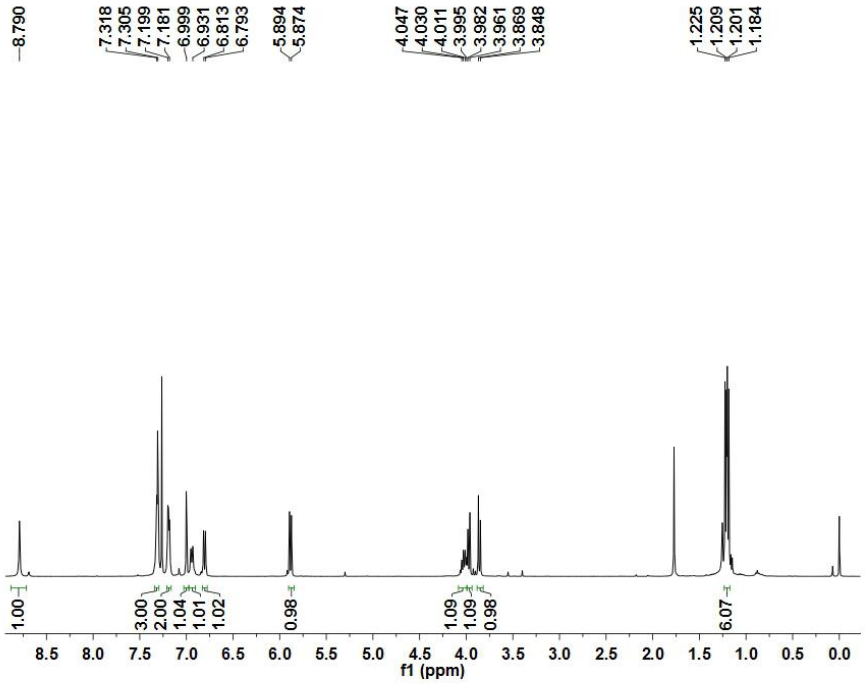

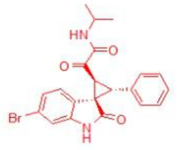

4ia

Current Data Parameters

F2 - Acquisition Parameters
DATE: 2015-01-15T16:11:31
PULPROG: zg30
TD: 32768
Solvent: CDCl3
NS: 64
DS: undefined
SWH: 8223.7 Hz
AQ: undefined
TE: 293.8 C

======== CHANNEL f1 ========
NUC1: 1H
P1: 9.93 usec
SFO1: undefined MHz

F2 - Processing Parameters
SI: 65536
DC: 0.05
LB: 0.30 Hz
First Point: 0.50
FT: Hyper Quadrature
Phase: Manual
Ph0: -267.13
Ph1: 17.79

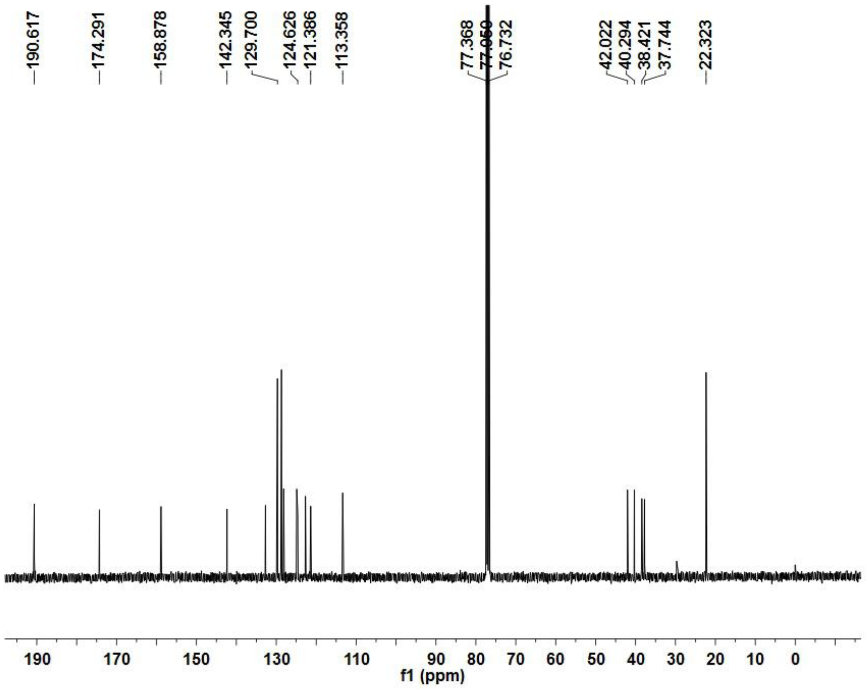

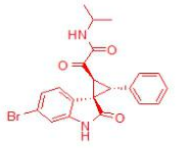

4ia

Current Data Parameters

F2 - Acquisition Parameters
DATE: 2015-01-15T16:41:42
PULPROG: zgpg30
TD: 32768
Solvent: CDCl3
NS: 512
DS: undefined
SWH: 24038.5 Hz
AQ: undefined
TE: 294.2 C

======== CHANNEL f1 ========
NUC1: 13C
P1: 9.63 usec
SFO1: undefined MHz

F2 - Processing Parameters
SI: 65536
DC: 0.05
LB: 1.00 Hz
First Point: 0.50
FT: Hyper Quadrature
Phase: Manual
Ph0: -52.50
Ph1: 39.06

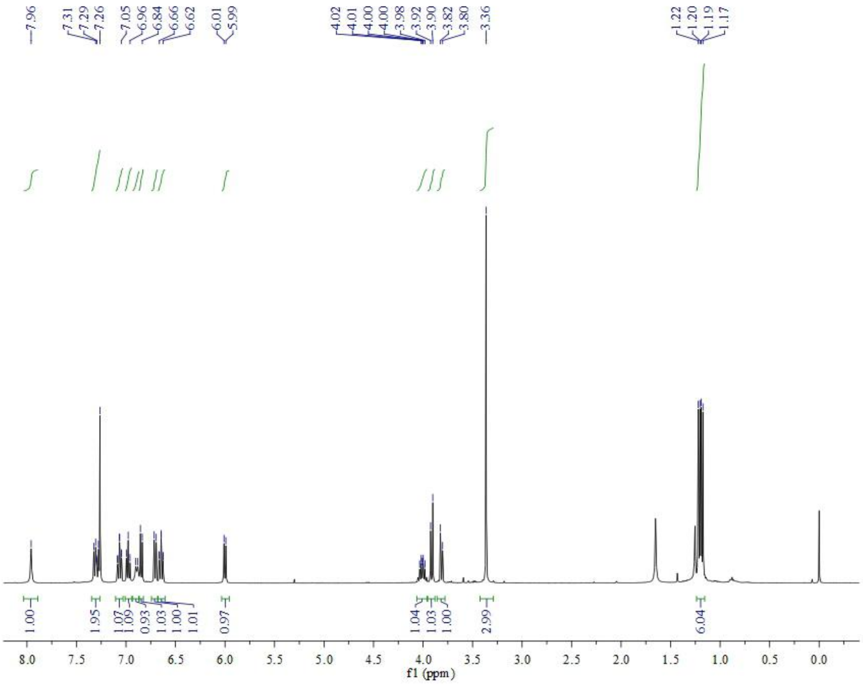

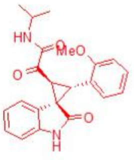

4ah

Current Data Parameters

F2 - Acquisition Parameters
DATE: 2015-04-28T17:37:01
PULPROG: zg30
TD: 32768
Solvent: CDCl3
NS: 64
DS: undefined
SWH: 8223.7 Hz
AQ: undefined
TE: 295.8 C

======== CHANNEL f1 ========
NUC1: 1H
P1: 9.93 usec
SFO1: undefined MHz

F2 - Processing Parameters
SI: 65536
DC: 0.05
LB: 0.30 Hz
First Point: 0.50
FT: Hyper Quadrature
Phase: Manual
Ph0: 92.60
Ph1: 12.98

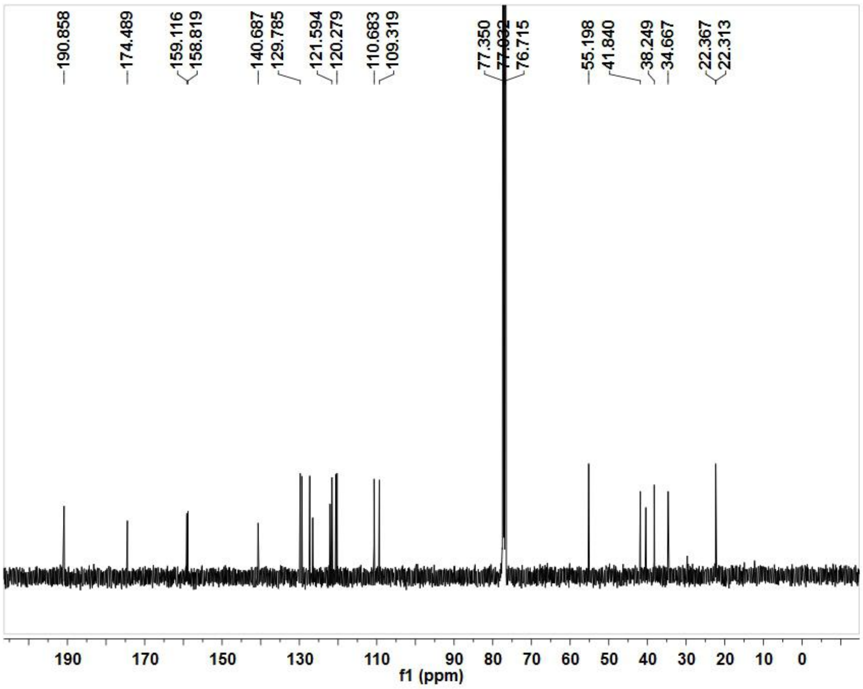

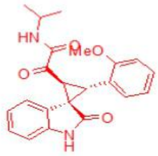

4ah

Current Data Parameters

F2 - Acquisition Parameters
DATE: 2015-04-28T18:07:15
PULPROG: zgpg30
TD: 32768
Solvent: CDCl3
NS: 512
DS: undefined
SWH: 24038.5 Hz
AQ: undefined
TE: 296.2 C

======== CHANNEL f1 ========
NUC1: 13C
P1: 9.63 usec
SFO1: undefined MHz

F2 - Processing Parameters
SI: 65536
DC: 0.05
LB: 1.00 Hz
First Point: 0.50
FT: Hyper Quadrature
Phase: Manual
Ph0: -55.04
Ph1: 41.14

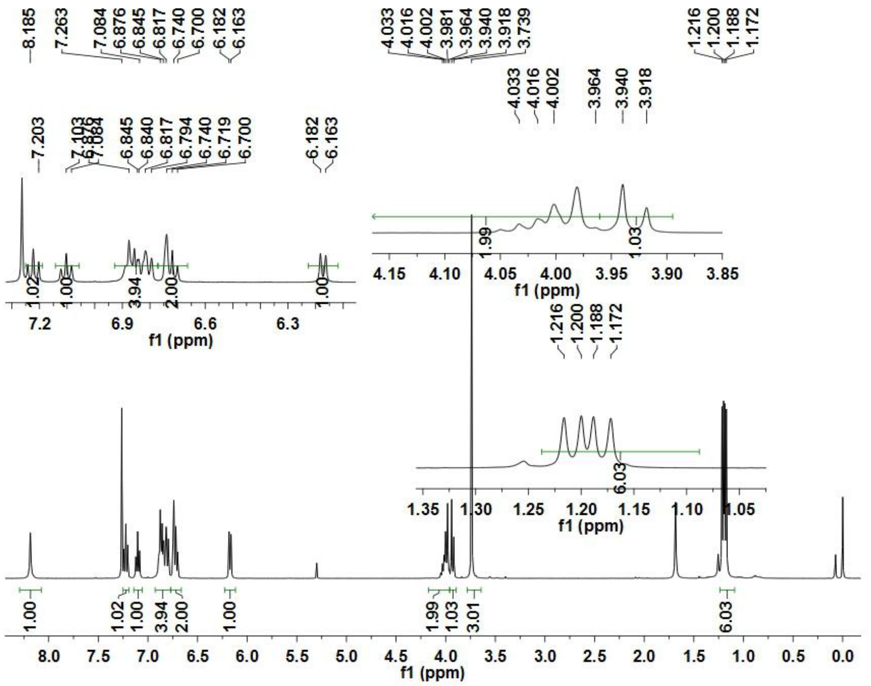

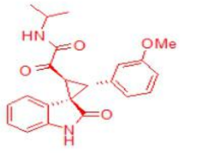

4ai

Current Data Parameters

F2 - Acquisition Parameters
DATE: 2015-01-16T03:06:48
PULPROG: zg30
TD: 32768
Solvent: CDCl3
NS: 64
DS: undefined
SWH: 8223.7 Hz
AQ: undefined
TE: 294.5 C

======== CHANNEL f1 ========
NUC1: 1H
P1: 9.93 usec
SFO1: undefined MHz

F2 - Processing Parameters
SI: 65536
DC: 0.05
LB: 0.30 Hz
First Point: 0.50
FT: Hyper Quadrature
Phase: Manual
Ph0: 92.31
Ph1: 17.61

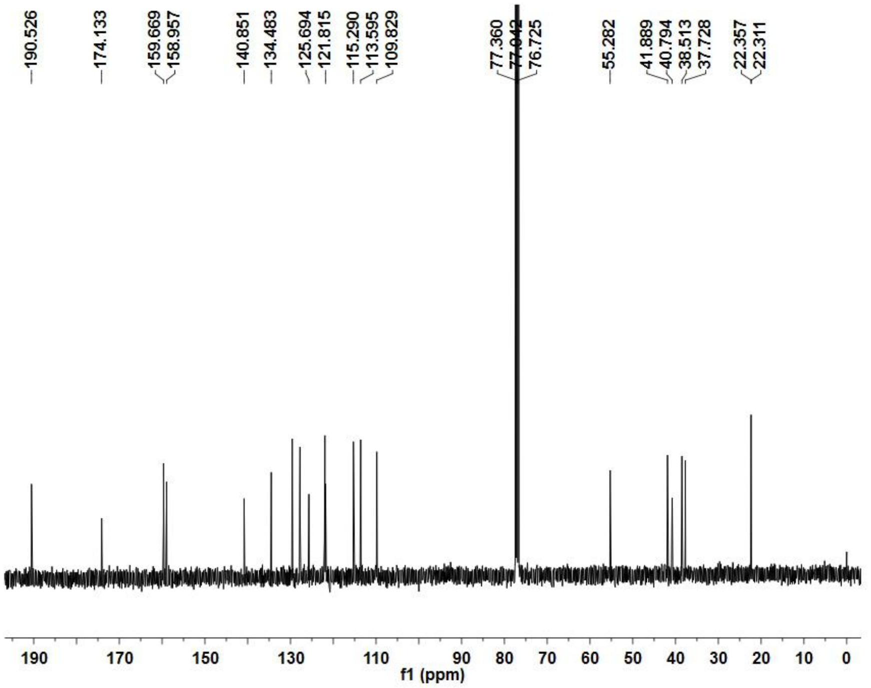

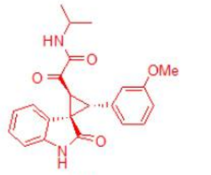

4ai

Current Data Parameters

F2 - Acquisition Parameters
DATE: 2015-01-16T03:37:00
PULPROG: zgpg30
TD: 32768
Solvent: CDCl3
NS: 512
DS: undefined
SWH: 24038.5 Hz
AQ: undefined
TE: 294.8 C

======== CHANNEL f1 ========
NUC1: 13C
P1: 9.63 usec
SFO1: undefined MHz

F2 - Processing Parameters
SI: 65536
DC: 0.05
LB: 1.00 Hz
First Point: 0.50
FT: Hyper Quadrature
Phase: Manual
Ph0: -69.63
Ph1: 68.93

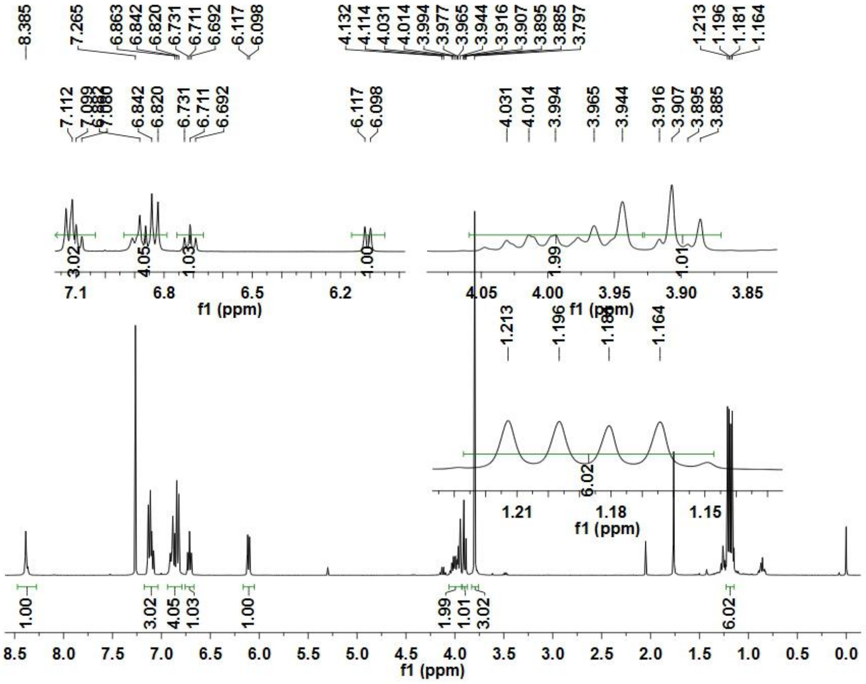

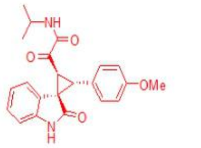

4aj

Current Data Parameters

F2 - Acquisition Parameters
DATE: 2015-01-29T03:38:32
PULPROG: zg30
TD: 32768
Solvent: CDCl3
NS: 64
DS: undefined
SWH: 8223.7 Hz
AQ: undefined
TE: 293.9 C

======== CHANNEL f1 ========
NUC1: 1H
P1: 9.93 usec
SFO1: undefined MHz

F2 - Processing Parameters
SI: 65536
DC: 0.05
LB: 0.30 Hz
First Point: 0.50
FT: Hyper Quadrature
Phase: Manual
Ph0: -265.60
Ph1: 17.71

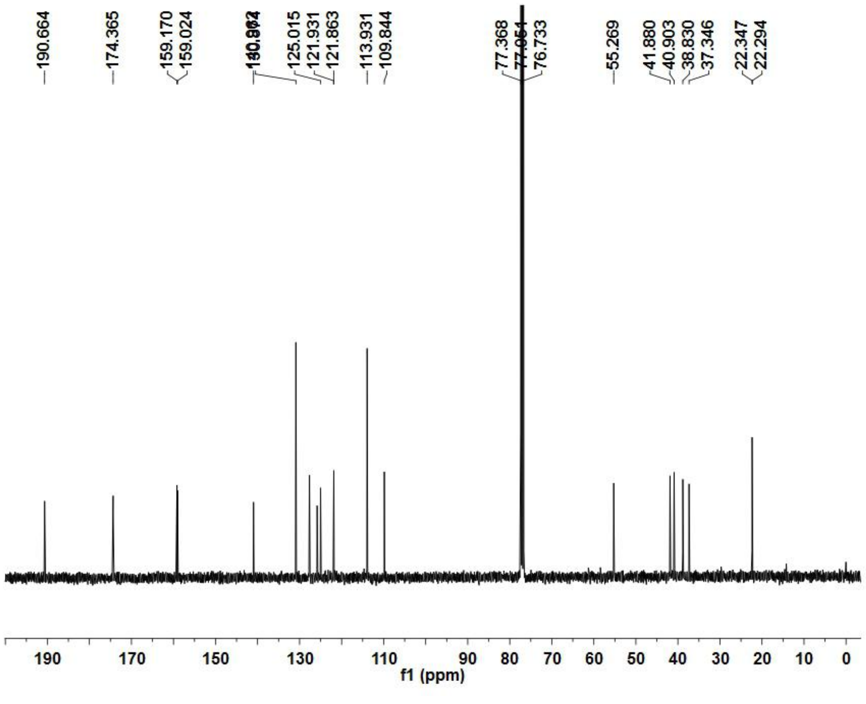

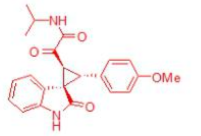

4aj

Current Data Parameters

F2 - Acquisition Parameters
DATE: 2015-01-29T04:08:44
PULPROG: zgpg30
TD: 32768
Solvent: CDCl3
NS: 512
DS: undefined
SWH: 24038.5 Hz
AQ: undefined
TE: 294.3 C

======== CHANNEL f1 ========
NUC1: 13C
P1: 9.63 usec
SFO1: undefined MHz

F2 - Processing Parameters
SI: 65536
DC: 0.05
LB: 1.00 Hz
First Point: 0.50
FT: Hyper Quadrature
Phase: Manual
Ph0: -59.45
Ph1: 60.22

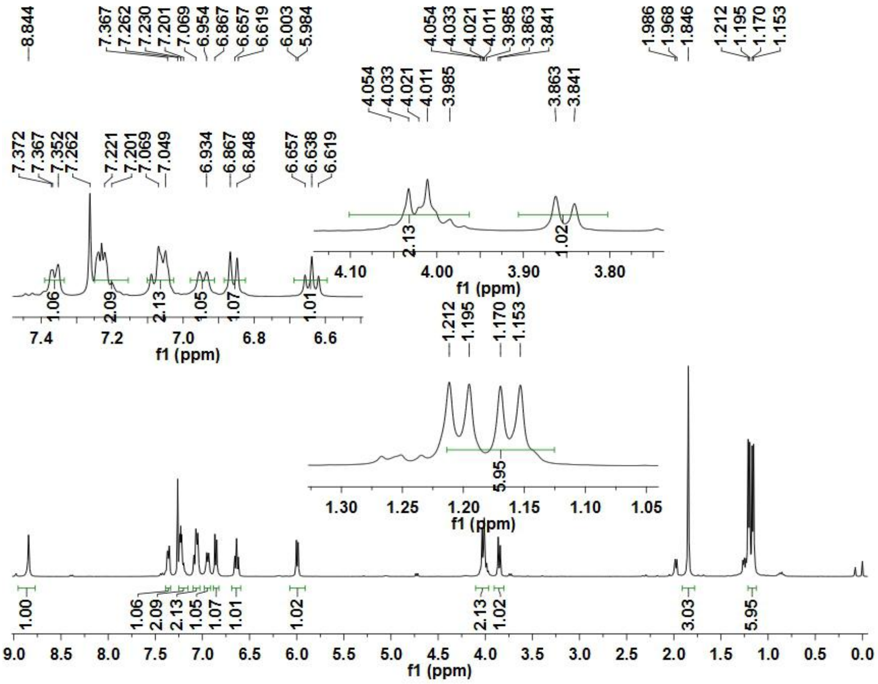

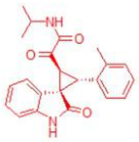

4ak

Current Data Parameters

F2 - Acquisition Parameters
DATE: 2015-01-16T22:53:21
PULPROG: zg30
TD: 32768
Solvent: CDCl3
NS: 64
DS: undefined
SWH: 8223.7 Hz
AQ: undefined
TE: 293.6 C

======== CHANNEL f1 ========
NUC1: 1H
P1: 9.93 usec
SFO1: undefined MHz

F2 - Processing Parameters
SI: 65536
DC: 0.05
LB: 0.30 Hz
First Point: 0.50
FT: Hyper Quadrature
Phase: Manual
Ph0: -264.97
Ph1: 19.49

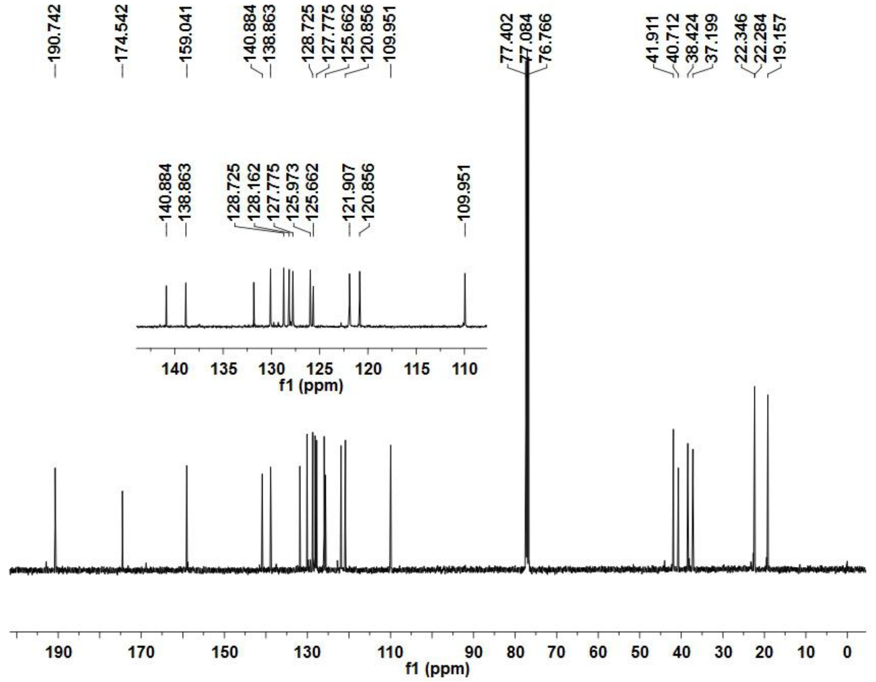

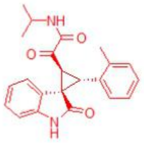

4ak

Current Data Parameters

F2 - Acquisition Parameters
DATE: 2015-01-16T23:23:33
PULPROG: zgpg30
TD: 32768
Solvent: CDCl3
NS: 512
DS: undefined
SWH: 24038.5 Hz
AQ: undefined
TE: 294 C

======== CHANNEL f1 ========
NUC1: 13C
P1: 9.63 usec
SFO1: undefined MHz

F2 - Processing Parameters
SI: 65536
DC: 0.05
LB: 1.00 Hz
First Point: 0.50
FT: Hyper Quadrature
Phase: Manual
Ph0: -68.53
Ph1: 71.96

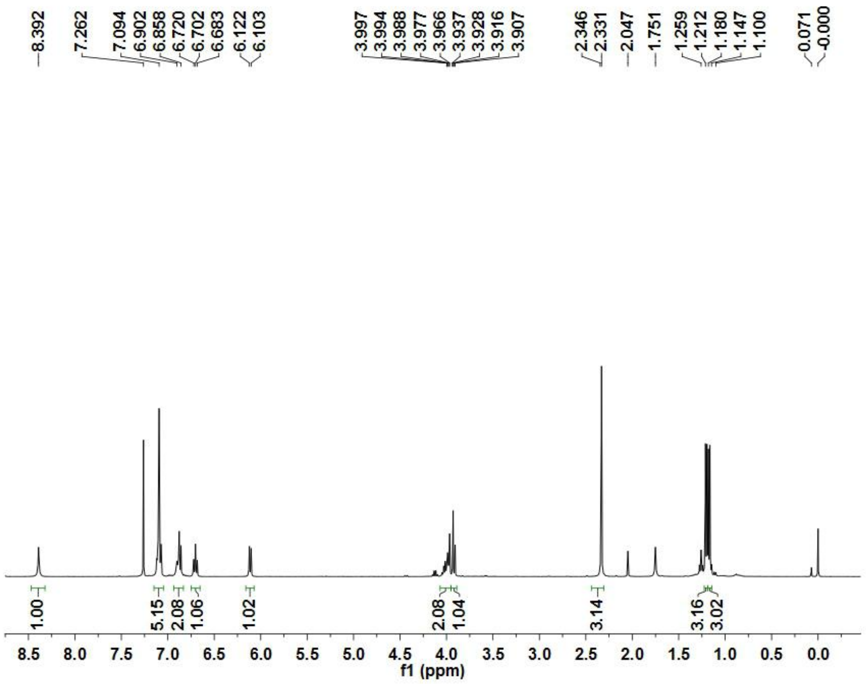

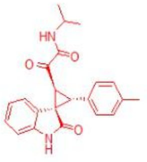

4al

Current Data Parameters

F2 - Acquisition Parameters
DATE: 2014-11-26T16:29:43
PULPROG: zg30
TD: 32768
Solvent: CDCl3
NS: 64
DS: undefined
SWH: 8223.7 Hz
AQ: undefined
TE: 295.8 C

======== CHANNEL f1 ========
NUC1: 1H
P1: 9.93 usec
SFO1: undefined MHz

F2 - Processing Parameters
SI: 65536
DC: 0.05
LB: 0.30 Hz
First Point: 0.50
FT: Hyper Quadrature
Phase: Manual
Ph0: 89.73
Ph1: 17.78

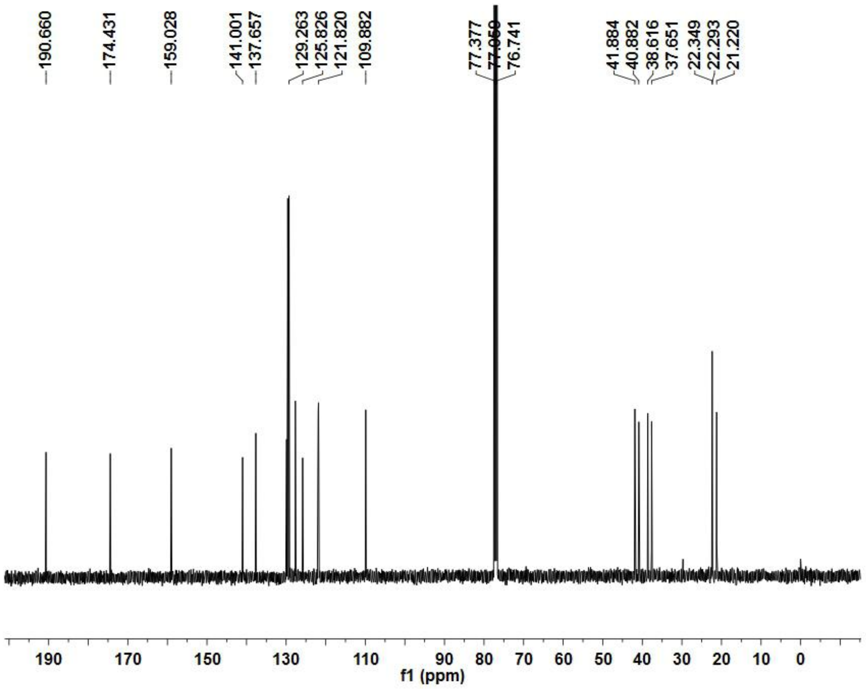

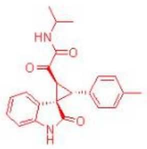

4al

Current Data Parameters

F2 - Acquisition Parameters
DATE: 2015-01-14T03:28:00
PULPROG: zgpg30
TD: 32768
Solvent: CDCl3
NS: 512
DS: undefined
SWH: 24038.5 Hz
AQ: undefined
TE: 294.1 C

======== CHANNEL f1 ========
NUC1: 13C
P1: 9.63 usec
SFO1: undefined MHz

F2 - Processing Parameters
SI: 65536
DC: 0.05
LB: 1.00 Hz
First Point: 0.50
FT: Hyper Quadrature
Phase: Manual
Ph0: -72.39
Ph1: 66.45

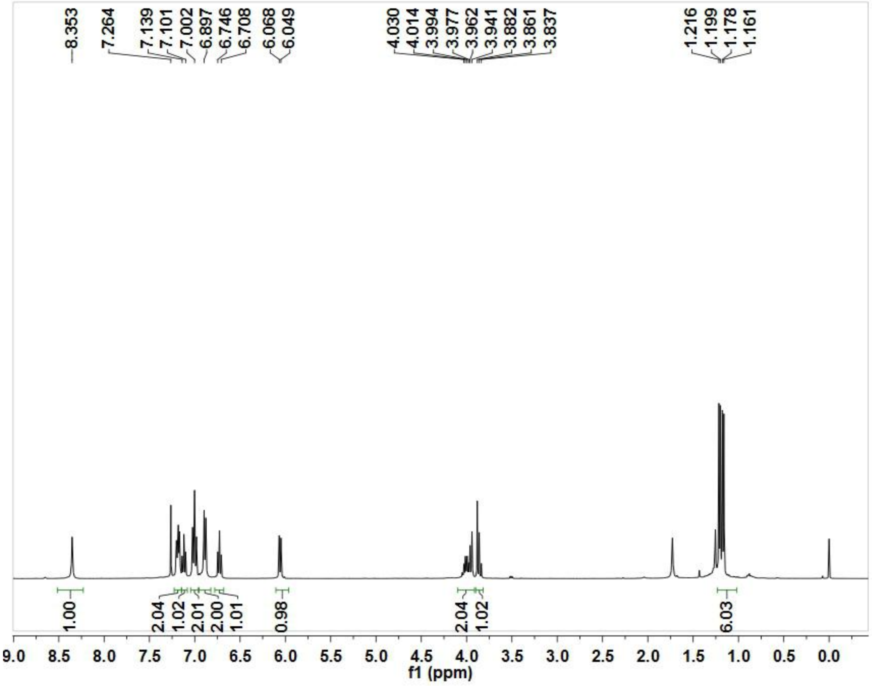

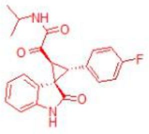

4av

```
Current Data Parameters

F2 - Acquisition Parameters
DATE: 2015-04-28T18:15:18
PULPROG: zg30
TD: 32768
Solvent: CDCl3
NS: 64
DS: undefined
SWH: 8223.7 Hz
AQ: undefined
TE: 295.8 C

======== CHANNEL f1 ========
NUC1: 1H
P1: 9.93 usec
SFO1: undefined MHz

F2 - Processing Parameters
SI: 65536
DC: 0.05
LB: 0.30 Hz
First Point: 0.50
FT: Hyper Quadrature
Phase: Manual
Ph0: 91.37
Ph1: 16.19
```

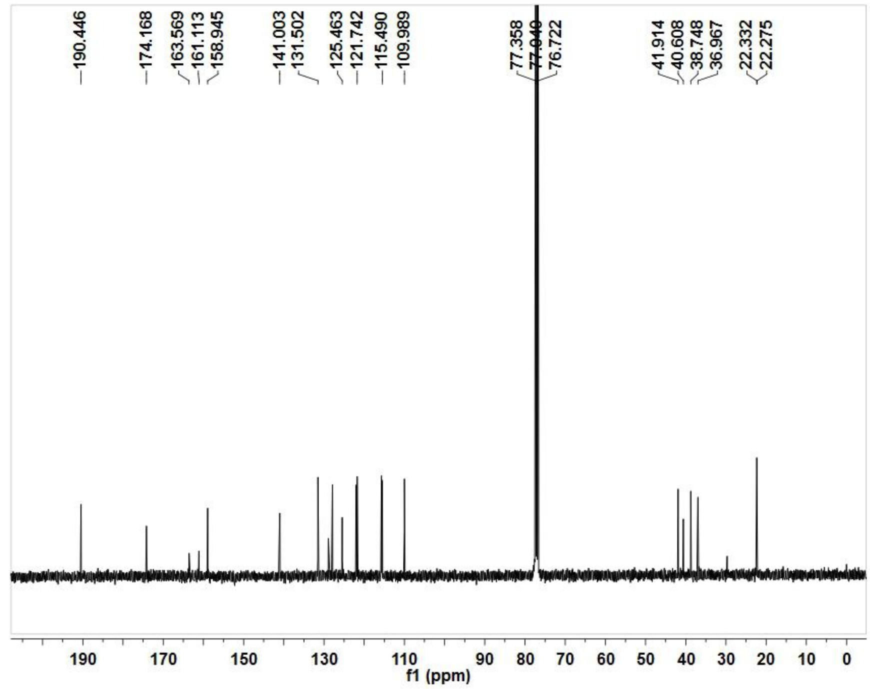

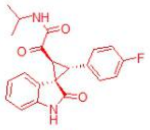

4av

```
Current Data Parameters

F2 - Acquisition Parameters
DATE: 2015-04-28T18:45:30
PULPROG: zgpg30
TD: 32768
Solvent: CDCl3
NS: 512
DS: undefined
SWH: 24038.5 Hz
AQ: undefined
TE: 296.1 C

======== CHANNEL f1 ========
NUC1: 13C
P1: 9.63 usec
SFO1: undefined MHz

F2 - Processing Parameters
SI: 65536
DC: 0.05
LB: 1.00 Hz
First Point: 0.50
FT: Hyper Quadrature
Phase: Manual
Ph0: -61.91
Ph1: 58.39
```

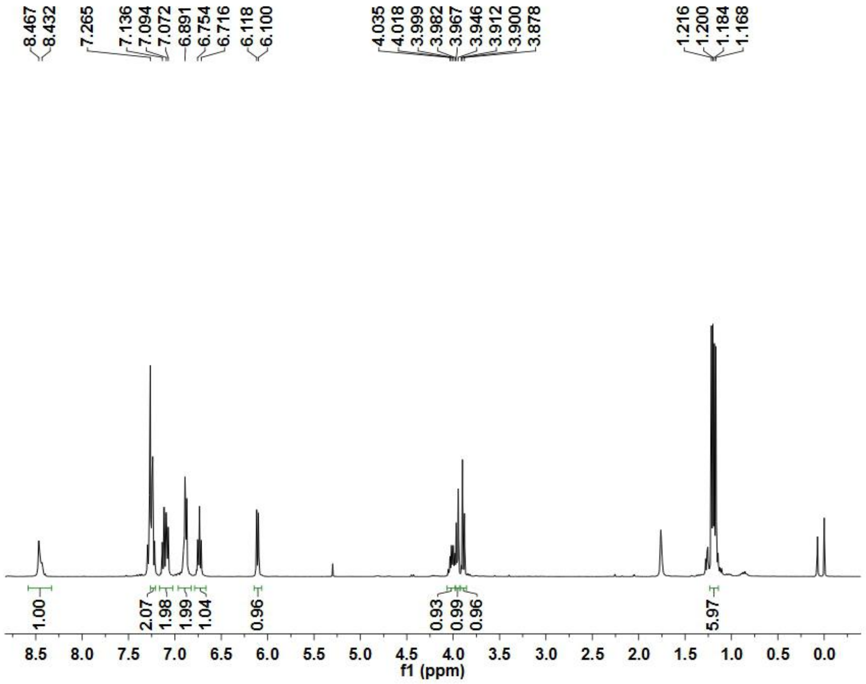

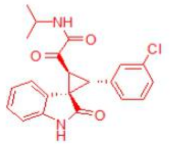

4am

Current Data Parameters

F2 - Acquisition Parameters
DATE: 2015-01-17T00:49:09
PULPROG: zg30
TD: 32768
Solvent: CDCl3
NS: 64
DS: undefined
SWH: 8223.7 Hz
AQ: undefined
TE: 293.5 C

======== CHANNEL f1 ========
NUC1: 1H
P1: 9.93 usec
SFO1: undefined MHz

F2 - Processing Parameters
SI: 65536
DC: 0.05
LB: 0.30 Hz
First Point: 0.50
FT: Hyper Quadrature
Phase: Manual
Ph0: -263.61
Ph1: 15.16

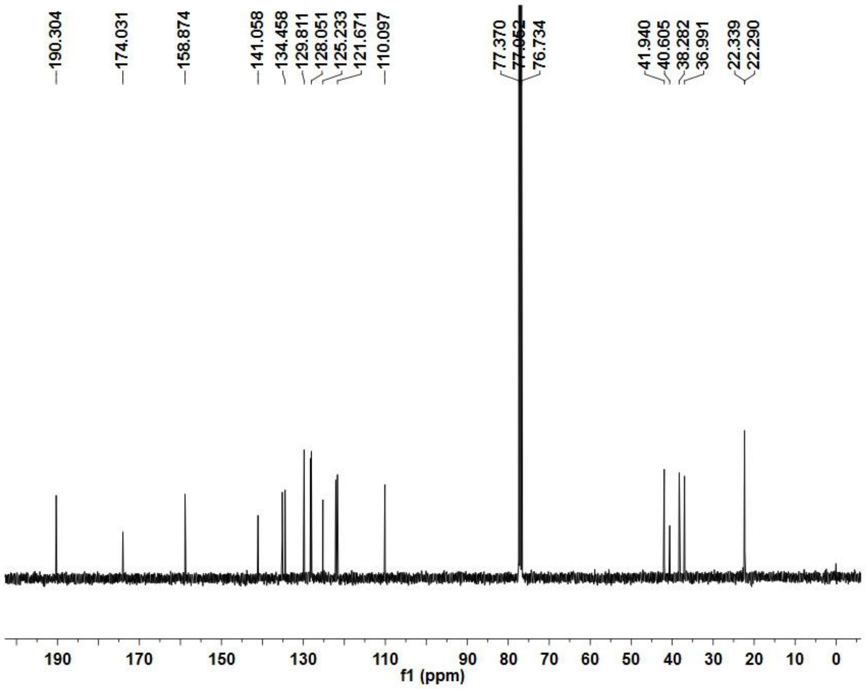

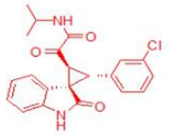

4am

Current Data Parameters

F2 - Acquisition Parameters
DATE: 2015-01-17T01:19:21
PULPROG: zgpg30
TD: 32768
Solvent: CDCl3
NS: 512
DS: undefined
SWH: 24038.5 Hz
AQ: undefined
TE: 294.1 C

======== CHANNEL f1 ========
NUC1: 13C
P1: 9.63 usec
SFO1: undefined MHz

F2 - Processing Parameters
SI: 65536
DC: 0.05
LB: 1.00 Hz
First Point: 0.50
FT: Hyper Quadrature
Phase: Manual
Ph0: -62.21
Ph1: 66.47

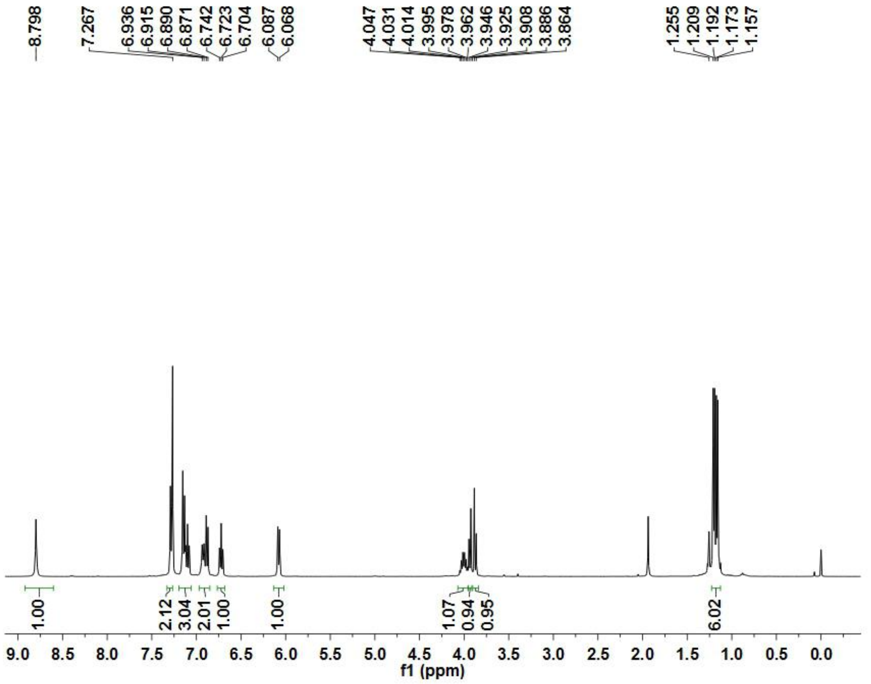

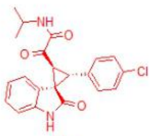

4an

Current Data Parameters

F2 - Acquisition Parameters
DATE: 2015-01-15T11:14:42
PULPROG: zg30
TD: 32768
Solvent: CDCl3
NS: 64
DS: undefined
SWH: 8223.7 Hz
AQ: undefined
TE: 293.5 C

======== CHANNEL f1 ========
NUC1: 1H
P1: 9.93 usec
SFO1: undefined MHz

F2 - Processing Parameters
SI: 65536
DC: 0.05
LB: 0.30 Hz
First Point: 0.50
FT: Hyper Quadrature
Phase: Manual
Ph0: 94.32
Ph1: 20.69

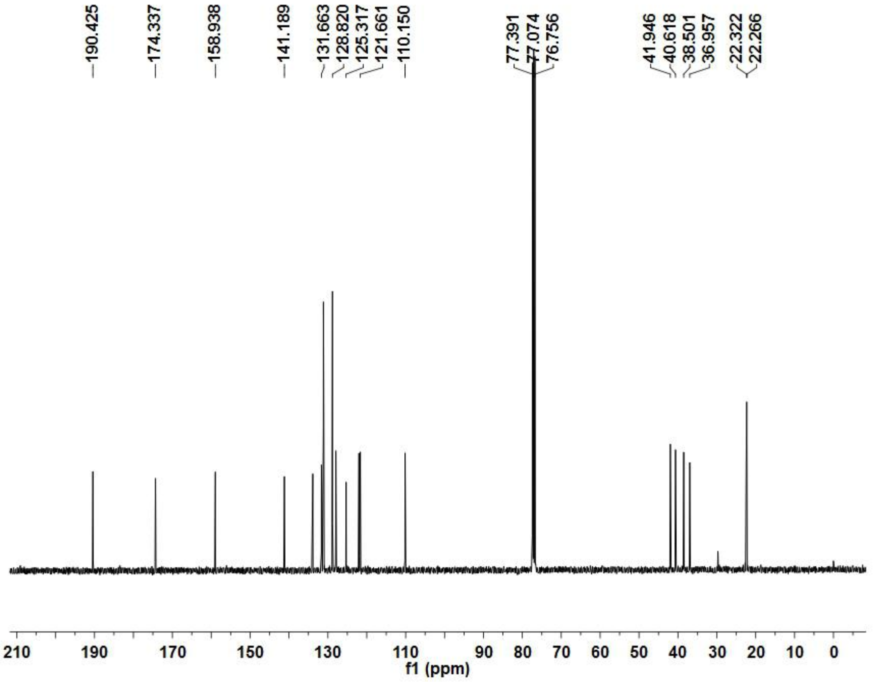

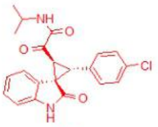

4an

Current Data Parameters

F2 - Acquisition Parameters
DATE: 2015-01-15T11:44:54
PULPROG: zgpg30
TD: 32768
Solvent: CDCl3
NS: 512
DS: undefined
SWH: 24038.5 Hz
AQ: undefined
TE: 294.2 C

======== CHANNEL f1 ========
NUC1: 13C
P1: 9.63 usec
SFO1: undefined MHz

F2 - Processing Parameters
SI: 65536
DC: 0.05
LB: 1.00 Hz
First Point: 0.50
FT: Hyper Quadrature
Phase: Manual
Ph0: -64.42
Ph1: 64.86

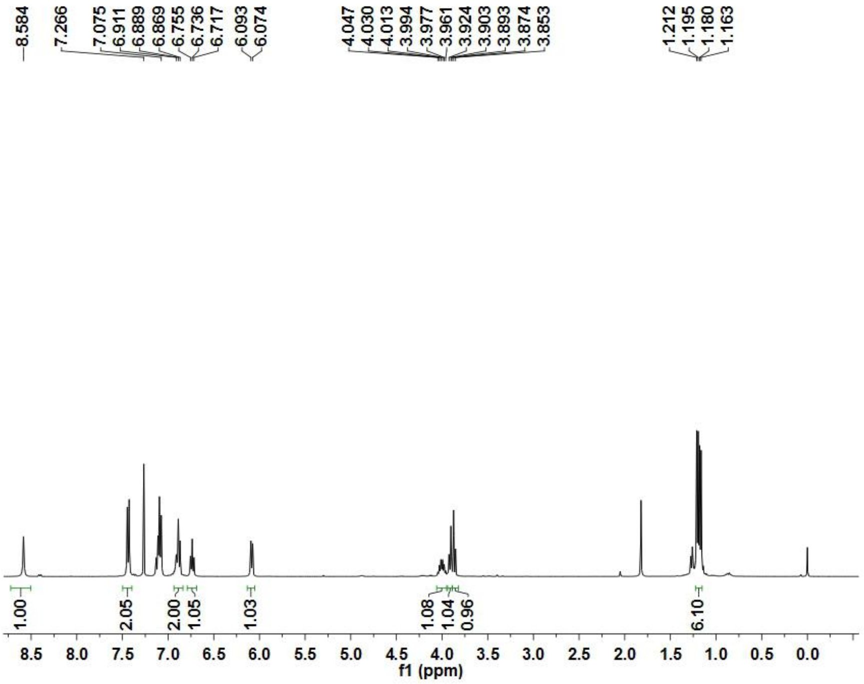

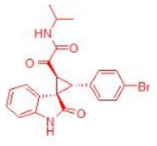

4ap

Current Data Parameters

F2 - Acquisition Parameters
DATE: 2015-01-14T02:19:34
PULPROG: zg30
TD: 32768
Solvent: CDCl3
NS: 64
DS: undefined
SWH: 8223.7 Hz
AQ: undefined
TE: 293.9 C

======== CHANNEL f1 ========
NUC1: 1H
P1: 9.93 usec
SFO1: undefined MHz

F2 - Processing Parameters
SI: 65536
DC: 0.05
LB: 0.30 Hz
First Point: 0.50
FT: Hyper Quadrature
Phase: Manual
Ph0: 93.74
Ph1: 20.44

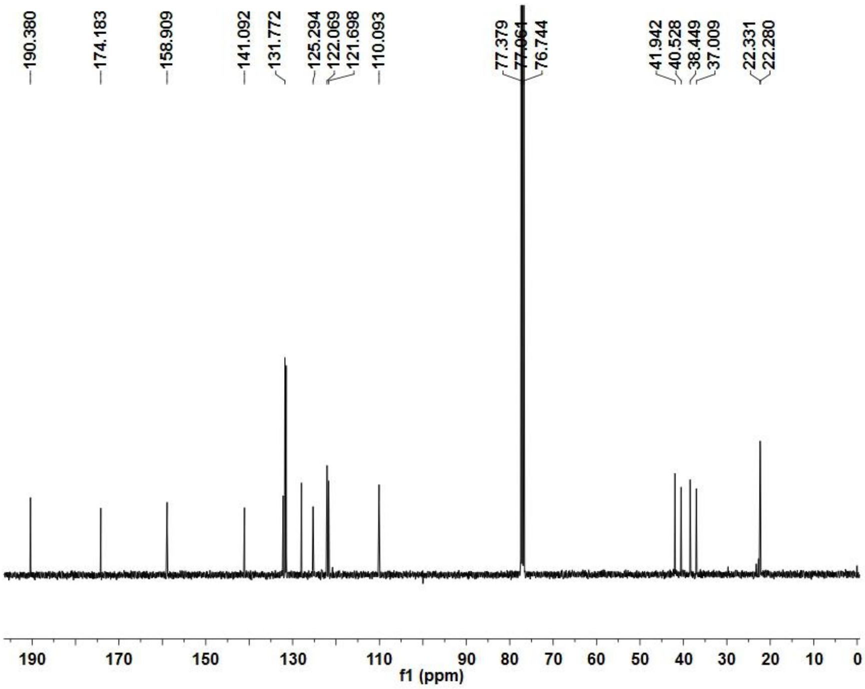

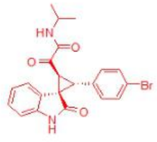

4ap

Current Data Parameters

F2 - Acquisition Parameters
DATE: 2015-01-14T02:49:45
PULPROG: zgpg30
TD: 32768
Solvent: CDCl3
NS: 512
DS: undefined
SWH: 24038.5 Hz
AQ: undefined
TE: 294.2 C

======== CHANNEL f1 ========
NUC1: 13C
P1: 9.63 usec
SFO1: undefined MHz

F2 - Processing Parameters
SI: 65536
DC: 0.05
LB: 1.00 Hz
First Point: 0.50
FT: Hyper Quadrature
Phase: Manual
Ph0: -64.40
Ph1: 56.36

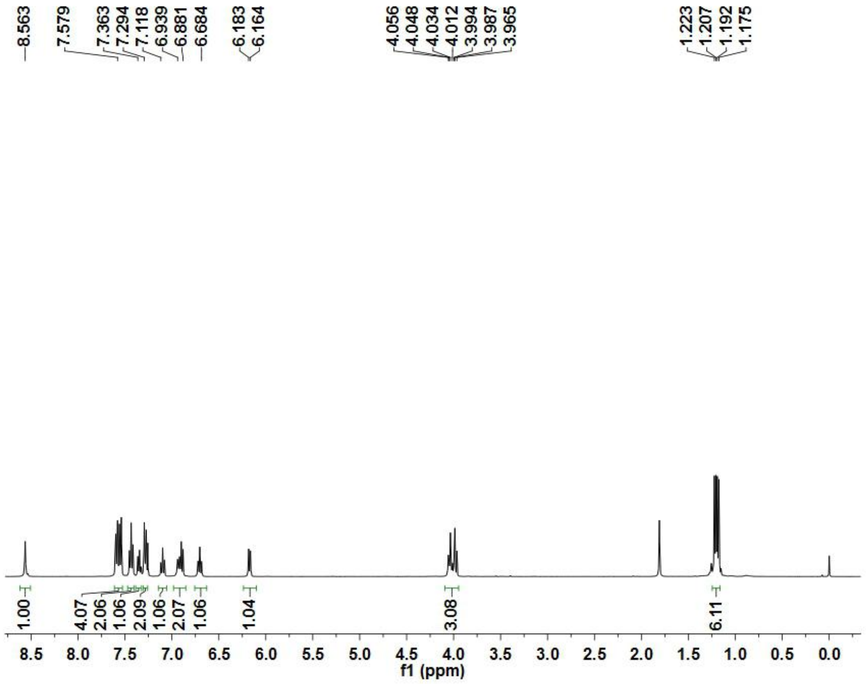

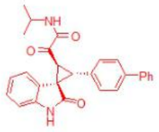

4aq

Current Data Parameters

F2 - Acquisition Parameters
DATE: 2015-01-14T01:41:21
PULPROG: zg30
TD: 32768
Solvent: CDCl3
NS: 64
DS: undefined
SWH: 8223.7 Hz
AQ: undefined
TE: 293.7 C

======== CHANNEL f1 ========
NUC1: 1H
P1: 9.93 usec
SFO1: undefined MHz

F2 - Processing Parameters
SI: 65536
DC: 0.05
LB: 0.30 Hz
First Point: 0.50
FT: Hyper Quadrature
Phase: Manual
Ph0: -264.15
Ph1: 19.97

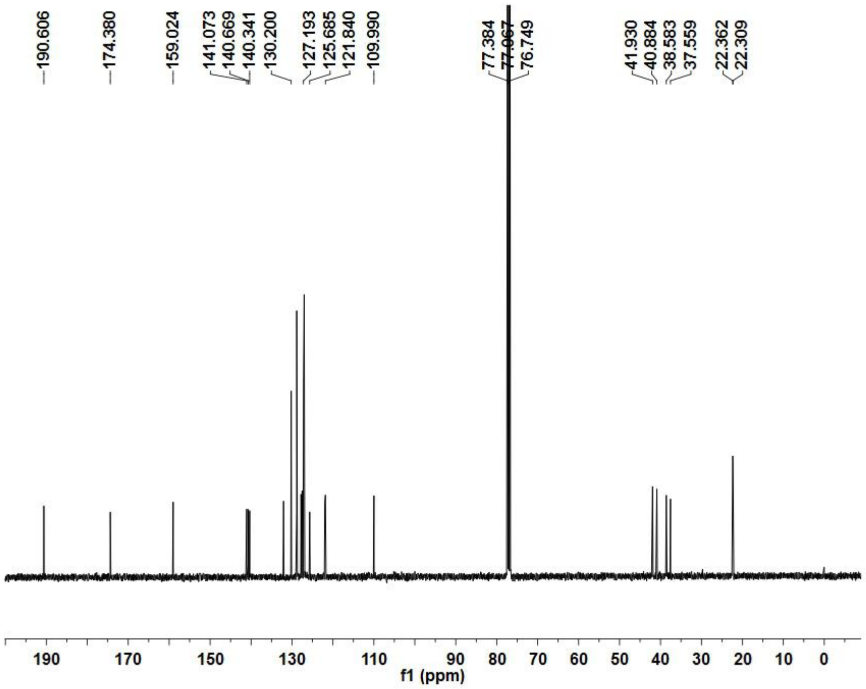

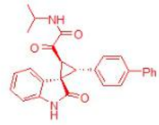

4aq

Current Data Parameters

F2 - Acquisition Parameters
DATE: 2015-01-14T02:11:33
PULPROG: zgpg30
TD: 32768
Solvent: CDCl3
NS: 512
DS: undefined
SWH: 24038.5 Hz
AQ: undefined
TE: 294.2 C

======== CHANNEL f1 ========
NUC1: 13C
P1: 9.63 usec
SFO1: undefined MHz

F2 - Processing Parameters
SI: 65536
DC: 0.05
LB: 1.00 Hz
First Point: 0.50
FT: Hyper Quadrature
Phase: Manual
Ph0: -73.61
Ph1: 76.14

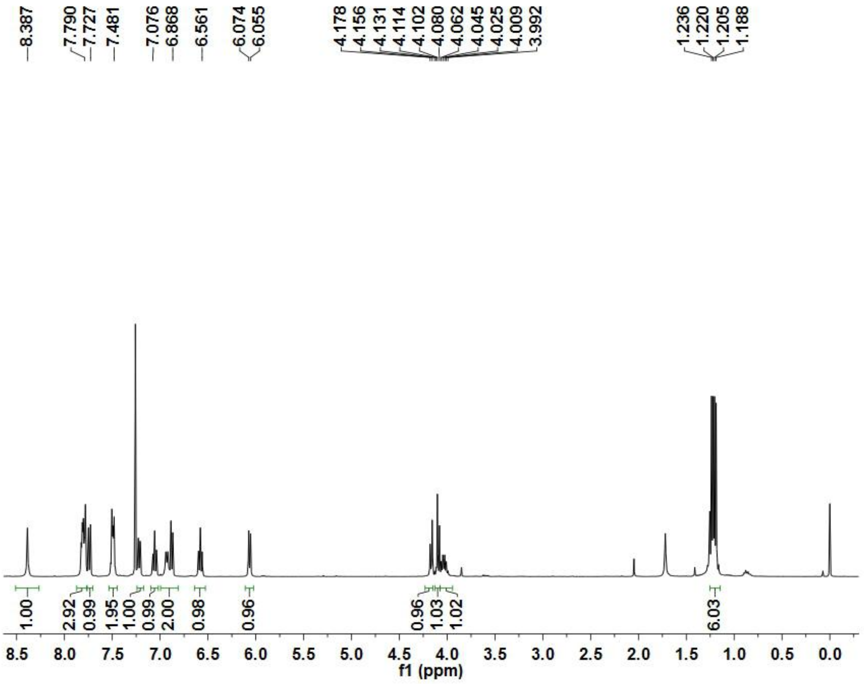

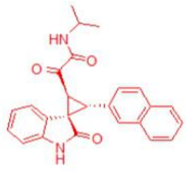

4ar

Current Data Parameters

F2 - Acquisition Parameters
DATE: 2014-11-20T21:23:04
PULPROG: zg30
TD: 32768
Solvent: CDCl3
NS: 64
DS: undefined
SWH: 8223.7 Hz
AQ: undefined
TE: 295.6 C

======== CHANNEL f1 ========
NUC1: 1H
P1: 9.93 usec
SFO1: undefined MHz

F2 - Processing Parameters
SI: 65536
DC: 0.05
LB: 0.30 Hz
First Point: 0.50
FT: Hyper Quadrature
Phase: Manual
Ph0: 89.48
Ph1: 16.00

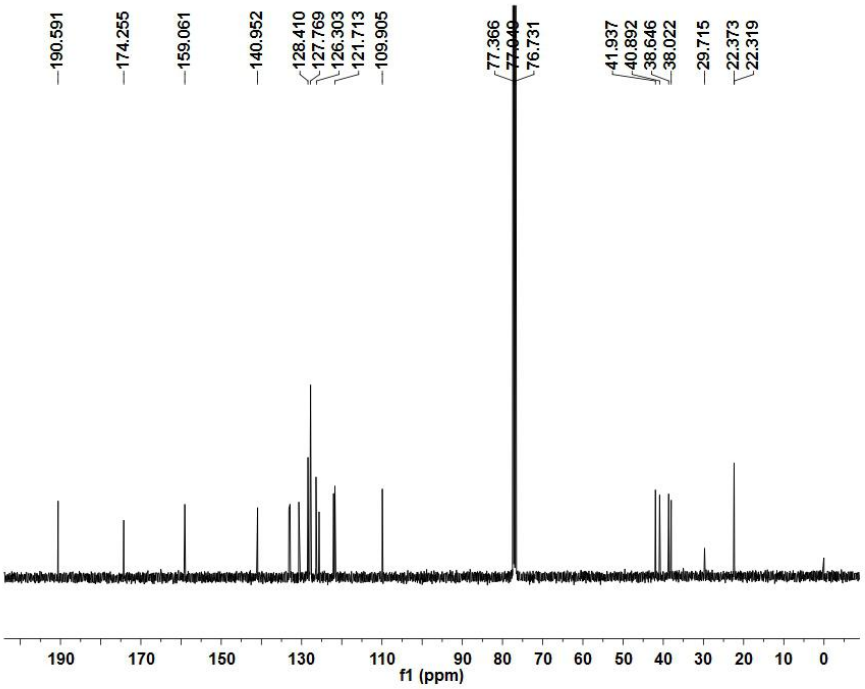

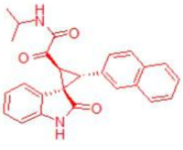

4ar

Current Data Parameters

F2 - Acquisition Parameters
DATE: 2014-11-21T01:21:33
PULPROG: zgpg30
TD: 32768
Solvent: CDCl3
NS: 512
DS: undefined
SWH: 24038.5 Hz
AQ: undefined
TE: 295.9 C

======== CHANNEL f1 ========
NUC1: 13C
P1: 9.63 usec
SFO1: undefined MHz

F2 - Processing Parameters
SI: 65536
DC: 0.05
LB: 1.00 Hz
First Point: 0.50
FT: Hyper Quadrature
Phase: Manual
Ph0: -68.42
Ph1: 63.36

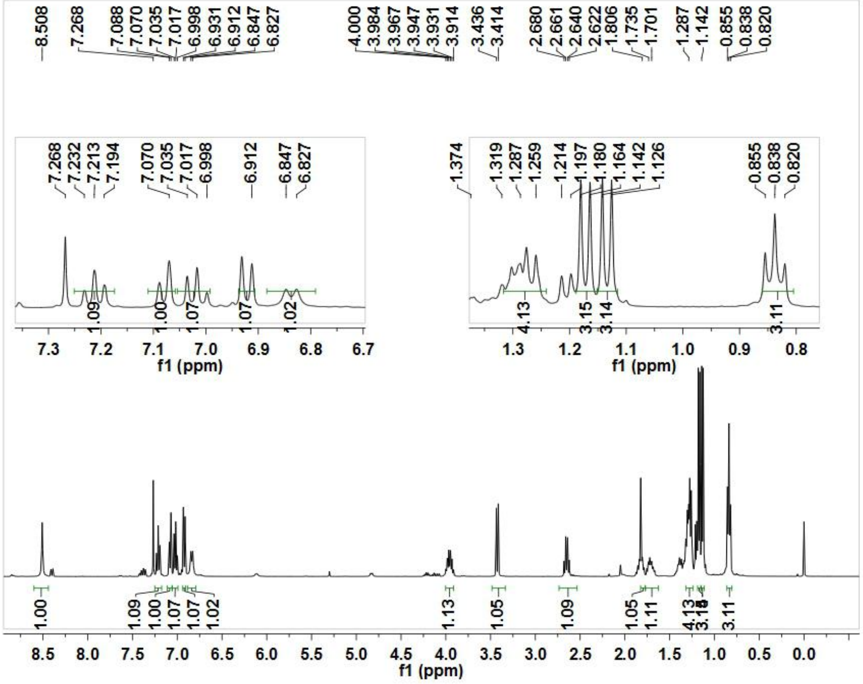

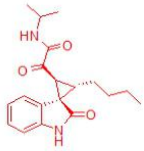

4at

Current Data Parameters

F2 - Acquisition Parameters
DATE: 2015-02-05T03:16:16
PULPROG: zg30
TD: 32768
Solvent: CDCl3
NS: 64
DS: undefined
SWH: 8223.7 Hz
AQ: undefined
TE: 293.7 C

======== CHANNEL f1 ========
NUC1: 1H
P1: 9.93 usec
SFO1: undefined MHz

F2 - Processing Parameters
SI: 65536
DC: 0.05
LB: 0.30 Hz
First Point: 0.50
FT: Hyper Quadrature
Phase: Manual
Ph0: 92.28
Ph1: 19.82

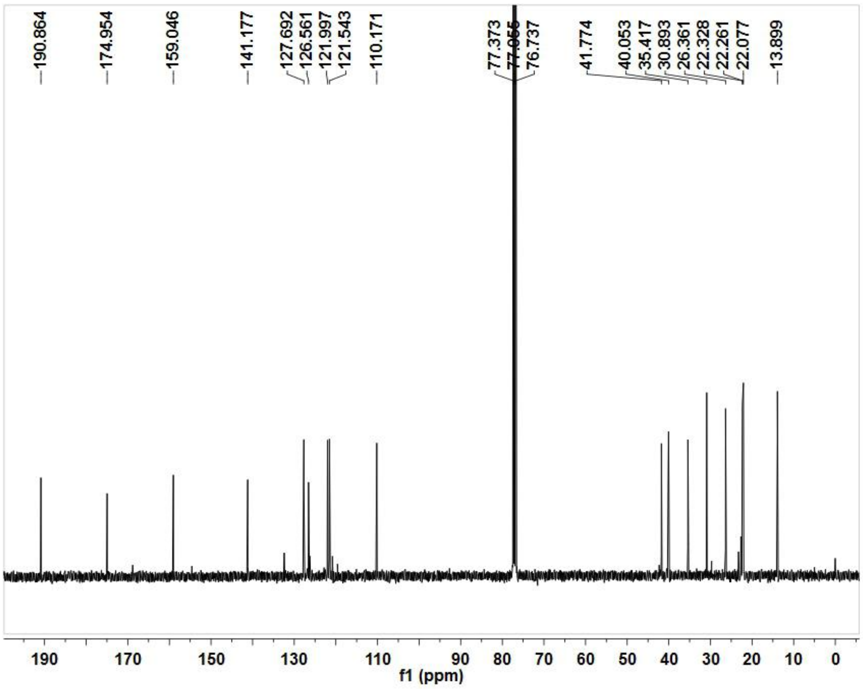

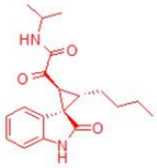

4at

Current Data Parameters

F2 - Acquisition Parameters
DATE: 2015-02-05T03:46:28
PULPROG: zgpg30
TD: 32768
Solvent: CDCl3
NS: 512
DS: undefined
SWH: 24038.5 Hz
AQ: undefined
TE: 294.2 C

======== CHANNEL f1 ========
NUC1: 13C
P1: 9.63 usec
SFO1: undefined MHz

F2 - Processing Parameters
SI: 65536
DC: 0.05
LB: 1.00 Hz
First Point: 0.50
FT: Hyper Quadrature
Phase: Manual
Ph0: -71.82
Ph1: 65.94

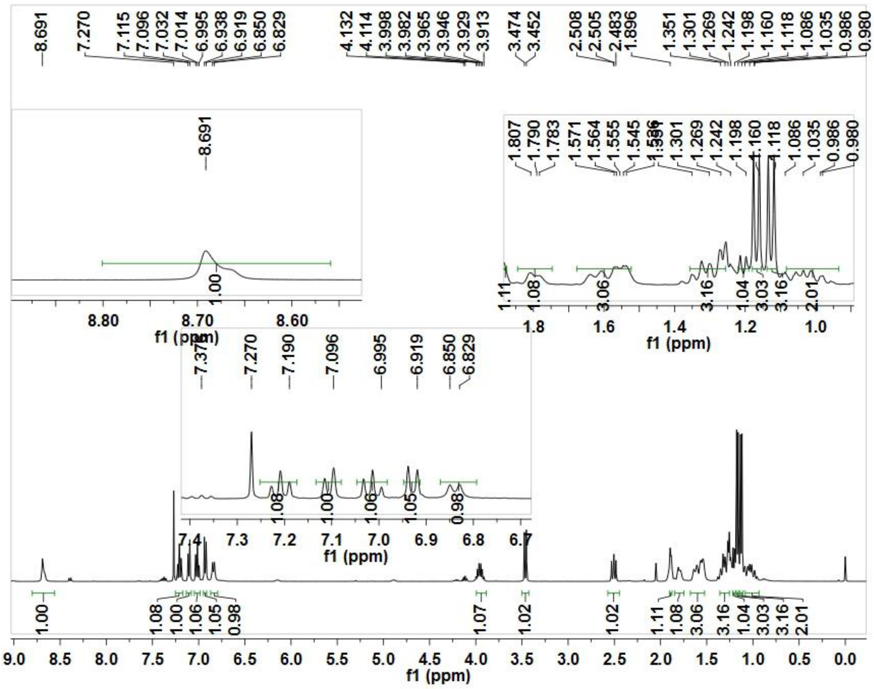

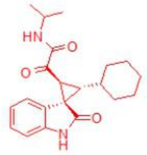

4au

Current Data Parameters

F2 - Acquisition Parameters
DATE: 2015-02-05T04:33:36
PULPROG: zg30
TD: 32768
Solvent: CDCl3
NS: 64
DS: undefined
SWH: 8223.7 Hz
AQ: undefined
TE: 293.7 C

======== CHANNEL f1 ========
NUC1: 1H
P1: 9.93 usec
SFO1: undefined MHz

F2 - Processing Parameters
SI: 65536
DC: 0.05
LB: 0.30 Hz
First Point: 0.50
FT: Hyper Quadrature
Phase: Manual
Ph0: -268.12
Ph1: 23.78

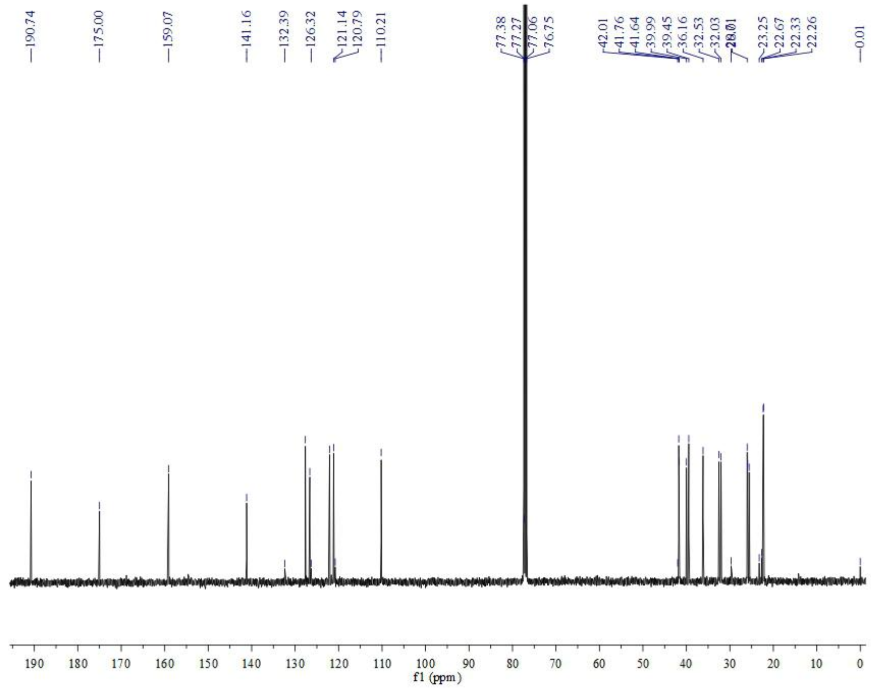

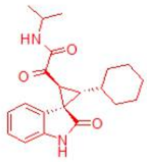

4au

Current Data Parameters

F2 - Acquisition Parameters
DATE: 2015-02-05T05:03:48
PULPROG: zgpg30
TD: 32768
Solvent: CDCl3
NS: 512
DS: undefined
SWH: 24038.5 Hz
AQ: undefined
TE: 294.2 C

======== CHANNEL f1 ========
NUC1: 13C
P1: 9.63 usec
SFO1: undefined MHz

F2 - Processing Parameters
SI: 65536
DC: 0.05
LB: 1.00 Hz
First Point: 0.50
FT: Hyper Quadrature
Phase: Manual
Ph0: -61.56
Ph1: 55.08

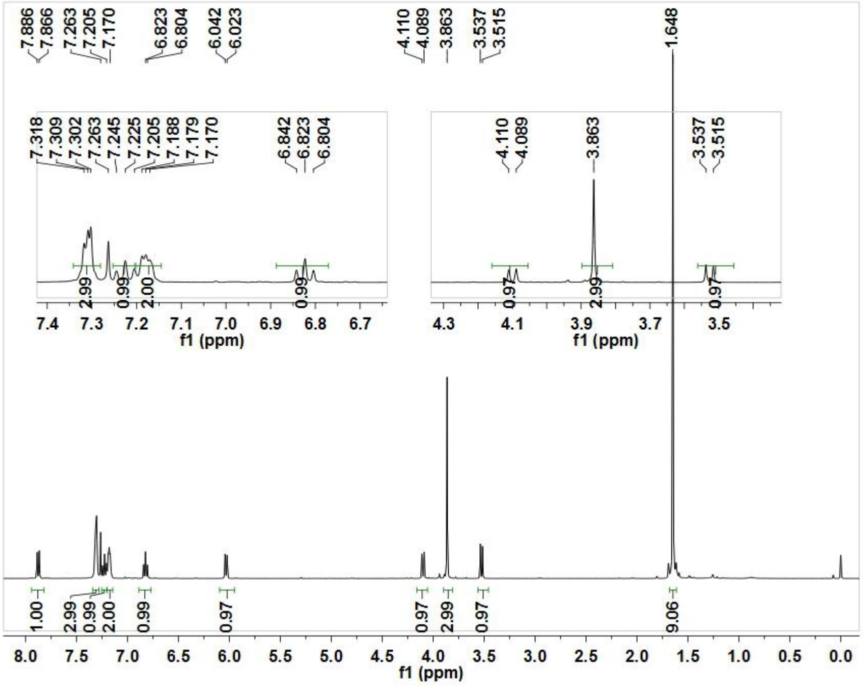

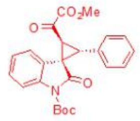

5aa

Current Data Parameters

F2 - Acquisition Parameters
DATE: 2015-01-31T03:11:13
PULPROG: zg30
TD: 32768
Solvent: CDCl3
NS: 64
DS: undefined
SWH: 8223.7 Hz
AQ: undefined
TE: 294.2 C

======== CHANNEL f1 ========
NUC1: 1H
P1: 9.93 usec
SFO1: undefined MHz

F2 - Processing Parameters
SI: 65536
DC: 0.05
LB: 0.30 Hz
First Point: 0.50
FT: Hyper Quadrature
Phase: Manual
Ph0: 93.46
Ph1: 16.52

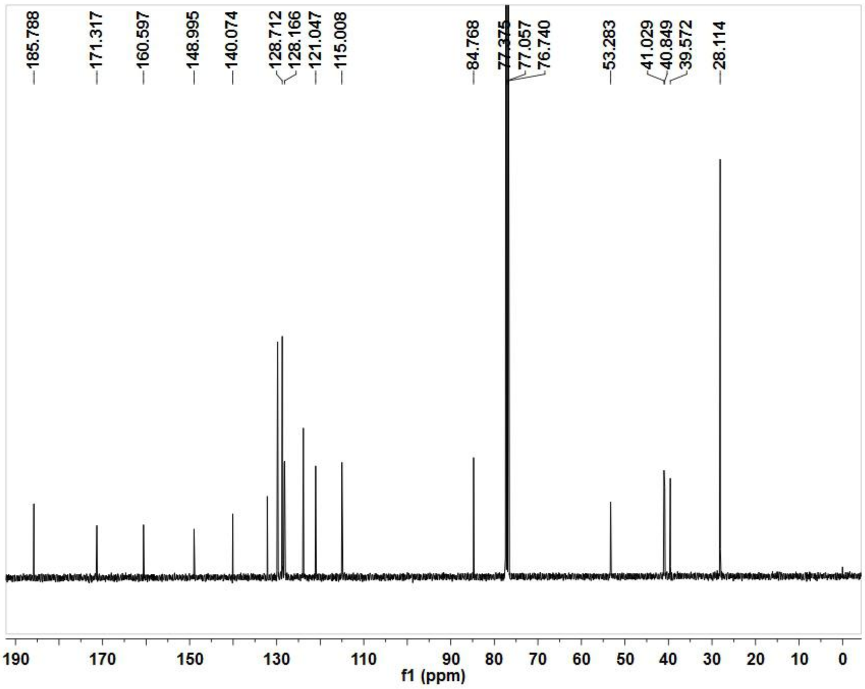

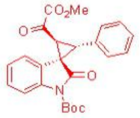

5aa

Current Data Parameters

F2 - Acquisition Parameters
DATE: 2015-01-31T03:41:23
PULPROG: zgpg30
TD: 32768
Solvent: CDCl3
NS: 512
DS: undefined
SWH: 24038.5 Hz
AQ: undefined
TE: 294.3 C

======== CHANNEL f1 ========
NUC1: 13C
P1: 9.63 usec
SFO1: undefined MHz

F2 - Processing Parameters
SI: 65536
DC: 0.05
LB: 1.00 Hz
First Point: 0.50
FT: Hyper Quadrature
Phase: Manual
Ph0: -67.88
Ph1: 67.26

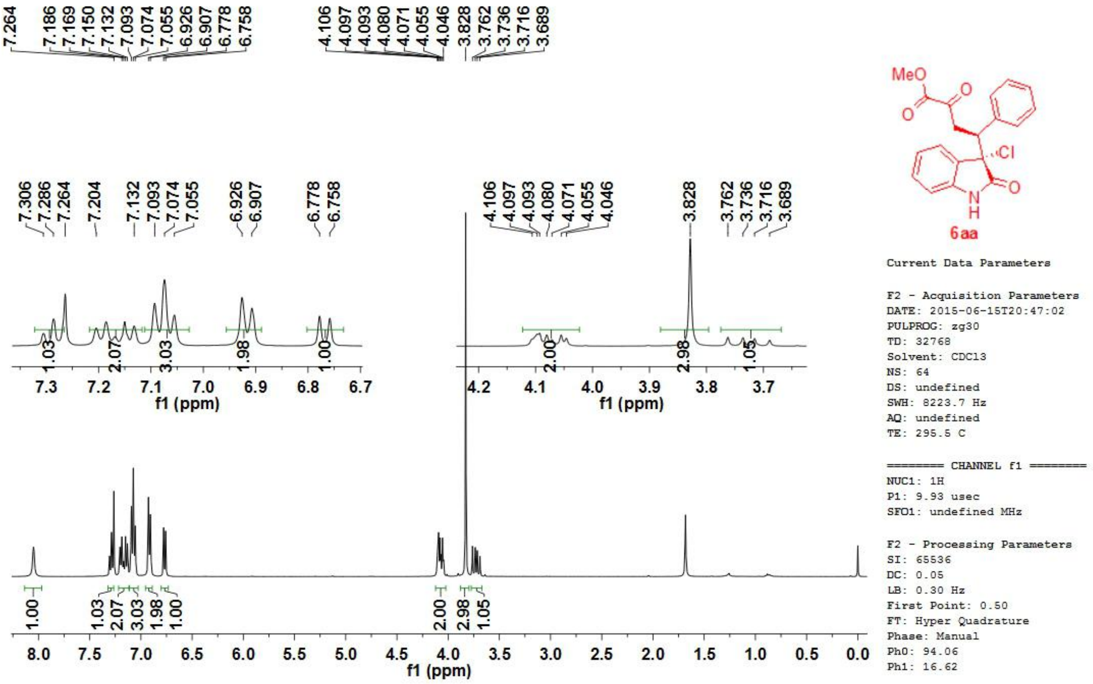
7.264 7.186 7.169 7.150 7.132 7.093 7.074 7.055 6.926 6.907 6.778 6.758
4.106 4.097 4.093 4.080 4.071 4.055 4.046 3.828 3.762 3.736 3.716 3.689
7.306 7.286 7.264 7.204 7.132 7.093 7.074 7.055 6.926 6.907 6.778 6.758
4.106 4.097 4.093 4.080 4.071 4.055 4.046 3.828 3.762 3.736 3.716 3.689
1.03 2.07 3.03 1.98 1.00
2.00 2.98 1.05
f1 (ppm)
1.00 1.03 2.07 3.03 1.98 1.00 2.00 2.98 1.05
MeO
O
O
Cl
N
H
O
6aa
Current Data Parameters
F2 - Acquisition Parameters
DATE: 2015-06-15T20:47:02
PULPROG: zg30
TD: 32768
Solvent: CDCl3
NS: 64
DS: undefined
SWH: 8223.7 Hz
AQ: undefined
TE: 295.5 C
======== CHANNEL f1 ========
NUC1: 1H
P1: 9.93 usec
SFO1: undefined MHz
F2 - Processing Parameters
SI: 65536
DC: 0.05
LB: 0.30 Hz
First Point: 0.50
FT: Hyper Quadrature
Phase: Manual
Ph0: 94.06
Ph1: 16.62

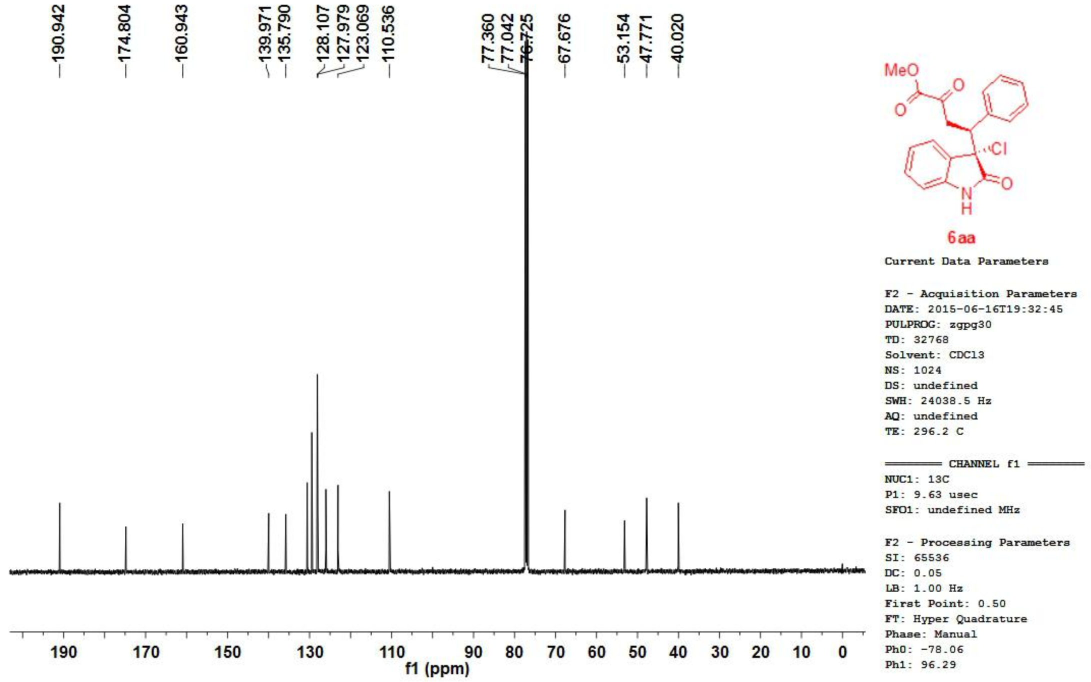
190.942 174.804 160.943 139.971 135.790 128.107 127.979 123.069 110.536 77.360 77.042 76.725 67.676 53.154 47.771 40.020
f1 (ppm)
MeO
O
O
Cl
N
H
O
6aa
Current Data Parameters
F2 - Acquisition Parameters
DATE: 2015-06-16T19:32:45
PULPROG: zgpg30
TD: 32768
Solvent: CDCl3
NS: 1024
DS: undefined
SWH: 24038.5 Hz
AQ: undefined
TE: 296.2 C
======== CHANNEL f1 ========
NUC1: 13C
P1: 9.63 usec
SFO1: undefined MHz
F2 - Processing Parameters
SI: 65536
DC: 0.05
LB: 1.00 Hz
First Point: 0.50
FT: Hyper Quadrature
Phase: Manual
Ph0: -78.06
Ph1: 96.29

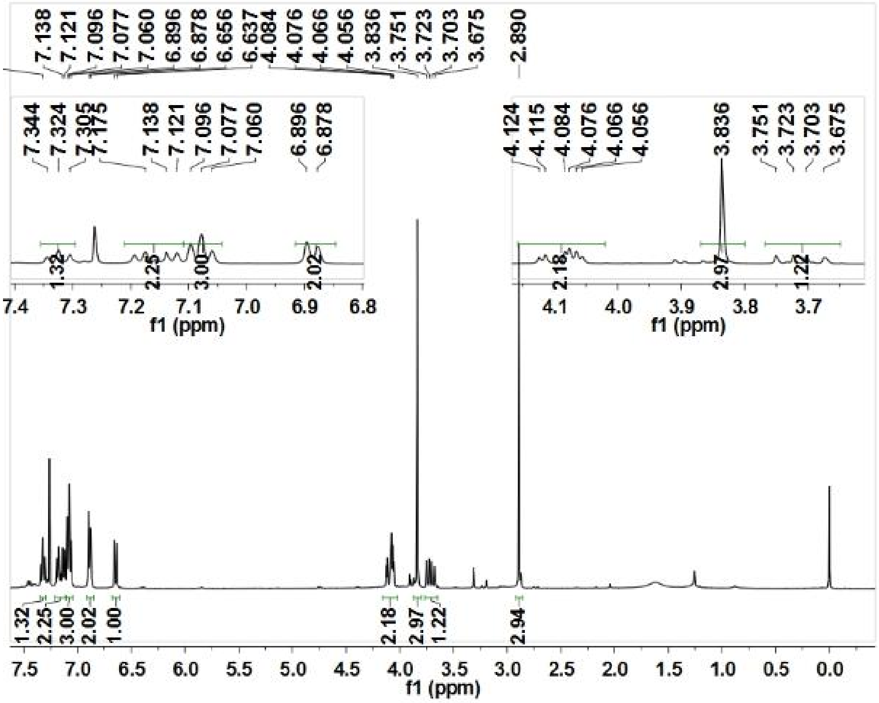

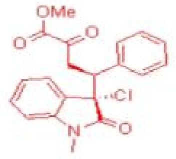

7

Current Data Parameters

F2 - Acquisition Parameters
DATE: 2017-10-22T21:02:16
PULPROG: zg30
TD: 32768
Solvent: CDCl3
NS: 64
DS: undefined
SWH: 8223.7 Hz
AQ: undefined
TE: 298.1 C

======== CHANNEL f1 ========
NUC1: 1H
P1: 9.93 usec
SFO1: undefined MHz

F2 - Processing Parameters
SI: 65536
DC: 0.05
LB: 0.30 Hz
First Point: 0.50
FT: Hyper Quadrature
Phase: Manual
Ph0: 87.36
Ph1: 20.24

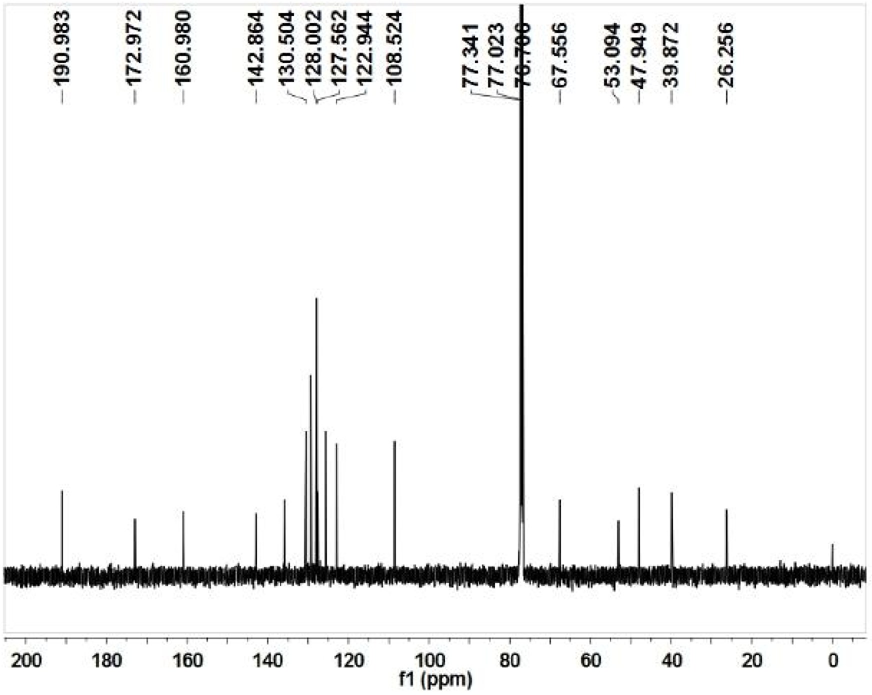

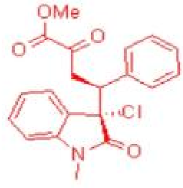

7

Current Data Parameters

F2 - Acquisition Parameters
DATE: 2017-10-22T22:01:39
PULPROG: zgpg30
TD: 32768
Solvent: CDCl3
NS: 1024
DS: undefined
SWH: 24038.5 Hz
AQ: undefined
TE: 298.5 C

======== CHANNEL f1 ========
NUC1: 13C
P1: 9.63 usec
SFO1: undefined MHz

F2 - Processing Parameters
SI: 65536
DC: 0.05
LB: 1.00 Hz
First Point: 0.50
FT: Hyper Quadrature
Phase: Manual
Ph0: -77.19
Ph1: 73.25

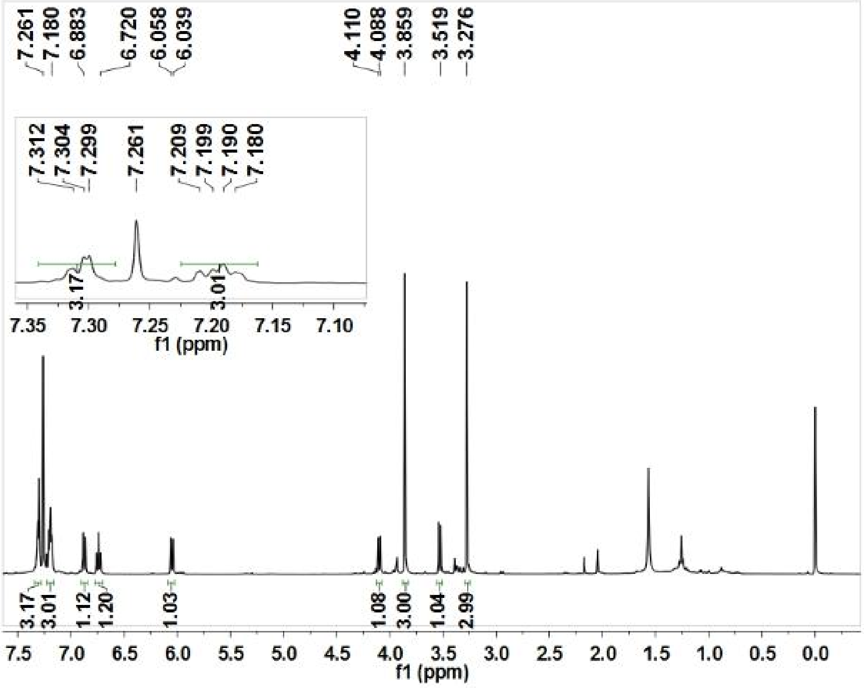

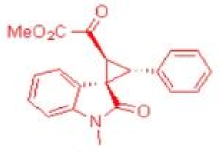

8aa

Current Data Parameters

F2 - Acquisition Parameters
DATE: 2017-10-22T22:09:50
PULPROG: zg30
TD: 32768
Solvent: CDCl3
NS: 64
DS: undefined
SWH: 8223.7 Hz
AQ: undefined
TE: 298.1 C

======== CHANNEL f1 ========
NUC1: 1H
P1: 9.93 usec
SFO1: undefined MHz

F2 - Processing Parameters
SI: 65536
DC: 0.05
LB: 0.30 Hz
First Point: 0.50
FT: Hyper Quadrature
Phase: Manual
Ph0: 90.93
Ph1: 7.30

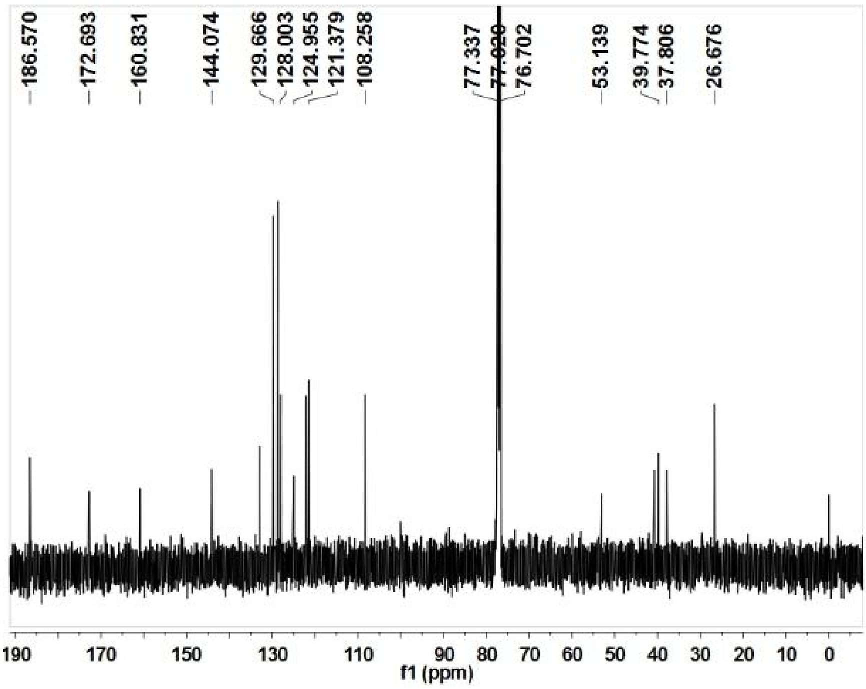

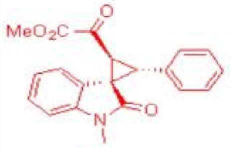

8aa

Current Data Parameters

F2 - Acquisition Parameters
DATE: 2017-10-22T23:09:14
PULPROG: zgpg30
TD: 32768
Solvent: CDCl3
NS: 1024
DS: undefined
SWH: 24038.5 Hz
AQ: undefined
TE: 298.5 C

======== CHANNEL f1 ========
NUC1: 13C
P1: 9.63 usec
SFO1: undefined MHz

F2 - Processing Parameters
SI: 65536
DC: 0.05
LB: 1.00 Hz
First Point: 0.50
FT: Hyper Quadrature
Phase: Manual
Ph0: -97.33
Ph1: 103.90

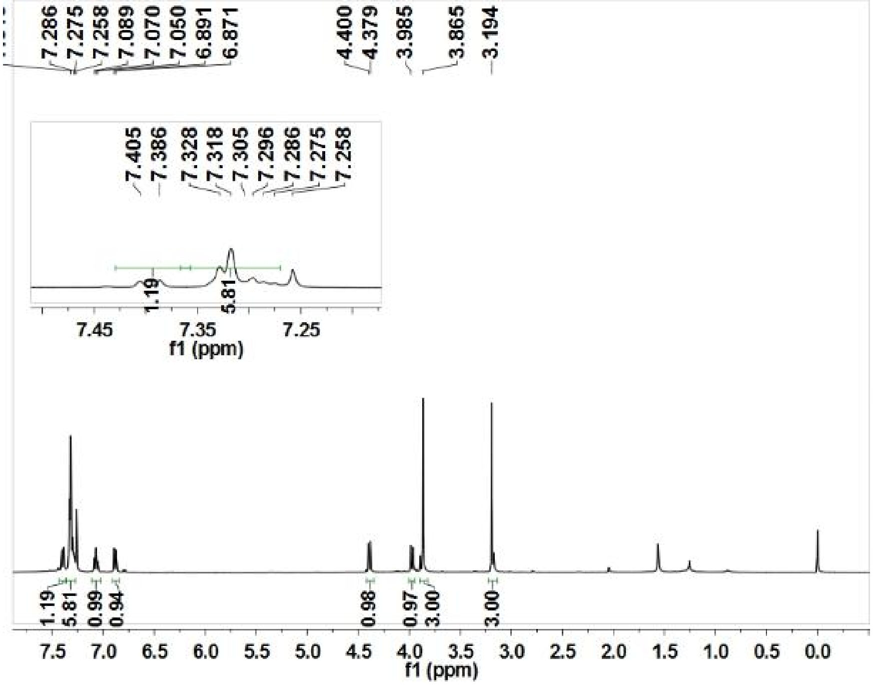

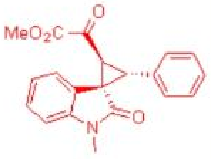

8aa'

Current Data Parameters

F2 - Acquisition Parameters
DATE: 2017-10-27T14:13:17
PULPROG: zg30
TD: 32768
Solvent: CDCl3
NS: 16
DS: undefined
SWH: 8223.7 Hz
AQ: undefined
TE: 298.2 C

======== CHANNEL f1 ========
NUC1: 1H
P1: 9.93 usec
SFO1: undefined MHz

F2 - Processing Parameters
SI: 65536
DC: 0.05
LB: 0.30 Hz
First Point: 0.50
FT: Hyper Quadrature
Phase: Manual
Ph0: 91.37
Ph1: 5.50

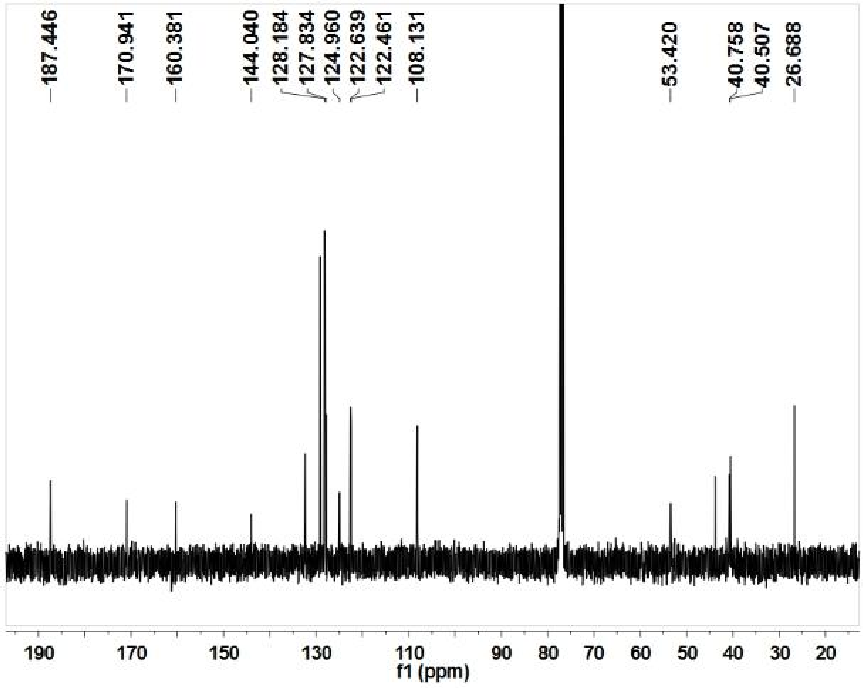

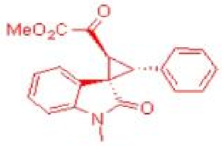

8aa'

Current Data Parameters

F2 - Acquisition Parameters
DATE: 2017-10-27T14:28:50
PULPROG: zgpg30
TD: 32768
Solvent: CDCl3
NS: 256
DS: undefined
SWH: 24038.5 Hz
AQ: undefined
TE: 298.6 C

======== CHANNEL f1 ========
NUC1: 13C
P1: 9.63 usec
SFO1: undefined MHz

F2 - Processing Parameters
SI: 65536
DC: 0.05
LB: 1.00 Hz
First Point: 0.50
FT: Hyper Quadrature
Phase: Manual
Ph0: -61.00
Ph1: 39.30
